# Burden of anxiety disorders in Colombia: subnational DALY estimates and territorial inequalities, 2016–2022

**DOI:** 10.1186/s12963-026-00510-4

**Published:** 2026-09-15

**Authors:** Oscar Alexander Gutierrez-Lesmes, Emilce Salamanca Ramos, Zulma Johana Velasco Páez

**Affiliations:** School of Public Health, University of the Llanos, Villavicencio, Colombia

**Keywords:** Anxiety disorders, Burden of disease, Disability-adjusted life years, Health status disparities, Colombia

## Abstract

**Background:**

Anxiety disorders are among the leading contributors to non-fatal health loss worldwide. However, subnational evidence on their burden and territorial distribution in Colombia remains limited. This study estimated the burden of anxiety disorders across Colombian departments and assessed temporal trends, geographic inequalities, and differences by sex and age between 2016 and 2022.

**Objective:**

To estimate the subnational burden of anxiety disorders in Colombia between 2016 and 2022 using disability-adjusted life years (DALYs), and to assess temporal trends, territorial inequalities, and differences by sex and age.

**Methods:**

A longitudinal ecological study was conducted using official national administrative databases. Disability-adjusted life years (DALYs) were estimated according to the World Health Organization Global Health Estimates framework using ICD-10 codes F40–F44. Morbidity data were obtained from the Individual Health Service Provision Records (RIPS), mortality data from the Single Registry of Affiliates (RUAF), and population denominators from the National Administrative Department of Statistics (DANE). Crude and age-standardized DALY rates were calculated by department, sex, and age group. Temporal trends were assessed using annual percent change (APC) models.

**Results:**

During 2016–2022, 3,055,505 cases and 21 deaths attributable to anxiety disorders were identified, corresponding to an estimated 1,266,489 DALYs. The national age-standardized DALY rate increased from 177.9 per 100,000 population in 2016 to 687.4 per 100,000 in 2022, with an APC of 25.41% (95% CI 19.48–31.63). Women consistently showed higher burden than men throughout the study period. Marked territorial inequalities were identified, with the highest burden concentrated in departments of the Andean region, particularly Risaralda, Caldas, and Bogotá, D.C. whereas lower rates were observed in Amazonian and Orinoquía territories.

**Conclusions:**

Anxiety disorders accounted for a substantial and increasing estimated burden among individuals recorded in the Colombian health system during 2016–2022, with marked territorial heterogeneity and important differences by sex and age. These findings provide subnational evidence to support population health monitoring and burden-of-disease assessment, while recognizing that administrative health records reflect treated morbidity and healthcare utilization rather than population prevalence.

**Supplementary Information:**

The online version contains supplementary material available at https://doi.org/10.1186/s12963-026-00510-4.

## Introduction

Anxiety disorders constitute one of the most prevalent and disabling groups of mental illnesses globally. They are characterized by excessive fear and persistent worry, with physical and cognitive symptoms that affect daily functioning, productivity, and quality of life [1].

Globally, the Global Burden of Disease Study 2023 estimated 171 million (95% UI 127–228 million) DALYs attributable to mental disorders in 2023, representing 6.1% (95% UI 4.8–7.6) of all-cause DALYs worldwide. Mental disorders were the leading cause of years lived with disability (YLDs), accounting for 17.3% (95% UI 14.8–20.6) of global YLDs. Anxiety disorders were the leading contributor to mental-disorder DALYs and ranked 11th among the 304 Level 4 causes in the GBD hierarchy [2]. Evidence also indicates that anxiety disorders have experienced a sustained increase in burden over recent decades, with projections suggesting that this burden will continue to rise in the coming years [3–5]

In Latin America, the burden of anxiety disorders acquires particular relevance in contexts characterized by persistent social inequalities, violence, forced displacement, accelerated urbanization, and limitations in timely access to mental health services [6–8].

In Colombia, anxiety disorders constitute an important public health problem. The 2015 National Mental Health Survey, which included 10,870 adults, reported a 12-month prevalence of any of the assessed depressive or anxiety disorders of 5.1% (95% CI 4.3–6.0) among adults aged 18–44 years and 2.3% (95% CI 1.8–3.0) among those aged 45 years or older [9]. More recently, a national burden-of-disease analysis for 2022 estimated 156,099.6 DALYs (95% UI 140,954.4–171,244.8) attributable specifically to anxiety disorders, corresponding to a rate of 302.0 DALYs per 100,000 population (95% UI 272.7–331.3); anxiety disorders accounted for 52.7% of the total DALYs attributable to mental and behavioral disorders in that analysis [10]. Despite this evidence, information on subnational temporal trends, territorial inequalities, and differences by sex and age remains limited.

In this context, estimating disability-adjusted life years (DALYs) at the departmental level makes it possible to understand heterogeneity in the burden of disease, identify differences between departments, as well as differences by sex and age. Therefore, the objective of this study was to estimate the burden of disease attributable to anxiety disorders in the departments of Colombia during the period 2016–2022, to characterize the subnational distribution of the burden across Colombian departments.

## Materials and methods

### Study design

A longitudinal ecological study with temporal and spatial components was conducted to estimate the burden of anxiety disorders in Colombia at the departmental level during the period 2016–2022. The study followed the methodological principles of the *Global Health Estimates* of the World Health Organization, conceptually consistent with the approaches used in *Global Burden of Disease* studies [11].

### Data sources

Official publicly available secondary data sources were used. Morbidity data were obtained from the Integrated Social Protection Information System (SISPRO), specifically the Individual Health Service Provision Records (RIPS). Mortality data were obtained from the non-fetal death registry of the Single Registry of Affiliates (RUAF). Population denominators for rate calculations were obtained from the official population projections by year, sex, age, and department published by the National Administrative Department of Statistics (DANE).

The study period was restricted to 2016–2022 because 2022 was the most recent year for which morbidity (RIPS), mortality (RUAF), and official population data were fully consolidated and available for integrated DALY estimation at the time of analysis. Restricting the study to a complete observation period ensured temporal comparability and methodological consistency across all data sources.

### Study population and case definition

All records corresponding to individuals aged 18 years or older residing in Colombia during the period 2016–2022 were included. Cases of anxiety disorders were identified using ICD-10 codes F40–F44, consistent with the disease grouping adopted by the WHO Global Health Estimates framework used for burden-of-disease estimation. Information was disaggregated by calendar year, sex, age group, and department of residence.

### Data processing and analysis

Administrative health records obtained from SISPRO were processed using Python for database cleaning, validation, harmonization, and consolidation. Morbidity and mortality datasets were integrated into analytical databases used for the estimation of burden of disease indicators. Data cleaning included verification of duplicate records, standardization of department names and administrative codes, validation of age and sex variables, consistency checks across years, and harmonization of variable formats before database integration. No missing data were identified for the variables required for DALY estimation after data cleaning and validation; therefore, no imputation procedures were necessary.

### Burden of disease estimation

Disability-adjusted life years (DALYs) were estimated according to the methodological framework of the World Health Organization Global Health Estimates (WHO-GHE) [11], in which the burden of disease is quantified as the sum of Years of Life Lost (YLLs) due to premature mortality and Years Lived with Disability (YLDs).

Morbidity data were obtained from the Colombian Individual Records of Health Service Provision (RIPS) available through the Integrated Social Protection Information System (SISPRO). Records were linked using the anonymized individual identifier available in RIPS to identify unique individuals. Multiple records corresponding to the same individual with the same ICD-10 diagnosis within the same calendar year were consolidated; consequently, each individual was counted only once per diagnosis per year. Therefore, the morbidity input used for YLD estimation was based on unique individuals rather than healthcare encounters, preventing overestimation due to repeated consultations. Because RIPS is an administrative healthcare utilization database, these records represent treated morbidity rather than the true population prevalence of anxiety disorder.

Years Lived with Disability (YLDs) were estimated using the WHO Global Health Estimates (WHO-GHE) prevalence-based framework [11]. Treated morbidity cases identified from the Colombian Individual Health Services Registry (RIPS) were used as the morbidity input and combined with the corresponding disability weights to estimate YLDs. Disability weights were obtained from the Global Burden of Disease Study 2021 [12], which provides standardized severity weights for each health state. Because the WHO-GHE prevalence-based framework was applied, no assumptions regarding disease duration were required for YLD estimation. Consistent with the WHO-GHE methodology, neither age-weighting nor time discounting was applied.

Years of Life Lost (YLLs) were estimated by multiplying the number of deaths attributed to anxiety disorders, obtained from the Colombian Vital Statistics System (RUAF), by the remaining standard life expectancy at the age of death using the WHO-GHE standard expected life table (standard loss function) [11].

### Disability-Adjusted Life Years (DALYs)

The Disability-Adjusted Life Years (DALYs) indicator combines premature mortality and disability into a single measure. It is composed of the sum of Years of Life Lost (YLLs) and Years Lived with Disability (YLDs). YLLs represent the years of life lost when a person dies before reaching a theoretical life expectancy. YLDs quantify health loss associated with disability across different functional domains.

The calculation formula was as follows:

$${\rm{}}DALYs = YLD + YLL$$

$$YLDs = \sum\nolimits_c {{W_c}} {P_c}$$

$$YLLs = \sum\nolimits_x {{D_x}} {e_x}^*$$

Where (*W*_*c*_) denotes the disability weight for condition c, (Pc) denotes the morbidity input, operationalized as the annual number of unique individuals with anxiety disorders recorded in the RIPS administrative database. The term (*D*_*x*_) denotes the number of deaths occurring at age x, and $(e_x^*$) denotes the remaining standard life expectancy at age x.

For the calculation of Years of Life Lost, the number of deaths whose underlying cause corresponded to anxiety disorders was used. For the calculation of Years Lived with Disability, unique individuals with a principal diagnosis of anxiety disorders recorded in the RIPS database were used as the morbidity input.

### Statistical analysis

Descriptive statistics were used to summarize the characteristics of individuals diagnosed with anxiety disorders. Mean and median ages were calculated separately for men and women using the individual age recorded in the morbidity database. Corresponding 95% confidence intervals (95% CIs) for the mean age were estimated using the standard error of the mean.

Crude and age-standardized annual and period Disability-Adjusted Life Year (DALY) rates were calculated for the period 2016–2022. Estimates were based on DALY databases and population projections from the National Administrative Department of Statistics (DANE).

For age-standardized rates, the direct standardization method was applied using the World Health Organization (WHO) Standard Population (2000–2025) as the reference population to control for differences in age structure between departments.

For the overall 2016–2022 period, DALYs and population denominators were pooled across years. The crude period rate was calculated by dividing the total DALYs accumulated during the study period by the sum of the corresponding annual adult population denominators and multiplying by 100,000. For the period age-standardized rate, DALYs and population denominators were first pooled within each age group across the seven years. Age-specific period rates were then calculated and standardized using the direct method with the WHO Standard Population.

Ninety-five percent confidence intervals (95% CIs) were calculated for crude and age-standardized rates using variance estimates derived from the direct standardization method, based on a Poisson approximation for the underlying event rates. Confidence limits were estimated using the normal approximation, an approach widely used in burden-of-disease studies to quantify statistical uncertainty around standardized rates. Because the normal approximation is symmetric, lower confidence limits may occasionally become negative in departments with small populations or few observed events. These values reflect the high statistical uncertainty associated with sparse data rather than epidemiologically meaningful negative DALY rates.

To assess temporal trends in age-standardized DALY rates, the Annual Percent Change (APC) was estimated using log-linear regression models. The analysis used annual age-standardized rates by department as the dependent variable and calendar year as the independent variable.

The APC was interpreted as the average annual percentage change in the age-standardized rate. Statistical significance was established according to whether the 95% confidence interval included the null value (0%). A single-trend model was selected, consistent with methodological recommendations for short time series [13]. Joinpoint regression was not applied because the study period comprised only seven annual observations (2016–2022), providing insufficient statistical power to reliably identify changes in trend over time. Therefore, a single log-linear regression model was considered the most appropriate approach for estimating the overall annual trend across the study period. Given the limited length of the time series, APC estimates should be interpreted with caution, as they may be influenced by interannual variation and contextual events, including the COVID-19 pandemic. Additionally, trend analysis was conducted at the departmental level by estimating the APC and corresponding 95% confidence intervals for each department.

### Software and reproducibility

Statistical analyses were conducted in Python 3.12. Data visualization and epidemiological figures were generated using Matplotlib. Geospatial analyses and cartographic visualization were performed using QGIS version 3.44.11 (Solothurn). All data processing and statistical analyses were implemented using reproducible Python scripts to ensure analytical consistency and reproducibility.

### Spatial analysis

Spatial classification was performed using the *Natural Breaks (Jenks)* classification algorithm. This method identifies optimal breakpoints in the data distribution by minimizing within-class variance while simultaneously maximizing differences between classes [14].

For spatial representation, the departmental vector layer from the National Geostatistical Framework published by the National Administrative Department of Statistics was used [15].

### Bias control and data quality

Studies based on administrative records may be affected by differences in access to health services, underreporting, and diagnostic variability. To minimize these potential sources of bias, all morbidity cases recorded across the entire Colombian network of healthcare provider institutions were included, regardless of the level of care, together with all registered non-fetal deaths from the national vital statistics system.

Differences in population age structure between departments were controlled through direct age standardization. In accordance with the World Health Organization Global Health Estimates (WHO-GHE) methodology, deaths were attributed exclusively to the underlying cause of death to ensure mutually exclusive attribution of mortality across disease categories and to prevent double counting of Years of Life Lost (YLLs). This procedure standardizes mortality attribution for burden-of-disease estimation but does not constitute a formal competing-risk analysis. Additionally, potential underreporting, diagnostic coding errors, differential access to healthcare services, and territorial heterogeneity in diagnostic capacity remain inherent limitations of administrative health databases and were considered when interpreting the findings.

### Ethical considerations

This research was classified as no-risk according to Resolution 8430 of 1993 of the Colombian Ministry of Health, as it exclusively used anonymized, aggregated, and publicly available secondary data sources, without direct intervention on individuals or access to identifiable personal information.

The analyzed data were obtained from official information systems administered by Colombian governmental institutions for purposes of health care delivery, epidemiological surveillance, health system management, and public policy development. These systems are regulated by national legal frameworks that mandate the reporting of health events by healthcare providers and competent authorities. Consequently, the databases are publicly available for research, epidemiological analyses, and public health evaluation.

Because the study used secondary anonymized administrative databases and did not involve identifiable personal information or direct participant contact, individual informed consent was not required. This approach was described in the approved research protocol and is consistent with Guideline 12 of the International Ethical Guidelines for Health-related Research Involving Humans issued by the Council for International Organizations of Medical Sciences (CIOMS).

The research protocol received ethical approval from the Bioethics Committee of Universidad de los Llanos (Approval Act No. 002, May 3, 2022).

## Results

During the period 2016–2022 in Colombia, 3,055,505 cases of anxiety disorders were recorded, of which 69.4% corresponded to women. In addition, 21 deaths were documented, including 14 among women (Supplementary Table S1).

Among individuals diagnosed with anxiety disorders, the mean age was 44.78 years (95% CI 44.74–44.80) in men and 46.90 years (95% CI 46.88–46.93), with median ages of 43 and 47 years, respectively.

The estimated burden of disease based on recorded morbidity showed a sustained increase, both in absolute and relative terms. In total, 1,266,489 Disability-Adjusted Life Years (DALYs) were estimated during the study period. The cumulative age-standardized rate was 439.9 per 100,000 inhabitants (95% CI 420.4–459.4), whereas the cumulative crude rate reached 521.2 per 100,000 (95% CI 520.3–522.1). Overall, Years Lived with Disability (YLDs) accounted for 99.95% of total DALYs, whereas Years of Life Lost (YLLs) contributed only 0.05%, indicating that the burden of anxiety disorders in Colombia was driven almost entirely by disability rather than premature mortality.

**Fig. 1:**
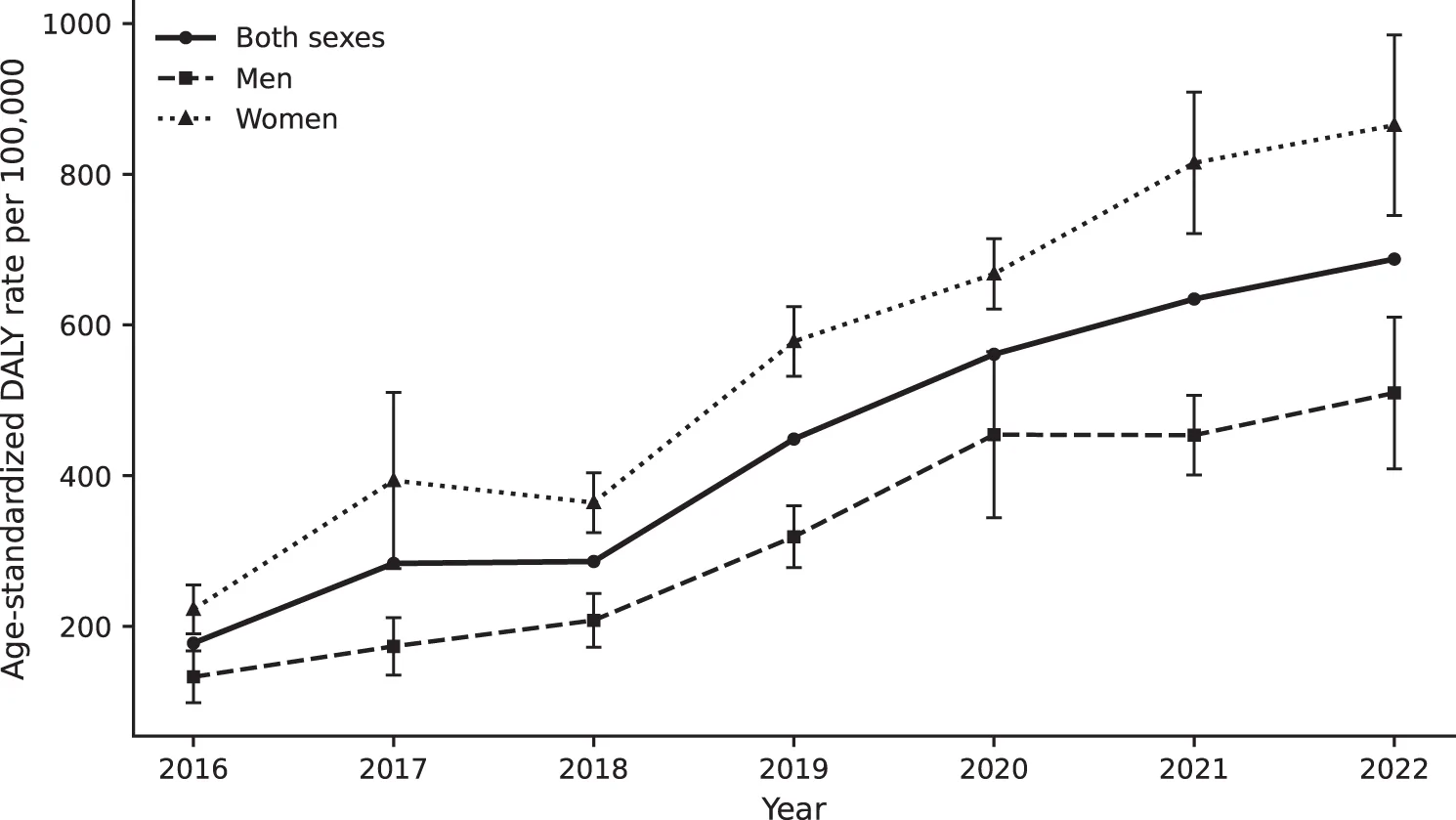
Temporal trends in age-standardized DALY rates for anxiety disorders in Colombia by sex, 2016–2022. Temporal trends in age-standardized DALY rates for anxiety disorders in Colombia by sex, 2016–2022. Solid line indicates both sexes; dashed line indicates men; dotted line indicates women. Vertical bars represent 95% confidence intervals for sex-specific estimates

In the temporal analysis, the age-standardized rate increased from 177.9 per 100,000 (95% CI 154.2–201.5) in 2016 to 687.4 per 100,000 (95% CI 609.2–765.6) in 2022 (Fig. 1), corresponding to an Annual Percent Change (APC) of 25.41% (95% CI 19.48–31.63) (Table 1). The annual series showed a continuous upward trajectory throughout the study period (Fig. 1).

**Table 1:** Department-level trends in age-standardized DALY rates for anxiety disorders in Colombia, 2016–2022

Department	APC % (95% CI)	Age-standardized rate 2016 (95% CI)	Age-standardized rate 2022 (95% CI)	Period age-standardized rate (95% CI)
Risaralda	34.65 (19.81–51.33)	351.1 (198.7–503.5)	1417.4 (1139.1–1695.7)	918.6 (831.0–1006.1)
Santander	33.19 (23.82–43.27)	280.4 (187.6–373.2)	1401.5 (1200.4–1602.6)	860.3 (800.3–920.2)
Archipiélago^a^	90.26 (−2.72–272.10)	10.1 (5.5–14.6)	1221.7 (−794.8–3238.3)	422.2 (−26.3–870.7)
Bolívar	37.03 (23.57–51.95)	115.1 (52.6–177.7)	1168.5 (953.1–1383.9)	521.2 (467.4–575.0)
Sucre	27.03 (12.89–42.95)	157.2 (40.3–274.0)	1159.8 (852.1–1467.5)	499.8 (422.4–577.2)
Atlántico	21.07 (17.02–25.25)	383.6 (257.9–509.2)	1086.0 (901.1–1270.8)	650.5 (595.5–705.5)
Valle del Cauca	25.95 (18.22–34.19)	317.4 (249.0–385.8)	1076.2 (962.2–1190.2)	747.9 (710.9–784.8)
Caldas	30.75 (15.49–48.02)	231.4 (130.2–332.6)	1037.3 (817.3–1257.2)	831.0 (754.8–907.3)
Chocó	89.70 (38.95–159.00)	19.1 (−25.3–63.4)	1015.6 (587.7–1443.4)	244.0 (165.5–322.5)
Bogotá, D.C.	17.29 (6.80–28.81)	395.2 (323.0–467.5)	938.4 (840.0–1036.8)	864.4 (827.2–901.7)
Tolima	28.57 (21.06–36.54)	193.2 (105.5–281.0)	789.3 (623.2–955.5)	537.2 (484.5–589.9)
Arauca	79.34 (47.08–118.68)	15.2 (12.1–18.4)	760.4 (190.0–1330.8)	322.5 (184.1–461.0)
Quindío	20.61 (8.59–33.96)	334.5 (139.8–529.2)	726.5 (479.9–973.2)	645.1 (554.7–735.6)
Meta	36.87 (12.97–65.83)	205.8 (50.9–360.6)	723.6 (465.2–982.1)	449.7 (370.4–528.9)
Boyacá	36.86 (25.59–49.15)	109.6 (48.8–170.4)	691.1 (538.6–843.5)	431.7 (384.8–478.7)
Cesar	17.23 (8.87–26.24)	243.7 (74.0–413.3)	676.2 (425.6–926.8)	509.7 (424.0–595.3)
Córdoba	33.25 (28.58–38.08)	108.7 (38.4–179.1)	673.5 (511.5–835.5)	303.2 (261.0–345.4)
Magdalena	33.15 (23.35–43.73)	148.4 (38.4–258.3)	672.5 (448.0–896.9)	357.5 (296.2–418.8)
Antioquia	13.60 (2.00–26.53)	399.0 (324.1–474.0)	662.6 (579.3–745.9)	728.1 (694.3–761.8)
Cauca	30.16 (24.83–35.72)	155.5 (71.3–239.7)	653.0 (477.4–828.6)	384.5 (333.3–435.8)
Huila	24.01 (8.49–41.75)	136.2 (40.9–231.5)	611.9 (403.1–820.8)	500.4 (427.4–573.3)
Cundinamarca	16.03 (10.01–22.39)	274.4 (185.5–363.2)	595.7 (479.6–711.9)	500.6 (459.0–542.2)
Norte de Santander	30.70 (18.14–44.61)	122.3 (43.0–201.6)	588.7 (423.5–753.9)	417.8 (363.3–472.2)
Caquetá	19.64 (−9.18–57.60)	181.3 (−64.8–427.5)	487.4 (122.4–852.4)	217.0 (125.5–308.5)
Guaviare	73.41 (44.80–107.67)	10.3 (5.5–15.0)	436.5 (−519.3–1392.3)	93.2 (−43.3–229.8)
Casanare	−11.49 (−29.78–11.56)	537.0 (35.4–1038.6)	368.3 (50.3–686.3)	474.7 (312.9–636.4)
La Guajira	31.24 (10.58–55.76)	42.0 (−24.4–108.4)	340.0 (100.4–579.6)	202.1 (135.2–269.0)
Nariño	9.70 (−4.48–26.00)	225.5 (141.1–309.9)	302.5 (200.9–404.0)	426.2 (380.4–472.0)
Putumayo	9.20 (−11.19–34.27)	142.0 (−67.1–351.0)	218.4 (−34.5–471.2)	279.7 (167.3–392.2)
Amazonas	21.11 (8.66–34.98)	13.2 (6.9–19.6)	61.1 (49.5–72.8)	37.1 (33.5–40.7)
Vaupés	57.66 (25.78–97.63)	3.9 (−1.2–9.1)	52.0 (35.4–68.6)	20.8 (16.7–24.9)
Guainía	64.62 (21.33–123.35)	6.0 (0.4–11.5)	40.6 (28.6–52.5)	27.2 (23.3–31.1)
Vichada	65.12 (−4.01–184.07)	1.6 (−0.2–3.4)	29.6 (22.9–36.2)	90.0 (−100.8–280.9)

^a^Archipiélago de San Andrés, providencia y Santa Catalina

Department-level annual percentage change (APC), age-standardized rates in 2016 and 2022, and period age-standardized rates for anxiety disorders across Colombian departments. Rates are expressed per 100,000 population.

Sex-stratified analysis showed a higher burden of disease in women throughout the entire study period. The cumulative age-standardized rate was 558.1 per 100,000 inhabitants (95% CI 528.2–587.9) in women, compared with 321.7 per 100,000 (95% CI 296.7–346.7) in men. This difference remained consistent across all years analyzed.

In 2022, the rates reached 865.0 per 100,000 (95% CI 745.3–984.7) in women and 509.8 per 100,000 (95% CI 409.1–610.4) in men (Fig. 1).

Regarding temporal trends, both sexes showed statistically significant increases over time, with an APC of 27.18% (95% CI 21.00–33.68) in men and 24.49% (95% CI 17.81–31.55) in women, indicating sustained growth in both groups, with a higher relative rate of increase among men (Supplementary Table S2).

### Geographic inequalities and subnational variation

**Fig. 2:**
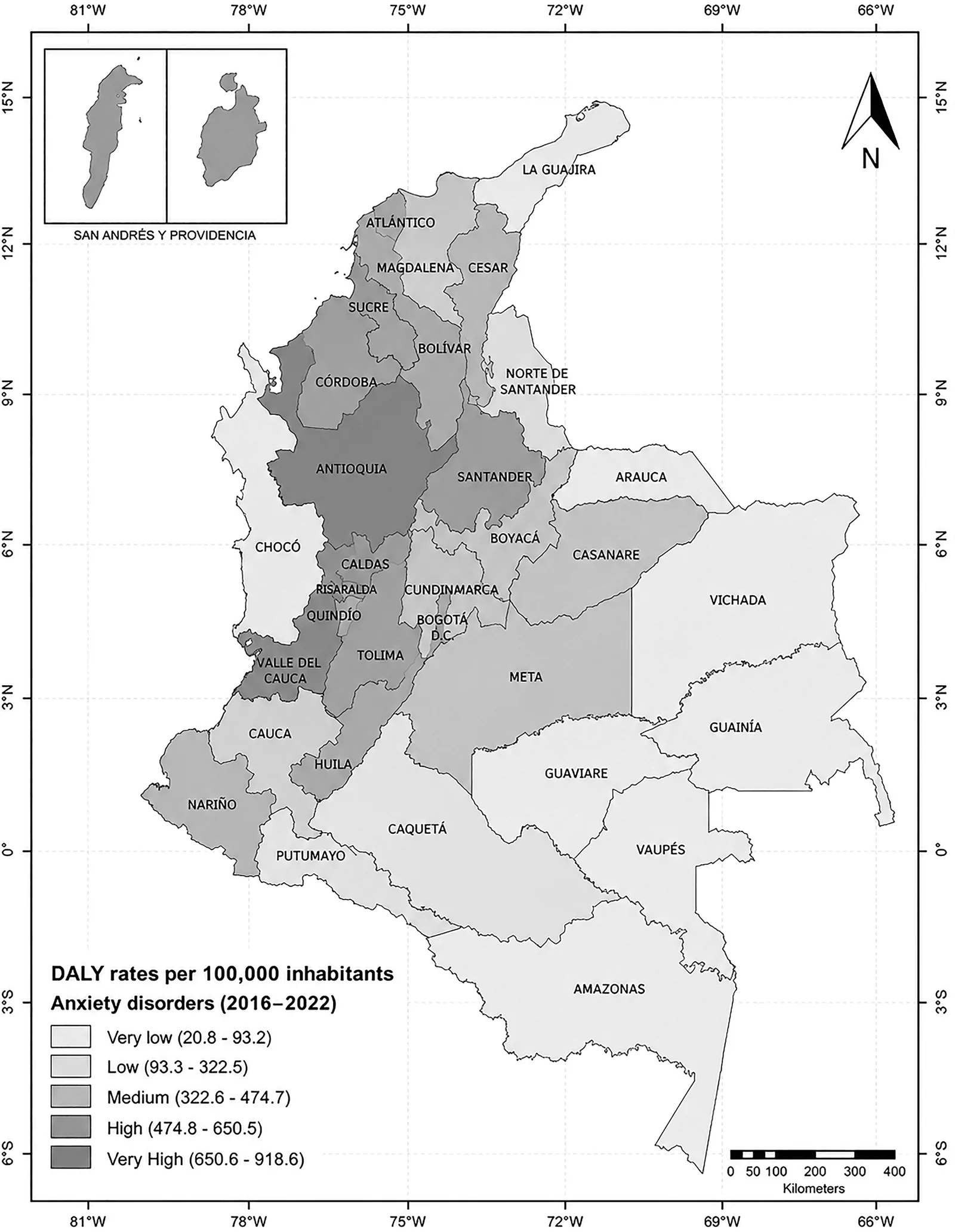
Geographic distribution of age-standardized DALY rates for anxiety disorders across departments of Colombia, 2016–2022. Geographic distribution of age-standardized DALY rates for anxiety disorders across departments of Colombia, 2016–2022. Higher values indicate greater burden of disease. Rates are expressed per 100,000 population

The burden of disease showed marked territorial heterogeneity, both in level and in the dynamics of change (Table 1). The spatial distribution of the burden of disease revealed a clearly structured geographic pattern, particularly in the Coffee Region and adjacent departments, where the highest burden levels in the country were identified. In contrast, the lowest values were located in the Amazon and Orinoquía regions, configuring a center-periphery territorial gradient and indicating marked territorial inequality (Fig. 2).

A comparative summary of cumulative age-standardized DALY rates, annual percentage change (APC) estimates, corresponding 95% confidence intervals, and trend classification for all departments is presented in Supplementary Table S3, facilitating direct comparison of temporal patterns across territories

The magnitude of territorial inequality was considerable, with differences greater than one order of magnitude between the departments with the highest and lowest burden. Higher age-standardized DALY rates were concentrated in departments of the Andean region. Risaralda presented the highest cumulative age-standardized rate (918.6 per 100,000; 95% CI 831.0–1006.1), followed by Bogotá, D.C. (864.4; 95% CI 827.2–901.7), Santander (860.3; 95% CI 800.3–920.2), and Caldas (831.0; 95% CI 754.8–907.3). Valle del Cauca (747.9; 95% CI 710.9–784.8) and Antioquia (728.1; 95% CI 694.3–761.8) also showed elevated burden levels. By contrast, the lowest cumulative age-standardized rates were observed in Vaupés (20.8 per 100,000; 95% CI 16.7–24.9), Guainía (27.2; 95% CI 23.3–31.1) and Amazonas (37.1; 95% CI 33.5–40.7). Estimates for sparsely populated departments showed greater statistical uncertainty, as reflected by wider confidence intervals for some annual and period age-standardized rates, particularly in the Archipelago of San Andrés, Guaviare, Vichada, and other departments with small population denominators. In a few instances, the lower confidence limits were negative because confidence intervals were estimated using a normal approximation under a Poisson assumption. These negative values represent a statistical artifact of the approximation rather than epidemiologically meaningful negative DALY rates.

**Fig. 3:**
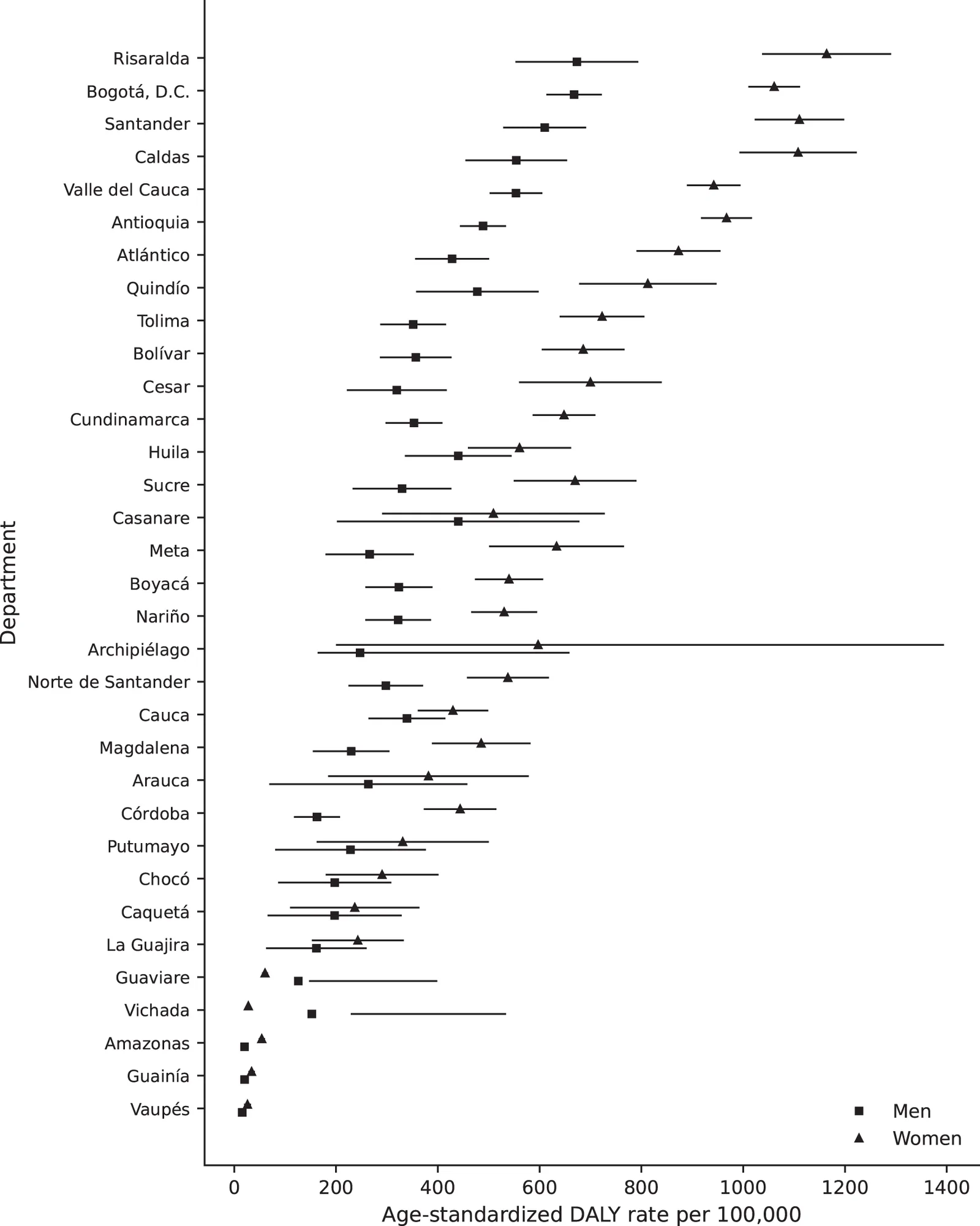
Department-level age-standardized DALY rates for anxiety disorders by sex in Colombia, 2016–2022. Department-level age-standardized DALY rates for anxiety disorders by sex in Colombia, 2016–2022. Squares represent men and triangles represent women. Horizontal bars indicate 95% confidence intervals. Rates are expressed per 100,000 population

The differential pattern of burden by sex observed at the national level was replicated across all departments (Fig. 3). In Antioquia, the cumulative age-standardized rate was 967.3 (95% CI 917.2–1017.4) in women compared with 488.8 (95% CI 443.6–534.0) in men. In Amazonas, rates were also higher in women, at 54.1 (95% CI 47.9–60.3), compared with 20.2 (95% CI 16.6–23.8) in men.

Temporal trend analysis showed statistically significant increases in all departments (Supplementary Table S2). However, the magnitude of change was heterogeneous. In lower-burden departments such as Amazonas, the APC was 21.11% (95% CI 8.66–34.98), with sex-specific values of 25.48% in men (95% CI 7.33–46.69) and 19.55% in women (95% CI 8.60–31.61). By contrast, in higher-burden departments such as Antioquia, the APC was more moderate, at 14.23% in men (95% CI 1.79–28.20), 13.37% in women (95% CI 1.72–26.34), and 13.60% overall (95% CI 2.00–26.53).

In terms of annual change, high-burden departments showed consistently upward trajectories. In Antioquia, for example, the rate in women increased from 509.6 (95% CI 400.2–619.0) in 2016 to 866.8 (95% CI 746.3–987.4) in 2022, with a peak of 1505.5 (95% CI 1343.7–1667.3) in 2021. In lower-burden departments such as Amazonas, rates remained low, although with relative increases, rising from 13.2 (95% CI 6.9–19.6) in 2016 to 61.1 (95% CI 49.5–72.8) in 2022.

**Fig. 4:**
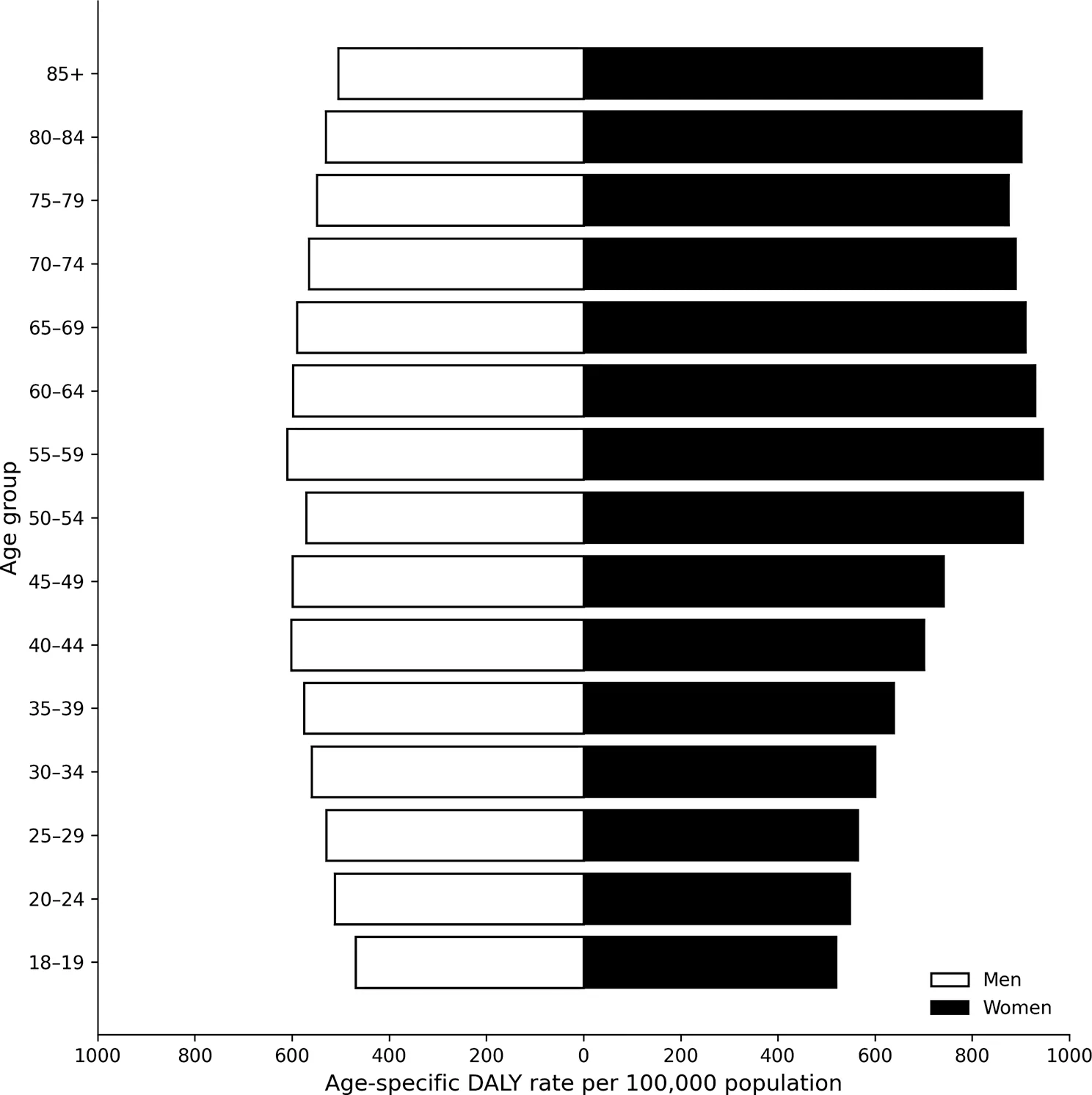
Age- and sex-specific distribution of anxiety disorder DALY rates in Colombia, 2016–2022. The study population was restricted to adults aged ≥ 18 years; therefore, the first age group includes only individuals aged 18–19 years, whereas subsequent age groups follow five-year intervals

Age-stratified analysis showed a heterogeneous distribution of the burden of disease across the life course (Fig. 4). Cumulative age-specific DALY rates showed a marked and progressive increase in the burden attributable to anxiety disorders across the life course, with substantial differences by sex. Overall, women consistently presented higher rates than men across all age groups, indicating a greater sustained burden throughout the study period. In both sexes, rates were relatively low during adolescence and early adulthood, followed by a gradual increase from 25 to 29 years up to 55–59 years.

Population pyramid showing cumulative age- and sex-specific DALY rates attributable to anxiety disorders in Colombia during 2016–2022. Bars represent cumulative rates per 100,000 population across five-year age groups. Male rates are shown on the left and female rates on the right. Higher rates were observed among women across most age groups, with the greatest burden concentrated in working-age adults.

Finally, regarding recorded mortality, an estimated 680.22 Years of Life Lost (YLLs) (95% CI 497.3–863.2) were calculated for Colombia. The mean age at death was 62.57 years (95% CI 51.74–73.40) in women and 57.71 years (95% CI 35.29–80.14) in men.

Overall, the burden of disease due to anxiety disorders in Colombia was characterized by a sustained increase over time, a high cumulative burden, marked territorial inequalities, and a differential distribution by sex and age. This pattern reflects a concentration of burden in specific regions of the country, a greater impact among women, and an age distribution centered in adulthood.

## Discussion

This study shows that anxiety disorders represented a significant and increasing burden of disease in Colombia during the period 2016–2022. The observed burden was explained predominantly by disability rather than mortality, with YLDs accounting for 99.95% of total DALYs, showed a sustained and statistically significant increase over time, and presented marked territorial inequalities, as well as persistent differences by sex and age.

Taken together, these findings suggest that anxiety disorders constitute a relevant priority for public health and for the organization of mental health services in the country. They also suggest that the observed increase does not respond solely to short-term events, but also reflects structural dynamics of greater complexity, consistent with what has been described in global and regional analyses, where these disorders rank among the leading causes of non-fatal disability worldwide [7, 16].

The sustained increase observed at both the national and departmental levels suggests a growing burden of anxiety disorders in Colombia. However, this increase should be interpreted with caution because administrative healthcare databases reflect both underlying epidemiological changes and changes in healthcare utilization and case detection. Therefore, the observed temporal trend should not be interpreted exclusively as an increase in disease occurrence [17].

The magnitude of the Annual Percent Change (APC) estimated in this study is consistent with global trends showing an increasing burden of anxiety disorders. However, the relatively high magnitude observed in Colombia may be influenced by the short length of the time series, interannual fluctuations, and recent contextual events that modified health care demand or the clinical expression of these disorders [3–5, 18, 19]. Therefore, although the direction of the trend appears robust, the specific magnitude of the APC should be interpreted with caution.

The marked increase in DALY rates observed during the study period is unlikely to be explained by a single factor. In addition to the potential effects of the COVID-19 pandemic, several changes within the Colombian health system may have contributed to the observed trend. Progressive improvements in mental health awareness, increased healthcare-seeking behavior, expanded access to mental health services, strengthening of diagnostic practices, improvements in administrative reporting, and greater coverage and completeness of the RIPS information system may have increased the number of clinically diagnosed cases recorded over time [3–7, 20–22]. Consequently, part of the observed increase in DALY rates may reflect improvements in case detection and health information systems in addition to genuine changes in the epidemiology of anxiety disorders. Because the present study was based on administrative healthcare data, these factors cannot be completely disentangled and should be considered when interpreting temporal trends.

Visual inspection of the annual DALY estimates suggests an apparent acceleration during 2019–2020; however, the present study was not designed to formally evaluate changes in temporal slopes, and this observation should therefore be interpreted cautiously, where a substantial increase in the prevalence, symptomatology, and burden of anxiety disorders has been reported [3–7, 22]. The pandemic generated social isolation, economic uncertainty, bereavement, disruption of routines, and barriers to access to services, factors potentially associated with deterioration in psychological well-being. However, given the ecological design of the study, it is not possible to attribute direct causality to this event; therefore, this relationship should be interpreted as a temporal association consistent with the available evidence.

The results also showed a territorial temporal dynamic characterized by an increase in burden across all departments, with greater relative growth in territories that had previously reported lower burden. This pattern could suggest a process of territorial expansion of the observed burden. Similar findings have been described in Global Burden of Disease (GBD) studies, which have noted a progressive diffusion of burden toward regions previously less affected [17]. However, it is also plausible that this pattern reflects differential improvements in access to services, greater diagnostic availability, and strengthened administrative reporting in historically lagging territories [20, 21]. In turn, the more moderate growth observed in high-burden departments could be related to relative stabilization or saturation effects in contexts where the disease already presents elevated levels.

The marked geographical inequalities observed in this study are consistent with international evidence indicating that the distribution of anxiety disorders is influenced not only by differences in disease occurrence but also by socioeconomic conditions, urbanization, access to health services, and the organization of mental healthcare systems [6, 7, 22]. The concentration of higher DALY rates in departments of the Andean region, particularly Risaralda, Caldas, Bogotá, D.C., Santander, Antioquia, and Valle del Cauca, may reflect the combined influence of greater urbanization, higher population density, increased psychosocial demands, and greater availability and accessibility of specialized mental health services, which facilitate case detection and diagnosis. These regions have also experienced persistent social inequalities and different forms of violence that may contribute to a greater burden of anxiety disorders [23, 24]. However, because the present study is ecological, these factors should be interpreted as plausible contextual explanations rather than causal determinants.

Conversely, the substantially lower burden observed in the Amazon and Orinoquía regions should be interpreted with caution. Rather than indicating a truly lower occurrence of anxiety disorders, these findings may reflect geographic barriers to healthcare, reduced availability of specialized mental health services, lower diagnostic capacity, underdiagnosis, and differences in the completeness of administrative health records [20, 25, 26]. Consequently, the observed territorial inequalities are likely to reflect both differences in population health needs and inequalities in access to mental healthcare and case detection.

The higher burden observed in women throughout the study period is consistent with abundant international evidence showing a greater prevalence and burden of anxiety disorders in this population group [6, 22, 27]. These differences have been attributed to a complex combination of biological, hormonal, and psychosocial factors, including greater cumulative exposure to stressors, gender-based violence, double work burdens, caregiving overload, and persistent structural inequalities [22, 27–29].

The finding of relatively greater growth in men suggests possible recent changes in the epidemiological dynamics. Plausible explanations include improvements in diagnostic recognition, changes in health care-seeking patterns, progressive reduction of stigma associated with mental health in this group, and greater institutional sensitivity to detect previously overlooked cases [28, 30]. However, this interpretation should be considered with caution due to the limitations inherent to the data used.

The consistency of the sex-specific pattern across all departments suggests that the observed differences have a structural component and do not depend exclusively on geographic context. However, variation in the magnitude of the gap between territories indicates that social and contextual determinants modulate this relationship [1, 8, 24]. Recent evidence from Latin America has indicated that the interaction between gender and socioeconomic context constitutes a key determinant in the distribution of mental disorders [4, 19, 27, 29].

The gradual increase in burden from 25 to 29 years up to 54–59 years has particularly important implications. These ages correspond to stages of high economic productivity, family responsibilities, and active labor force participation. Therefore, the impact of anxiety disorders extends beyond the clinical sphere and includes work absenteeism, presenteeism, reduced income, impairment of family caregiving, and substantial indirect costs for households and social systems [4, 27, 31]. These results are similar to those reported in other international and regional studies, which place the greatest burden in early and middle adulthood [22, 27].

Finally, it is a priority to strengthen mental health information systems, improve diagnostic quality, and advance toward more sensitive and timely surveillance systems to monitor trends, identify inequalities, and evaluate implemented policies [1]. In summary, the results show that anxiety disorders constitute a growing and unequally distributed burden in Colombia, which requires comprehensive, territorially targeted, and evidence-informed responses. These findings are particularly relevant for middle-income countries with fragmented mental health systems and pronounced territorial inequalities.

## Conclusions

Anxiety disorders accounted for a substantial estimated burden among individuals recorded in the Colombian health system during 2016–2022, predominantly driven by disability rather than premature mortality. Age-standardized DALY rates based on recorded morbidity increased substantially over the study period and showed marked territorial heterogeneity, together with persistent differences by sex and age.

Higher burden estimates were concentrated in departments of the Andean region, whereas lower recorded rates were observed in Amazonian and Orinoquía territories, revealing important subnational heterogeneity. Women consistently showed higher estimated burden than men across all departments and age groups.

These findings provide standardized subnational evidence for monitoring anxiety-related burden in Colombia and contribute to the understanding of territorial inequalities in mental health within middle-income settings. They may also inform territorial prioritization, mental health planning, and epidemiological surveillance. The study further demonstrates the usefulness of nationwide administrative health databases and WHO-compatible burden-of-disease methodology for population-level burden assessment.

## Limitations

This study has several limitations that should be considered when interpreting the findings. Morbidity estimates were derived from administrative records of health service utilization rather than from population-based epidemiological surveys. Consequently, the estimated Years Lived with Disability (YLDs) reflect the burden among individuals who accessed healthcare services and were recorded in the Colombian health system, rather than the total burden of anxiety disorders in the general population. The use of administrative records may be affected by underreporting, coding errors, variability in diagnostic practices, differences in access to healthcare, and regional variations in reporting quality. Consequently, part of the observed territorial variation may reflect differences in healthcare utilization and recording practices rather than true differences in the burden of anxiety disorders. Accordingly, temporal increases in DALY rates should be interpreted as increases in the estimated burden derived from recorded and treated morbidity and cannot be assumed to represent equivalent increases in the underlying population prevalence or incidence of anxiety disorders.

Mortality attributed to anxiety disorders was infrequent during the study period; therefore, burden estimates were explained predominantly by disability (YLDs). This pattern is consistent with the largely non-fatal nature of these disorders, although it limits the interpretation of the mortality component

DALYs were estimated using standard burden-of-disease methodology based on disability weights and standard life expectancy tables. As with all burden-of-disease studies, these assumptions may influence the absolute magnitude of the estimated burden. In addition, uncertainty around crude and age-standardized DALY rates was estimated using variance formulas based on a Poisson approximation. Although this approach is widely used in burden-of-disease analyses, estimates for sparsely populated departments were associated with greater statistical uncertainty because of the limited number of observed events and smaller population denominators. Consequently, confidence intervals were wider in these territories, and the normal approximation occasionally produced negative lower confidence limits, which should be interpreted as a statistical artifact rather than epidemiologically meaningful negative DALY rates. Furthermore, the estimation of years of life lost did not explicitly account for competing risks of mortality, which may result in a slight overestimation of YLLs, particularly in older age groups.

Annual Percent Change (APC) estimates were based on a relatively short time series (2016–2022). Furthermore, temporal trends were evaluated using a single log-linear regression model that estimated an overall APC across the study period and therefore did not formally assess changes in trend over time. Consequently, the observed variations may have been influenced by interannual fluctuations or short-term events, including the COVID-19 pandemic.

Finally, due to the ecological design of the study, the results should be interpreted at the population level and do not allow causal associations or inferences at the individual level.

Despite these limitations, the study provides relevant subnational evidence for service planning, territorial prioritization, and the strengthening of public mental health policies in Colombia.

## Electronic supplementary material

Below is the link to the electronic supplementary material.

Supplementary Material 1

Supplementary Material 2

Supplementary Material 3

Supplementary Material 4

## Data Availability

The datasets analyzed during the current study were obtained from official Colombian national administrative health information systems, including the Integrated Social Protection Information System (SISPRO), the Single Registry of Affiliates (RUAF), and population projections from the National Administrative Department of Statistics (DANE). SISPRO databases are publicly accessible through the Colombian Ministry of Health and Social Protection. However, access to individual databases requires institutional authorization and user credentials issued by the Ministry of Health and Social Protection of Colombia. Researchers interested in accessing these data should request authorization directly from the Ministry through the official SISPRO platform. RUAF mortality data and DANE population projections are publicly available from the corresponding governmental institutions. No new datasets were generated during the current study.
