# Supplementary material for: Burden of anxiety disorders in Colombia: subnational DALY estimates and territorial inequalities, 2016–2022
Source: Popul Health Metr. 2026 Sep 15;24:55. doi: 10.1186/s12963-026-00510-4 (PMC13591821; doi:10.1186/s12963-026-00510-4)
Supplement: Supplementary file 1 [file 12963_2026_510_MOESM1_ESM.docx]

**Supplementary Table S1**

Supplementary Table S1. Annual frequency of recorded morbidity and mortality associated with anxiety disorders in Colombia by department, sex, ICD-10 code, and year

Annual counts of recorded morbidity and mortality associated with anxiety disorders, stratified by department, sex, ICD-10 code, and calendar year during 2016–2022.

|  |  | Men | | | | | | | | | | | | | | Women | | | | | | | | | | | | | | Total | | | | | | | | | | | | | |
| --- | --- | --- | --- | --- | --- | --- | --- | --- | --- | --- | --- | --- | --- | --- | --- | --- | --- | --- | --- | --- | --- | --- | --- | --- | --- | --- | --- | --- | --- | --- | --- | --- | --- | --- | --- | --- | --- | --- | --- | --- | --- | --- | --- |
|  |  | Morbidity | | | | | | | Mortality | | | | | | | Morbidity | | | | | | | Mortality | | | | | | | Morbidity | | | | | | | Mortality | | | | | | |
| Department | ICD-10 | 2016 | 2017 | 2018 | 2019 | 2020 | 2021 | 2022 | 2016 | 2017 | 2018 | 2019 | 2020 | 2021 | 2022 | 2016 | 2017 | 2018 | 2019 | 2020 | 2021 | 2022 | 2016 | 2017 | 2018 | 2019 | 2020 | 2021 | 2022 | 2016 | 2017 | 2018 | 2019 | 2020 | 2021 | 2022 | 2016 | 2017 | 2018 | 2019 | 2020 | 2021 | 2022 |
| Amazonas | F402 | 0 | 0 | 0 | 0 | 0 | 0 | 0 | 0 | 0 | 0 | 0 | 0 | 0 | 0 | 1 | 1 | 0 | 0 | 0 | 1 | 0 | 0 | 0 | 0 | 0 | 0 | 0 | 0 | 1 | 1 | 0 | 0 | 0 | 1 | 0 | 0 | 0 | 0 | 0 | 0 | 0 | 0 |
|  | F408 | 1 | 0 | 2 | 0 | 0 | 1 | 0 | 0 | 0 | 0 | 0 | 0 | 0 | 0 | 1 | 2 | 0 | 1 | 4 | 0 | 0 | 0 | 0 | 0 | 0 | 0 | 0 | 0 | 2 | 2 | 2 | 1 | 4 | 1 | 0 | 0 | 0 | 0 | 0 | 0 | 0 | 0 |
|  | F409 | 0 | 0 | 0 | 1 | 0 | 1 | 0 | 0 | 0 | 0 | 0 | 0 | 0 | 0 | 1 | 1 | 3 | 1 | 0 | 1 | 2 | 0 | 0 | 0 | 0 | 0 | 0 | 0 | 1 | 1 | 3 | 2 | 0 | 2 | 2 | 0 | 0 | 0 | 0 | 0 | 0 | 0 |
|  | F410 | 0 | 1 | 0 | 3 | 1 | 0 | 3 | 0 | 0 | 0 | 0 | 0 | 0 | 0 | 1 | 0 | 3 | 7 | 1 | 2 | 4 | 0 | 0 | 0 | 0 | 0 | 0 | 0 | 1 | 1 | 3 | 10 | 2 | 2 | 7 | 0 | 0 | 0 | 0 | 0 | 0 | 0 |
|  | F411 | 0 | 0 | 0 | 7 | 3 | 2 | 10 | 0 | 0 | 0 | 0 | 0 | 0 | 0 | 4 | 7 | 16 | 20 | 7 | 4 | 25 | 0 | 0 | 0 | 0 | 0 | 0 | 0 | 4 | 7 | 16 | 27 | 10 | 6 | 35 | 0 | 0 | 0 | 0 | 0 | 0 | 0 |
|  | F412 | 2 | 9 | 10 | 12 | 12 | 10 | 17 | 0 | 0 | 0 | 0 | 0 | 0 | 0 | 12 | 36 | 30 | 38 | 44 | 28 | 66 | 0 | 0 | 0 | 0 | 0 | 0 | 0 | 14 | 45 | 40 | 50 | 56 | 38 | 83 | 0 | 0 | 0 | 0 | 0 | 0 | 0 |
|  | F413 | 0 | 1 | 1 | 1 | 1 | 0 | 4 | 0 | 0 | 0 | 0 | 0 | 0 | 0 | 1 | 4 | 3 | 2 | 1 | 1 | 3 | 0 | 0 | 0 | 0 | 0 | 0 | 0 | 1 | 5 | 4 | 3 | 2 | 1 | 7 | 0 | 0 | 0 | 0 | 0 | 0 | 0 |
|  | F418 | 2 | 2 | 1 | 5 | 8 | 2 | 8 | 0 | 0 | 0 | 0 | 0 | 0 | 0 | 3 | 2 | 9 | 5 | 4 | 6 | 10 | 0 | 0 | 0 | 0 | 0 | 0 | 0 | 5 | 4 | 10 | 10 | 12 | 8 | 18 | 0 | 0 | 0 | 0 | 0 | 0 | 0 |
|  | F419 | 5 | 6 | 12 | 26 | 26 | 12 | 27 | 0 | 0 | 0 | 0 | 0 | 0 | 0 | 8 | 20 | 16 | 41 | 42 | 45 | 58 | 0 | 0 | 0 | 0 | 0 | 0 | 0 | 13 | 26 | 28 | 67 | 68 | 57 | 85 | 0 | 0 | 0 | 0 | 0 | 0 | 0 |
|  | F420 | 0 | 0 | 0 | 0 | 0 | 0 | 1 | 0 | 0 | 0 | 0 | 0 | 0 | 0 | 0 | 0 | 0 | 1 | 0 | 0 | 1 | 0 | 0 | 0 | 0 | 0 | 0 | 0 | 0 | 0 | 0 | 1 | 0 | 0 | 2 | 0 | 0 | 0 | 0 | 0 | 0 | 0 |
|  | F422 | 0 | 0 | 0 | 0 | 0 | 0 | 0 | 0 | 0 | 0 | 0 | 0 | 0 | 0 | 0 | 0 | 0 | 1 | 0 | 0 | 0 | 0 | 0 | 0 | 0 | 0 | 0 | 0 | 0 | 0 | 0 | 1 | 0 | 0 | 0 | 0 | 0 | 0 | 0 | 0 | 0 | 0 |
|  | F428 | 0 | 0 | 0 | 0 | 0 | 0 | 1 | 0 | 0 | 0 | 0 | 0 | 0 | 0 | 0 | 0 | 0 | 0 | 0 | 0 | 0 | 0 | 0 | 0 | 0 | 0 | 0 | 0 | 0 | 0 | 0 | 0 | 0 | 0 | 1 | 0 | 0 | 0 | 0 | 0 | 0 | 0 |
|  | F429 | 0 | 0 | 0 | 0 | 0 | 0 | 0 | 0 | 0 | 0 | 0 | 0 | 0 | 0 | 0 | 0 | 0 | 0 | 0 | 1 | 1 | 0 | 0 | 0 | 0 | 0 | 0 | 0 | 0 | 0 | 0 | 0 | 0 | 1 | 1 | 0 | 0 | 0 | 0 | 0 | 0 | 0 |
|  | F430 | 0 | 0 | 0 | 0 | 0 | 0 | 1 | 0 | 0 | 0 | 0 | 0 | 0 | 0 | 0 | 0 | 0 | 1 | 2 | 4 | 2 | 0 | 0 | 0 | 0 | 0 | 0 | 0 | 0 | 0 | 0 | 1 | 2 | 4 | 3 | 0 | 0 | 0 | 0 | 0 | 0 | 0 |
|  | F431 | 0 | 0 | 0 | 4 | 0 | 1 | 5 | 0 | 0 | 0 | 0 | 0 | 0 | 0 | 1 | 0 | 4 | 3 | 2 | 2 | 3 | 0 | 0 | 0 | 0 | 0 | 0 | 0 | 1 | 0 | 4 | 7 | 2 | 3 | 8 | 0 | 0 | 0 | 0 | 0 | 0 | 0 |
|  | F432 | 0 | 1 | 1 | 4 | 1 | 1 | 9 | 0 | 0 | 0 | 0 | 0 | 0 | 0 | 2 | 5 | 4 | 4 | 2 | 7 | 10 | 0 | 0 | 0 | 0 | 0 | 0 | 0 | 2 | 6 | 5 | 8 | 3 | 8 | 19 | 0 | 0 | 0 | 0 | 0 | 0 | 0 |
|  | F438 | 0 | 0 | 0 | 0 | 0 | 0 | 0 | 0 | 0 | 0 | 0 | 0 | 0 | 0 | 0 | 0 | 0 | 0 | 1 | 0 | 0 | 0 | 0 | 0 | 0 | 0 | 0 | 0 | 0 | 0 | 0 | 0 | 1 | 0 | 0 | 0 | 0 | 0 | 0 | 0 | 0 | 0 |
|  | F439 | 0 | 0 | 0 | 0 | 6 | 2 | 0 | 0 | 0 | 0 | 0 | 0 | 0 | 0 | 0 | 0 | 0 | 0 | 6 | 6 | 0 | 0 | 0 | 0 | 0 | 0 | 0 | 0 | 0 | 0 | 0 | 0 | 12 | 8 | 0 | 0 | 0 | 0 | 0 | 0 | 0 | 0 |
| Antioquia | F400 | 12 | 21 | 19 | 32 | 18 | 21 | 16 | 0 | 0 | 0 | 0 | 0 | 0 | 0 | 20 | 21 | 25 | 42 | 32 | 40 | 24 | 0 | 0 | 0 | 0 | 0 | 0 | 0 | 32 | 42 | 44 | 74 | 50 | 61 | 40 | 0 | 0 | 0 | 0 | 0 | 0 | 0 |
|  | F401 | 38 | 57 | 60 | 83 | 48 | 74 | 78 | 0 | 0 | 0 | 0 | 0 | 0 | 0 | 29 | 48 | 39 | 51 | 51 | 84 | 63 | 0 | 0 | 0 | 0 | 0 | 0 | 0 | 67 | 105 | 99 | 134 | 99 | 158 | 141 | 0 | 0 | 0 | 0 | 0 | 0 | 0 |
|  | F402 | 25 | 26 | 33 | 42 | 25 | 48 | 28 | 0 | 0 | 0 | 0 | 0 | 0 | 0 | 34 | 57 | 58 | 85 | 68 | 85 | 54 | 0 | 0 | 0 | 0 | 0 | 0 | 0 | 59 | 83 | 91 | 127 | 93 | 133 | 82 | 0 | 0 | 0 | 0 | 0 | 0 | 0 |
|  | F408 | 58 | 59 | 88 | 102 | 107 | 107 | 98 | 0 | 0 | 0 | 0 | 0 | 0 | 0 | 79 | 112 | 166 | 208 | 209 | 205 | 192 | 0 | 0 | 0 | 0 | 0 | 0 | 0 | 137 | 171 | 254 | 310 | 316 | 312 | 290 | 0 | 0 | 0 | 0 | 0 | 0 | 0 |
|  | F409 | 90 | 150 | 108 | 133 | 148 | 166 | 84 | 0 | 0 | 0 | 0 | 0 | 0 | 0 | 207 | 308 | 268 | 357 | 324 | 362 | 265 | 0 | 0 | 0 | 0 | 0 | 0 | 0 | 297 | 458 | 376 | 490 | 472 | 528 | 349 | 0 | 0 | 0 | 0 | 0 | 0 | 0 |
|  | F410 | 731 | 923 | 1083 | 1446 | 1230 | 1312 | 824 | 0 | 0 | 0 | 0 | 0 | 0 | 0 | 1547 | 1894 | 2370 | 3243 | 2435 | 2867 | 1924 | 0 | 0 | 0 | 0 | 0 | 0 | 0 | 2278 | 2817 | 3453 | 4689 | 3665 | 4179 | 2748 | 0 | 0 | 0 | 0 | 0 | 0 | 0 |
|  | F411 | 1425 | 2108 | 2970 | 6201 | 5518 | 6739 | 4240 | 0 | 0 | 0 | 0 | 0 | 0 | 0 | 3894 | 5537 | 7470 | 15041 | 12635 | 15879 | 10126 | 0 | 0 | 0 | 0 | 0 | 0 | 0 | 5319 | 7645 | 10440 | 21242 | 18153 | 22618 | 14366 | 0 | 0 | 0 | 0 | 0 | 0 | 0 |
|  | F412 | 3565 | 4948 | 6793 | 10372 | 7870 | 10865 | 7231 | 0 | 0 | 0 | 0 | 0 | 0 | 0 | 12873 | 17202 | 22067 | 31967 | 23338 | 32199 | 22946 | 0 | 0 | 0 | 1 | 0 | 0 | 0 | 16438 | 22150 | 28860 | 42339 | 31208 | 43064 | 30177 | 0 | 0 | 0 | 1 | 0 | 0 | 0 |
|  | F413 | 226 | 408 | 583 | 904 | 701 | 838 | 556 | 0 | 0 | 0 | 0 | 0 | 0 | 0 | 601 | 1050 | 1543 | 2144 | 1602 | 2107 | 1278 | 1 | 0 | 0 | 0 | 0 | 0 | 0 | 827 | 1458 | 2126 | 3048 | 2303 | 2945 | 1834 | 1 | 0 | 0 | 0 | 0 | 0 | 0 |
|  | F418 | 526 | 591 | 660 | 1190 | 1385 | 1677 | 1078 | 0 | 0 | 0 | 0 | 0 | 0 | 0 | 1384 | 1571 | 1552 | 2670 | 2986 | 3647 | 2372 | 0 | 0 | 0 | 0 | 0 | 0 | 0 | 1910 | 2162 | 2212 | 3860 | 4371 | 5324 | 3450 | 0 | 0 | 0 | 0 | 0 | 0 | 0 |
|  | F419 | 2990 | 3735 | 4145 | 6301 | 5385 | 6812 | 4328 | 0 | 0 | 0 | 0 | 0 | 0 | 0 | 7229 | 9108 | 9759 | 14638 | 12062 | 15514 | 10405 | 0 | 0 | 0 | 0 | 0 | 0 | 0 | 10219 | 12843 | 13904 | 20939 | 17447 | 22326 | 14733 | 0 | 0 | 0 | 0 | 0 | 0 | 0 |
|  | F420 | 115 | 97 | 152 | 147 | 97 | 140 | 123 | 0 | 0 | 0 | 0 | 0 | 0 | 0 | 133 | 112 | 159 | 132 | 89 | 169 | 138 | 0 | 0 | 0 | 0 | 0 | 0 | 0 | 248 | 209 | 311 | 279 | 186 | 309 | 261 | 0 | 0 | 0 | 0 | 0 | 0 | 0 |
|  | F421 | 23 | 27 | 32 | 42 | 32 | 29 | 42 | 0 | 0 | 0 | 0 | 0 | 0 | 0 | 32 | 34 | 42 | 48 | 46 | 35 | 38 | 0 | 0 | 0 | 0 | 0 | 0 | 0 | 55 | 61 | 74 | 90 | 78 | 64 | 80 | 0 | 0 | 0 | 0 | 0 | 0 | 0 |
|  | F422 | 49 | 61 | 38 | 53 | 32 | 42 | 34 | 0 | 0 | 0 | 0 | 0 | 0 | 0 | 81 | 83 | 89 | 80 | 36 | 68 | 32 | 0 | 0 | 0 | 0 | 0 | 0 | 0 | 130 | 144 | 127 | 133 | 68 | 110 | 66 | 0 | 0 | 0 | 0 | 0 | 0 | 0 |
|  | F428 | 82 | 94 | 168 | 206 | 130 | 150 | 127 | 0 | 0 | 0 | 0 | 0 | 0 | 0 | 85 | 88 | 143 | 173 | 127 | 160 | 164 | 0 | 0 | 0 | 0 | 0 | 0 | 0 | 167 | 182 | 311 | 379 | 257 | 310 | 291 | 0 | 0 | 0 | 0 | 0 | 0 | 0 |
|  | F429 | 208 | 252 | 335 | 437 | 315 | 388 | 277 | 0 | 0 | 0 | 0 | 0 | 0 | 0 | 190 | 211 | 300 | 350 | 262 | 351 | 258 | 0 | 0 | 0 | 0 | 0 | 0 | 0 | 398 | 463 | 635 | 787 | 577 | 739 | 535 | 0 | 0 | 0 | 0 | 0 | 0 | 0 |
|  | F430 | 75 | 106 | 252 | 244 | 254 | 290 | 185 | 0 | 0 | 0 | 0 | 0 | 0 | 0 | 211 | 286 | 366 | 512 | 654 | 699 | 448 | 0 | 0 | 0 | 0 | 0 | 0 | 0 | 286 | 392 | 618 | 756 | 908 | 989 | 633 | 0 | 0 | 0 | 0 | 0 | 0 | 0 |
|  | F431 | 151 | 162 | 406 | 568 | 401 | 461 | 395 | 0 | 0 | 0 | 0 | 0 | 0 | 0 | 242 | 274 | 373 | 536 | 377 | 472 | 467 | 0 | 0 | 0 | 0 | 0 | 0 | 0 | 393 | 436 | 779 | 1104 | 778 | 933 | 862 | 0 | 0 | 0 | 0 | 0 | 0 | 0 |
|  | F432 | 370 | 362 | 585 | 927 | 756 | 1019 | 1089 | 0 | 0 | 0 | 0 | 0 | 0 | 0 | 887 | 883 | 1186 | 1540 | 1316 | 1826 | 1789 | 0 | 0 | 0 | 0 | 0 | 0 | 0 | 1257 | 1245 | 1771 | 2467 | 2072 | 2845 | 2878 | 0 | 0 | 0 | 0 | 0 | 0 | 0 |
|  | F438 | 28 | 27 | 23 | 48 | 40 | 29 | 32 | 0 | 0 | 0 | 0 | 0 | 0 | 0 | 72 | 35 | 52 | 89 | 67 | 68 | 64 | 0 | 0 | 0 | 0 | 0 | 0 | 0 | 100 | 62 | 75 | 137 | 107 | 97 | 96 | 0 | 0 | 0 | 0 | 0 | 0 | 0 |
|  | F439 | 9 | 16 | 11 | 30 | 19 | 33 | 23 | 0 | 0 | 0 | 0 | 0 | 0 | 0 | 25 | 28 | 41 | 77 | 41 | 52 | 47 | 0 | 0 | 0 | 0 | 0 | 0 | 0 | 34 | 44 | 52 | 107 | 60 | 85 | 70 | 0 | 0 | 0 | 0 | 0 | 0 | 0 |
| Arauca | F400 | 0 | 0 | 0 | 0 | 0 | 2 | 0 | 0 | 0 | 0 | 0 | 0 | 0 | 0 | 0 | 0 | 0 | 1 | 0 | 0 | 0 | 0 | 0 | 0 | 0 | 0 | 0 | 0 | 0 | 0 | 0 | 1 | 0 | 2 | 0 | 0 | 0 | 0 | 0 | 0 | 0 | 0 |
|  | F401 | 0 | 0 | 0 | 0 | 1 | 2 | 0 | 0 | 0 | 0 | 0 | 0 | 0 | 0 | 0 | 0 | 0 | 1 | 1 | 4 | 1 | 0 | 0 | 0 | 0 | 0 | 0 | 0 | 0 | 0 | 0 | 1 | 2 | 6 | 1 | 0 | 0 | 0 | 0 | 0 | 0 | 0 |
|  | F402 | 0 | 1 | 0 | 2 | 1 | 0 | 3 | 0 | 0 | 0 | 0 | 0 | 0 | 0 | 1 | 1 | 1 | 3 | 2 | 1 | 4 | 0 | 0 | 0 | 0 | 0 | 0 | 0 | 1 | 2 | 1 | 5 | 3 | 1 | 7 | 0 | 0 | 0 | 0 | 0 | 0 | 0 |
|  | F408 | 1 | 1 | 5 | 6 | 3 | 0 | 3 | 0 | 0 | 0 | 0 | 0 | 0 | 0 | 0 | 3 | 3 | 2 | 4 | 4 | 3 | 0 | 0 | 0 | 0 | 0 | 0 | 0 | 1 | 4 | 8 | 8 | 7 | 4 | 6 | 0 | 0 | 0 | 0 | 0 | 0 | 0 |
|  | F409 | 0 | 2 | 3 | 5 | 8 | 5 | 9 | 0 | 0 | 0 | 0 | 0 | 0 | 0 | 2 | 5 | 7 | 6 | 13 | 15 | 16 | 0 | 0 | 0 | 0 | 0 | 0 | 0 | 2 | 7 | 10 | 11 | 21 | 20 | 25 | 0 | 0 | 0 | 0 | 0 | 0 | 0 |
|  | F410 | 0 | 3 | 8 | 12 | 12 | 18 | 24 | 0 | 0 | 0 | 0 | 0 | 0 | 0 | 5 | 5 | 9 | 7 | 16 | 44 | 43 | 0 | 0 | 0 | 0 | 0 | 0 | 0 | 5 | 8 | 17 | 19 | 28 | 62 | 67 | 0 | 0 | 0 | 0 | 0 | 0 | 0 |
|  | F411 | 5 | 12 | 23 | 34 | 46 | 46 | 72 | 0 | 0 | 0 | 0 | 0 | 0 | 0 | 9 | 21 | 33 | 56 | 99 | 121 | 148 | 0 | 0 | 0 | 0 | 0 | 0 | 0 | 14 | 33 | 56 | 90 | 145 | 167 | 220 | 0 | 0 | 0 | 0 | 0 | 0 | 0 |
|  | F412 | 17 | 41 | 73 | 142 | 199 | 296 | 371 | 0 | 0 | 0 | 0 | 0 | 0 | 0 | 79 | 156 | 230 | 453 | 484 | 722 | 976 | 0 | 0 | 0 | 0 | 0 | 0 | 0 | 96 | 197 | 303 | 595 | 683 | 1018 | 1347 | 0 | 0 | 0 | 0 | 0 | 0 | 0 |
|  | F413 | 0 | 5 | 3 | 9 | 8 | 34 | 62 | 0 | 0 | 0 | 0 | 0 | 0 | 0 | 3 | 6 | 11 | 9 | 17 | 53 | 81 | 0 | 0 | 0 | 0 | 0 | 0 | 0 | 3 | 11 | 14 | 18 | 25 | 87 | 143 | 0 | 0 | 0 | 0 | 0 | 0 | 0 |
|  | F418 | 2 | 7 | 9 | 13 | 33 | 42 | 54 | 0 | 0 | 0 | 0 | 0 | 0 | 0 | 6 | 11 | 17 | 28 | 49 | 65 | 93 | 0 | 0 | 0 | 0 | 0 | 0 | 0 | 8 | 18 | 26 | 41 | 82 | 107 | 147 | 0 | 0 | 0 | 0 | 0 | 0 | 0 |
|  | F419 | 17 | 29 | 50 | 84 | 114 | 132 | 129 | 0 | 0 | 0 | 0 | 0 | 0 | 0 | 47 | 82 | 86 | 163 | 205 | 272 | 259 | 0 | 0 | 0 | 0 | 0 | 0 | 0 | 64 | 111 | 136 | 247 | 319 | 404 | 388 | 0 | 0 | 0 | 0 | 0 | 0 | 0 |
|  | F420 | 0 | 0 | 1 | 0 | 1 | 2 | 4 | 0 | 0 | 0 | 0 | 0 | 0 | 0 | 0 | 0 | 1 | 1 | 2 | 2 | 3 | 0 | 0 | 0 | 0 | 0 | 0 | 0 | 0 | 0 | 2 | 1 | 3 | 4 | 7 | 0 | 0 | 0 | 0 | 0 | 0 | 0 |
|  | F421 | 0 | 0 | 2 | 0 | 1 | 2 | 1 | 0 | 0 | 0 | 0 | 0 | 0 | 0 | 0 | 1 | 3 | 0 | 0 | 2 | 0 | 0 | 0 | 0 | 0 | 0 | 0 | 0 | 0 | 1 | 5 | 0 | 1 | 4 | 1 | 0 | 0 | 0 | 0 | 0 | 0 | 0 |
|  | F422 | 0 | 1 | 0 | 1 | 0 | 19 | 20 | 0 | 0 | 0 | 0 | 0 | 0 | 0 | 1 | 2 | 1 | 1 | 0 | 18 | 35 | 0 | 0 | 0 | 0 | 0 | 0 | 0 | 1 | 3 | 1 | 2 | 0 | 37 | 55 | 0 | 0 | 0 | 0 | 0 | 0 | 0 |
|  | F428 | 0 | 0 | 0 | 1 | 1 | 0 | 0 | 0 | 0 | 0 | 0 | 0 | 0 | 0 | 0 | 0 | 1 | 0 | 3 | 2 | 2 | 0 | 0 | 0 | 0 | 0 | 0 | 0 | 0 | 0 | 1 | 1 | 4 | 2 | 2 | 0 | 0 | 0 | 0 | 0 | 0 | 0 |
|  | F429 | 0 | 0 | 2 | 1 | 0 | 0 | 2 | 0 | 0 | 0 | 0 | 0 | 0 | 0 | 0 | 1 | 1 | 3 | 1 | 1 | 3 | 0 | 0 | 0 | 0 | 0 | 0 | 0 | 0 | 1 | 3 | 4 | 1 | 1 | 5 | 0 | 0 | 0 | 0 | 0 | 0 | 0 |
|  | F430 | 0 | 4 | 15 | 10 | 6 | 7 | 6 | 0 | 0 | 0 | 0 | 0 | 0 | 0 | 3 | 6 | 5 | 16 | 12 | 20 | 31 | 0 | 0 | 0 | 0 | 0 | 0 | 0 | 3 | 10 | 20 | 26 | 18 | 27 | 37 | 0 | 0 | 0 | 0 | 0 | 0 | 0 |
|  | F431 | 2 | 5 | 16 | 28 | 19 | 34 | 37 | 0 | 0 | 0 | 0 | 0 | 0 | 0 | 1 | 9 | 15 | 52 | 14 | 67 | 78 | 0 | 0 | 0 | 0 | 0 | 0 | 0 | 3 | 14 | 31 | 80 | 33 | 101 | 115 | 0 | 0 | 0 | 0 | 0 | 0 | 0 |
|  | F432 | 4 | 2 | 18 | 22 | 17 | 18 | 18 | 0 | 0 | 0 | 0 | 0 | 0 | 0 | 5 | 5 | 8 | 19 | 13 | 20 | 34 | 0 | 0 | 0 | 0 | 0 | 0 | 0 | 9 | 7 | 26 | 41 | 30 | 38 | 52 | 0 | 0 | 0 | 0 | 0 | 0 | 0 |
|  | F438 | 0 | 1 | 0 | 0 | 0 | 0 | 0 | 0 | 0 | 0 | 0 | 0 | 0 | 0 | 0 | 1 | 2 | 1 | 0 | 0 | 0 | 0 | 0 | 0 | 0 | 0 | 0 | 0 | 0 | 2 | 2 | 1 | 0 | 0 | 0 | 0 | 0 | 0 | 0 | 0 | 0 | 0 |
|  | F439 | 0 | 1 | 0 | 0 | 0 | 1 | 3 | 0 | 0 | 0 | 0 | 0 | 0 | 0 | 1 | 0 | 1 | 1 | 0 | 3 | 2 | 0 | 0 | 0 | 0 | 0 | 0 | 0 | 1 | 1 | 1 | 1 | 0 | 4 | 5 | 0 | 0 | 0 | 0 | 0 | 0 | 0 |
| Archipiélago | F400 | 0 | 0 | 0 | 0 | 0 | 0 | 0 | 0 | 0 | 0 | 0 | 0 | 0 | 0 | 0 | 0 | 0 | 0 | 0 | 0 | 1 | 0 | 0 | 0 | 0 | 0 | 0 | 0 | 0 | 0 | 0 | 0 | 0 | 0 | 1 | 0 | 0 | 0 | 0 | 0 | 0 | 0 |
|  | F401 | 0 | 0 | 0 | 0 | 0 | 0 | 1 | 0 | 0 | 0 | 0 | 0 | 0 | 0 | 0 | 0 | 0 | 0 | 0 | 1 | 0 | 0 | 0 | 0 | 0 | 0 | 0 | 0 | 0 | 0 | 0 | 0 | 0 | 1 | 1 | 0 | 0 | 0 | 0 | 0 | 0 | 0 |
|  | F402 | 0 | 0 | 0 | 0 | 0 | 0 | 1 | 0 | 0 | 0 | 0 | 0 | 0 | 0 | 0 | 0 | 0 | 0 | 0 | 1 | 1 | 0 | 0 | 0 | 0 | 0 | 0 | 0 | 0 | 0 | 0 | 0 | 0 | 1 | 2 | 0 | 0 | 0 | 0 | 0 | 0 | 0 |
|  | F408 | 0 | 2 | 0 | 1 | 2 | 0 | 1 | 0 | 0 | 0 | 0 | 0 | 0 | 0 | 1 | 0 | 0 | 0 | 1 | 5 | 1 | 0 | 0 | 0 | 0 | 0 | 0 | 0 | 1 | 2 | 0 | 1 | 3 | 5 | 2 | 0 | 0 | 0 | 0 | 0 | 0 | 0 |
|  | F409 | 0 | 1 | 0 | 0 | 0 | 1 | 5 | 0 | 0 | 0 | 0 | 0 | 0 | 0 | 0 | 4 | 0 | 1 | 2 | 5 | 5 | 0 | 0 | 0 | 0 | 0 | 0 | 0 | 0 | 5 | 0 | 1 | 2 | 6 | 10 | 0 | 0 | 0 | 0 | 0 | 0 | 0 |
|  | F410 | 4 | 4 | 0 | 2 | 5 | 8 | 8 | 0 | 0 | 0 | 0 | 0 | 0 | 0 | 6 | 7 | 2 | 6 | 10 | 12 | 9 | 0 | 0 | 0 | 0 | 0 | 0 | 0 | 10 | 11 | 2 | 8 | 15 | 20 | 17 | 0 | 0 | 0 | 0 | 0 | 0 | 0 |
|  | F411 | 1 | 6 | 2 | 3 | 22 | 30 | 52 | 0 | 0 | 0 | 0 | 0 | 0 | 0 | 3 | 3 | 2 | 7 | 41 | 87 | 129 | 0 | 0 | 0 | 0 | 0 | 0 | 0 | 4 | 9 | 4 | 10 | 63 | 117 | 181 | 0 | 0 | 0 | 0 | 0 | 0 | 0 |
|  | F412 | 2 | 18 | 8 | 11 | 32 | 38 | 51 | 0 | 0 | 0 | 0 | 0 | 0 | 0 | 18 | 43 | 8 | 44 | 67 | 92 | 169 | 0 | 0 | 0 | 0 | 0 | 0 | 0 | 20 | 61 | 16 | 55 | 99 | 130 | 220 | 0 | 0 | 0 | 0 | 0 | 0 | 0 |
|  | F413 | 0 | 2 | 0 | 2 | 4 | 12 | 8 | 0 | 0 | 0 | 0 | 0 | 0 | 0 | 1 | 4 | 0 | 3 | 12 | 25 | 39 | 0 | 0 | 0 | 0 | 0 | 0 | 0 | 1 | 6 | 0 | 5 | 16 | 37 | 47 | 0 | 0 | 0 | 0 | 0 | 0 | 0 |
|  | F418 | 2 | 2 | 0 | 3 | 7 | 5 | 2 | 0 | 0 | 0 | 0 | 0 | 0 | 0 | 1 | 4 | 2 | 5 | 20 | 6 | 32 | 0 | 0 | 0 | 0 | 0 | 0 | 0 | 3 | 6 | 2 | 8 | 27 | 11 | 34 | 0 | 0 | 0 | 0 | 0 | 0 | 0 |
|  | F419 | 5 | 11 | 2 | 10 | 40 | 30 | 48 | 0 | 0 | 0 | 0 | 0 | 0 | 0 | 4 | 30 | 4 | 36 | 67 | 82 | 102 | 0 | 0 | 0 | 0 | 0 | 0 | 0 | 9 | 41 | 6 | 46 | 107 | 112 | 150 | 0 | 0 | 0 | 0 | 0 | 0 | 0 |
|  | F420 | 0 | 1 | 0 | 0 | 0 | 2 | 0 | 0 | 0 | 0 | 0 | 0 | 0 | 0 | 0 | 0 | 0 | 0 | 1 | 0 | 2 | 0 | 0 | 0 | 0 | 0 | 0 | 0 | 0 | 1 | 0 | 0 | 1 | 2 | 2 | 0 | 0 | 0 | 0 | 0 | 0 | 0 |
|  | F422 | 0 | 0 | 0 | 0 | 0 | 0 | 1 | 0 | 0 | 0 | 0 | 0 | 0 | 0 | 0 | 0 | 0 | 0 | 0 | 2 | 0 | 0 | 0 | 0 | 0 | 0 | 0 | 0 | 0 | 0 | 0 | 0 | 0 | 2 | 1 | 0 | 0 | 0 | 0 | 0 | 0 | 0 |
|  | F428 | 0 | 0 | 0 | 0 | 0 | 0 | 1 | 0 | 0 | 0 | 0 | 0 | 0 | 0 | 0 | 0 | 0 | 0 | 0 | 0 | 0 | 0 | 0 | 0 | 0 | 0 | 0 | 0 | 0 | 0 | 0 | 0 | 0 | 0 | 1 | 0 | 0 | 0 | 0 | 0 | 0 | 0 |
|  | F429 | 0 | 0 | 0 | 0 | 0 | 0 | 0 | 0 | 0 | 0 | 0 | 0 | 0 | 0 | 0 | 0 | 0 | 0 | 1 | 1 | 3 | 0 | 0 | 0 | 0 | 0 | 0 | 0 | 0 | 0 | 0 | 0 | 1 | 1 | 3 | 0 | 0 | 0 | 0 | 0 | 0 | 0 |
|  | F430 | 0 | 0 | 0 | 1 | 5 | 5 | 2 | 0 | 0 | 0 | 0 | 0 | 0 | 0 | 0 | 1 | 0 | 1 | 5 | 6 | 9 | 0 | 0 | 0 | 0 | 0 | 0 | 0 | 0 | 1 | 0 | 2 | 10 | 11 | 11 | 0 | 0 | 0 | 0 | 0 | 0 | 0 |
|  | F431 | 1 | 2 | 1 | 5 | 9 | 4 | 5 | 0 | 0 | 0 | 0 | 0 | 0 | 0 | 2 | 3 | 3 | 2 | 13 | 10 | 15 | 0 | 0 | 0 | 0 | 0 | 0 | 0 | 3 | 5 | 4 | 7 | 22 | 14 | 20 | 0 | 0 | 0 | 0 | 0 | 0 | 0 |
|  | F432 | 0 | 0 | 0 | 1 | 3 | 3 | 10 | 0 | 0 | 0 | 0 | 0 | 0 | 0 | 3 | 0 | 0 | 1 | 1 | 5 | 26 | 0 | 0 | 0 | 0 | 0 | 0 | 0 | 3 | 0 | 0 | 2 | 4 | 8 | 36 | 0 | 0 | 0 | 0 | 0 | 0 | 0 |
|  | F438 | 0 | 1 | 0 | 0 | 0 | 0 | 0 | 0 | 0 | 0 | 0 | 0 | 0 | 0 | 0 | 0 | 1 | 0 | 1 | 0 | 1 | 0 | 0 | 0 | 0 | 0 | 0 | 0 | 0 | 1 | 1 | 0 | 1 | 0 | 1 | 0 | 0 | 0 | 0 | 0 | 0 | 0 |
|  | F439 | 0 | 0 | 0 | 1 | 0 | 0 | 0 | 0 | 0 | 0 | 0 | 0 | 0 | 0 | 1 | 0 | 0 | 0 | 1 | 1 | 1 | 0 | 0 | 0 | 0 | 0 | 0 | 0 | 1 | 0 | 0 | 1 | 1 | 1 | 1 | 0 | 0 | 0 | 0 | 0 | 0 | 0 |
| Atlántico | F400 | 3 | 3 | 13 | 18 | 11 | 6 | 3 | 0 | 0 | 0 | 0 | 0 | 0 | 0 | 4 | 4 | 11 | 32 | 18 | 15 | 8 | 0 | 0 | 0 | 0 | 0 | 0 | 0 | 7 | 7 | 24 | 50 | 29 | 21 | 11 | 0 | 0 | 0 | 0 | 0 | 0 | 0 |
|  | F401 | 5 | 16 | 4 | 15 | 15 | 16 | 19 | 0 | 0 | 0 | 0 | 0 | 0 | 0 | 7 | 7 | 5 | 34 | 18 | 13 | 17 | 0 | 0 | 0 | 0 | 0 | 0 | 0 | 12 | 23 | 9 | 49 | 33 | 29 | 36 | 0 | 0 | 0 | 0 | 0 | 0 | 0 |
|  | F402 | 4 | 4 | 9 | 20 | 9 | 7 | 10 | 0 | 0 | 0 | 0 | 0 | 0 | 0 | 6 | 8 | 14 | 47 | 20 | 24 | 23 | 0 | 0 | 0 | 0 | 0 | 0 | 0 | 10 | 12 | 23 | 67 | 29 | 31 | 33 | 0 | 0 | 0 | 0 | 0 | 0 | 0 |
|  | F408 | 18 | 11 | 23 | 50 | 43 | 32 | 32 | 0 | 0 | 0 | 0 | 0 | 0 | 0 | 31 | 35 | 45 | 82 | 107 | 98 | 73 | 0 | 0 | 0 | 0 | 0 | 0 | 0 | 49 | 46 | 68 | 132 | 150 | 130 | 105 | 0 | 0 | 0 | 0 | 0 | 0 | 0 |
|  | F409 | 47 | 26 | 32 | 69 | 126 | 109 | 50 | 0 | 0 | 0 | 0 | 0 | 0 | 0 | 62 | 49 | 56 | 118 | 195 | 177 | 197 | 0 | 0 | 0 | 0 | 0 | 0 | 0 | 109 | 75 | 88 | 187 | 321 | 286 | 247 | 0 | 0 | 0 | 0 | 0 | 0 | 0 |
|  | F410 | 72 | 91 | 118 | 217 | 262 | 333 | 241 | 0 | 0 | 0 | 0 | 0 | 0 | 0 | 143 | 149 | 168 | 392 | 451 | 600 | 460 | 0 | 0 | 0 | 0 | 0 | 0 | 0 | 215 | 240 | 286 | 609 | 713 | 933 | 701 | 0 | 0 | 0 | 0 | 0 | 0 | 0 |
|  | F411 | 163 | 259 | 399 | 762 | 1028 | 1389 | 1216 | 0 | 0 | 0 | 0 | 0 | 0 | 0 | 476 | 462 | 664 | 1277 | 1841 | 2748 | 2597 | 0 | 0 | 0 | 0 | 0 | 0 | 0 | 639 | 721 | 1063 | 2039 | 2869 | 4137 | 3813 | 0 | 0 | 0 | 0 | 0 | 0 | 0 |
|  | F412 | 1172 | 1330 | 2373 | 2990 | 2909 | 3605 | 5318 | 0 | 0 | 0 | 0 | 0 | 0 | 0 | 2815 | 3073 | 4434 | 6331 | 6632 | 9383 | 12242 | 0 | 0 | 0 | 0 | 0 | 0 | 0 | 3987 | 4403 | 6807 | 9321 | 9541 | 12988 | 17560 | 0 | 0 | 0 | 0 | 0 | 0 | 0 |
|  | F413 | 81 | 104 | 189 | 273 | 231 | 303 | 285 | 0 | 0 | 0 | 0 | 0 | 0 | 0 | 168 | 193 | 319 | 494 | 468 | 688 | 600 | 0 | 0 | 0 | 0 | 0 | 0 | 0 | 249 | 297 | 508 | 767 | 699 | 991 | 885 | 0 | 0 | 0 | 0 | 0 | 0 | 0 |
|  | F418 | 145 | 264 | 578 | 745 | 626 | 562 | 479 | 0 | 0 | 0 | 0 | 0 | 0 | 0 | 340 | 511 | 614 | 1046 | 882 | 999 | 900 | 0 | 0 | 0 | 0 | 0 | 0 | 0 | 485 | 775 | 1192 | 1791 | 1508 | 1561 | 1379 | 0 | 0 | 0 | 0 | 0 | 0 | 0 |
|  | F419 | 861 | 997 | 1308 | 1806 | 1667 | 2387 | 2383 | 0 | 0 | 0 | 0 | 0 | 0 | 0 | 1899 | 2027 | 2309 | 3667 | 3133 | 5082 | 5299 | 0 | 0 | 0 | 0 | 0 | 0 | 0 | 2760 | 3024 | 3617 | 5473 | 4800 | 7469 | 7682 | 0 | 0 | 0 | 0 | 0 | 0 | 0 |
|  | F420 | 12 | 16 | 19 | 33 | 21 | 26 | 13 | 0 | 0 | 0 | 0 | 0 | 0 | 0 | 14 | 17 | 16 | 51 | 31 | 22 | 17 | 0 | 0 | 0 | 0 | 0 | 0 | 0 | 26 | 33 | 35 | 84 | 52 | 48 | 30 | 0 | 0 | 0 | 0 | 0 | 0 | 0 |
|  | F421 | 5 | 8 | 7 | 18 | 12 | 5 | 11 | 0 | 0 | 0 | 0 | 0 | 0 | 0 | 3 | 6 | 8 | 20 | 22 | 12 | 7 | 0 | 0 | 0 | 0 | 0 | 0 | 0 | 8 | 14 | 15 | 38 | 34 | 17 | 18 | 0 | 0 | 0 | 0 | 0 | 0 | 0 |
|  | F422 | 5 | 4 | 4 | 19 | 10 | 4 | 3 | 0 | 0 | 0 | 0 | 0 | 0 | 0 | 6 | 2 | 0 | 24 | 13 | 7 | 10 | 0 | 0 | 0 | 0 | 0 | 0 | 0 | 11 | 6 | 4 | 43 | 23 | 11 | 13 | 0 | 0 | 0 | 0 | 0 | 0 | 0 |
|  | F428 | 14 | 12 | 24 | 29 | 27 | 40 | 26 | 0 | 0 | 0 | 0 | 0 | 0 | 0 | 6 | 15 | 17 | 40 | 42 | 27 | 33 | 0 | 0 | 0 | 0 | 0 | 0 | 0 | 20 | 27 | 41 | 69 | 69 | 67 | 59 | 0 | 0 | 0 | 0 | 0 | 0 | 0 |
|  | F429 | 27 | 32 | 42 | 72 | 71 | 84 | 74 | 0 | 0 | 0 | 0 | 0 | 0 | 0 | 16 | 23 | 37 | 60 | 57 | 66 | 65 | 0 | 0 | 0 | 0 | 0 | 0 | 0 | 43 | 55 | 79 | 132 | 128 | 150 | 139 | 0 | 0 | 0 | 0 | 0 | 0 | 0 |
|  | F430 | 46 | 53 | 61 | 121 | 101 | 87 | 81 | 0 | 0 | 0 | 0 | 0 | 0 | 0 | 142 | 161 | 157 | 246 | 242 | 238 | 176 | 0 | 0 | 0 | 0 | 0 | 0 | 0 | 188 | 214 | 218 | 367 | 343 | 325 | 257 | 0 | 0 | 0 | 0 | 0 | 0 | 0 |
|  | F431 | 25 | 39 | 256 | 308 | 186 | 178 | 228 | 0 | 0 | 0 | 0 | 0 | 0 | 0 | 43 | 34 | 69 | 114 | 95 | 133 | 164 | 0 | 0 | 0 | 0 | 0 | 0 | 0 | 68 | 73 | 325 | 422 | 281 | 311 | 392 | 0 | 0 | 0 | 0 | 0 | 0 | 0 |
|  | F432 | 25 | 21 | 54 | 215 | 198 | 294 | 302 | 0 | 0 | 0 | 0 | 0 | 0 | 0 | 34 | 41 | 60 | 237 | 233 | 401 | 498 | 0 | 0 | 0 | 0 | 0 | 0 | 0 | 59 | 62 | 114 | 452 | 431 | 695 | 800 | 0 | 0 | 0 | 0 | 0 | 0 | 0 |
|  | F438 | 6 | 16 | 21 | 35 | 20 | 8 | 16 | 0 | 0 | 0 | 0 | 0 | 0 | 0 | 32 | 19 | 27 | 40 | 41 | 51 | 44 | 0 | 0 | 0 | 0 | 0 | 0 | 0 | 38 | 35 | 48 | 75 | 61 | 59 | 60 | 0 | 0 | 0 | 0 | 0 | 0 | 0 |
|  | F439 | 7 | 6 | 20 | 50 | 65 | 12 | 12 | 0 | 0 | 0 | 0 | 0 | 0 | 0 | 19 | 12 | 39 | 116 | 202 | 37 | 20 | 0 | 0 | 0 | 0 | 0 | 0 | 0 | 26 | 18 | 59 | 166 | 267 | 49 | 32 | 0 | 0 | 0 | 0 | 0 | 0 | 0 |
| Bogotá, D.C. | F400 | 14 | 16 | 15 | 27 | 19 | 41 | 31 | 0 | 0 | 0 | 0 | 0 | 0 | 0 | 30 | 36 | 27 | 55 | 35 | 49 | 54 | 0 | 0 | 0 | 0 | 0 | 0 | 0 | 44 | 52 | 42 | 82 | 54 | 90 | 85 | 0 | 0 | 0 | 0 | 0 | 0 | 0 |
|  | F401 | 38 | 26 | 61 | 95 | 91 | 115 | 181 | 0 | 0 | 0 | 0 | 0 | 0 | 0 | 47 | 33 | 58 | 83 | 104 | 237 | 293 | 0 | 0 | 0 | 0 | 0 | 0 | 0 | 85 | 59 | 119 | 178 | 195 | 352 | 474 | 0 | 0 | 0 | 0 | 0 | 0 | 0 |
|  | F402 | 42 | 46 | 41 | 60 | 37 | 94 | 57 | 0 | 0 | 0 | 0 | 0 | 0 | 0 | 67 | 58 | 92 | 131 | 76 | 85 | 76 | 0 | 0 | 0 | 0 | 0 | 0 | 0 | 109 | 104 | 133 | 191 | 113 | 179 | 133 | 0 | 0 | 0 | 0 | 0 | 0 | 0 |
|  | F408 | 71 | 79 | 96 | 144 | 123 | 115 | 117 | 0 | 0 | 0 | 0 | 0 | 0 | 0 | 103 | 108 | 174 | 198 | 227 | 231 | 202 | 0 | 0 | 0 | 0 | 0 | 0 | 0 | 174 | 187 | 270 | 342 | 350 | 346 | 319 | 0 | 0 | 0 | 0 | 0 | 0 | 0 |
|  | F409 | 99 | 107 | 154 | 238 | 235 | 223 | 197 | 0 | 0 | 0 | 0 | 0 | 0 | 0 | 192 | 219 | 260 | 463 | 473 | 467 | 342 | 0 | 0 | 0 | 0 | 0 | 0 | 0 | 291 | 326 | 414 | 701 | 708 | 690 | 539 | 0 | 0 | 0 | 0 | 0 | 0 | 0 |
|  | F410 | 495 | 572 | 603 | 922 | 954 | 911 | 703 | 0 | 0 | 0 | 0 | 0 | 0 | 0 | 777 | 830 | 1016 | 1520 | 1609 | 1695 | 1486 | 0 | 0 | 0 | 0 | 0 | 0 | 0 | 1272 | 1402 | 1619 | 2442 | 2563 | 2606 | 2189 | 0 | 0 | 0 | 0 | 0 | 0 | 0 |
|  | F411 | 1357 | 1538 | 2045 | 3146 | 3973 | 4234 | 3401 | 0 | 0 | 0 | 0 | 0 | 0 | 0 | 2652 | 2739 | 3520 | 5781 | 7659 | 9132 | 7419 | 0 | 0 | 0 | 0 | 0 | 0 | 0 | 4009 | 4277 | 5565 | 8927 | 11632 | 13366 | 10820 | 0 | 0 | 0 | 0 | 0 | 0 | 0 |
|  | F412 | 4033 | 4330 | 5713 | 8823 | 9411 | 11442 | 10699 | 0 | 0 | 0 | 0 | 1 | 0 | 1 | 13084 | 13893 | 17230 | 26273 | 25845 | 31221 | 29701 | 0 | 0 | 0 | 0 | 0 | 0 | 0 | 17117 | 18223 | 22943 | 35096 | 35256 | 42663 | 40400 | 0 | 0 | 0 | 0 | 1 | 0 | 1 |
|  | F413 | 333 | 393 | 609 | 854 | 948 | 1132 | 867 | 0 | 0 | 0 | 0 | 0 | 0 | 0 | 749 | 916 | 1214 | 1717 | 1868 | 2376 | 1889 | 0 | 0 | 0 | 0 | 0 | 0 | 0 | 1082 | 1309 | 1823 | 2571 | 2816 | 3508 | 2756 | 0 | 0 | 0 | 0 | 0 | 0 | 0 |
|  | F418 | 879 | 1201 | 1421 | 2204 | 2717 | 3055 | 2428 | 0 | 0 | 0 | 0 | 0 | 0 | 0 | 1783 | 2342 | 2719 | 4261 | 4911 | 5888 | 4647 | 0 | 0 | 0 | 0 | 0 | 0 | 0 | 2662 | 3543 | 4140 | 6465 | 7628 | 8943 | 7075 | 0 | 0 | 0 | 0 | 0 | 0 | 0 |
|  | F419 | 4258 | 5001 | 6321 | 9143 | 10276 | 11981 | 10806 | 0 | 0 | 0 | 0 | 0 | 0 | 1 | 8620 | 9632 | 12483 | 17548 | 19593 | 23482 | 21340 | 0 | 0 | 0 | 0 | 0 | 0 | 0 | 12878 | 14633 | 18804 | 26691 | 29869 | 35463 | 32146 | 0 | 0 | 0 | 0 | 0 | 0 | 1 |
|  | F420 | 88 | 72 | 77 | 191 | 134 | 125 | 97 | 0 | 0 | 0 | 0 | 0 | 0 | 0 | 105 | 70 | 66 | 155 | 138 | 143 | 156 | 0 | 0 | 0 | 0 | 0 | 0 | 0 | 193 | 142 | 143 | 346 | 272 | 268 | 253 | 0 | 0 | 0 | 0 | 0 | 0 | 0 |
|  | F421 | 15 | 15 | 22 | 29 | 37 | 29 | 28 | 0 | 0 | 0 | 0 | 0 | 0 | 0 | 18 | 19 | 23 | 19 | 41 | 40 | 39 | 0 | 0 | 0 | 0 | 0 | 0 | 0 | 33 | 34 | 45 | 48 | 78 | 69 | 67 | 0 | 0 | 0 | 0 | 0 | 0 | 0 |
|  | F422 | 22 | 24 | 27 | 32 | 40 | 41 | 33 | 0 | 0 | 0 | 0 | 0 | 0 | 0 | 27 | 15 | 12 | 34 | 25 | 48 | 36 | 0 | 0 | 0 | 0 | 0 | 0 | 0 | 49 | 39 | 39 | 66 | 65 | 89 | 69 | 0 | 0 | 0 | 0 | 0 | 0 | 0 |
|  | F428 | 104 | 126 | 124 | 427 | 477 | 740 | 945 | 0 | 0 | 0 | 0 | 0 | 0 | 0 | 91 | 82 | 103 | 1182 | 1649 | 2450 | 2664 | 0 | 0 | 0 | 0 | 0 | 0 | 0 | 195 | 208 | 227 | 1609 | 2126 | 3190 | 3609 | 0 | 0 | 0 | 0 | 0 | 0 | 0 |
|  | F429 | 216 | 268 | 340 | 549 | 788 | 599 | 754 | 0 | 0 | 0 | 0 | 0 | 0 | 0 | 149 | 154 | 218 | 882 | 1136 | 943 | 1088 | 0 | 0 | 0 | 0 | 0 | 0 | 0 | 365 | 422 | 558 | 1431 | 1924 | 1542 | 1842 | 0 | 0 | 0 | 0 | 0 | 0 | 0 |
|  | F430 | 220 | 311 | 371 | 589 | 599 | 437 | 572 | 0 | 0 | 0 | 0 | 0 | 0 | 0 | 1632 | 1358 | 1019 | 2068 | 2814 | 2547 | 2162 | 0 | 0 | 0 | 0 | 0 | 1 | 0 | 1852 | 1669 | 1390 | 2657 | 3413 | 2984 | 2734 | 0 | 0 | 0 | 0 | 0 | 1 | 0 |
|  | F431 | 129 | 164 | 198 | 346 | 246 | 294 | 317 | 0 | 0 | 0 | 0 | 0 | 0 | 0 | 245 | 237 | 250 | 475 | 353 | 536 | 541 | 0 | 0 | 0 | 0 | 0 | 0 | 0 | 374 | 401 | 448 | 821 | 599 | 830 | 858 | 0 | 0 | 0 | 0 | 0 | 0 | 0 |
|  | F432 | 2170 | 2181 | 2960 | 4479 | 3966 | 4066 | 4749 | 0 | 0 | 0 | 0 | 0 | 0 | 0 | 5840 | 5787 | 7106 | 9855 | 9210 | 9218 | 10501 | 0 | 0 | 0 | 0 | 0 | 0 | 0 | 8010 | 7968 | 10066 | 14334 | 13176 | 13284 | 15250 | 0 | 0 | 0 | 0 | 0 | 0 | 0 |
|  | F438 | 34 | 51 | 43 | 72 | 102 | 109 | 108 | 0 | 0 | 0 | 0 | 0 | 0 | 0 | 112 | 171 | 101 | 198 | 307 | 240 | 301 | 0 | 0 | 0 | 0 | 0 | 0 | 0 | 146 | 222 | 144 | 270 | 409 | 349 | 409 | 0 | 0 | 0 | 0 | 0 | 0 | 0 |
|  | F439 | 28 | 42 | 45 | 59 | 33 | 36 | 44 | 0 | 0 | 0 | 0 | 0 | 0 | 0 | 59 | 93 | 72 | 73 | 72 | 80 | 73 | 0 | 0 | 0 | 0 | 0 | 0 | 0 | 87 | 135 | 117 | 132 | 105 | 116 | 117 | 0 | 0 | 0 | 0 | 0 | 0 | 0 |
| Bolívar | F400 | 1 | 1 | 3 | 12 | 7 | 7 | 6 | 0 | 0 | 0 | 0 | 0 | 0 | 0 | 1 | 4 | 5 | 13 | 14 | 15 | 10 | 0 | 0 | 0 | 0 | 0 | 0 | 0 | 2 | 5 | 8 | 25 | 21 | 22 | 16 | 0 | 0 | 0 | 0 | 0 | 0 | 0 |
|  | F401 | 1 | 4 | 3 | 12 | 2 | 10 | 41 | 0 | 0 | 0 | 0 | 0 | 0 | 0 | 1 | 3 | 4 | 9 | 12 | 10 | 98 | 0 | 0 | 0 | 0 | 0 | 0 | 0 | 2 | 7 | 7 | 21 | 14 | 20 | 139 | 0 | 0 | 0 | 0 | 0 | 0 | 0 |
|  | F402 | 10 | 7 | 6 | 13 | 5 | 11 | 17 | 0 | 0 | 0 | 0 | 0 | 0 | 0 | 11 | 5 | 15 | 35 | 12 | 18 | 16 | 0 | 0 | 0 | 0 | 0 | 0 | 0 | 21 | 12 | 21 | 48 | 17 | 29 | 33 | 0 | 0 | 0 | 0 | 0 | 0 | 0 |
|  | F408 | 6 | 6 | 10 | 26 | 31 | 27 | 28 | 0 | 0 | 0 | 0 | 0 | 0 | 0 | 15 | 22 | 29 | 52 | 50 | 64 | 68 | 0 | 0 | 0 | 0 | 0 | 0 | 0 | 21 | 28 | 39 | 78 | 81 | 91 | 96 | 0 | 0 | 0 | 0 | 0 | 0 | 0 |
|  | F409 | 11 | 20 | 23 | 39 | 41 | 44 | 57 | 0 | 0 | 0 | 0 | 0 | 0 | 0 | 44 | 44 | 35 | 71 | 81 | 112 | 117 | 0 | 0 | 0 | 0 | 0 | 0 | 0 | 55 | 64 | 58 | 110 | 122 | 156 | 174 | 0 | 0 | 0 | 0 | 0 | 0 | 0 |
|  | F410 | 38 | 46 | 123 | 93 | 110 | 129 | 123 | 0 | 0 | 0 | 0 | 0 | 0 | 0 | 87 | 91 | 167 | 224 | 191 | 247 | 284 | 0 | 0 | 0 | 0 | 0 | 0 | 0 | 125 | 137 | 290 | 317 | 301 | 376 | 407 | 0 | 0 | 0 | 0 | 0 | 0 | 0 |
|  | F411 | 129 | 225 | 307 | 661 | 991 | 1485 | 1515 | 0 | 0 | 0 | 0 | 0 | 0 | 0 | 308 | 491 | 702 | 1285 | 1640 | 2572 | 2990 | 0 | 0 | 0 | 0 | 0 | 0 | 0 | 437 | 716 | 1009 | 1946 | 2631 | 4057 | 4505 | 0 | 0 | 0 | 0 | 0 | 0 | 0 |
|  | F412 | 530 | 923 | 1239 | 1508 | 1445 | 2117 | 4176 | 0 | 0 | 0 | 1 | 0 | 0 | 0 | 1567 | 2359 | 3092 | 3688 | 3531 | 5590 | 9534 | 0 | 0 | 0 | 0 | 1 | 0 | 0 | 2097 | 3282 | 4331 | 5196 | 4976 | 7707 | 13710 | 0 | 0 | 0 | 1 | 1 | 0 | 0 |
|  | F413 | 27 | 52 | 70 | 100 | 107 | 110 | 102 | 0 | 0 | 0 | 0 | 0 | 0 | 0 | 54 | 103 | 129 | 252 | 210 | 230 | 207 | 0 | 0 | 0 | 0 | 0 | 0 | 0 | 81 | 155 | 199 | 352 | 317 | 340 | 309 | 0 | 0 | 0 | 0 | 0 | 0 | 0 |
|  | F418 | 52 | 70 | 131 | 264 | 416 | 520 | 379 | 0 | 0 | 0 | 0 | 0 | 0 | 0 | 108 | 176 | 282 | 497 | 618 | 951 | 837 | 0 | 0 | 1 | 0 | 0 | 0 | 0 | 160 | 246 | 413 | 761 | 1034 | 1471 | 1216 | 0 | 0 | 1 | 0 | 0 | 0 | 0 |
|  | F419 | 417 | 509 | 665 | 977 | 956 | 1349 | 1346 | 0 | 0 | 0 | 0 | 0 | 0 | 0 | 1143 | 1230 | 1428 | 1985 | 1935 | 2841 | 3020 | 0 | 0 | 0 | 0 | 0 | 1 | 0 | 1560 | 1739 | 2093 | 2962 | 2891 | 4190 | 4366 | 0 | 0 | 0 | 0 | 0 | 1 | 0 |
|  | F420 | 0 | 8 | 7 | 22 | 22 | 17 | 22 | 0 | 0 | 0 | 0 | 0 | 0 | 0 | 3 | 5 | 12 | 22 | 22 | 10 | 21 | 0 | 0 | 0 | 0 | 0 | 0 | 0 | 3 | 13 | 19 | 44 | 44 | 27 | 43 | 0 | 0 | 0 | 0 | 0 | 0 | 0 |
|  | F421 | 1 | 0 | 11 | 8 | 9 | 3 | 8 | 0 | 0 | 0 | 0 | 0 | 0 | 0 | 2 | 3 | 1 | 12 | 9 | 4 | 3 | 0 | 0 | 0 | 0 | 0 | 0 | 0 | 3 | 3 | 12 | 20 | 18 | 7 | 11 | 0 | 0 | 0 | 0 | 0 | 0 | 0 |
|  | F422 | 0 | 0 | 3 | 7 | 4 | 1 | 3 | 0 | 0 | 0 | 0 | 0 | 0 | 0 | 1 | 4 | 1 | 11 | 11 | 1 | 0 | 0 | 0 | 0 | 0 | 0 | 0 | 0 | 1 | 4 | 4 | 18 | 15 | 2 | 3 | 0 | 0 | 0 | 0 | 0 | 0 | 0 |
|  | F428 | 12 | 14 | 43 | 28 | 25 | 65 | 86 | 0 | 0 | 0 | 0 | 0 | 0 | 0 | 7 | 15 | 26 | 23 | 22 | 42 | 95 | 0 | 0 | 0 | 0 | 0 | 0 | 0 | 19 | 29 | 69 | 51 | 47 | 107 | 181 | 0 | 0 | 0 | 0 | 0 | 0 | 0 |
|  | F429 | 11 | 14 | 25 | 50 | 34 | 39 | 46 | 0 | 0 | 0 | 0 | 0 | 0 | 0 | 10 | 12 | 27 | 46 | 31 | 46 | 48 | 0 | 0 | 0 | 0 | 0 | 0 | 0 | 21 | 26 | 52 | 96 | 65 | 85 | 94 | 0 | 0 | 0 | 0 | 0 | 0 | 0 |
|  | F430 | 24 | 24 | 50 | 36 | 46 | 48 | 48 | 0 | 0 | 0 | 0 | 0 | 0 | 0 | 49 | 38 | 76 | 108 | 98 | 117 | 158 | 0 | 0 | 0 | 0 | 0 | 0 | 0 | 73 | 62 | 126 | 144 | 144 | 165 | 206 | 0 | 0 | 0 | 0 | 0 | 0 | 0 |
|  | F431 | 33 | 57 | 156 | 148 | 118 | 155 | 161 | 0 | 0 | 0 | 0 | 0 | 0 | 0 | 20 | 36 | 71 | 85 | 67 | 116 | 129 | 0 | 0 | 0 | 0 | 0 | 0 | 0 | 53 | 93 | 227 | 233 | 185 | 271 | 290 | 0 | 0 | 0 | 0 | 0 | 0 | 0 |
|  | F432 | 24 | 22 | 97 | 113 | 94 | 124 | 171 | 0 | 0 | 0 | 0 | 0 | 0 | 0 | 57 | 57 | 100 | 162 | 132 | 233 | 294 | 0 | 0 | 0 | 0 | 0 | 0 | 0 | 81 | 79 | 197 | 275 | 226 | 357 | 465 | 0 | 0 | 0 | 0 | 0 | 0 | 0 |
|  | F438 | 3 | 6 | 9 | 9 | 8 | 3 | 5 | 0 | 0 | 0 | 0 | 0 | 0 | 0 | 12 | 11 | 10 | 27 | 17 | 12 | 26 | 0 | 0 | 0 | 0 | 0 | 0 | 0 | 15 | 17 | 19 | 36 | 25 | 15 | 31 | 0 | 0 | 0 | 0 | 0 | 0 | 0 |
|  | F439 | 4 | 7 | 11 | 17 | 6 | 2 | 4 | 0 | 0 | 0 | 0 | 0 | 0 | 0 | 16 | 12 | 5 | 17 | 13 | 11 | 14 | 0 | 0 | 0 | 0 | 0 | 0 | 0 | 20 | 19 | 16 | 34 | 19 | 13 | 18 | 0 | 0 | 0 | 0 | 0 | 0 | 0 |
| Boyacá | F400 | 5 | 2 | 8 | 2 | 4 | 2 | 5 | 0 | 0 | 0 | 0 | 0 | 0 | 0 | 3 | 2 | 4 | 9 | 4 | 0 | 7 | 0 | 0 | 0 | 0 | 0 | 0 | 0 | 8 | 4 | 12 | 11 | 8 | 2 | 12 | 0 | 0 | 0 | 0 | 0 | 0 | 0 |
|  | F401 | 4 | 11 | 4 | 2 | 0 | 9 | 28 | 0 | 0 | 0 | 0 | 0 | 0 | 0 | 4 | 2 | 5 | 5 | 8 | 23 | 24 | 0 | 0 | 0 | 0 | 0 | 0 | 0 | 8 | 13 | 9 | 7 | 8 | 32 | 52 | 0 | 0 | 0 | 0 | 0 | 0 | 0 |
|  | F402 | 1 | 5 | 5 | 4 | 3 | 2 | 5 | 0 | 0 | 0 | 0 | 0 | 0 | 0 | 6 | 9 | 18 | 19 | 19 | 12 | 15 | 0 | 0 | 0 | 0 | 0 | 0 | 0 | 7 | 14 | 23 | 23 | 22 | 14 | 20 | 0 | 0 | 0 | 0 | 0 | 0 | 0 |
|  | F408 | 1 | 11 | 9 | 11 | 16 | 9 | 23 | 0 | 0 | 0 | 0 | 0 | 0 | 0 | 7 | 15 | 24 | 27 | 20 | 25 | 35 | 0 | 0 | 0 | 0 | 0 | 0 | 0 | 8 | 26 | 33 | 38 | 36 | 34 | 58 | 0 | 0 | 0 | 0 | 0 | 0 | 0 |
|  | F409 | 13 | 12 | 16 | 22 | 32 | 21 | 30 | 0 | 0 | 0 | 0 | 0 | 0 | 0 | 32 | 22 | 40 | 46 | 45 | 28 | 64 | 0 | 0 | 0 | 0 | 0 | 0 | 0 | 45 | 34 | 56 | 68 | 77 | 49 | 94 | 0 | 0 | 0 | 0 | 0 | 0 | 0 |
|  | F410 | 17 | 26 | 40 | 61 | 72 | 58 | 83 | 0 | 0 | 0 | 0 | 0 | 0 | 0 | 42 | 54 | 90 | 134 | 141 | 138 | 212 | 0 | 0 | 0 | 0 | 0 | 0 | 0 | 59 | 80 | 130 | 195 | 213 | 196 | 295 | 0 | 0 | 0 | 0 | 0 | 0 | 0 |
|  | F411 | 82 | 105 | 212 | 288 | 370 | 484 | 679 | 0 | 0 | 0 | 0 | 0 | 0 | 0 | 159 | 237 | 371 | 588 | 702 | 1065 | 1259 | 0 | 0 | 0 | 0 | 0 | 0 | 0 | 241 | 342 | 583 | 876 | 1072 | 1549 | 1938 | 0 | 0 | 0 | 0 | 0 | 0 | 0 |
|  | F412 | 312 | 460 | 877 | 1001 | 1123 | 1332 | 2004 | 0 | 0 | 0 | 0 | 0 | 0 | 0 | 968 | 1417 | 2235 | 2812 | 2840 | 3874 | 5682 | 0 | 0 | 0 | 0 | 0 | 0 | 0 | 1280 | 1877 | 3112 | 3813 | 3963 | 5206 | 7686 | 0 | 0 | 0 | 0 | 0 | 0 | 0 |
|  | F413 | 42 | 68 | 83 | 112 | 120 | 136 | 150 | 0 | 0 | 0 | 0 | 0 | 0 | 0 | 81 | 149 | 184 | 227 | 266 | 335 | 401 | 0 | 0 | 0 | 0 | 0 | 0 | 0 | 123 | 217 | 267 | 339 | 386 | 471 | 551 | 0 | 0 | 0 | 0 | 0 | 0 | 0 |
|  | F418 | 86 | 136 | 205 | 313 | 342 | 313 | 294 | 0 | 0 | 0 | 0 | 0 | 0 | 0 | 177 | 265 | 410 | 704 | 654 | 640 | 603 | 0 | 0 | 0 | 0 | 0 | 0 | 0 | 263 | 401 | 615 | 1017 | 996 | 953 | 897 | 0 | 0 | 0 | 0 | 0 | 0 | 0 |
|  | F419 | 351 | 516 | 634 | 958 | 1161 | 1262 | 1426 | 0 | 0 | 0 | 0 | 0 | 0 | 0 | 669 | 992 | 1255 | 1936 | 2114 | 2583 | 2707 | 0 | 0 | 0 | 0 | 0 | 0 | 0 | 1020 | 1508 | 1889 | 2894 | 3275 | 3845 | 4133 | 0 | 0 | 0 | 0 | 0 | 0 | 0 |
|  | F420 | 5 | 4 | 9 | 13 | 12 | 8 | 19 | 0 | 0 | 0 | 0 | 0 | 0 | 0 | 11 | 13 | 10 | 14 | 20 | 6 | 21 | 0 | 0 | 0 | 0 | 0 | 0 | 0 | 16 | 17 | 19 | 27 | 32 | 14 | 40 | 0 | 0 | 0 | 0 | 0 | 0 | 0 |
|  | F421 | 2 | 1 | 2 | 1 | 7 | 3 | 1 | 0 | 0 | 0 | 0 | 0 | 0 | 0 | 8 | 3 | 1 | 3 | 1 | 2 | 2 | 0 | 0 | 0 | 0 | 0 | 0 | 0 | 10 | 4 | 3 | 4 | 8 | 5 | 3 | 0 | 0 | 0 | 0 | 0 | 0 | 0 |
|  | F422 | 0 | 2 | 7 | 5 | 5 | 2 | 6 | 0 | 0 | 0 | 0 | 0 | 0 | 0 | 1 | 3 | 6 | 1 | 7 | 12 | 19 | 0 | 0 | 0 | 0 | 0 | 0 | 0 | 1 | 5 | 13 | 6 | 12 | 14 | 25 | 0 | 0 | 0 | 0 | 0 | 0 | 0 |
|  | F428 | 3 | 7 | 8 | 16 | 15 | 23 | 36 | 0 | 0 | 0 | 0 | 0 | 0 | 0 | 7 | 9 | 10 | 18 | 15 | 18 | 30 | 0 | 0 | 0 | 0 | 0 | 0 | 0 | 10 | 16 | 18 | 34 | 30 | 41 | 66 | 0 | 0 | 0 | 0 | 0 | 0 | 0 |
|  | F429 | 13 | 16 | 27 | 39 | 33 | 35 | 61 | 0 | 0 | 0 | 0 | 0 | 0 | 0 | 10 | 15 | 25 | 33 | 30 | 26 | 41 | 0 | 0 | 0 | 0 | 0 | 0 | 0 | 23 | 31 | 52 | 72 | 63 | 61 | 102 | 0 | 0 | 0 | 0 | 0 | 0 | 0 |
|  | F430 | 6 | 8 | 49 | 57 | 50 | 16 | 70 | 0 | 0 | 0 | 0 | 0 | 0 | 0 | 26 | 42 | 58 | 111 | 164 | 138 | 240 | 0 | 0 | 0 | 0 | 0 | 0 | 0 | 32 | 50 | 107 | 168 | 214 | 154 | 310 | 0 | 0 | 0 | 0 | 0 | 0 | 0 |
|  | F431 | 12 | 29 | 90 | 126 | 88 | 107 | 152 | 0 | 0 | 0 | 0 | 0 | 0 | 0 | 31 | 26 | 33 | 76 | 55 | 79 | 131 | 0 | 0 | 0 | 0 | 0 | 0 | 0 | 43 | 55 | 123 | 202 | 143 | 186 | 283 | 0 | 0 | 0 | 0 | 0 | 0 | 0 |
|  | F432 | 116 | 123 | 173 | 310 | 239 | 304 | 471 | 0 | 0 | 0 | 0 | 0 | 0 | 0 | 159 | 179 | 326 | 645 | 425 | 518 | 881 | 0 | 0 | 0 | 0 | 0 | 0 | 0 | 275 | 302 | 499 | 955 | 664 | 822 | 1352 | 0 | 0 | 0 | 0 | 0 | 0 | 0 |
|  | F438 | 3 | 0 | 14 | 15 | 3 | 12 | 8 | 0 | 0 | 0 | 0 | 0 | 0 | 0 | 6 | 7 | 8 | 17 | 19 | 17 | 27 | 0 | 0 | 0 | 0 | 0 | 0 | 0 | 9 | 7 | 22 | 32 | 22 | 29 | 35 | 0 | 0 | 0 | 0 | 0 | 0 | 0 |
|  | F439 | 3 | 4 | 18 | 10 | 8 | 11 | 12 | 0 | 0 | 0 | 0 | 0 | 0 | 0 | 8 | 13 | 21 | 37 | 30 | 31 | 27 | 0 | 0 | 0 | 0 | 0 | 0 | 0 | 11 | 17 | 39 | 47 | 38 | 42 | 39 | 0 | 0 | 0 | 0 | 0 | 0 | 0 |
| Caldas | F400 | 1 | 2 | 5 | 4 | 2 | 8 | 5 | 0 | 0 | 0 | 0 | 0 | 0 | 0 | 7 | 14 | 9 | 6 | 4 | 10 | 2 | 0 | 0 | 0 | 0 | 0 | 0 | 0 | 8 | 16 | 14 | 10 | 6 | 18 | 7 | 0 | 0 | 0 | 0 | 0 | 0 | 0 |
|  | F401 | 4 | 3 | 7 | 22 | 10 | 25 | 19 | 0 | 0 | 0 | 0 | 0 | 0 | 0 | 4 | 10 | 6 | 11 | 12 | 20 | 12 | 0 | 0 | 0 | 0 | 0 | 0 | 0 | 8 | 13 | 13 | 33 | 22 | 45 | 31 | 0 | 0 | 0 | 0 | 0 | 0 | 0 |
|  | F402 | 6 | 5 | 1 | 3 | 4 | 15 | 7 | 0 | 0 | 0 | 0 | 0 | 0 | 0 | 8 | 3 | 6 | 15 | 10 | 16 | 17 | 0 | 0 | 0 | 0 | 0 | 0 | 0 | 14 | 8 | 7 | 18 | 14 | 31 | 24 | 0 | 0 | 0 | 0 | 0 | 0 | 0 |
|  | F408 | 1 | 2 | 8 | 24 | 10 | 17 | 10 | 0 | 0 | 0 | 0 | 0 | 0 | 0 | 10 | 14 | 16 | 24 | 28 | 28 | 24 | 0 | 0 | 0 | 0 | 0 | 0 | 0 | 11 | 16 | 24 | 48 | 38 | 45 | 34 | 0 | 0 | 0 | 0 | 0 | 0 | 0 |
|  | F409 | 9 | 7 | 14 | 21 | 23 | 18 | 10 | 0 | 0 | 0 | 0 | 0 | 0 | 0 | 21 | 24 | 18 | 29 | 33 | 49 | 42 | 0 | 0 | 0 | 0 | 0 | 0 | 0 | 30 | 31 | 32 | 50 | 56 | 67 | 52 | 0 | 0 | 0 | 0 | 0 | 0 | 0 |
|  | F410 | 45 | 54 | 66 | 114 | 108 | 119 | 149 | 0 | 0 | 0 | 0 | 0 | 0 | 0 | 94 | 127 | 167 | 225 | 199 | 278 | 316 | 0 | 0 | 0 | 0 | 0 | 0 | 0 | 139 | 181 | 233 | 339 | 307 | 397 | 465 | 0 | 0 | 0 | 0 | 0 | 0 | 0 |
|  | F411 | 142 | 166 | 206 | 441 | 349 | 419 | 399 | 0 | 0 | 0 | 0 | 0 | 0 | 0 | 363 | 375 | 433 | 1157 | 881 | 1069 | 907 | 0 | 0 | 0 | 0 | 0 | 0 | 0 | 505 | 541 | 639 | 1598 | 1230 | 1488 | 1306 | 0 | 0 | 0 | 0 | 0 | 0 | 0 |
|  | F412 | 537 | 679 | 999 | 2074 | 2179 | 2377 | 2253 | 0 | 0 | 0 | 0 | 0 | 0 | 0 | 1980 | 2242 | 3176 | 6066 | 6019 | 6463 | 6741 | 0 | 0 | 0 | 0 | 0 | 0 | 0 | 2517 | 2921 | 4175 | 8140 | 8198 | 8840 | 8994 | 0 | 0 | 0 | 0 | 0 | 0 | 0 |
|  | F413 | 36 | 51 | 109 | 199 | 247 | 262 | 182 | 0 | 0 | 0 | 0 | 0 | 0 | 0 | 121 | 174 | 305 | 526 | 513 | 708 | 518 | 0 | 0 | 0 | 0 | 0 | 0 | 0 | 157 | 225 | 414 | 725 | 760 | 970 | 700 | 0 | 0 | 0 | 0 | 0 | 0 | 0 |
|  | F418 | 121 | 136 | 167 | 521 | 735 | 844 | 732 | 0 | 0 | 0 | 0 | 0 | 0 | 0 | 261 | 328 | 399 | 1059 | 1333 | 2038 | 1557 | 0 | 0 | 0 | 0 | 0 | 0 | 0 | 382 | 464 | 566 | 1580 | 2068 | 2882 | 2289 | 0 | 0 | 0 | 0 | 0 | 0 | 0 |
|  | F419 | 513 | 583 | 767 | 1575 | 1706 | 1730 | 1384 | 0 | 0 | 0 | 0 | 0 | 0 | 0 | 1428 | 1340 | 1863 | 3622 | 3565 | 3887 | 3090 | 0 | 0 | 0 | 0 | 0 | 0 | 0 | 1941 | 1923 | 2630 | 5197 | 5271 | 5617 | 4474 | 0 | 0 | 0 | 0 | 0 | 0 | 0 |
|  | F420 | 2 | 6 | 2 | 9 | 7 | 17 | 10 | 0 | 0 | 0 | 0 | 0 | 0 | 0 | 0 | 4 | 5 | 9 | 2 | 21 | 15 | 0 | 0 | 0 | 0 | 0 | 0 | 0 | 2 | 10 | 7 | 18 | 9 | 38 | 25 | 0 | 0 | 0 | 0 | 0 | 0 | 0 |
|  | F421 | 2 | 1 | 0 | 3 | 1 | 4 | 7 | 0 | 0 | 0 | 0 | 0 | 0 | 0 | 2 | 0 | 3 | 3 | 6 | 9 | 9 | 0 | 0 | 0 | 0 | 0 | 0 | 0 | 4 | 1 | 3 | 6 | 7 | 13 | 16 | 0 | 0 | 0 | 0 | 0 | 0 | 0 |
|  | F422 | 2 | 1 | 6 | 6 | 3 | 10 | 18 | 0 | 0 | 0 | 0 | 0 | 0 | 0 | 2 | 3 | 2 | 5 | 1 | 0 | 9 | 0 | 0 | 0 | 0 | 0 | 0 | 0 | 4 | 4 | 8 | 11 | 4 | 10 | 27 | 0 | 0 | 0 | 0 | 0 | 0 | 0 |
|  | F428 | 10 | 4 | 14 | 26 | 11 | 21 | 16 | 0 | 0 | 0 | 0 | 0 | 0 | 0 | 7 | 9 | 14 | 18 | 20 | 26 | 29 | 0 | 0 | 0 | 0 | 0 | 0 | 0 | 17 | 13 | 28 | 44 | 31 | 47 | 45 | 0 | 0 | 0 | 0 | 0 | 0 | 0 |
|  | F429 | 33 | 30 | 50 | 63 | 39 | 61 | 68 | 0 | 0 | 0 | 0 | 0 | 0 | 0 | 44 | 36 | 59 | 66 | 40 | 52 | 58 | 0 | 0 | 0 | 0 | 0 | 0 | 0 | 77 | 66 | 109 | 129 | 79 | 113 | 126 | 0 | 0 | 0 | 0 | 0 | 0 | 0 |
|  | F430 | 15 | 8 | 38 | 48 | 78 | 26 | 51 | 0 | 0 | 0 | 0 | 0 | 0 | 0 | 65 | 44 | 91 | 125 | 85 | 96 | 108 | 0 | 0 | 0 | 0 | 0 | 0 | 0 | 80 | 52 | 129 | 173 | 163 | 122 | 159 | 0 | 0 | 0 | 0 | 0 | 0 | 0 |
|  | F431 | 35 | 37 | 73 | 106 | 91 | 109 | 117 | 0 | 0 | 0 | 0 | 0 | 0 | 0 | 46 | 59 | 64 | 85 | 54 | 89 | 109 | 0 | 0 | 0 | 0 | 0 | 0 | 0 | 81 | 96 | 137 | 191 | 145 | 198 | 226 | 0 | 0 | 0 | 0 | 0 | 0 | 0 |
|  | F432 | 234 | 250 | 316 | 470 | 398 | 517 | 973 | 0 | 0 | 0 | 0 | 0 | 0 | 0 | 597 | 541 | 623 | 884 | 828 | 1068 | 1972 | 0 | 0 | 0 | 0 | 0 | 0 | 0 | 831 | 791 | 939 | 1354 | 1226 | 1585 | 2945 | 0 | 0 | 0 | 0 | 0 | 0 | 0 |
|  | F438 | 10 | 5 | 10 | 8 | 4 | 11 | 15 | 0 | 0 | 0 | 0 | 0 | 0 | 0 | 18 | 9 | 13 | 21 | 16 | 28 | 62 | 0 | 0 | 0 | 0 | 0 | 0 | 0 | 28 | 14 | 23 | 29 | 20 | 39 | 77 | 0 | 0 | 0 | 0 | 0 | 0 | 0 |
|  | F439 | 16 | 13 | 9 | 7 | 5 | 5 | 13 | 0 | 0 | 0 | 0 | 0 | 0 | 0 | 17 | 13 | 15 | 8 | 7 | 10 | 39 | 0 | 0 | 0 | 0 | 0 | 0 | 0 | 33 | 26 | 24 | 15 | 12 | 15 | 52 | 0 | 0 | 0 | 0 | 0 | 0 | 0 |
| Caquetá | F400 | 0 | 0 | 1 | 1 | 0 | 0 | 0 | 0 | 0 | 0 | 0 | 0 | 0 | 0 | 0 | 0 | 0 | 0 | 0 | 0 | 1 | 0 | 0 | 0 | 0 | 0 | 0 | 0 | 0 | 0 | 1 | 1 | 0 | 0 | 1 | 0 | 0 | 0 | 0 | 0 | 0 | 0 |
|  | F401 | 1 | 1 | 0 | 1 | 0 | 2 | 0 | 0 | 0 | 0 | 0 | 0 | 0 | 0 | 0 | 0 | 0 | 0 | 1 | 0 | 1 | 0 | 0 | 0 | 0 | 0 | 0 | 0 | 1 | 1 | 0 | 1 | 1 | 2 | 1 | 0 | 0 | 0 | 0 | 0 | 0 | 0 |
|  | F402 | 1 | 2 | 2 | 3 | 1 | 2 | 2 | 0 | 0 | 0 | 0 | 0 | 0 | 0 | 1 | 0 | 2 | 2 | 0 | 1 | 1 | 0 | 0 | 0 | 0 | 0 | 0 | 0 | 2 | 2 | 4 | 5 | 1 | 3 | 3 | 0 | 0 | 0 | 0 | 0 | 0 | 0 |
|  | F408 | 1 | 2 | 2 | 3 | 1 | 1 | 2 | 0 | 0 | 0 | 0 | 0 | 0 | 0 | 7 | 5 | 6 | 7 | 2 | 1 | 13 | 0 | 0 | 0 | 0 | 0 | 0 | 0 | 8 | 7 | 8 | 10 | 3 | 2 | 15 | 0 | 0 | 0 | 0 | 0 | 0 | 0 |
|  | F409 | 2 | 5 | 6 | 6 | 5 | 5 | 11 | 0 | 0 | 0 | 0 | 0 | 0 | 0 | 6 | 4 | 8 | 12 | 13 | 11 | 14 | 0 | 0 | 0 | 0 | 0 | 0 | 0 | 8 | 9 | 14 | 18 | 18 | 16 | 25 | 0 | 0 | 0 | 0 | 0 | 0 | 0 |
|  | F410 | 3 | 1 | 3 | 16 | 11 | 7 | 10 | 0 | 0 | 0 | 0 | 0 | 0 | 0 | 14 | 9 | 19 | 16 | 16 | 29 | 22 | 0 | 0 | 0 | 0 | 0 | 0 | 0 | 17 | 10 | 22 | 32 | 27 | 36 | 32 | 0 | 0 | 0 | 0 | 0 | 0 | 0 |
|  | F411 | 28 | 36 | 47 | 63 | 85 | 89 | 84 | 0 | 0 | 0 | 0 | 0 | 0 | 0 | 38 | 74 | 98 | 139 | 124 | 175 | 162 | 0 | 0 | 0 | 0 | 0 | 0 | 0 | 66 | 110 | 145 | 202 | 209 | 264 | 246 | 0 | 0 | 0 | 0 | 0 | 0 | 0 |
|  | F412 | 54 | 66 | 121 | 203 | 158 | 277 | 382 | 0 | 0 | 0 | 0 | 0 | 0 | 0 | 179 | 191 | 291 | 393 | 465 | 773 | 1001 | 0 | 0 | 0 | 0 | 0 | 0 | 0 | 233 | 257 | 412 | 596 | 623 | 1050 | 1383 | 0 | 0 | 0 | 0 | 0 | 0 | 0 |
|  | F413 | 5 | 6 | 10 | 13 | 24 | 16 | 19 | 0 | 0 | 0 | 0 | 0 | 0 | 0 | 7 | 12 | 18 | 14 | 13 | 25 | 42 | 0 | 0 | 0 | 0 | 0 | 0 | 0 | 12 | 18 | 28 | 27 | 37 | 41 | 61 | 0 | 0 | 0 | 0 | 0 | 0 | 0 |
|  | F418 | 7 | 6 | 4 | 14 | 18 | 19 | 31 | 0 | 0 | 0 | 0 | 0 | 0 | 0 | 5 | 17 | 25 | 28 | 27 | 48 | 41 | 0 | 0 | 0 | 0 | 0 | 0 | 0 | 12 | 23 | 29 | 42 | 45 | 67 | 72 | 0 | 0 | 0 | 0 | 0 | 0 | 0 |
|  | F419 | 56 | 68 | 109 | 124 | 144 | 213 | 298 | 0 | 0 | 0 | 0 | 0 | 0 | 0 | 134 | 153 | 167 | 274 | 278 | 391 | 584 | 0 | 0 | 0 | 0 | 0 | 0 | 0 | 190 | 221 | 276 | 398 | 422 | 604 | 882 | 0 | 0 | 0 | 0 | 0 | 0 | 0 |
|  | F420 | 0 | 1 | 2 | 2 | 2 | 2 | 0 | 0 | 0 | 0 | 0 | 0 | 0 | 0 | 0 | 1 | 0 | 1 | 0 | 2 | 0 | 0 | 0 | 0 | 0 | 0 | 0 | 0 | 0 | 2 | 2 | 3 | 2 | 4 | 0 | 0 | 0 | 0 | 0 | 0 | 0 | 0 |
|  | F421 | 1 | 0 | 0 | 0 | 0 | 0 | 0 | 0 | 0 | 0 | 0 | 0 | 0 | 0 | 0 | 0 | 0 | 0 | 0 | 0 | 0 | 0 | 0 | 0 | 0 | 0 | 0 | 0 | 1 | 0 | 0 | 0 | 0 | 0 | 0 | 0 | 0 | 0 | 0 | 0 | 0 | 0 |
|  | F422 | 0 | 0 | 0 | 0 | 1 | 1 | 0 | 0 | 0 | 0 | 0 | 0 | 0 | 0 | 0 | 0 | 0 | 0 | 0 | 0 | 0 | 0 | 0 | 0 | 0 | 0 | 0 | 0 | 0 | 0 | 0 | 0 | 1 | 1 | 0 | 0 | 0 | 0 | 0 | 0 | 0 | 0 |
|  | F428 | 2 | 1 | 0 | 1 | 1 | 1 | 0 | 0 | 0 | 0 | 0 | 0 | 0 | 0 | 0 | 0 | 0 | 1 | 1 | 0 | 0 | 0 | 0 | 0 | 0 | 0 | 0 | 0 | 2 | 1 | 0 | 2 | 2 | 1 | 0 | 0 | 0 | 0 | 0 | 0 | 0 | 0 |
|  | F429 | 0 | 0 | 1 | 4 | 2 | 4 | 3 | 0 | 0 | 0 | 0 | 0 | 0 | 0 | 2 | 2 | 3 | 4 | 3 | 6 | 3 | 0 | 0 | 0 | 0 | 0 | 0 | 0 | 2 | 2 | 4 | 8 | 5 | 10 | 6 | 0 | 0 | 0 | 0 | 0 | 0 | 0 |
|  | F430 | 4 | 7 | 6 | 6 | 5 | 6 | 7 | 0 | 0 | 0 | 0 | 0 | 0 | 0 | 4 | 10 | 9 | 24 | 16 | 52 | 27 | 0 | 0 | 0 | 0 | 0 | 0 | 0 | 8 | 17 | 15 | 30 | 21 | 58 | 34 | 0 | 0 | 0 | 0 | 0 | 0 | 0 |
|  | F431 | 8 | 12 | 35 | 42 | 30 | 32 | 49 | 0 | 0 | 0 | 0 | 0 | 0 | 0 | 10 | 7 | 7 | 10 | 17 | 21 | 39 | 0 | 0 | 0 | 0 | 0 | 0 | 0 | 18 | 19 | 42 | 52 | 47 | 53 | 88 | 0 | 0 | 0 | 0 | 0 | 0 | 0 |
|  | F432 | 1 | 2 | 9 | 3 | 8 | 10 | 113 | 0 | 0 | 0 | 0 | 0 | 0 | 0 | 7 | 2 | 6 | 4 | 9 | 17 | 207 | 0 | 0 | 0 | 0 | 0 | 0 | 0 | 8 | 4 | 15 | 7 | 17 | 27 | 320 | 0 | 0 | 0 | 0 | 0 | 0 | 0 |
|  | F438 | 0 | 2 | 0 | 2 | 4 | 0 | 0 | 0 | 0 | 0 | 0 | 0 | 0 | 0 | 0 | 6 | 1 | 5 | 7 | 1 | 7 | 0 | 0 | 0 | 0 | 0 | 0 | 0 | 0 | 8 | 1 | 7 | 11 | 1 | 7 | 0 | 0 | 0 | 0 | 0 | 0 | 0 |
|  | F439 | 0 | 1 | 1 | 5 | 2 | 10 | 21 | 0 | 0 | 0 | 0 | 0 | 0 | 0 | 4 | 2 | 0 | 4 | 6 | 73 | 107 | 0 | 0 | 0 | 0 | 0 | 0 | 0 | 4 | 3 | 1 | 9 | 8 | 83 | 128 | 0 | 0 | 0 | 0 | 0 | 0 | 0 |
| Casanare | F400 | 0 | 0 | 0 | 0 | 0 | 1 | 0 | 0 | 0 | 0 | 0 | 0 | 0 | 0 | 0 | 2 | 0 | 1 | 0 | 1 | 0 | 0 | 0 | 0 | 0 | 0 | 0 | 0 | 0 | 2 | 0 | 1 | 0 | 2 | 0 | 0 | 0 | 0 | 0 | 0 | 0 | 0 |
|  | F401 | 0 | 0 | 2 | 4 | 1 | 2 | 1 | 0 | 0 | 0 | 0 | 0 | 0 | 0 | 0 | 2 | 0 | 1 | 1 | 0 | 1 | 0 | 0 | 0 | 0 | 0 | 0 | 0 | 0 | 2 | 2 | 5 | 2 | 2 | 2 | 0 | 0 | 0 | 0 | 0 | 0 | 0 |
|  | F402 | 0 | 0 | 2 | 0 | 0 | 3 | 0 | 0 | 0 | 0 | 0 | 0 | 0 | 0 | 1 | 2 | 0 | 5 | 1 | 3 | 0 | 0 | 0 | 0 | 0 | 0 | 0 | 0 | 1 | 2 | 2 | 5 | 1 | 6 | 0 | 0 | 0 | 0 | 0 | 0 | 0 | 0 |
|  | F408 | 1 | 2 | 0 | 1 | 4 | 2 | 2 | 0 | 0 | 0 | 0 | 0 | 0 | 0 | 3 | 4 | 2 | 6 | 1 | 7 | 1 | 0 | 0 | 0 | 0 | 0 | 0 | 0 | 4 | 6 | 2 | 7 | 5 | 9 | 3 | 0 | 0 | 0 | 0 | 0 | 0 | 0 |
|  | F409 | 3 | 4 | 2 | 4 | 3 | 7 | 5 | 0 | 0 | 0 | 0 | 0 | 0 | 0 | 3 | 8 | 4 | 7 | 2 | 11 | 7 | 0 | 0 | 0 | 0 | 0 | 0 | 0 | 6 | 12 | 6 | 11 | 5 | 18 | 12 | 0 | 0 | 0 | 0 | 0 | 0 | 0 |
|  | F410 | 1 | 7 | 6 | 10 | 18 | 12 | 15 | 0 | 0 | 0 | 0 | 0 | 0 | 0 | 7 | 13 | 17 | 21 | 12 | 27 | 18 | 0 | 0 | 0 | 0 | 0 | 0 | 0 | 8 | 20 | 23 | 31 | 30 | 39 | 33 | 0 | 0 | 0 | 0 | 0 | 0 | 0 |
|  | F411 | 17 | 33 | 15 | 34 | 57 | 77 | 86 | 0 | 0 | 0 | 0 | 0 | 0 | 0 | 34 | 44 | 30 | 53 | 74 | 132 | 156 | 0 | 0 | 0 | 0 | 0 | 0 | 0 | 51 | 77 | 45 | 87 | 131 | 209 | 242 | 0 | 0 | 0 | 0 | 0 | 0 | 0 |
|  | F412 | 209 | 324 | 244 | 342 | 280 | 481 | 438 | 0 | 0 | 0 | 0 | 0 | 0 | 0 | 503 | 738 | 486 | 803 | 522 | 1006 | 1119 | 0 | 0 | 0 | 0 | 0 | 0 | 0 | 712 | 1062 | 730 | 1145 | 802 | 1487 | 1557 | 0 | 0 | 0 | 0 | 0 | 0 | 0 |
|  | F413 | 13 | 10 | 24 | 41 | 29 | 33 | 43 | 0 | 0 | 0 | 0 | 0 | 0 | 0 | 22 | 20 | 16 | 73 | 38 | 64 | 70 | 0 | 0 | 0 | 0 | 0 | 0 | 0 | 35 | 30 | 40 | 114 | 67 | 97 | 113 | 0 | 0 | 0 | 0 | 0 | 0 | 0 |
|  | F418 | 11 | 15 | 29 | 32 | 51 | 46 | 43 | 0 | 0 | 0 | 0 | 0 | 0 | 0 | 19 | 46 | 56 | 66 | 70 | 93 | 74 | 0 | 0 | 0 | 0 | 0 | 0 | 0 | 30 | 61 | 85 | 98 | 121 | 139 | 117 | 0 | 0 | 0 | 0 | 0 | 0 | 0 |
|  | F419 | 52 | 74 | 94 | 149 | 107 | 196 | 156 | 0 | 0 | 0 | 0 | 0 | 0 | 0 | 97 | 129 | 136 | 233 | 173 | 344 | 318 | 0 | 0 | 0 | 0 | 0 | 0 | 0 | 149 | 203 | 230 | 382 | 280 | 540 | 474 | 0 | 0 | 0 | 0 | 0 | 0 | 0 |
|  | F420 | 1 | 15 | 0 | 6 | 4 | 3 | 6 | 0 | 0 | 0 | 0 | 0 | 0 | 0 | 2 | 30 | 3 | 3 | 4 | 6 | 7 | 0 | 0 | 0 | 0 | 0 | 0 | 0 | 3 | 45 | 3 | 9 | 8 | 9 | 13 | 0 | 0 | 0 | 0 | 0 | 0 | 0 |
|  | F421 | 0 | 1 | 1 | 0 | 1 | 4 | 2 | 0 | 0 | 0 | 0 | 0 | 0 | 0 | 0 | 0 | 0 | 1 | 0 | 2 | 0 | 0 | 0 | 0 | 0 | 0 | 0 | 0 | 0 | 1 | 1 | 1 | 1 | 6 | 2 | 0 | 0 | 0 | 0 | 0 | 0 | 0 |
|  | F422 | 0 | 0 | 0 | 0 | 0 | 0 | 0 | 0 | 0 | 0 | 0 | 0 | 0 | 0 | 0 | 0 | 1 | 0 | 0 | 0 | 0 | 0 | 0 | 0 | 0 | 0 | 0 | 0 | 0 | 0 | 1 | 0 | 0 | 0 | 0 | 0 | 0 | 0 | 0 | 0 | 0 | 0 |
|  | F428 | 0 | 1 | 0 | 1 | 1 | 3 | 5 | 0 | 0 | 0 | 0 | 0 | 0 | 0 | 0 | 1 | 2 | 2 | 0 | 2 | 2 | 0 | 0 | 0 | 0 | 0 | 0 | 0 | 0 | 2 | 2 | 3 | 1 | 5 | 7 | 0 | 0 | 0 | 0 | 0 | 0 | 0 |
|  | F429 | 1 | 4 | 2 | 2 | 3 | 4 | 4 | 0 | 0 | 0 | 0 | 0 | 0 | 0 | 3 | 4 | 3 | 6 | 5 | 3 | 4 | 0 | 0 | 0 | 0 | 0 | 0 | 0 | 4 | 8 | 5 | 8 | 8 | 7 | 8 | 0 | 0 | 0 | 0 | 0 | 0 | 0 |
|  | F430 | 6 | 10 | 10 | 16 | 7 | 16 | 35 | 0 | 0 | 0 | 0 | 0 | 0 | 0 | 16 | 19 | 15 | 28 | 26 | 64 | 54 | 0 | 0 | 0 | 0 | 0 | 0 | 0 | 22 | 29 | 25 | 44 | 33 | 80 | 89 | 0 | 0 | 0 | 0 | 0 | 0 | 0 |
|  | F431 | 11 | 16 | 27 | 32 | 24 | 23 | 26 | 0 | 0 | 0 | 0 | 0 | 0 | 0 | 12 | 11 | 19 | 28 | 19 | 50 | 51 | 0 | 0 | 0 | 0 | 0 | 0 | 0 | 23 | 27 | 46 | 60 | 43 | 73 | 77 | 0 | 0 | 0 | 0 | 0 | 0 | 0 |
|  | F432 | 3 | 7 | 9 | 33 | 11 | 39 | 146 | 0 | 0 | 0 | 0 | 0 | 0 | 0 | 14 | 12 | 21 | 32 | 18 | 87 | 324 | 0 | 0 | 0 | 0 | 0 | 0 | 0 | 17 | 19 | 30 | 65 | 29 | 126 | 470 | 0 | 0 | 0 | 0 | 0 | 0 | 0 |
|  | F438 | 0 | 2 | 2 | 2 | 3 | 1 | 0 | 0 | 0 | 0 | 0 | 0 | 0 | 0 | 4 | 2 | 6 | 2 | 8 | 4 | 2 | 0 | 0 | 0 | 0 | 0 | 0 | 0 | 4 | 4 | 8 | 4 | 11 | 5 | 2 | 0 | 0 | 0 | 0 | 0 | 0 | 0 |
|  | F439 | 0 | 0 | 1 | 2 | 0 | 1 | 1 | 0 | 0 | 0 | 0 | 0 | 0 | 0 | 1 | 3 | 2 | 1 | 0 | 5 | 4 | 0 | 0 | 0 | 0 | 0 | 0 | 0 | 1 | 3 | 3 | 3 | 0 | 6 | 5 | 0 | 0 | 0 | 0 | 0 | 0 | 0 |
| Cauca | F400 | 0 | 0 | 1 | 1 | 1 | 4 | 1 | 0 | 0 | 0 | 0 | 0 | 0 | 0 | 2 | 2 | 0 | 5 | 1 | 1 | 2 | 0 | 0 | 0 | 0 | 0 | 0 | 0 | 2 | 2 | 1 | 6 | 2 | 5 | 3 | 0 | 0 | 0 | 0 | 0 | 0 | 0 |
|  | F401 | 4 | 2 | 0 | 3 | 2 | 5 | 7 | 0 | 0 | 0 | 0 | 0 | 0 | 0 | 3 | 1 | 1 | 5 | 3 | 3 | 8 | 0 | 0 | 0 | 0 | 0 | 0 | 0 | 7 | 3 | 1 | 8 | 5 | 8 | 15 | 0 | 0 | 0 | 0 | 0 | 0 | 0 |
|  | F402 | 0 | 3 | 3 | 3 | 4 | 3 | 3 | 0 | 0 | 0 | 0 | 0 | 0 | 0 | 2 | 2 | 4 | 5 | 6 | 7 | 13 | 0 | 0 | 0 | 0 | 0 | 0 | 0 | 2 | 5 | 7 | 8 | 10 | 10 | 16 | 0 | 0 | 0 | 0 | 0 | 0 | 0 |
|  | F408 | 9 | 8 | 8 | 10 | 8 | 9 | 11 | 0 | 0 | 0 | 0 | 0 | 0 | 0 | 16 | 11 | 14 | 21 | 15 | 17 | 17 | 0 | 0 | 0 | 0 | 0 | 0 | 0 | 25 | 19 | 22 | 31 | 23 | 26 | 28 | 0 | 0 | 0 | 0 | 0 | 0 | 0 |
|  | F409 | 15 | 17 | 16 | 37 | 34 | 32 | 26 | 0 | 0 | 0 | 0 | 0 | 0 | 0 | 35 | 35 | 26 | 74 | 66 | 62 | 50 | 0 | 0 | 0 | 0 | 0 | 0 | 0 | 50 | 52 | 42 | 111 | 100 | 94 | 76 | 0 | 0 | 0 | 0 | 0 | 0 | 0 |
|  | F410 | 20 | 29 | 41 | 45 | 43 | 56 | 47 | 0 | 0 | 0 | 0 | 0 | 0 | 0 | 48 | 33 | 34 | 45 | 53 | 82 | 106 | 0 | 0 | 0 | 0 | 0 | 0 | 0 | 68 | 62 | 75 | 90 | 96 | 138 | 153 | 0 | 0 | 0 | 0 | 0 | 0 | 0 |
|  | F411 | 61 | 91 | 107 | 240 | 237 | 327 | 328 | 0 | 0 | 0 | 0 | 0 | 0 | 0 | 139 | 185 | 235 | 471 | 517 | 741 | 730 | 0 | 0 | 0 | 0 | 0 | 0 | 0 | 200 | 276 | 342 | 711 | 754 | 1068 | 1058 | 0 | 0 | 0 | 0 | 0 | 0 | 0 |
|  | F412 | 276 | 389 | 519 | 843 | 794 | 1114 | 1591 | 0 | 0 | 0 | 0 | 0 | 0 | 0 | 805 | 1020 | 1336 | 2372 | 2089 | 3152 | 4088 | 0 | 0 | 0 | 0 | 0 | 0 | 0 | 1081 | 1409 | 1855 | 3215 | 2883 | 4266 | 5679 | 0 | 0 | 0 | 0 | 0 | 0 | 0 |
|  | F413 | 19 | 36 | 48 | 53 | 105 | 131 | 62 | 0 | 0 | 0 | 0 | 0 | 0 | 0 | 34 | 103 | 78 | 140 | 225 | 271 | 180 | 0 | 0 | 0 | 0 | 0 | 0 | 0 | 53 | 139 | 126 | 193 | 330 | 402 | 242 | 0 | 0 | 0 | 0 | 0 | 0 | 0 |
|  | F418 | 50 | 71 | 85 | 134 | 196 | 235 | 141 | 0 | 0 | 0 | 0 | 0 | 0 | 0 | 128 | 191 | 182 | 288 | 378 | 497 | 297 | 0 | 0 | 0 | 0 | 0 | 0 | 0 | 178 | 262 | 267 | 422 | 574 | 732 | 438 | 0 | 0 | 0 | 0 | 0 | 0 | 0 |
|  | F419 | 275 | 292 | 388 | 625 | 617 | 914 | 818 | 0 | 0 | 0 | 0 | 0 | 0 | 0 | 491 | 860 | 1027 | 1302 | 1169 | 2034 | 1659 | 0 | 0 | 0 | 0 | 0 | 0 | 0 | 766 | 1152 | 1415 | 1927 | 1786 | 2948 | 2477 | 0 | 0 | 0 | 0 | 0 | 0 | 0 |
|  | F420 | 3 | 6 | 4 | 6 | 1 | 10 | 6 | 0 | 0 | 0 | 0 | 0 | 0 | 0 | 1 | 15 | 5 | 5 | 11 | 6 | 1 | 0 | 0 | 0 | 0 | 0 | 0 | 0 | 4 | 21 | 9 | 11 | 12 | 16 | 7 | 0 | 0 | 0 | 0 | 0 | 0 | 0 |
|  | F421 | 2 | 4 | 1 | 7 | 5 | 11 | 8 | 0 | 0 | 0 | 0 | 0 | 0 | 0 | 3 | 2 | 2 | 8 | 9 | 4 | 4 | 0 | 0 | 0 | 0 | 0 | 0 | 0 | 5 | 6 | 3 | 15 | 14 | 15 | 12 | 0 | 0 | 0 | 0 | 0 | 0 | 0 |
|  | F422 | 0 | 1 | 0 | 0 | 0 | 1 | 1 | 0 | 0 | 0 | 0 | 0 | 0 | 0 | 1 | 1 | 3 | 3 | 3 | 5 | 3 | 0 | 0 | 0 | 0 | 0 | 0 | 0 | 1 | 2 | 3 | 3 | 3 | 6 | 4 | 0 | 0 | 0 | 0 | 0 | 0 | 0 |
|  | F428 | 5 | 1 | 5 | 14 | 8 | 15 | 14 | 0 | 0 | 0 | 0 | 0 | 0 | 0 | 0 | 1 | 4 | 11 | 10 | 12 | 21 | 0 | 0 | 0 | 0 | 0 | 0 | 0 | 5 | 2 | 9 | 25 | 18 | 27 | 35 | 0 | 0 | 0 | 0 | 0 | 0 | 0 |
|  | F429 | 10 | 17 | 20 | 29 | 16 | 20 | 20 | 0 | 0 | 0 | 0 | 0 | 0 | 0 | 7 | 16 | 11 | 22 | 23 | 26 | 21 | 0 | 0 | 0 | 0 | 0 | 0 | 0 | 17 | 33 | 31 | 51 | 39 | 46 | 41 | 0 | 0 | 0 | 0 | 0 | 0 | 0 |
|  | F430 | 3 | 18 | 22 | 61 | 32 | 52 | 64 | 0 | 0 | 0 | 0 | 0 | 0 | 0 | 17 | 60 | 54 | 107 | 105 | 149 | 182 | 0 | 0 | 0 | 0 | 0 | 0 | 0 | 20 | 78 | 76 | 168 | 137 | 201 | 246 | 0 | 0 | 0 | 0 | 0 | 0 | 0 |
|  | F431 | 42 | 61 | 82 | 160 | 138 | 141 | 162 | 0 | 0 | 0 | 0 | 0 | 0 | 0 | 65 | 88 | 66 | 122 | 94 | 106 | 188 | 0 | 0 | 0 | 0 | 0 | 0 | 0 | 107 | 149 | 148 | 282 | 232 | 247 | 350 | 0 | 0 | 0 | 0 | 0 | 0 | 0 |
|  | F432 | 35 | 36 | 49 | 83 | 63 | 94 | 111 | 0 | 0 | 0 | 0 | 0 | 0 | 0 | 62 | 105 | 70 | 202 | 115 | 160 | 230 | 0 | 0 | 0 | 0 | 0 | 0 | 0 | 97 | 141 | 119 | 285 | 178 | 254 | 341 | 0 | 0 | 0 | 0 | 0 | 0 | 0 |
|  | F438 | 3 | 4 | 1 | 13 | 13 | 15 | 23 | 0 | 0 | 0 | 0 | 0 | 0 | 0 | 1 | 2 | 11 | 37 | 41 | 23 | 64 | 0 | 0 | 0 | 0 | 0 | 0 | 0 | 4 | 6 | 12 | 50 | 54 | 38 | 87 | 0 | 0 | 0 | 0 | 0 | 0 | 0 |
|  | F439 | 1 | 4 | 7 | 13 | 11 | 8 | 11 | 0 | 0 | 0 | 0 | 0 | 0 | 0 | 1 | 16 | 19 | 29 | 22 | 21 | 27 | 0 | 0 | 0 | 0 | 0 | 0 | 0 | 2 | 20 | 26 | 42 | 33 | 29 | 38 | 0 | 0 | 0 | 0 | 0 | 0 | 0 |
| Cesar | F400 | 4 | 5 | 2 | 2 | 0 | 0 | 3 | 0 | 0 | 0 | 0 | 0 | 0 | 0 | 4 | 4 | 5 | 5 | 1 | 2 | 6 | 0 | 0 | 0 | 0 | 0 | 0 | 0 | 8 | 9 | 7 | 7 | 1 | 2 | 9 | 0 | 0 | 0 | 0 | 0 | 0 | 0 |
|  | F401 | 2 | 1 | 3 | 1 | 2 | 5 | 3 | 0 | 0 | 0 | 0 | 0 | 0 | 0 | 0 | 3 | 2 | 0 | 0 | 2 | 2 | 0 | 0 | 0 | 0 | 0 | 0 | 0 | 2 | 4 | 5 | 1 | 2 | 7 | 5 | 0 | 0 | 0 | 0 | 0 | 0 | 0 |
|  | F402 | 3 | 2 | 4 | 6 | 3 | 1 | 4 | 0 | 0 | 0 | 0 | 0 | 0 | 0 | 4 | 2 | 4 | 9 | 4 | 7 | 11 | 0 | 0 | 0 | 0 | 0 | 0 | 0 | 7 | 4 | 8 | 15 | 7 | 8 | 15 | 0 | 0 | 0 | 0 | 0 | 0 | 0 |
|  | F408 | 1 | 3 | 12 | 9 | 6 | 9 | 8 | 0 | 0 | 0 | 0 | 0 | 0 | 0 | 3 | 10 | 6 | 14 | 16 | 22 | 29 | 0 | 0 | 0 | 0 | 0 | 0 | 0 | 4 | 13 | 18 | 23 | 22 | 31 | 37 | 0 | 0 | 0 | 0 | 0 | 0 | 0 |
|  | F409 | 9 | 11 | 7 | 20 | 14 | 23 | 24 | 0 | 0 | 0 | 0 | 0 | 0 | 0 | 17 | 15 | 18 | 40 | 38 | 36 | 40 | 0 | 0 | 0 | 0 | 0 | 0 | 0 | 26 | 26 | 25 | 60 | 52 | 59 | 64 | 0 | 0 | 0 | 0 | 0 | 0 | 0 |
|  | F410 | 16 | 27 | 25 | 35 | 37 | 49 | 57 | 0 | 0 | 0 | 0 | 0 | 0 | 0 | 37 | 36 | 44 | 74 | 89 | 75 | 107 | 0 | 0 | 0 | 0 | 0 | 0 | 0 | 53 | 63 | 69 | 109 | 126 | 124 | 164 | 0 | 0 | 0 | 0 | 0 | 0 | 0 |
|  | F411 | 65 | 81 | 148 | 209 | 267 | 323 | 473 | 0 | 0 | 0 | 0 | 0 | 0 | 0 | 130 | 120 | 245 | 399 | 451 | 596 | 732 | 0 | 0 | 0 | 0 | 0 | 0 | 0 | 195 | 201 | 393 | 608 | 718 | 919 | 1205 | 0 | 0 | 0 | 0 | 0 | 0 | 0 |
|  | F412 | 393 | 670 | 901 | 1442 | 1193 | 1046 | 1537 | 0 | 0 | 0 | 0 | 0 | 0 | 0 | 759 | 1295 | 1617 | 2585 | 2173 | 2312 | 3436 | 0 | 0 | 0 | 0 | 0 | 0 | 0 | 1152 | 1965 | 2518 | 4027 | 3366 | 3358 | 4973 | 0 | 0 | 0 | 0 | 0 | 0 | 0 |
|  | F413 | 31 | 60 | 59 | 62 | 110 | 97 | 86 | 0 | 0 | 0 | 0 | 0 | 0 | 0 | 52 | 96 | 97 | 176 | 223 | 197 | 211 | 0 | 0 | 0 | 0 | 0 | 0 | 0 | 83 | 156 | 156 | 238 | 333 | 294 | 297 | 0 | 0 | 0 | 0 | 0 | 0 | 0 |
|  | F418 | 77 | 62 | 84 | 182 | 160 | 143 | 150 | 0 | 0 | 0 | 0 | 0 | 0 | 0 | 74 | 121 | 146 | 428 | 381 | 472 | 366 | 0 | 0 | 0 | 0 | 0 | 0 | 0 | 151 | 183 | 230 | 610 | 541 | 615 | 516 | 0 | 0 | 0 | 0 | 0 | 0 | 0 |
|  | F419 | 300 | 393 | 520 | 731 | 724 | 1017 | 1005 | 0 | 0 | 0 | 0 | 0 | 0 | 0 | 511 | 706 | 947 | 1371 | 1375 | 1740 | 1896 | 0 | 0 | 0 | 0 | 0 | 0 | 0 | 811 | 1099 | 1467 | 2102 | 2099 | 2757 | 2901 | 0 | 0 | 0 | 0 | 0 | 0 | 0 |
|  | F420 | 1 | 1 | 4 | 1 | 3 | 5 | 7 | 0 | 0 | 0 | 0 | 0 | 0 | 0 | 1 | 4 | 4 | 3 | 2 | 3 | 9 | 0 | 0 | 0 | 0 | 0 | 0 | 0 | 2 | 5 | 8 | 4 | 5 | 8 | 16 | 0 | 0 | 0 | 0 | 0 | 0 | 0 |
|  | F421 | 1 | 0 | 1 | 0 | 0 | 2 | 2 | 0 | 0 | 0 | 0 | 0 | 0 | 0 | 0 | 0 | 1 | 2 | 1 | 2 | 2 | 0 | 0 | 0 | 0 | 0 | 0 | 0 | 1 | 0 | 2 | 2 | 1 | 4 | 4 | 0 | 0 | 0 | 0 | 0 | 0 | 0 |
|  | F422 | 3 | 3 | 1 | 4 | 4 | 2 | 0 | 0 | 0 | 0 | 0 | 0 | 0 | 0 | 2 | 1 | 0 | 1 | 0 | 1 | 0 | 0 | 0 | 0 | 0 | 0 | 0 | 0 | 5 | 4 | 1 | 5 | 4 | 3 | 0 | 0 | 0 | 0 | 0 | 0 | 0 | 0 |
|  | F428 | 3 | 4 | 2 | 5 | 2 | 8 | 5 | 0 | 0 | 0 | 0 | 0 | 0 | 0 | 0 | 0 | 1 | 3 | 1 | 2 | 5 | 0 | 0 | 0 | 0 | 0 | 0 | 0 | 3 | 4 | 3 | 8 | 3 | 10 | 10 | 0 | 0 | 0 | 0 | 0 | 0 | 0 |
|  | F429 | 2 | 7 | 6 | 18 | 12 | 10 | 27 | 0 | 0 | 0 | 0 | 0 | 0 | 0 | 3 | 10 | 10 | 13 | 14 | 10 | 10 | 0 | 0 | 0 | 0 | 0 | 0 | 0 | 5 | 17 | 16 | 31 | 26 | 20 | 37 | 0 | 0 | 0 | 0 | 0 | 0 | 0 |
|  | F430 | 21 | 36 | 32 | 116 | 32 | 37 | 21 | 0 | 0 | 0 | 0 | 0 | 0 | 0 | 96 | 175 | 30 | 67 | 79 | 96 | 61 | 0 | 0 | 0 | 0 | 0 | 0 | 0 | 117 | 211 | 62 | 183 | 111 | 133 | 82 | 0 | 0 | 0 | 0 | 0 | 0 | 0 |
|  | F431 | 47 | 56 | 120 | 169 | 148 | 139 | 153 | 0 | 0 | 0 | 0 | 0 | 0 | 0 | 39 | 58 | 56 | 81 | 61 | 68 | 86 | 0 | 0 | 0 | 0 | 0 | 0 | 0 | 86 | 114 | 176 | 250 | 209 | 207 | 239 | 0 | 0 | 0 | 0 | 0 | 0 | 0 |
|  | F432 | 23 | 21 | 27 | 123 | 110 | 170 | 301 | 0 | 0 | 0 | 0 | 0 | 0 | 0 | 21 | 32 | 46 | 106 | 118 | 233 | 467 | 0 | 0 | 0 | 0 | 0 | 0 | 0 | 44 | 53 | 73 | 229 | 228 | 403 | 768 | 0 | 0 | 0 | 0 | 0 | 0 | 0 |
|  | F438 | 3 | 0 | 1 | 3 | 2 | 1 | 1 | 0 | 0 | 0 | 0 | 0 | 0 | 0 | 5 | 4 | 3 | 3 | 2 | 4 | 7 | 0 | 0 | 0 | 0 | 0 | 0 | 0 | 8 | 4 | 4 | 6 | 4 | 5 | 8 | 0 | 0 | 0 | 0 | 0 | 0 | 0 |
|  | F439 | 3 | 0 | 2 | 3 | 1 | 3 | 2 | 0 | 0 | 0 | 0 | 0 | 0 | 0 | 1 | 2 | 5 | 7 | 4 | 7 | 9 | 0 | 0 | 0 | 0 | 0 | 0 | 0 | 4 | 2 | 7 | 10 | 5 | 10 | 11 | 0 | 0 | 0 | 0 | 0 | 0 | 0 |
| Chocó | F400 | 0 | 0 | 0 | 0 | 0 | 1 | 0 | 0 | 0 | 0 | 0 | 0 | 0 | 0 | 0 | 0 | 0 | 0 | 0 | 0 | 1 | 0 | 0 | 0 | 0 | 0 | 0 | 0 | 0 | 0 | 0 | 0 | 0 | 1 | 1 | 0 | 0 | 0 | 0 | 0 | 0 | 0 |
|  | F401 | 0 | 0 | 0 | 0 | 0 | 1 | 0 | 0 | 0 | 0 | 0 | 0 | 0 | 0 | 1 | 1 | 1 | 2 | 1 | 1 | 1 | 0 | 0 | 0 | 0 | 0 | 0 | 0 | 1 | 1 | 1 | 2 | 1 | 2 | 1 | 0 | 0 | 0 | 0 | 0 | 0 | 0 |
|  | F402 | 0 | 0 | 0 | 1 | 0 | 3 | 2 | 0 | 0 | 0 | 0 | 0 | 0 | 0 | 1 | 1 | 0 | 4 | 1 | 3 | 1 | 0 | 0 | 0 | 0 | 0 | 0 | 0 | 1 | 1 | 0 | 5 | 1 | 6 | 3 | 0 | 0 | 0 | 0 | 0 | 0 | 0 |
|  | F408 | 2 | 0 | 3 | 1 | 2 | 2 | 3 | 0 | 0 | 0 | 0 | 0 | 0 | 0 | 2 | 0 | 2 | 5 | 6 | 6 | 9 | 0 | 0 | 0 | 0 | 0 | 0 | 0 | 4 | 0 | 5 | 6 | 8 | 8 | 12 | 0 | 0 | 0 | 0 | 0 | 0 | 0 |
|  | F409 | 1 | 0 | 4 | 6 | 4 | 148 | 3 | 0 | 0 | 0 | 0 | 0 | 0 | 0 | 11 | 13 | 18 | 9 | 8 | 192 | 8 | 0 | 0 | 0 | 0 | 0 | 0 | 0 | 12 | 13 | 22 | 15 | 12 | 340 | 11 | 0 | 0 | 0 | 0 | 0 | 0 | 0 |
|  | F410 | 12 | 8 | 11 | 9 | 10 | 12 | 10 | 0 | 0 | 0 | 0 | 0 | 0 | 0 | 33 | 21 | 25 | 29 | 23 | 39 | 38 | 0 | 0 | 0 | 0 | 0 | 0 | 0 | 45 | 29 | 36 | 38 | 33 | 51 | 48 | 0 | 0 | 0 | 0 | 0 | 0 | 0 |
|  | F411 | 18 | 19 | 37 | 17 | 22 | 43 | 52 | 0 | 0 | 0 | 0 | 0 | 0 | 0 | 70 | 97 | 76 | 73 | 90 | 139 | 157 | 0 | 0 | 0 | 0 | 0 | 0 | 0 | 88 | 116 | 113 | 90 | 112 | 182 | 209 | 0 | 0 | 0 | 0 | 0 | 0 | 0 |
|  | F412 | 15 | 15 | 27 | 78 | 60 | 52 | 1208 | 0 | 0 | 0 | 0 | 0 | 0 | 0 | 81 | 74 | 118 | 234 | 143 | 240 | 3648 | 0 | 0 | 0 | 0 | 0 | 0 | 0 | 96 | 89 | 145 | 312 | 203 | 292 | 4856 | 0 | 0 | 0 | 0 | 0 | 0 | 0 |
|  | F413 | 0 | 2 | 6 | 7 | 14 | 7 | 4 | 0 | 0 | 0 | 0 | 0 | 0 | 0 | 3 | 1 | 7 | 12 | 19 | 24 | 17 | 0 | 0 | 0 | 0 | 0 | 0 | 0 | 3 | 3 | 13 | 19 | 33 | 31 | 21 | 0 | 0 | 0 | 0 | 0 | 0 | 0 |
|  | F418 | 5 | 7 | 26 | 14 | 16 | 17 | 20 | 0 | 0 | 0 | 0 | 0 | 0 | 0 | 18 | 13 | 10 | 24 | 27 | 44 | 61 | 0 | 0 | 0 | 0 | 0 | 0 | 0 | 23 | 20 | 36 | 38 | 43 | 61 | 81 | 0 | 0 | 0 | 0 | 0 | 0 | 0 |
|  | F419 | 22 | 22 | 35 | 44 | 40 | 56 | 54 | 0 | 0 | 0 | 0 | 0 | 0 | 0 | 78 | 71 | 64 | 114 | 163 | 229 | 222 | 0 | 0 | 0 | 0 | 0 | 0 | 0 | 100 | 93 | 99 | 158 | 203 | 285 | 276 | 0 | 0 | 0 | 0 | 0 | 0 | 0 |
|  | F420 | 1 | 0 | 4 | 4 | 2 | 0 | 2 | 0 | 0 | 0 | 0 | 0 | 0 | 0 | 1 | 2 | 2 | 3 | 1 | 3 | 2 | 0 | 0 | 0 | 0 | 0 | 0 | 0 | 2 | 2 | 6 | 7 | 3 | 3 | 4 | 0 | 0 | 0 | 0 | 0 | 0 | 0 |
|  | F421 | 0 | 0 | 0 | 1 | 0 | 0 | 0 | 0 | 0 | 0 | 0 | 0 | 0 | 0 | 0 | 1 | 1 | 1 | 0 | 1 | 1 | 0 | 0 | 0 | 0 | 0 | 0 | 0 | 0 | 1 | 1 | 2 | 0 | 1 | 1 | 0 | 0 | 0 | 0 | 0 | 0 | 0 |
|  | F422 | 0 | 0 | 0 | 0 | 0 | 0 | 0 | 0 | 0 | 0 | 0 | 0 | 0 | 0 | 0 | 1 | 1 | 1 | 0 | 0 | 0 | 0 | 0 | 0 | 0 | 0 | 0 | 0 | 0 | 1 | 1 | 1 | 0 | 0 | 0 | 0 | 0 | 0 | 0 | 0 | 0 | 0 |
|  | F428 | 0 | 3 | 0 | 0 | 1 | 1 | 0 | 0 | 0 | 0 | 0 | 0 | 0 | 0 | 0 | 0 | 0 | 1 | 2 | 1 | 1 | 0 | 0 | 0 | 0 | 0 | 0 | 0 | 0 | 3 | 0 | 1 | 3 | 2 | 1 | 0 | 0 | 0 | 0 | 0 | 0 | 0 |
|  | F429 | 1 | 0 | 3 | 0 | 1 | 2 | 1 | 0 | 0 | 0 | 0 | 0 | 0 | 0 | 1 | 0 | 3 | 4 | 5 | 5 | 6 | 0 | 0 | 0 | 0 | 0 | 0 | 0 | 2 | 0 | 6 | 4 | 6 | 7 | 7 | 0 | 0 | 0 | 0 | 0 | 0 | 0 |
|  | F430 | 0 | 0 | 6 | 13 | 7 | 2 | 15 | 0 | 0 | 0 | 0 | 0 | 0 | 0 | 7 | 3 | 6 | 8 | 18 | 27 | 79 | 0 | 0 | 0 | 0 | 0 | 0 | 0 | 7 | 3 | 12 | 21 | 25 | 29 | 94 | 0 | 0 | 0 | 0 | 0 | 0 | 0 |
|  | F431 | 7 | 7 | 34 | 15 | 11 | 26 | 16 | 0 | 0 | 0 | 0 | 0 | 0 | 0 | 12 | 19 | 22 | 24 | 18 | 35 | 46 | 0 | 0 | 0 | 0 | 0 | 0 | 0 | 19 | 26 | 56 | 39 | 29 | 61 | 62 | 0 | 0 | 0 | 0 | 0 | 0 | 0 |
|  | F432 | 4 | 5 | 8 | 13 | 9 | 11 | 9 | 0 | 0 | 0 | 0 | 0 | 0 | 0 | 26 | 18 | 17 | 34 | 50 | 68 | 24 | 0 | 0 | 0 | 0 | 0 | 0 | 0 | 30 | 23 | 25 | 47 | 59 | 79 | 33 | 0 | 0 | 0 | 0 | 0 | 0 | 0 |
|  | F438 | 0 | 0 | 0 | 0 | 0 | 0 | 0 | 0 | 0 | 0 | 0 | 0 | 0 | 0 | 2 | 2 | 0 | 1 | 0 | 5 | 3 | 0 | 0 | 0 | 0 | 0 | 0 | 0 | 2 | 2 | 0 | 1 | 0 | 5 | 3 | 0 | 0 | 0 | 0 | 0 | 0 | 0 |
|  | F439 | 0 | 0 | 1 | 3 | 0 | 1 | 0 | 0 | 0 | 0 | 0 | 0 | 0 | 0 | 0 | 1 | 1 | 4 | 3 | 2 | 4 | 0 | 0 | 0 | 0 | 0 | 0 | 0 | 0 | 1 | 2 | 7 | 3 | 3 | 4 | 0 | 0 | 0 | 0 | 0 | 0 | 0 |
| Córdoba | F400 | 3 | 1 | 3 | 10 | 10 | 0 | 3 | 0 | 0 | 0 | 0 | 0 | 0 | 0 | 0 | 0 | 0 | 23 | 13 | 5 | 5 | 0 | 0 | 0 | 0 | 0 | 0 | 0 | 3 | 1 | 3 | 33 | 23 | 5 | 8 | 0 | 0 | 0 | 0 | 0 | 0 | 0 |
|  | F401 | 8 | 5 | 2 | 15 | 11 | 4 | 8 | 0 | 0 | 0 | 0 | 0 | 0 | 0 | 3 | 7 | 4 | 22 | 15 | 6 | 12 | 0 | 0 | 0 | 0 | 0 | 0 | 0 | 11 | 12 | 6 | 37 | 26 | 10 | 20 | 0 | 0 | 0 | 0 | 0 | 0 | 0 |
|  | F402 | 0 | 1 | 4 | 12 | 9 | 3 | 5 | 0 | 0 | 0 | 0 | 0 | 0 | 0 | 2 | 6 | 0 | 18 | 18 | 4 | 6 | 0 | 0 | 0 | 0 | 0 | 0 | 0 | 2 | 7 | 4 | 30 | 27 | 7 | 11 | 0 | 0 | 0 | 0 | 0 | 0 | 0 |
|  | F408 | 6 | 5 | 6 | 35 | 20 | 10 | 8 | 0 | 0 | 0 | 0 | 0 | 0 | 0 | 7 | 11 | 3 | 32 | 36 | 25 | 31 | 0 | 0 | 0 | 0 | 0 | 0 | 0 | 13 | 16 | 9 | 67 | 56 | 35 | 39 | 0 | 0 | 0 | 0 | 0 | 0 | 0 |
|  | F409 | 7 | 7 | 11 | 19 | 27 | 19 | 41 | 0 | 0 | 0 | 0 | 0 | 0 | 0 | 16 | 20 | 27 | 50 | 53 | 53 | 65 | 0 | 0 | 0 | 0 | 0 | 0 | 0 | 23 | 27 | 38 | 69 | 80 | 72 | 106 | 0 | 0 | 0 | 0 | 0 | 0 | 0 |
|  | F410 | 48 | 88 | 104 | 162 | 178 | 195 | 272 | 0 | 0 | 0 | 0 | 0 | 0 | 0 | 121 | 168 | 232 | 321 | 314 | 382 | 527 | 0 | 0 | 0 | 0 | 0 | 0 | 0 | 169 | 256 | 336 | 483 | 492 | 577 | 799 | 0 | 0 | 0 | 0 | 0 | 0 | 0 |
|  | F411 | 60 | 74 | 94 | 178 | 233 | 485 | 949 | 0 | 0 | 0 | 0 | 0 | 0 | 0 | 173 | 179 | 236 | 354 | 538 | 1036 | 1929 | 0 | 0 | 0 | 0 | 0 | 0 | 0 | 233 | 253 | 330 | 532 | 771 | 1521 | 2878 | 0 | 0 | 0 | 0 | 0 | 0 | 0 |
|  | F412 | 211 | 302 | 385 | 707 | 761 | 978 | 1697 | 0 | 0 | 0 | 0 | 0 | 0 | 0 | 618 | 919 | 1167 | 1753 | 1933 | 2839 | 4658 | 0 | 0 | 0 | 0 | 0 | 0 | 0 | 829 | 1221 | 1552 | 2460 | 2694 | 3817 | 6355 | 0 | 0 | 0 | 0 | 0 | 0 | 0 |
|  | F413 | 9 | 29 | 34 | 97 | 73 | 86 | 132 | 0 | 0 | 0 | 0 | 0 | 0 | 0 | 39 | 80 | 119 | 201 | 157 | 264 | 395 | 0 | 0 | 0 | 0 | 0 | 0 | 0 | 48 | 109 | 153 | 298 | 230 | 350 | 527 | 0 | 0 | 0 | 0 | 0 | 0 | 0 |
|  | F418 | 60 | 88 | 105 | 149 | 224 | 245 | 224 | 0 | 0 | 0 | 0 | 0 | 0 | 0 | 139 | 217 | 246 | 340 | 460 | 499 | 601 | 0 | 0 | 0 | 0 | 0 | 0 | 0 | 199 | 305 | 351 | 489 | 684 | 744 | 825 | 0 | 0 | 0 | 0 | 0 | 0 | 0 |
|  | F419 | 214 | 320 | 350 | 475 | 603 | 778 | 1005 | 0 | 0 | 0 | 0 | 0 | 0 | 1 | 503 | 736 | 915 | 1028 | 1184 | 1657 | 2301 | 0 | 0 | 0 | 0 | 0 | 0 | 0 | 717 | 1056 | 1265 | 1503 | 1787 | 2435 | 3306 | 0 | 0 | 0 | 0 | 0 | 0 | 1 |
|  | F420 | 1 | 0 | 3 | 7 | 5 | 2 | 3 | 0 | 0 | 0 | 0 | 0 | 0 | 0 | 3 | 0 | 1 | 15 | 16 | 6 | 8 | 0 | 0 | 0 | 0 | 0 | 0 | 0 | 4 | 0 | 4 | 22 | 21 | 8 | 11 | 0 | 0 | 0 | 0 | 0 | 0 | 0 |
|  | F421 | 1 | 0 | 3 | 6 | 7 | 1 | 5 | 0 | 0 | 0 | 0 | 0 | 0 | 0 | 0 | 0 | 2 | 16 | 17 | 3 | 6 | 0 | 0 | 0 | 0 | 0 | 0 | 0 | 1 | 0 | 5 | 22 | 24 | 4 | 11 | 0 | 0 | 0 | 0 | 0 | 0 | 0 |
|  | F422 | 0 | 2 | 4 | 7 | 8 | 0 | 3 | 0 | 0 | 0 | 0 | 0 | 0 | 0 | 0 | 4 | 0 | 17 | 10 | 4 | 5 | 0 | 0 | 0 | 0 | 0 | 0 | 0 | 0 | 6 | 4 | 24 | 18 | 4 | 8 | 0 | 0 | 0 | 0 | 0 | 0 | 0 |
|  | F428 | 4 | 4 | 1 | 14 | 16 | 7 | 11 | 0 | 0 | 0 | 0 | 0 | 0 | 0 | 3 | 4 | 6 | 29 | 23 | 9 | 11 | 0 | 0 | 0 | 0 | 0 | 0 | 0 | 7 | 8 | 7 | 43 | 39 | 16 | 22 | 0 | 0 | 0 | 0 | 0 | 0 | 0 |
|  | F429 | 13 | 16 | 16 | 32 | 31 | 26 | 32 | 0 | 0 | 0 | 0 | 0 | 0 | 0 | 9 | 9 | 13 | 28 | 23 | 16 | 22 | 0 | 0 | 0 | 0 | 0 | 0 | 0 | 22 | 25 | 29 | 60 | 54 | 42 | 54 | 0 | 0 | 0 | 0 | 0 | 0 | 0 |
|  | F430 | 8 | 10 | 10 | 21 | 19 | 11 | 24 | 0 | 0 | 0 | 0 | 0 | 0 | 0 | 40 | 20 | 25 | 57 | 45 | 53 | 89 | 0 | 0 | 0 | 0 | 0 | 0 | 0 | 48 | 30 | 35 | 78 | 64 | 64 | 113 | 0 | 0 | 0 | 0 | 0 | 0 | 0 |
|  | F431 | 18 | 17 | 81 | 108 | 73 | 73 | 94 | 0 | 0 | 0 | 0 | 0 | 0 | 0 | 13 | 32 | 35 | 67 | 48 | 64 | 87 | 0 | 0 | 0 | 0 | 0 | 0 | 0 | 31 | 49 | 116 | 175 | 121 | 137 | 181 | 0 | 0 | 0 | 0 | 0 | 0 | 0 |
|  | F432 | 14 | 19 | 21 | 47 | 59 | 68 | 93 | 0 | 0 | 0 | 0 | 0 | 0 | 0 | 25 | 31 | 33 | 45 | 88 | 117 | 153 | 0 | 0 | 0 | 0 | 0 | 0 | 0 | 39 | 50 | 54 | 92 | 147 | 185 | 246 | 0 | 0 | 0 | 0 | 0 | 0 | 0 |
|  | F438 | 2 | 2 | 2 | 8 | 7 | 1 | 2 | 0 | 0 | 0 | 0 | 0 | 0 | 0 | 2 | 8 | 4 | 19 | 20 | 5 | 5 | 0 | 0 | 0 | 0 | 0 | 0 | 0 | 4 | 10 | 6 | 27 | 27 | 6 | 7 | 0 | 0 | 0 | 0 | 0 | 0 | 0 |
|  | F439 | 4 | 1 | 0 | 9 | 5 | 1 | 4 | 0 | 0 | 0 | 0 | 0 | 0 | 0 | 1 | 4 | 2 | 19 | 15 | 9 | 11 | 0 | 0 | 0 | 0 | 0 | 0 | 0 | 5 | 5 | 2 | 28 | 20 | 10 | 15 | 0 | 0 | 0 | 0 | 0 | 0 | 0 |
| Cundinamarca | F400 | 6 | 14 | 14 | 7 | 3 | 7 | 7 | 0 | 0 | 0 | 0 | 0 | 0 | 0 | 25 | 58 | 11 | 8 | 13 | 9 | 15 | 0 | 0 | 0 | 0 | 0 | 0 | 0 | 31 | 72 | 25 | 15 | 16 | 16 | 22 | 0 | 0 | 0 | 0 | 0 | 0 | 0 |
|  | F401 | 12 | 10 | 16 | 17 | 12 | 13 | 19 | 0 | 0 | 0 | 0 | 0 | 0 | 0 | 15 | 7 | 21 | 16 | 14 | 18 | 26 | 0 | 0 | 0 | 0 | 0 | 0 | 0 | 27 | 17 | 37 | 33 | 26 | 31 | 45 | 0 | 0 | 0 | 0 | 0 | 0 | 0 |
|  | F402 | 4 | 7 | 16 | 18 | 6 | 15 | 10 | 0 | 0 | 0 | 0 | 0 | 0 | 0 | 16 | 18 | 24 | 23 | 20 | 20 | 28 | 1 | 0 | 0 | 0 | 0 | 0 | 0 | 20 | 25 | 40 | 41 | 26 | 35 | 38 | 1 | 0 | 0 | 0 | 0 | 0 | 0 |
|  | F408 | 11 | 26 | 32 | 19 | 32 | 37 | 29 | 0 | 0 | 0 | 0 | 0 | 0 | 0 | 25 | 28 | 43 | 62 | 55 | 64 | 56 | 0 | 0 | 0 | 0 | 0 | 0 | 0 | 36 | 54 | 75 | 81 | 87 | 101 | 85 | 0 | 0 | 0 | 0 | 0 | 0 | 0 |
|  | F409 | 24 | 34 | 55 | 53 | 69 | 76 | 61 | 0 | 0 | 0 | 0 | 0 | 0 | 0 | 52 | 61 | 113 | 137 | 120 | 121 | 105 | 0 | 0 | 0 | 0 | 0 | 0 | 0 | 76 | 95 | 168 | 190 | 189 | 197 | 166 | 0 | 0 | 0 | 0 | 0 | 0 | 0 |
|  | F410 | 113 | 98 | 124 | 175 | 176 | 165 | 129 | 0 | 0 | 0 | 0 | 0 | 0 | 0 | 200 | 190 | 256 | 280 | 296 | 341 | 258 | 0 | 0 | 0 | 0 | 0 | 0 | 0 | 313 | 288 | 380 | 455 | 472 | 506 | 387 | 0 | 0 | 0 | 0 | 0 | 0 | 0 |
|  | F411 | 265 | 348 | 484 | 651 | 798 | 1027 | 815 | 0 | 0 | 0 | 0 | 0 | 0 | 0 | 642 | 652 | 905 | 1176 | 1507 | 2007 | 1639 | 0 | 0 | 0 | 0 | 0 | 0 | 0 | 907 | 1000 | 1389 | 1827 | 2305 | 3034 | 2454 | 0 | 0 | 0 | 0 | 0 | 0 | 0 |
|  | F412 | 909 | 1059 | 1668 | 2037 | 2017 | 2830 | 2818 | 0 | 0 | 0 | 0 | 1 | 0 | 0 | 3215 | 3762 | 5267 | 6370 | 5802 | 8188 | 8176 | 0 | 0 | 0 | 0 | 0 | 0 | 0 | 4124 | 4821 | 6935 | 8407 | 7819 | 11018 | 10994 | 0 | 0 | 0 | 0 | 1 | 0 | 0 |
|  | F413 | 83 | 120 | 163 | 197 | 234 | 243 | 223 | 0 | 0 | 0 | 0 | 0 | 0 | 0 | 196 | 258 | 357 | 380 | 469 | 559 | 503 | 0 | 0 | 0 | 0 | 0 | 0 | 0 | 279 | 378 | 520 | 577 | 703 | 802 | 726 | 0 | 0 | 0 | 0 | 0 | 0 | 0 |
|  | F418 | 190 | 276 | 394 | 516 | 607 | 721 | 542 | 0 | 0 | 0 | 0 | 0 | 0 | 0 | 384 | 676 | 876 | 1092 | 1154 | 1477 | 1098 | 0 | 0 | 0 | 0 | 0 | 0 | 0 | 574 | 952 | 1270 | 1608 | 1761 | 2198 | 1640 | 0 | 0 | 0 | 0 | 0 | 0 | 0 |
|  | F419 | 831 | 1118 | 1653 | 2098 | 2382 | 3009 | 2552 | 0 | 0 | 0 | 0 | 0 | 0 | 0 | 1868 | 2303 | 3600 | 4452 | 4643 | 6040 | 4940 | 0 | 0 | 0 | 0 | 0 | 0 | 0 | 2699 | 3421 | 5253 | 6550 | 7025 | 9049 | 7492 | 0 | 0 | 0 | 0 | 0 | 0 | 0 |
|  | F420 | 31 | 24 | 26 | 50 | 36 | 21 | 20 | 0 | 0 | 0 | 0 | 0 | 0 | 0 | 18 | 21 | 28 | 41 | 23 | 26 | 37 | 0 | 0 | 0 | 0 | 0 | 0 | 0 | 49 | 45 | 54 | 91 | 59 | 47 | 57 | 0 | 0 | 0 | 0 | 0 | 0 | 0 |
|  | F421 | 1 | 5 | 4 | 3 | 5 | 13 | 8 | 0 | 0 | 0 | 0 | 0 | 0 | 0 | 5 | 3 | 10 | 5 | 4 | 7 | 14 | 0 | 0 | 0 | 0 | 0 | 0 | 0 | 6 | 8 | 14 | 8 | 9 | 20 | 22 | 0 | 0 | 0 | 0 | 0 | 0 | 0 |
|  | F422 | 3 | 5 | 5 | 8 | 6 | 3 | 7 | 0 | 0 | 0 | 0 | 0 | 0 | 0 | 3 | 5 | 3 | 8 | 5 | 4 | 2 | 0 | 0 | 0 | 0 | 0 | 0 | 0 | 6 | 10 | 8 | 16 | 11 | 7 | 9 | 0 | 0 | 0 | 0 | 0 | 0 | 0 |
|  | F428 | 32 | 22 | 30 | 33 | 22 | 41 | 74 | 0 | 0 | 0 | 0 | 0 | 0 | 0 | 15 | 20 | 16 | 15 | 18 | 28 | 86 | 0 | 0 | 0 | 0 | 0 | 0 | 0 | 47 | 42 | 46 | 48 | 40 | 69 | 160 | 0 | 0 | 0 | 0 | 0 | 0 | 0 |
|  | F429 | 26 | 48 | 49 | 60 | 69 | 72 | 92 | 0 | 0 | 0 | 0 | 0 | 0 | 0 | 24 | 22 | 43 | 55 | 93 | 52 | 102 | 0 | 0 | 0 | 0 | 0 | 0 | 0 | 50 | 70 | 92 | 115 | 162 | 124 | 194 | 0 | 0 | 0 | 0 | 0 | 0 | 0 |
|  | F430 | 35 | 53 | 50 | 87 | 95 | 87 | 100 | 0 | 0 | 0 | 0 | 0 | 0 | 0 | 115 | 116 | 152 | 231 | 317 | 451 | 366 | 0 | 0 | 0 | 0 | 0 | 0 | 0 | 150 | 169 | 202 | 318 | 412 | 538 | 466 | 0 | 0 | 0 | 0 | 0 | 0 | 0 |
|  | F431 | 61 | 65 | 68 | 90 | 77 | 96 | 83 | 0 | 0 | 0 | 0 | 0 | 0 | 0 | 108 | 148 | 105 | 136 | 124 | 155 | 145 | 0 | 0 | 0 | 0 | 0 | 0 | 0 | 169 | 213 | 173 | 226 | 201 | 251 | 228 | 0 | 0 | 0 | 0 | 0 | 0 | 0 |
|  | F432 | 352 | 416 | 666 | 764 | 730 | 777 | 659 | 0 | 0 | 0 | 0 | 0 | 0 | 0 | 948 | 1094 | 1619 | 1985 | 1498 | 1785 | 1512 | 0 | 0 | 0 | 0 | 0 | 0 | 0 | 1300 | 1510 | 2285 | 2749 | 2228 | 2562 | 2171 | 0 | 0 | 0 | 0 | 0 | 0 | 0 |
|  | F438 | 9 | 22 | 11 | 10 | 19 | 12 | 15 | 0 | 0 | 0 | 0 | 0 | 0 | 0 | 23 | 48 | 25 | 16 | 33 | 42 | 52 | 0 | 0 | 0 | 0 | 0 | 0 | 0 | 32 | 70 | 36 | 26 | 52 | 54 | 67 | 0 | 0 | 0 | 0 | 0 | 0 | 0 |
|  | F439 | 4 | 10 | 4 | 6 | 8 | 2 | 11 | 0 | 0 | 0 | 0 | 0 | 0 | 0 | 4 | 17 | 15 | 23 | 17 | 23 | 26 | 0 | 0 | 0 | 0 | 0 | 0 | 0 | 8 | 27 | 19 | 29 | 25 | 25 | 37 | 0 | 0 | 0 | 0 | 0 | 0 | 0 |
| Guainía | F408 | 0 | 0 | 0 | 0 | 0 | 0 | 0 | 0 | 0 | 0 | 0 | 0 | 0 | 0 | 0 | 0 | 0 | 1 | 0 | 1 | 0 | 0 | 0 | 0 | 0 | 0 | 0 | 0 | 0 | 0 | 0 | 1 | 0 | 1 | 0 | 0 | 0 | 0 | 0 | 0 | 0 | 0 |
|  | F409 | 0 | 0 | 0 | 0 | 0 | 0 | 1 | 0 | 0 | 0 | 0 | 0 | 0 | 0 | 0 | 0 | 0 | 2 | 1 | 0 | 0 | 0 | 0 | 0 | 0 | 0 | 0 | 0 | 0 | 0 | 0 | 2 | 1 | 0 | 1 | 0 | 0 | 0 | 0 | 0 | 0 | 0 |
|  | F410 | 0 | 0 | 0 | 3 | 2 | 4 | 0 | 0 | 0 | 0 | 0 | 0 | 0 | 0 | 0 | 0 | 0 | 5 | 2 | 4 | 4 | 0 | 0 | 0 | 0 | 0 | 0 | 0 | 0 | 0 | 0 | 8 | 4 | 8 | 4 | 0 | 0 | 0 | 0 | 0 | 0 | 0 |
|  | F411 | 0 | 0 | 0 | 8 | 4 | 4 | 5 | 0 | 0 | 0 | 0 | 0 | 0 | 0 | 0 | 1 | 1 | 10 | 6 | 4 | 5 | 0 | 0 | 0 | 0 | 0 | 0 | 0 | 0 | 1 | 1 | 18 | 10 | 8 | 10 | 0 | 0 | 0 | 0 | 0 | 0 | 0 |
|  | F412 | 1 | 1 | 1 | 17 | 8 | 15 | 13 | 0 | 0 | 0 | 0 | 0 | 0 | 0 | 2 | 3 | 1 | 24 | 31 | 38 | 32 | 0 | 0 | 0 | 0 | 0 | 0 | 0 | 3 | 4 | 2 | 41 | 39 | 53 | 45 | 0 | 0 | 0 | 0 | 0 | 0 | 0 |
|  | F413 | 0 | 0 | 0 | 2 | 1 | 1 | 0 | 0 | 0 | 0 | 0 | 0 | 0 | 0 | 0 | 0 | 0 | 0 | 1 | 3 | 0 | 0 | 0 | 0 | 0 | 0 | 0 | 0 | 0 | 0 | 0 | 2 | 2 | 4 | 0 | 0 | 0 | 0 | 0 | 0 | 0 | 0 |
|  | F418 | 1 | 0 | 0 | 1 | 4 | 3 | 1 | 0 | 0 | 0 | 0 | 0 | 0 | 0 | 0 | 0 | 1 | 1 | 2 | 1 | 1 | 0 | 0 | 0 | 0 | 0 | 0 | 0 | 1 | 0 | 1 | 2 | 6 | 4 | 2 | 0 | 0 | 0 | 0 | 0 | 0 | 0 |
|  | F419 | 3 | 0 | 2 | 12 | 17 | 16 | 14 | 0 | 0 | 0 | 0 | 0 | 0 | 0 | 1 | 0 | 1 | 16 | 15 | 21 | 18 | 0 | 0 | 0 | 0 | 0 | 0 | 0 | 4 | 0 | 3 | 28 | 32 | 37 | 32 | 0 | 0 | 0 | 0 | 0 | 0 | 0 |
|  | F420 | 0 | 0 | 0 | 0 | 1 | 2 | 0 | 0 | 0 | 0 | 0 | 0 | 0 | 0 | 0 | 0 | 0 | 0 | 0 | 0 | 0 | 0 | 0 | 0 | 0 | 0 | 0 | 0 | 0 | 0 | 0 | 0 | 1 | 2 | 0 | 0 | 0 | 0 | 0 | 0 | 0 | 0 |
|  | F422 | 0 | 0 | 0 | 0 | 0 | 1 | 0 | 0 | 0 | 0 | 0 | 0 | 0 | 0 | 0 | 0 | 0 | 0 | 0 | 0 | 0 | 0 | 0 | 0 | 0 | 0 | 0 | 0 | 0 | 0 | 0 | 0 | 0 | 1 | 0 | 0 | 0 | 0 | 0 | 0 | 0 | 0 |
|  | F428 | 0 | 1 | 0 | 0 | 0 | 0 | 0 | 0 | 0 | 0 | 0 | 0 | 0 | 0 | 0 | 0 | 0 | 0 | 0 | 0 | 0 | 0 | 0 | 0 | 0 | 0 | 0 | 0 | 0 | 1 | 0 | 0 | 0 | 0 | 0 | 0 | 0 | 0 | 0 | 0 | 0 | 0 |
|  | F429 | 0 | 0 | 0 | 1 | 0 | 2 | 1 | 0 | 0 | 0 | 0 | 0 | 0 | 0 | 0 | 0 | 0 | 0 | 1 | 0 | 0 | 0 | 0 | 0 | 0 | 0 | 0 | 0 | 0 | 0 | 0 | 1 | 1 | 2 | 1 | 0 | 0 | 0 | 0 | 0 | 0 | 0 |
|  | F430 | 0 | 0 | 0 | 1 | 3 | 0 | 1 | 0 | 0 | 0 | 0 | 0 | 0 | 0 | 0 | 0 | 0 | 0 | 1 | 0 | 3 | 0 | 0 | 0 | 0 | 0 | 0 | 0 | 0 | 0 | 0 | 1 | 4 | 0 | 4 | 0 | 0 | 0 | 0 | 0 | 0 | 0 |
|  | F431 | 0 | 0 | 0 | 1 | 0 | 3 | 2 | 0 | 0 | 0 | 0 | 0 | 0 | 0 | 0 | 0 | 0 | 0 | 1 | 0 | 5 | 0 | 0 | 0 | 0 | 0 | 0 | 0 | 0 | 0 | 0 | 1 | 1 | 3 | 7 | 0 | 0 | 0 | 0 | 0 | 0 | 0 |
|  | F432 | 0 | 0 | 0 | 5 | 2 | 1 | 6 | 0 | 0 | 0 | 0 | 0 | 0 | 0 | 3 | 1 | 1 | 3 | 5 | 3 | 5 | 0 | 0 | 0 | 0 | 0 | 0 | 0 | 3 | 1 | 1 | 8 | 7 | 4 | 11 | 0 | 0 | 0 | 0 | 0 | 0 | 0 |
|  | F438 | 0 | 0 | 0 | 0 | 0 | 0 | 2 | 0 | 0 | 0 | 0 | 0 | 0 | 0 | 0 | 0 | 0 | 0 | 0 | 0 | 0 | 0 | 0 | 0 | 0 | 0 | 0 | 0 | 0 | 0 | 0 | 0 | 0 | 0 | 2 | 0 | 0 | 0 | 0 | 0 | 0 | 0 |
|  | F439 | 0 | 0 | 0 | 0 | 0 | 0 | 1 | 0 | 0 | 0 | 0 | 0 | 0 | 0 | 0 | 0 | 0 | 0 | 0 | 1 | 0 | 0 | 0 | 0 | 0 | 0 | 0 | 0 | 0 | 0 | 0 | 0 | 0 | 1 | 1 | 0 | 0 | 0 | 0 | 0 | 0 | 0 |
| Guaviare | F400 | 0 | 0 | 0 | 0 | 1 | 1 | 0 | 0 | 0 | 0 | 0 | 0 | 0 | 0 | 0 | 0 | 0 | 0 | 2 | 0 | 0 | 0 | 0 | 0 | 0 | 0 | 0 | 0 | 0 | 0 | 0 | 0 | 3 | 1 | 0 | 0 | 0 | 0 | 0 | 0 | 0 | 0 |
|  | F401 | 0 | 0 | 0 | 0 | 0 | 0 | 3 | 0 | 0 | 0 | 0 | 0 | 0 | 0 | 0 | 0 | 0 | 1 | 0 | 0 | 0 | 0 | 0 | 0 | 0 | 0 | 0 | 0 | 0 | 0 | 0 | 1 | 0 | 0 | 3 | 0 | 0 | 0 | 0 | 0 | 0 | 0 |
|  | F402 | 0 | 0 | 0 | 0 | 0 | 0 | 0 | 0 | 0 | 0 | 0 | 0 | 0 | 0 | 0 | 0 | 0 | 0 | 0 | 0 | 3 | 0 | 0 | 0 | 0 | 0 | 0 | 0 | 0 | 0 | 0 | 0 | 0 | 0 | 3 | 0 | 0 | 0 | 0 | 0 | 0 | 0 |
|  | F408 | 0 | 0 | 1 | 0 | 4 | 0 | 2 | 0 | 0 | 0 | 0 | 0 | 0 | 0 | 0 | 0 | 0 | 1 | 2 | 2 | 1 | 0 | 0 | 0 | 0 | 0 | 0 | 0 | 0 | 0 | 1 | 1 | 6 | 2 | 3 | 0 | 0 | 0 | 0 | 0 | 0 | 0 |
|  | F409 | 0 | 0 | 0 | 0 | 2 | 1 | 2 | 0 | 0 | 0 | 0 | 0 | 0 | 0 | 0 | 0 | 2 | 4 | 3 | 0 | 3 | 0 | 0 | 0 | 0 | 0 | 0 | 0 | 0 | 0 | 2 | 4 | 5 | 1 | 5 | 0 | 0 | 0 | 0 | 0 | 0 | 0 |
|  | F410 | 0 | 0 | 8 | 2 | 2 | 5 | 9 | 0 | 0 | 0 | 0 | 0 | 0 | 0 | 1 | 1 | 5 | 4 | 5 | 13 | 13 | 0 | 0 | 0 | 0 | 0 | 0 | 0 | 1 | 1 | 13 | 6 | 7 | 18 | 22 | 0 | 0 | 0 | 0 | 0 | 0 | 0 |
|  | F411 | 1 | 3 | 2 | 7 | 12 | 19 | 22 | 0 | 0 | 0 | 0 | 0 | 0 | 0 | 3 | 2 | 1 | 18 | 26 | 30 | 56 | 0 | 0 | 0 | 0 | 0 | 0 | 0 | 4 | 5 | 3 | 25 | 38 | 49 | 78 | 0 | 0 | 0 | 0 | 0 | 0 | 0 |
|  | F412 | 3 | 7 | 8 | 21 | 22 | 22 | 46 | 0 | 0 | 0 | 0 | 0 | 0 | 0 | 16 | 18 | 16 | 56 | 85 | 76 | 97 | 0 | 0 | 0 | 0 | 0 | 0 | 0 | 19 | 25 | 24 | 77 | 107 | 98 | 143 | 0 | 0 | 0 | 0 | 0 | 0 | 0 |
|  | F413 | 1 | 2 | 5 | 2 | 2 | 1 | 2 | 0 | 0 | 0 | 0 | 0 | 0 | 0 | 0 | 4 | 7 | 10 | 6 | 4 | 3 | 0 | 0 | 0 | 0 | 0 | 0 | 0 | 1 | 6 | 12 | 12 | 8 | 5 | 5 | 0 | 0 | 0 | 0 | 0 | 0 | 0 |
|  | F418 | 2 | 1 | 4 | 11 | 5 | 17 | 5 | 0 | 0 | 0 | 0 | 0 | 0 | 0 | 5 | 2 | 8 | 10 | 19 | 22 | 9 | 0 | 0 | 0 | 0 | 0 | 0 | 0 | 7 | 3 | 12 | 21 | 24 | 39 | 14 | 0 | 0 | 0 | 0 | 0 | 0 | 0 |
|  | F419 | 1 | 6 | 11 | 22 | 23 | 24 | 27 | 0 | 0 | 0 | 0 | 0 | 0 | 0 | 8 | 11 | 6 | 30 | 51 | 53 | 63 | 0 | 0 | 0 | 0 | 0 | 0 | 0 | 9 | 17 | 17 | 52 | 74 | 77 | 90 | 0 | 0 | 0 | 0 | 0 | 0 | 0 |
|  | F420 | 0 | 0 | 0 | 2 | 0 | 0 | 0 | 0 | 0 | 0 | 0 | 0 | 0 | 0 | 0 | 0 | 1 | 3 | 1 | 0 | 0 | 0 | 0 | 0 | 0 | 0 | 0 | 0 | 0 | 0 | 1 | 5 | 1 | 0 | 0 | 0 | 0 | 0 | 0 | 0 | 0 | 0 |
|  | F421 | 0 | 0 | 0 | 0 | 0 | 1 | 0 | 0 | 0 | 0 | 0 | 0 | 0 | 0 | 0 | 0 | 0 | 1 | 0 | 0 | 0 | 0 | 0 | 0 | 0 | 0 | 0 | 0 | 0 | 0 | 0 | 1 | 0 | 1 | 0 | 0 | 0 | 0 | 0 | 0 | 0 | 0 |
|  | F428 | 0 | 0 | 0 | 0 | 2 | 1 | 1 | 0 | 0 | 0 | 0 | 0 | 0 | 0 | 0 | 0 | 0 | 4 | 2 | 1 | 1 | 0 | 0 | 0 | 0 | 0 | 0 | 0 | 0 | 0 | 0 | 4 | 4 | 2 | 2 | 0 | 0 | 0 | 0 | 0 | 0 | 0 |
|  | F429 | 0 | 0 | 0 | 0 | 0 | 0 | 2 | 0 | 0 | 0 | 0 | 0 | 0 | 0 | 0 | 0 | 0 | 0 | 4 | 0 | 1 | 0 | 0 | 0 | 0 | 0 | 0 | 0 | 0 | 0 | 0 | 0 | 4 | 0 | 3 | 0 | 0 | 0 | 0 | 0 | 0 | 0 |
|  | F430 | 0 | 0 | 6 | 1 | 2 | 1 | 3 | 0 | 0 | 0 | 0 | 0 | 0 | 0 | 0 | 0 | 3 | 4 | 6 | 9 | 6 | 0 | 0 | 0 | 0 | 0 | 0 | 0 | 0 | 0 | 9 | 5 | 8 | 10 | 9 | 0 | 0 | 0 | 0 | 0 | 0 | 0 |
|  | F431 | 0 | 1 | 3 | 9 | 2 | 3 | 12 | 0 | 0 | 0 | 0 | 0 | 0 | 0 | 0 | 0 | 0 | 12 | 1 | 2 | 21 | 0 | 0 | 0 | 0 | 0 | 0 | 0 | 0 | 1 | 3 | 21 | 3 | 5 | 33 | 0 | 0 | 0 | 0 | 0 | 0 | 0 |
|  | F432 | 0 | 1 | 1 | 5 | 6 | 4 | 8 | 0 | 0 | 0 | 0 | 0 | 0 | 0 | 2 | 3 | 2 | 9 | 6 | 2 | 4 | 0 | 0 | 0 | 0 | 0 | 0 | 0 | 2 | 4 | 3 | 14 | 12 | 6 | 12 | 0 | 0 | 0 | 0 | 0 | 0 | 0 |
|  | F438 | 0 | 0 | 0 | 0 | 0 | 0 | 0 | 0 | 0 | 0 | 0 | 0 | 0 | 0 | 0 | 0 | 0 | 0 | 0 | 0 | 3 | 0 | 0 | 0 | 0 | 0 | 0 | 0 | 0 | 0 | 0 | 0 | 0 | 0 | 3 | 0 | 0 | 0 | 0 | 0 | 0 | 0 |
|  | F439 | 0 | 0 | 0 | 0 | 0 | 0 | 1 | 0 | 0 | 0 | 0 | 0 | 0 | 0 | 0 | 0 | 0 | 2 | 0 | 0 | 1 | 0 | 0 | 0 | 0 | 0 | 0 | 0 | 0 | 0 | 0 | 2 | 0 | 0 | 2 | 0 | 0 | 0 | 0 | 0 | 0 | 0 |
| Huila | F400 | 1 | 0 | 2 | 3 | 2 | 2 | 1 | 0 | 0 | 0 | 0 | 0 | 0 | 0 | 0 | 0 | 5 | 5 | 7 | 6 | 7 | 0 | 0 | 0 | 0 | 0 | 0 | 0 | 1 | 0 | 7 | 8 | 9 | 8 | 8 | 0 | 0 | 0 | 0 | 0 | 0 | 0 |
|  | F401 | 4 | 6 | 7 | 5 | 5 | 9 | 8 | 0 | 0 | 0 | 0 | 0 | 0 | 0 | 4 | 1 | 2 | 5 | 3 | 5 | 3 | 0 | 0 | 0 | 0 | 0 | 0 | 0 | 8 | 7 | 9 | 10 | 8 | 14 | 11 | 0 | 0 | 0 | 0 | 0 | 0 | 0 |
|  | F402 | 1 | 2 | 6 | 3 | 2 | 5 | 8 | 0 | 0 | 0 | 0 | 0 | 0 | 0 | 2 | 6 | 7 | 9 | 4 | 6 | 8 | 0 | 0 | 0 | 0 | 0 | 0 | 0 | 3 | 8 | 13 | 12 | 6 | 11 | 16 | 0 | 0 | 0 | 0 | 0 | 0 | 0 |
|  | F408 | 4 | 9 | 9 | 17 | 24 | 53 | 21 | 0 | 0 | 0 | 0 | 0 | 0 | 0 | 5 | 13 | 19 | 21 | 22 | 78 | 50 | 0 | 0 | 0 | 0 | 0 | 0 | 0 | 9 | 22 | 28 | 38 | 46 | 131 | 71 | 0 | 0 | 0 | 0 | 0 | 0 | 0 |
|  | F409 | 11 | 17 | 23 | 28 | 29 | 48 | 36 | 0 | 0 | 0 | 0 | 0 | 0 | 0 | 17 | 38 | 38 | 51 | 36 | 70 | 70 | 0 | 0 | 0 | 0 | 0 | 0 | 0 | 28 | 55 | 61 | 79 | 65 | 118 | 106 | 0 | 0 | 0 | 0 | 0 | 0 | 0 |
|  | F410 | 23 | 29 | 23 | 42 | 40 | 55 | 45 | 0 | 0 | 0 | 0 | 0 | 0 | 0 | 29 | 42 | 38 | 70 | 83 | 124 | 82 | 0 | 0 | 0 | 0 | 0 | 0 | 0 | 52 | 71 | 61 | 112 | 123 | 179 | 127 | 0 | 0 | 0 | 0 | 0 | 0 | 0 |
|  | F411 | 130 | 163 | 302 | 613 | 780 | 810 | 658 | 0 | 0 | 0 | 0 | 0 | 0 | 0 | 195 | 318 | 574 | 1051 | 1176 | 1300 | 1041 | 0 | 0 | 0 | 0 | 0 | 0 | 0 | 325 | 481 | 876 | 1664 | 1956 | 2110 | 1699 | 0 | 0 | 0 | 0 | 0 | 0 | 0 |
|  | F412 | 415 | 741 | 441 | 548 | 571 | 886 | 850 | 0 | 0 | 0 | 0 | 0 | 0 | 0 | 889 | 1447 | 1072 | 1280 | 1374 | 2445 | 2017 | 0 | 0 | 0 | 0 | 0 | 0 | 0 | 1304 | 2188 | 1513 | 1828 | 1945 | 3331 | 2867 | 0 | 0 | 0 | 0 | 0 | 0 | 0 |
|  | F413 | 26 | 66 | 45 | 71 | 84 | 168 | 103 | 0 | 0 | 0 | 0 | 0 | 0 | 0 | 58 | 118 | 108 | 129 | 151 | 283 | 210 | 0 | 0 | 0 | 0 | 0 | 0 | 0 | 84 | 184 | 153 | 200 | 235 | 451 | 313 | 0 | 0 | 0 | 0 | 0 | 0 | 0 |
|  | F418 | 75 | 75 | 97 | 145 | 179 | 202 | 113 | 0 | 0 | 0 | 0 | 0 | 0 | 0 | 131 | 141 | 173 | 246 | 317 | 333 | 191 | 0 | 0 | 0 | 0 | 0 | 0 | 0 | 206 | 216 | 270 | 391 | 496 | 535 | 304 | 0 | 0 | 0 | 0 | 0 | 0 | 0 |
|  | F419 | 270 | 423 | 553 | 707 | 961 | 1316 | 1129 | 0 | 0 | 0 | 0 | 0 | 0 | 0 | 571 | 907 | 1053 | 1411 | 1693 | 2203 | 1791 | 0 | 0 | 0 | 0 | 0 | 0 | 0 | 841 | 1330 | 1606 | 2118 | 2654 | 3519 | 2920 | 0 | 0 | 0 | 0 | 0 | 0 | 0 |
|  | F420 | 4 | 5 | 6 | 0 | 3 | 5 | 9 | 0 | 0 | 0 | 0 | 0 | 0 | 0 | 9 | 3 | 4 | 6 | 4 | 5 | 14 | 0 | 0 | 0 | 0 | 0 | 0 | 0 | 13 | 8 | 10 | 6 | 7 | 10 | 23 | 0 | 0 | 0 | 0 | 0 | 0 | 0 |
|  | F421 | 2 | 1 | 3 | 4 | 2 | 3 | 2 | 0 | 0 | 0 | 0 | 0 | 0 | 0 | 0 | 2 | 0 | 3 | 3 | 4 | 3 | 0 | 0 | 0 | 0 | 0 | 0 | 0 | 2 | 3 | 3 | 7 | 5 | 7 | 5 | 0 | 0 | 0 | 0 | 0 | 0 | 0 |
|  | F422 | 0 | 1 | 2 | 0 | 1 | 4 | 1 | 0 | 0 | 0 | 0 | 0 | 0 | 0 | 3 | 2 | 1 | 0 | 2 | 3 | 3 | 0 | 0 | 0 | 0 | 0 | 0 | 0 | 3 | 3 | 3 | 0 | 3 | 7 | 4 | 0 | 0 | 0 | 0 | 0 | 0 | 0 |
|  | F428 | 1 | 2 | 4 | 6 | 6 | 5 | 5 | 0 | 0 | 0 | 0 | 0 | 0 | 0 | 1 | 0 | 1 | 8 | 6 | 6 | 0 | 0 | 0 | 0 | 0 | 0 | 0 | 0 | 2 | 2 | 5 | 14 | 12 | 11 | 5 | 0 | 0 | 0 | 0 | 0 | 0 | 0 |
|  | F429 | 3 | 7 | 15 | 21 | 13 | 17 | 26 | 0 | 0 | 0 | 0 | 0 | 0 | 0 | 8 | 10 | 18 | 17 | 11 | 14 | 13 | 0 | 0 | 0 | 0 | 0 | 0 | 0 | 11 | 17 | 33 | 38 | 24 | 31 | 39 | 0 | 0 | 0 | 0 | 0 | 0 | 0 |
|  | F430 | 3 | 5 | 7 | 13 | 12 | 19 | 14 | 0 | 0 | 0 | 0 | 0 | 0 | 0 | 13 | 28 | 28 | 36 | 40 | 59 | 58 | 0 | 0 | 0 | 0 | 0 | 0 | 0 | 16 | 33 | 35 | 49 | 52 | 78 | 72 | 0 | 0 | 0 | 0 | 0 | 0 | 0 |
|  | F431 | 39 | 36 | 93 | 133 | 102 | 138 | 95 | 0 | 0 | 0 | 0 | 0 | 0 | 0 | 48 | 34 | 38 | 72 | 87 | 95 | 66 | 0 | 0 | 0 | 0 | 0 | 0 | 0 | 87 | 70 | 131 | 205 | 189 | 233 | 161 | 0 | 0 | 0 | 0 | 0 | 0 | 0 |
|  | F432 | 47 | 38 | 67 | 115 | 126 | 243 | 251 | 0 | 0 | 0 | 0 | 0 | 0 | 0 | 56 | 86 | 76 | 136 | 106 | 197 | 312 | 0 | 0 | 0 | 0 | 0 | 0 | 0 | 103 | 124 | 143 | 251 | 232 | 440 | 563 | 0 | 0 | 0 | 0 | 0 | 0 | 0 |
|  | F438 | 1 | 2 | 3 | 1 | 3 | 3 | 5 | 0 | 0 | 0 | 0 | 0 | 0 | 0 | 3 | 3 | 0 | 4 | 4 | 8 | 16 | 0 | 0 | 0 | 0 | 0 | 0 | 0 | 4 | 5 | 3 | 5 | 7 | 11 | 21 | 0 | 0 | 0 | 0 | 0 | 0 | 0 |
|  | F439 | 1 | 2 | 1 | 4 | 6 | 4 | 2 | 0 | 0 | 0 | 0 | 0 | 0 | 0 | 2 | 4 | 0 | 9 | 6 | 12 | 3 | 0 | 0 | 0 | 0 | 0 | 0 | 0 | 3 | 6 | 1 | 13 | 12 | 16 | 5 | 0 | 0 | 0 | 0 | 0 | 0 | 0 |
| La Guajira | F400 | 0 | 0 | 3 | 2 | 1 | 0 | 1 | 0 | 0 | 0 | 0 | 0 | 0 | 0 | 0 | 0 | 0 | 0 | 2 | 3 | 2 | 0 | 0 | 0 | 0 | 0 | 0 | 0 | 0 | 0 | 3 | 2 | 3 | 3 | 3 | 0 | 0 | 0 | 0 | 0 | 0 | 0 |
|  | F401 | 1 | 2 | 2 | 4 | 2 | 4 | 4 | 0 | 0 | 0 | 0 | 0 | 0 | 0 | 0 | 0 | 2 | 2 | 0 | 2 | 2 | 0 | 0 | 0 | 0 | 0 | 0 | 0 | 1 | 2 | 4 | 6 | 2 | 6 | 6 | 0 | 0 | 0 | 0 | 0 | 0 | 0 |
|  | F402 | 0 | 0 | 1 | 2 | 2 | 1 | 1 | 0 | 0 | 0 | 0 | 0 | 0 | 0 | 1 | 2 | 2 | 4 | 2 | 2 | 2 | 0 | 0 | 0 | 0 | 0 | 0 | 0 | 1 | 2 | 3 | 6 | 4 | 3 | 3 | 0 | 0 | 0 | 0 | 0 | 0 | 0 |
|  | F408 | 1 | 1 | 8 | 2 | 3 | 4 | 6 | 0 | 0 | 0 | 0 | 0 | 0 | 0 | 5 | 5 | 8 | 6 | 6 | 9 | 6 | 0 | 0 | 0 | 0 | 0 | 0 | 0 | 6 | 6 | 16 | 8 | 9 | 13 | 12 | 0 | 0 | 0 | 0 | 0 | 0 | 0 |
|  | F409 | 1 | 2 | 5 | 4 | 8 | 5 | 5 | 0 | 0 | 0 | 0 | 0 | 0 | 0 | 4 | 9 | 10 | 13 | 5 | 18 | 25 | 0 | 0 | 0 | 0 | 0 | 0 | 0 | 5 | 11 | 15 | 17 | 13 | 23 | 30 | 0 | 0 | 0 | 0 | 0 | 0 | 0 |
|  | F410 | 1 | 6 | 11 | 19 | 18 | 24 | 26 | 0 | 0 | 0 | 0 | 0 | 0 | 0 | 20 | 20 | 21 | 55 | 48 | 84 | 83 | 0 | 0 | 0 | 0 | 0 | 0 | 0 | 21 | 26 | 32 | 74 | 66 | 108 | 109 | 0 | 0 | 0 | 0 | 0 | 0 | 0 |
|  | F411 | 12 | 14 | 27 | 77 | 44 | 103 | 127 | 0 | 0 | 0 | 0 | 0 | 0 | 0 | 27 | 27 | 49 | 104 | 126 | 204 | 243 | 0 | 0 | 0 | 0 | 0 | 0 | 0 | 39 | 41 | 76 | 181 | 170 | 307 | 370 | 0 | 0 | 0 | 0 | 0 | 0 | 0 |
|  | F412 | 64 | 135 | 188 | 288 | 266 | 345 | 469 | 0 | 0 | 0 | 0 | 0 | 0 | 0 | 213 | 320 | 373 | 746 | 715 | 1200 | 1476 | 0 | 0 | 0 | 0 | 0 | 0 | 0 | 277 | 455 | 561 | 1034 | 981 | 1545 | 1945 | 0 | 0 | 0 | 0 | 0 | 0 | 0 |
|  | F413 | 27 | 21 | 37 | 39 | 58 | 35 | 47 | 0 | 0 | 0 | 0 | 0 | 0 | 0 | 46 | 61 | 60 | 76 | 83 | 72 | 113 | 0 | 0 | 0 | 0 | 0 | 0 | 0 | 73 | 82 | 97 | 115 | 141 | 107 | 160 | 0 | 0 | 0 | 0 | 0 | 0 | 0 |
|  | F418 | 16 | 31 | 40 | 46 | 71 | 90 | 64 | 0 | 0 | 0 | 0 | 0 | 0 | 0 | 47 | 64 | 69 | 143 | 138 | 264 | 145 | 0 | 0 | 0 | 0 | 0 | 0 | 0 | 63 | 95 | 109 | 189 | 209 | 354 | 209 | 0 | 0 | 0 | 0 | 0 | 0 | 0 |
|  | F419 | 40 | 79 | 132 | 223 | 226 | 314 | 284 | 0 | 0 | 0 | 0 | 0 | 0 | 0 | 139 | 197 | 241 | 390 | 446 | 634 | 648 | 0 | 0 | 0 | 0 | 0 | 0 | 0 | 179 | 276 | 373 | 613 | 672 | 948 | 932 | 0 | 0 | 0 | 0 | 0 | 0 | 0 |
|  | F420 | 0 | 1 | 0 | 2 | 3 | 6 | 1 | 0 | 0 | 0 | 0 | 0 | 0 | 0 | 2 | 1 | 3 | 5 | 2 | 2 | 1 | 0 | 0 | 0 | 0 | 0 | 0 | 0 | 2 | 2 | 3 | 7 | 5 | 8 | 2 | 0 | 0 | 0 | 0 | 0 | 0 | 0 |
|  | F421 | 0 | 0 | 0 | 0 | 1 | 1 | 0 | 0 | 0 | 0 | 0 | 0 | 0 | 0 | 0 | 0 | 1 | 2 | 3 | 1 | 0 | 0 | 0 | 0 | 0 | 0 | 0 | 0 | 0 | 0 | 1 | 2 | 4 | 2 | 0 | 0 | 0 | 0 | 0 | 0 | 0 | 0 |
|  | F422 | 0 | 0 | 0 | 0 | 1 | 0 | 0 | 0 | 0 | 0 | 0 | 0 | 0 | 0 | 0 | 0 | 0 | 0 | 0 | 0 | 0 | 0 | 0 | 0 | 0 | 0 | 0 | 0 | 0 | 0 | 0 | 0 | 1 | 0 | 0 | 0 | 0 | 0 | 0 | 0 | 0 | 0 |
|  | F428 | 0 | 1 | 1 | 2 | 2 | 2 | 6 | 0 | 0 | 0 | 0 | 0 | 0 | 0 | 0 | 0 | 0 | 0 | 1 | 0 | 5 | 0 | 0 | 0 | 0 | 0 | 0 | 0 | 0 | 1 | 1 | 2 | 3 | 2 | 11 | 0 | 0 | 0 | 0 | 0 | 0 | 0 |
|  | F429 | 2 | 2 | 3 | 10 | 3 | 6 | 8 | 0 | 0 | 0 | 0 | 0 | 0 | 0 | 2 | 3 | 0 | 6 | 2 | 1 | 5 | 0 | 0 | 0 | 0 | 0 | 0 | 0 | 4 | 5 | 3 | 16 | 5 | 7 | 13 | 0 | 0 | 0 | 0 | 0 | 0 | 0 |
|  | F430 | 2 | 7 | 6 | 6 | 6 | 11 | 28 | 0 | 0 | 0 | 0 | 0 | 0 | 0 | 14 | 8 | 8 | 12 | 10 | 27 | 129 | 0 | 0 | 0 | 0 | 0 | 0 | 0 | 16 | 15 | 14 | 18 | 16 | 38 | 157 | 0 | 0 | 0 | 0 | 0 | 0 | 0 |
|  | F431 | 5 | 14 | 31 | 39 | 30 | 35 | 39 | 0 | 0 | 0 | 0 | 0 | 0 | 0 | 12 | 11 | 9 | 31 | 46 | 35 | 46 | 0 | 0 | 0 | 0 | 0 | 0 | 0 | 17 | 25 | 40 | 70 | 76 | 70 | 85 | 0 | 0 | 0 | 0 | 0 | 0 | 0 |
|  | F432 | 2 | 3 | 3 | 14 | 24 | 45 | 63 | 0 | 0 | 0 | 0 | 0 | 0 | 0 | 4 | 2 | 9 | 18 | 11 | 43 | 63 | 0 | 0 | 0 | 0 | 0 | 0 | 0 | 6 | 5 | 12 | 32 | 35 | 88 | 126 | 0 | 0 | 0 | 0 | 0 | 0 | 0 |
|  | F438 | 0 | 1 | 2 | 3 | 2 | 1 | 3 | 0 | 0 | 0 | 0 | 0 | 0 | 0 | 0 | 2 | 1 | 1 | 3 | 6 | 16 | 0 | 0 | 0 | 0 | 0 | 0 | 0 | 0 | 3 | 3 | 4 | 5 | 7 | 19 | 0 | 0 | 0 | 0 | 0 | 0 | 0 |
|  | F439 | 1 | 0 | 2 | 2 | 2 | 2 | 12 | 0 | 0 | 0 | 0 | 0 | 0 | 0 | 1 | 2 | 2 | 3 | 2 | 3 | 15 | 0 | 0 | 0 | 0 | 0 | 0 | 0 | 2 | 2 | 4 | 5 | 4 | 5 | 27 | 0 | 0 | 0 | 0 | 0 | 0 | 0 |
| Magdalena | F400 | 2 | 1 | 2 | 2 | 4 | 3 | 1 | 0 | 0 | 0 | 0 | 0 | 0 | 0 | 1 | 4 | 5 | 10 | 8 | 2 | 0 | 0 | 0 | 0 | 0 | 0 | 0 | 0 | 3 | 5 | 7 | 12 | 12 | 5 | 1 | 0 | 0 | 0 | 0 | 0 | 0 | 0 |
|  | F401 | 1 | 1 | 3 | 4 | 19 | 7 | 57 | 0 | 0 | 0 | 0 | 0 | 0 | 0 | 0 | 1 | 0 | 15 | 14 | 8 | 22 | 0 | 0 | 0 | 0 | 0 | 0 | 0 | 1 | 2 | 3 | 19 | 33 | 15 | 79 | 0 | 0 | 0 | 0 | 0 | 0 | 0 |
|  | F402 | 1 | 3 | 2 | 4 | 28 | 3 | 3 | 0 | 0 | 0 | 0 | 0 | 0 | 0 | 3 | 6 | 3 | 7 | 29 | 2 | 10 | 0 | 0 | 0 | 0 | 0 | 0 | 0 | 4 | 9 | 5 | 11 | 57 | 5 | 13 | 0 | 0 | 0 | 0 | 0 | 0 | 0 |
|  | F408 | 4 | 3 | 5 | 14 | 37 | 8 | 8 | 0 | 0 | 0 | 0 | 0 | 0 | 0 | 8 | 1 | 11 | 19 | 38 | 22 | 20 | 0 | 0 | 0 | 0 | 0 | 0 | 0 | 12 | 4 | 16 | 33 | 75 | 30 | 28 | 0 | 0 | 0 | 0 | 0 | 0 | 0 |
|  | F409 | 5 | 10 | 13 | 23 | 51 | 25 | 24 | 0 | 0 | 0 | 0 | 0 | 0 | 0 | 15 | 16 | 16 | 39 | 82 | 27 | 51 | 0 | 0 | 0 | 0 | 0 | 0 | 0 | 20 | 26 | 29 | 62 | 133 | 52 | 75 | 0 | 0 | 0 | 0 | 0 | 0 | 0 |
|  | F410 | 34 | 22 | 28 | 43 | 55 | 102 | 75 | 0 | 0 | 0 | 0 | 0 | 0 | 0 | 60 | 35 | 44 | 46 | 94 | 147 | 151 | 0 | 0 | 0 | 0 | 0 | 0 | 0 | 94 | 57 | 72 | 89 | 149 | 249 | 226 | 0 | 0 | 0 | 0 | 0 | 0 | 0 |
|  | F411 | 59 | 119 | 91 | 123 | 246 | 249 | 582 | 0 | 0 | 0 | 0 | 0 | 0 | 0 | 120 | 180 | 285 | 412 | 479 | 454 | 1094 | 0 | 0 | 0 | 0 | 0 | 0 | 0 | 179 | 299 | 376 | 535 | 725 | 703 | 1676 | 0 | 0 | 0 | 0 | 0 | 0 | 0 |
|  | F412 | 247 | 375 | 761 | 897 | 998 | 1067 | 1395 | 0 | 0 | 0 | 0 | 0 | 0 | 0 | 460 | 757 | 1094 | 1626 | 1737 | 2051 | 2906 | 0 | 0 | 0 | 0 | 0 | 0 | 0 | 707 | 1132 | 1855 | 2523 | 2735 | 3118 | 4301 | 0 | 0 | 0 | 0 | 0 | 0 | 0 |
|  | F413 | 18 | 30 | 37 | 97 | 80 | 120 | 92 | 0 | 0 | 0 | 0 | 0 | 0 | 0 | 42 | 41 | 80 | 174 | 191 | 272 | 177 | 0 | 0 | 0 | 0 | 0 | 0 | 0 | 60 | 71 | 117 | 271 | 271 | 392 | 269 | 0 | 0 | 0 | 0 | 0 | 0 | 0 |
|  | F418 | 28 | 47 | 84 | 125 | 209 | 173 | 132 | 0 | 0 | 0 | 0 | 0 | 0 | 0 | 46 | 98 | 130 | 292 | 333 | 352 | 240 | 0 | 0 | 0 | 0 | 0 | 0 | 0 | 74 | 145 | 214 | 417 | 542 | 525 | 372 | 0 | 0 | 0 | 0 | 0 | 0 | 0 |
|  | F419 | 159 | 211 | 292 | 411 | 513 | 597 | 840 | 0 | 0 | 0 | 0 | 0 | 0 | 0 | 375 | 444 | 619 | 877 | 947 | 1295 | 1485 | 0 | 0 | 0 | 0 | 0 | 0 | 0 | 534 | 655 | 911 | 1288 | 1460 | 1892 | 2325 | 0 | 0 | 0 | 0 | 0 | 0 | 0 |
|  | F420 | 2 | 4 | 4 | 4 | 10 | 12 | 9 | 0 | 0 | 0 | 0 | 0 | 0 | 0 | 0 | 0 | 3 | 11 | 14 | 12 | 10 | 0 | 0 | 0 | 0 | 0 | 0 | 0 | 2 | 4 | 7 | 15 | 24 | 24 | 19 | 0 | 0 | 0 | 0 | 0 | 0 | 0 |
|  | F421 | 0 | 0 | 4 | 3 | 3 | 1 | 3 | 0 | 0 | 0 | 0 | 0 | 0 | 0 | 1 | 1 | 2 | 4 | 5 | 2 | 3 | 0 | 0 | 0 | 0 | 0 | 0 | 0 | 1 | 1 | 6 | 7 | 8 | 3 | 6 | 0 | 0 | 0 | 0 | 0 | 0 | 0 |
|  | F422 | 1 | 2 | 4 | 8 | 2 | 2 | 1 | 0 | 0 | 0 | 0 | 0 | 0 | 0 | 2 | 1 | 1 | 6 | 4 | 3 | 2 | 0 | 0 | 0 | 0 | 0 | 0 | 0 | 3 | 3 | 5 | 14 | 6 | 5 | 3 | 0 | 0 | 0 | 0 | 0 | 0 | 0 |
|  | F428 | 2 | 4 | 13 | 23 | 15 | 8 | 7 | 0 | 0 | 0 | 0 | 0 | 0 | 0 | 5 | 1 | 2 | 13 | 20 | 3 | 14 | 0 | 0 | 0 | 0 | 0 | 0 | 0 | 7 | 5 | 15 | 36 | 35 | 11 | 21 | 0 | 0 | 0 | 0 | 0 | 0 | 0 |
|  | F429 | 0 | 9 | 10 | 12 | 15 | 16 | 21 | 0 | 0 | 0 | 0 | 0 | 0 | 0 | 3 | 2 | 3 | 9 | 11 | 9 | 13 | 0 | 0 | 0 | 0 | 0 | 0 | 0 | 3 | 11 | 13 | 21 | 26 | 25 | 34 | 0 | 0 | 0 | 0 | 0 | 0 | 0 |
|  | F430 | 24 | 13 | 20 | 43 | 45 | 11 | 37 | 0 | 0 | 0 | 0 | 0 | 0 | 0 | 50 | 31 | 32 | 79 | 73 | 43 | 158 | 0 | 0 | 0 | 0 | 0 | 0 | 0 | 74 | 44 | 52 | 122 | 118 | 54 | 195 | 0 | 0 | 0 | 0 | 0 | 0 | 0 |
|  | F431 | 56 | 163 | 281 | 329 | 82 | 77 | 91 | 0 | 0 | 0 | 0 | 0 | 0 | 0 | 45 | 170 | 329 | 351 | 41 | 66 | 85 | 0 | 0 | 0 | 0 | 0 | 0 | 0 | 101 | 333 | 610 | 680 | 123 | 143 | 176 | 0 | 0 | 0 | 0 | 0 | 0 | 0 |
|  | F432 | 9 | 14 | 43 | 102 | 58 | 68 | 172 | 0 | 0 | 0 | 0 | 0 | 0 | 0 | 26 | 8 | 14 | 309 | 46 | 79 | 165 | 0 | 0 | 0 | 0 | 0 | 0 | 0 | 35 | 22 | 57 | 411 | 104 | 147 | 337 | 0 | 0 | 0 | 0 | 0 | 0 | 0 |
|  | F438 | 1 | 1 | 0 | 7 | 1 | 1 | 4 | 0 | 0 | 0 | 0 | 0 | 0 | 0 | 0 | 0 | 1 | 11 | 5 | 8 | 18 | 0 | 0 | 0 | 0 | 0 | 0 | 0 | 1 | 1 | 1 | 18 | 6 | 9 | 22 | 0 | 0 | 0 | 0 | 0 | 0 | 0 |
|  | F439 | 1 | 1 | 2 | 9 | 7 | 4 | 4 | 0 | 0 | 0 | 0 | 0 | 0 | 0 | 3 | 0 | 4 | 11 | 10 | 7 | 7 | 0 | 0 | 0 | 0 | 0 | 0 | 0 | 4 | 1 | 6 | 20 | 17 | 11 | 11 | 0 | 0 | 0 | 0 | 0 | 0 | 0 |
| Meta | F400 | 1 | 0 | 0 | 2 | 3 | 4 | 5 | 0 | 0 | 0 | 0 | 0 | 0 | 0 | 0 | 1 | 1 | 1 | 2 | 3 | 0 | 0 | 0 | 0 | 0 | 0 | 0 | 0 | 1 | 1 | 1 | 3 | 5 | 7 | 5 | 0 | 0 | 0 | 0 | 0 | 0 | 0 |
|  | F401 | 4 | 4 | 3 | 3 | 4 | 2 | 6 | 0 | 0 | 0 | 0 | 0 | 0 | 0 | 2 | 1 | 0 | 1 | 1 | 4 | 6 | 0 | 0 | 0 | 0 | 0 | 0 | 0 | 6 | 5 | 3 | 4 | 5 | 6 | 12 | 0 | 0 | 0 | 0 | 0 | 0 | 0 |
|  | F402 | 1 | 1 | 3 | 1 | 3 | 3 | 5 | 0 | 0 | 0 | 0 | 0 | 0 | 0 | 2 | 5 | 3 | 5 | 6 | 9 | 10 | 0 | 0 | 0 | 0 | 0 | 0 | 0 | 3 | 6 | 6 | 6 | 9 | 12 | 15 | 0 | 0 | 0 | 0 | 0 | 0 | 0 |
|  | F408 | 9 | 10 | 4 | 18 | 5 | 10 | 7 | 0 | 0 | 0 | 0 | 0 | 0 | 0 | 9 | 11 | 10 | 19 | 16 | 21 | 22 | 0 | 0 | 0 | 0 | 0 | 0 | 0 | 18 | 21 | 14 | 37 | 21 | 31 | 29 | 0 | 0 | 0 | 0 | 0 | 0 | 0 |
|  | F409 | 4 | 5 | 6 | 9 | 17 | 13 | 13 | 0 | 0 | 0 | 0 | 0 | 0 | 0 | 8 | 8 | 14 | 25 | 29 | 26 | 21 | 0 | 0 | 0 | 0 | 0 | 0 | 0 | 12 | 13 | 20 | 34 | 46 | 39 | 34 | 0 | 0 | 0 | 0 | 0 | 0 | 0 |
|  | F410 | 13 | 25 | 33 | 61 | 58 | 78 | 76 | 0 | 0 | 0 | 0 | 0 | 0 | 0 | 29 | 35 | 49 | 105 | 109 | 156 | 142 | 0 | 0 | 0 | 0 | 0 | 0 | 0 | 42 | 60 | 82 | 166 | 167 | 234 | 218 | 0 | 0 | 0 | 0 | 0 | 0 | 0 |
|  | F411 | 111 | 121 | 118 | 311 | 335 | 413 | 559 | 0 | 0 | 0 | 0 | 0 | 0 | 0 | 203 | 196 | 192 | 576 | 657 | 809 | 1035 | 0 | 0 | 0 | 0 | 0 | 0 | 0 | 314 | 317 | 310 | 887 | 992 | 1222 | 1594 | 0 | 0 | 0 | 0 | 0 | 0 | 0 |
|  | F412 | 135 | 200 | 397 | 841 | 840 | 1033 | 1146 | 0 | 0 | 0 | 0 | 0 | 0 | 0 | 427 | 601 | 869 | 2053 | 2006 | 2307 | 2743 | 0 | 0 | 0 | 0 | 0 | 0 | 0 | 562 | 801 | 1266 | 2894 | 2846 | 3340 | 3889 | 0 | 0 | 0 | 0 | 0 | 0 | 0 |
|  | F413 | 13 | 40 | 88 | 141 | 126 | 138 | 107 | 0 | 0 | 0 | 0 | 0 | 0 | 0 | 53 | 66 | 105 | 154 | 195 | 167 | 177 | 0 | 0 | 0 | 0 | 0 | 0 | 0 | 66 | 106 | 193 | 295 | 321 | 305 | 284 | 0 | 0 | 0 | 0 | 0 | 0 | 0 |
|  | F418 | 31 | 54 | 257 | 249 | 248 | 271 | 345 | 0 | 0 | 0 | 0 | 0 | 0 | 0 | 74 | 93 | 222 | 259 | 296 | 428 | 475 | 0 | 0 | 0 | 0 | 0 | 0 | 0 | 105 | 147 | 479 | 508 | 544 | 699 | 820 | 0 | 0 | 0 | 0 | 0 | 0 | 0 |
|  | F419 | 148 | 241 | 240 | 423 | 515 | 712 | 586 | 0 | 0 | 0 | 0 | 0 | 0 | 0 | 341 | 466 | 480 | 780 | 954 | 1303 | 1096 | 0 | 0 | 0 | 0 | 0 | 0 | 0 | 489 | 707 | 720 | 1203 | 1469 | 2015 | 1682 | 0 | 0 | 0 | 0 | 0 | 0 | 0 |
|  | F420 | 5 | 17 | 20 | 26 | 27 | 30 | 22 | 0 | 0 | 0 | 0 | 0 | 0 | 0 | 5 | 12 | 8 | 22 | 14 | 20 | 32 | 0 | 0 | 0 | 0 | 0 | 0 | 0 | 10 | 29 | 28 | 48 | 41 | 50 | 54 | 0 | 0 | 0 | 0 | 0 | 0 | 0 |
|  | F421 | 0 | 2 | 1 | 5 | 0 | 5 | 5 | 0 | 0 | 0 | 0 | 0 | 0 | 0 | 0 | 0 | 2 | 6 | 2 | 4 | 4 | 0 | 0 | 0 | 0 | 0 | 0 | 0 | 0 | 2 | 3 | 11 | 2 | 9 | 9 | 0 | 0 | 0 | 0 | 0 | 0 | 0 |
|  | F422 | 0 | 0 | 0 | 4 | 5 | 2 | 6 | 0 | 0 | 0 | 0 | 0 | 0 | 0 | 0 | 0 | 0 | 2 | 1 | 2 | 7 | 0 | 0 | 0 | 0 | 0 | 0 | 0 | 0 | 0 | 0 | 6 | 6 | 4 | 13 | 0 | 0 | 0 | 0 | 0 | 0 | 0 |
|  | F428 | 1 | 1 | 11 | 13 | 15 | 15 | 14 | 0 | 0 | 0 | 0 | 0 | 0 | 0 | 1 | 1 | 7 | 26 | 14 | 11 | 12 | 0 | 0 | 0 | 0 | 0 | 0 | 0 | 2 | 2 | 18 | 39 | 29 | 26 | 26 | 0 | 0 | 0 | 0 | 0 | 0 | 0 |
|  | F429 | 5 | 6 | 19 | 27 | 28 | 34 | 48 | 0 | 0 | 0 | 0 | 0 | 0 | 0 | 6 | 8 | 10 | 21 | 13 | 25 | 15 | 0 | 0 | 0 | 0 | 0 | 0 | 0 | 11 | 14 | 29 | 48 | 41 | 59 | 63 | 0 | 0 | 0 | 0 | 0 | 0 | 0 |
|  | F430 | 13 | 4 | 22 | 44 | 26 | 27 | 46 | 0 | 0 | 0 | 0 | 0 | 0 | 0 | 13 | 14 | 22 | 55 | 88 | 132 | 125 | 0 | 0 | 0 | 0 | 0 | 0 | 0 | 26 | 18 | 44 | 99 | 114 | 159 | 171 | 0 | 0 | 0 | 0 | 0 | 0 | 0 |
|  | F431 | 21 | 44 | 111 | 160 | 123 | 110 | 116 | 0 | 0 | 0 | 0 | 0 | 0 | 0 | 31 | 25 | 46 | 102 | 81 | 86 | 99 | 0 | 0 | 0 | 0 | 0 | 0 | 0 | 52 | 69 | 157 | 262 | 204 | 196 | 215 | 0 | 0 | 0 | 0 | 0 | 0 | 0 |
|  | F432 | 58 | 35 | 51 | 154 | 125 | 250 | 268 | 0 | 0 | 0 | 0 | 0 | 0 | 0 | 77 | 71 | 66 | 244 | 230 | 396 | 488 | 0 | 0 | 0 | 0 | 0 | 0 | 0 | 135 | 106 | 117 | 398 | 355 | 646 | 756 | 0 | 0 | 0 | 0 | 0 | 0 | 0 |
|  | F438 | 3 | 4 | 5 | 6 | 9 | 10 | 16 | 0 | 0 | 0 | 0 | 0 | 0 | 0 | 2 | 3 | 5 | 10 | 18 | 26 | 28 | 0 | 0 | 0 | 0 | 0 | 0 | 0 | 5 | 7 | 10 | 16 | 27 | 36 | 44 | 0 | 0 | 0 | 0 | 0 | 0 | 0 |
|  | F439 | 1 | 5 | 4 | 1 | 4 | 3 | 6 | 0 | 0 | 0 | 0 | 0 | 0 | 0 | 0 | 10 | 3 | 13 | 6 | 5 | 9 | 0 | 0 | 0 | 0 | 0 | 0 | 0 | 1 | 15 | 7 | 14 | 10 | 8 | 15 | 0 | 0 | 0 | 0 | 0 | 0 | 0 |
| Nariño | F400 | 2 | 2 | 4 | 2 | 2 | 39 | 1 | 0 | 0 | 0 | 0 | 0 | 0 | 0 | 3 | 3 | 2 | 5 | 4 | 40 | 3 | 0 | 0 | 0 | 0 | 0 | 0 | 0 | 5 | 5 | 6 | 7 | 6 | 79 | 4 | 0 | 0 | 0 | 0 | 0 | 0 | 0 |
|  | F401 | 10 | 15 | 18 | 26 | 20 | 10 | 4 | 0 | 0 | 0 | 0 | 0 | 0 | 0 | 12 | 21 | 17 | 19 | 14 | 19 | 4 | 0 | 0 | 0 | 0 | 0 | 0 | 0 | 22 | 36 | 35 | 45 | 34 | 29 | 8 | 0 | 0 | 0 | 0 | 0 | 0 | 0 |
|  | F402 | 2 | 2 | 4 | 8 | 5 | 5 | 3 | 0 | 0 | 0 | 0 | 0 | 0 | 0 | 13 | 7 | 5 | 13 | 8 | 12 | 8 | 0 | 0 | 0 | 0 | 0 | 0 | 0 | 15 | 9 | 9 | 21 | 13 | 17 | 11 | 0 | 0 | 0 | 0 | 0 | 0 | 0 |
|  | F408 | 11 | 9 | 9 | 19 | 14 | 20 | 8 | 0 | 0 | 0 | 0 | 0 | 0 | 0 | 24 | 21 | 18 | 32 | 46 | 35 | 21 | 0 | 0 | 0 | 0 | 0 | 0 | 0 | 35 | 30 | 27 | 51 | 60 | 55 | 29 | 0 | 0 | 0 | 0 | 0 | 0 | 0 |
|  | F409 | 31 | 22 | 18 | 33 | 26 | 33 | 10 | 0 | 0 | 0 | 0 | 0 | 0 | 0 | 51 | 67 | 44 | 52 | 58 | 65 | 26 | 0 | 0 | 0 | 0 | 0 | 0 | 0 | 82 | 89 | 62 | 85 | 84 | 98 | 36 | 0 | 0 | 0 | 0 | 0 | 0 | 0 |
|  | F410 | 60 | 65 | 76 | 123 | 102 | 88 | 38 | 0 | 0 | 0 | 0 | 0 | 0 | 0 | 120 | 112 | 122 | 198 | 164 | 143 | 78 | 0 | 0 | 0 | 0 | 0 | 0 | 0 | 180 | 177 | 198 | 321 | 266 | 231 | 116 | 0 | 0 | 0 | 0 | 0 | 0 | 0 |
|  | F411 | 228 | 246 | 252 | 550 | 535 | 469 | 250 | 0 | 0 | 0 | 0 | 0 | 0 | 0 | 642 | 550 | 502 | 1060 | 1049 | 1016 | 456 | 0 | 0 | 0 | 0 | 0 | 0 | 0 | 870 | 796 | 754 | 1610 | 1584 | 1485 | 706 | 0 | 0 | 0 | 0 | 0 | 0 | 0 |
|  | F412 | 832 | 1201 | 1451 | 2335 | 2066 | 2097 | 1198 | 0 | 0 | 0 | 0 | 0 | 0 | 0 | 2702 | 3655 | 3942 | 6739 | 5575 | 5724 | 2931 | 0 | 0 | 0 | 0 | 0 | 0 | 0 | 3534 | 4856 | 5393 | 9074 | 7641 | 7821 | 4129 | 0 | 0 | 0 | 0 | 0 | 0 | 0 |
|  | F413 | 48 | 71 | 61 | 106 | 142 | 150 | 67 | 0 | 0 | 0 | 0 | 0 | 0 | 0 | 117 | 190 | 129 | 186 | 256 | 216 | 100 | 0 | 0 | 0 | 0 | 0 | 0 | 0 | 165 | 261 | 190 | 292 | 398 | 366 | 167 | 0 | 0 | 0 | 0 | 0 | 0 | 0 |
|  | F418 | 116 | 180 | 173 | 245 | 322 | 259 | 97 | 0 | 0 | 0 | 0 | 0 | 0 | 0 | 272 | 385 | 322 | 574 | 623 | 576 | 202 | 0 | 0 | 0 | 0 | 0 | 0 | 0 | 388 | 565 | 495 | 819 | 945 | 835 | 299 | 0 | 0 | 0 | 0 | 0 | 0 | 0 |
|  | F419 | 481 | 597 | 540 | 1049 | 1241 | 1391 | 598 | 0 | 0 | 0 | 0 | 0 | 0 | 0 | 1270 | 1288 | 1177 | 2230 | 2267 | 2688 | 1144 | 0 | 0 | 0 | 0 | 0 | 0 | 0 | 1751 | 1885 | 1717 | 3279 | 3508 | 4079 | 1742 | 0 | 0 | 0 | 0 | 0 | 0 | 0 |
|  | F420 | 13 | 21 | 10 | 9 | 19 | 21 | 7 | 0 | 0 | 0 | 0 | 0 | 0 | 0 | 14 | 24 | 14 | 21 | 58 | 17 | 11 | 0 | 0 | 0 | 0 | 0 | 0 | 0 | 27 | 45 | 24 | 30 | 77 | 38 | 18 | 0 | 0 | 0 | 0 | 0 | 0 | 0 |
|  | F421 | 3 | 4 | 11 | 8 | 5 | 5 | 3 | 0 | 0 | 0 | 0 | 0 | 0 | 0 | 4 | 13 | 5 | 17 | 5 | 5 | 2 | 0 | 0 | 0 | 0 | 0 | 0 | 0 | 7 | 17 | 16 | 25 | 10 | 10 | 5 | 0 | 0 | 0 | 0 | 0 | 0 | 0 |
|  | F422 | 6 | 9 | 7 | 10 | 9 | 4 | 4 | 0 | 0 | 0 | 0 | 0 | 0 | 0 | 15 | 19 | 16 | 19 | 15 | 16 | 8 | 0 | 0 | 0 | 0 | 0 | 0 | 0 | 21 | 28 | 23 | 29 | 24 | 20 | 12 | 0 | 0 | 0 | 0 | 0 | 0 | 0 |
|  | F428 | 13 | 6 | 17 | 21 | 12 | 21 | 7 | 0 | 0 | 0 | 0 | 0 | 0 | 0 | 6 | 15 | 15 | 25 | 24 | 23 | 7 | 0 | 0 | 0 | 0 | 0 | 0 | 0 | 19 | 21 | 32 | 46 | 36 | 44 | 14 | 0 | 0 | 0 | 0 | 0 | 0 | 0 |
|  | F429 | 22 | 46 | 54 | 89 | 76 | 74 | 38 | 0 | 0 | 0 | 0 | 0 | 0 | 0 | 27 | 25 | 62 | 97 | 91 | 82 | 58 | 0 | 0 | 0 | 0 | 0 | 0 | 0 | 49 | 71 | 116 | 186 | 167 | 156 | 96 | 0 | 0 | 0 | 0 | 0 | 0 | 0 |
|  | F430 | 43 | 42 | 33 | 108 | 154 | 113 | 55 | 0 | 0 | 0 | 0 | 0 | 0 | 0 | 138 | 117 | 103 | 304 | 374 | 327 | 127 | 0 | 0 | 0 | 0 | 0 | 0 | 0 | 181 | 159 | 136 | 412 | 528 | 440 | 182 | 0 | 0 | 0 | 0 | 0 | 0 | 0 |
|  | F431 | 47 | 78 | 120 | 190 | 140 | 175 | 139 | 0 | 0 | 0 | 0 | 0 | 0 | 0 | 77 | 118 | 112 | 186 | 174 | 168 | 101 | 0 | 0 | 0 | 0 | 0 | 0 | 0 | 124 | 196 | 232 | 376 | 314 | 343 | 240 | 0 | 0 | 0 | 0 | 0 | 0 | 0 |
|  | F432 | 168 | 270 | 325 | 639 | 1061 | 992 | 330 | 0 | 0 | 0 | 0 | 0 | 0 | 0 | 1707 | 1678 | 1708 | 2530 | 3329 | 2818 | 830 | 0 | 0 | 0 | 0 | 0 | 0 | 0 | 1875 | 1948 | 2033 | 3169 | 4390 | 3810 | 1160 | 0 | 0 | 0 | 0 | 0 | 0 | 0 |
|  | F438 | 6 | 6 | 0 | 37 | 67 | 57 | 10 | 0 | 0 | 0 | 0 | 0 | 0 | 0 | 16 | 11 | 27 | 116 | 308 | 251 | 60 | 0 | 0 | 0 | 0 | 0 | 0 | 0 | 22 | 17 | 27 | 153 | 375 | 308 | 70 | 0 | 0 | 0 | 0 | 0 | 0 | 0 |
|  | F439 | 1 | 3 | 4 | 19 | 39 | 26 | 12 | 0 | 0 | 0 | 0 | 0 | 0 | 0 | 9 | 13 | 18 | 62 | 175 | 70 | 27 | 0 | 0 | 0 | 0 | 0 | 0 | 0 | 10 | 16 | 22 | 81 | 214 | 96 | 39 | 0 | 0 | 0 | 0 | 0 | 0 | 0 |
| Norte de Sant | F400 | 1 | 0 | 2 | 2 | 4 | 4 | 6 | 0 | 0 | 0 | 0 | 0 | 0 | 0 | 0 | 2 | 1 | 3 | 3 | 2 | 4 | 0 | 0 | 0 | 0 | 0 | 0 | 0 | 1 | 2 | 3 | 5 | 7 | 6 | 10 | 0 | 0 | 0 | 0 | 0 | 0 | 0 |
|  | F401 | 2 | 4 | 2 | 3 | 4 | 10 | 3 | 0 | 0 | 0 | 0 | 0 | 0 | 0 | 1 | 2 | 1 | 4 | 0 | 5 | 4 | 0 | 0 | 0 | 0 | 0 | 0 | 0 | 3 | 6 | 3 | 7 | 4 | 15 | 7 | 0 | 0 | 0 | 0 | 0 | 0 | 0 |
|  | F402 | 3 | 5 | 3 | 3 | 6 | 7 | 10 | 0 | 0 | 0 | 0 | 0 | 0 | 0 | 3 | 2 | 4 | 3 | 6 | 7 | 8 | 0 | 0 | 0 | 0 | 0 | 0 | 0 | 6 | 7 | 7 | 6 | 12 | 14 | 18 | 0 | 0 | 0 | 0 | 0 | 0 | 0 |
|  | F408 | 7 | 12 | 14 | 9 | 15 | 24 | 11 | 0 | 0 | 0 | 0 | 0 | 0 | 0 | 17 | 26 | 28 | 32 | 45 | 35 | 42 | 0 | 0 | 0 | 0 | 0 | 0 | 0 | 24 | 38 | 42 | 41 | 60 | 59 | 53 | 0 | 0 | 0 | 0 | 0 | 0 | 0 |
|  | F409 | 13 | 18 | 13 | 24 | 24 | 34 | 35 | 0 | 0 | 0 | 0 | 0 | 0 | 0 | 17 | 34 | 31 | 48 | 52 | 50 | 71 | 0 | 0 | 0 | 0 | 0 | 0 | 0 | 30 | 52 | 44 | 72 | 76 | 84 | 106 | 0 | 0 | 0 | 0 | 0 | 0 | 0 |
|  | F410 | 16 | 33 | 33 | 69 | 78 | 100 | 100 | 0 | 0 | 0 | 0 | 0 | 0 | 0 | 37 | 71 | 80 | 105 | 137 | 184 | 149 | 0 | 0 | 0 | 0 | 0 | 0 | 0 | 53 | 104 | 113 | 174 | 215 | 284 | 249 | 0 | 0 | 0 | 0 | 0 | 0 | 0 |
|  | F411 | 83 | 114 | 165 | 437 | 713 | 808 | 612 | 0 | 0 | 0 | 0 | 0 | 0 | 0 | 268 | 270 | 398 | 725 | 1648 | 1624 | 1158 | 0 | 0 | 0 | 0 | 0 | 0 | 0 | 351 | 384 | 563 | 1162 | 2361 | 2432 | 1770 | 0 | 0 | 0 | 0 | 0 | 0 | 0 |
|  | F412 | 353 | 561 | 765 | 1190 | 1324 | 1864 | 1865 | 0 | 0 | 0 | 0 | 0 | 0 | 0 | 867 | 1430 | 1802 | 3005 | 3175 | 4528 | 4806 | 0 | 0 | 0 | 0 | 0 | 0 | 0 | 1220 | 1991 | 2567 | 4195 | 4499 | 6392 | 6671 | 0 | 0 | 0 | 0 | 0 | 0 | 0 |
|  | F413 | 42 | 41 | 75 | 98 | 132 | 191 | 138 | 0 | 0 | 0 | 0 | 0 | 0 | 0 | 66 | 115 | 172 | 181 | 302 | 427 | 247 | 0 | 0 | 0 | 0 | 0 | 0 | 0 | 108 | 156 | 247 | 279 | 434 | 618 | 385 | 0 | 0 | 0 | 0 | 0 | 0 | 0 |
|  | F418 | 78 | 153 | 216 | 299 | 398 | 426 | 209 | 0 | 0 | 0 | 0 | 0 | 0 | 0 | 129 | 281 | 369 | 518 | 670 | 750 | 352 | 0 | 0 | 0 | 0 | 0 | 0 | 0 | 207 | 434 | 585 | 817 | 1068 | 1176 | 561 | 0 | 0 | 0 | 0 | 0 | 0 | 0 |
|  | F419 | 244 | 472 | 656 | 890 | 947 | 1298 | 1441 | 0 | 0 | 0 | 0 | 0 | 0 | 0 | 580 | 1072 | 1276 | 1721 | 1801 | 2506 | 2921 | 0 | 0 | 0 | 0 | 2 | 1 | 0 | 824 | 1544 | 1932 | 2611 | 2748 | 3804 | 4362 | 0 | 0 | 0 | 0 | 2 | 1 | 0 |
|  | F420 | 1 | 4 | 3 | 2 | 7 | 9 | 16 | 0 | 0 | 0 | 0 | 0 | 0 | 0 | 4 | 6 | 2 | 6 | 5 | 11 | 8 | 0 | 0 | 0 | 0 | 0 | 0 | 0 | 5 | 10 | 5 | 8 | 12 | 20 | 24 | 0 | 0 | 0 | 0 | 0 | 0 | 0 |
|  | F421 | 0 | 2 | 0 | 1 | 9 | 5 | 4 | 0 | 0 | 0 | 0 | 0 | 0 | 0 | 2 | 2 | 3 | 0 | 6 | 3 | 4 | 0 | 0 | 0 | 0 | 0 | 0 | 0 | 2 | 4 | 3 | 1 | 15 | 8 | 8 | 0 | 0 | 0 | 0 | 0 | 0 | 0 |
|  | F422 | 3 | 4 | 6 | 4 | 11 | 5 | 8 | 0 | 0 | 0 | 0 | 0 | 0 | 0 | 0 | 2 | 3 | 10 | 8 | 4 | 8 | 0 | 0 | 0 | 0 | 0 | 0 | 0 | 3 | 6 | 9 | 14 | 19 | 9 | 16 | 0 | 0 | 0 | 0 | 0 | 0 | 0 |
|  | F428 | 0 | 1 | 6 | 14 | 16 | 19 | 28 | 0 | 0 | 0 | 0 | 0 | 0 | 0 | 2 | 3 | 5 | 16 | 13 | 21 | 23 | 0 | 0 | 0 | 0 | 0 | 0 | 0 | 2 | 4 | 11 | 30 | 29 | 40 | 51 | 0 | 0 | 0 | 0 | 0 | 0 | 0 |
|  | F429 | 3 | 10 | 10 | 20 | 19 | 38 | 38 | 0 | 0 | 0 | 0 | 0 | 0 | 0 | 5 | 7 | 9 | 16 | 21 | 28 | 28 | 0 | 0 | 0 | 0 | 0 | 0 | 0 | 8 | 17 | 19 | 36 | 40 | 66 | 66 | 0 | 0 | 0 | 0 | 0 | 0 | 0 |
|  | F430 | 18 | 23 | 24 | 76 | 64 | 91 | 75 | 0 | 0 | 0 | 0 | 0 | 0 | 0 | 43 | 41 | 45 | 113 | 151 | 236 | 148 | 0 | 0 | 0 | 0 | 0 | 0 | 0 | 61 | 64 | 69 | 189 | 215 | 327 | 223 | 0 | 0 | 0 | 0 | 0 | 0 | 0 |
|  | F431 | 75 | 34 | 52 | 89 | 70 | 115 | 133 | 0 | 0 | 0 | 0 | 0 | 0 | 0 | 66 | 30 | 46 | 74 | 65 | 95 | 106 | 0 | 0 | 0 | 0 | 0 | 0 | 0 | 141 | 64 | 98 | 163 | 135 | 210 | 239 | 0 | 0 | 0 | 0 | 0 | 0 | 0 |
|  | F432 | 63 | 151 | 296 | 670 | 588 | 694 | 569 | 0 | 0 | 0 | 0 | 0 | 0 | 0 | 90 | 172 | 319 | 922 | 843 | 1038 | 863 | 0 | 0 | 0 | 0 | 0 | 0 | 0 | 153 | 323 | 615 | 1592 | 1431 | 1732 | 1432 | 0 | 0 | 0 | 0 | 0 | 0 | 0 |
|  | F438 | 0 | 6 | 4 | 6 | 6 | 11 | 19 | 0 | 0 | 0 | 0 | 0 | 0 | 0 | 5 | 9 | 2 | 12 | 12 | 4 | 8 | 0 | 0 | 0 | 0 | 0 | 0 | 0 | 5 | 15 | 6 | 18 | 18 | 15 | 27 | 0 | 0 | 0 | 0 | 0 | 0 | 0 |
|  | F439 | 2 | 3 | 1 | 5 | 6 | 20 | 26 | 0 | 0 | 0 | 0 | 0 | 0 | 0 | 3 | 7 | 13 | 11 | 16 | 30 | 24 | 0 | 0 | 0 | 0 | 0 | 0 | 0 | 5 | 10 | 14 | 16 | 22 | 50 | 50 | 0 | 0 | 0 | 0 | 0 | 0 | 0 |
| Putumayo | F400 | 1 | 1 | 0 | 1 | 0 | 0 | 0 | 0 | 0 | 0 | 0 | 0 | 0 | 0 | 0 | 0 | 0 | 0 | 0 | 0 | 2 | 0 | 0 | 0 | 0 | 0 | 0 | 0 | 1 | 1 | 0 | 1 | 0 | 0 | 2 | 0 | 0 | 0 | 0 | 0 | 0 | 0 |
|  | F401 | 1 | 1 | 1 | 3 | 1 | 0 | 1 | 0 | 0 | 0 | 0 | 0 | 0 | 0 | 1 | 0 | 0 | 4 | 1 | 1 | 2 | 0 | 0 | 0 | 0 | 0 | 0 | 0 | 2 | 1 | 1 | 7 | 2 | 1 | 3 | 0 | 0 | 0 | 0 | 0 | 0 | 0 |
|  | F402 | 0 | 0 | 0 | 2 | 2 | 0 | 0 | 0 | 0 | 0 | 0 | 0 | 0 | 0 | 1 | 2 | 3 | 2 | 0 | 1 | 3 | 0 | 0 | 0 | 0 | 0 | 0 | 0 | 1 | 2 | 3 | 4 | 2 | 1 | 3 | 0 | 0 | 0 | 0 | 0 | 0 | 0 |
|  | F408 | 1 | 1 | 2 | 1 | 6 | 5 | 1 | 0 | 0 | 0 | 0 | 0 | 0 | 0 | 5 | 2 | 5 | 3 | 5 | 4 | 7 | 0 | 0 | 0 | 0 | 0 | 0 | 0 | 6 | 3 | 7 | 4 | 11 | 9 | 8 | 0 | 0 | 0 | 0 | 0 | 0 | 0 |
|  | F409 | 2 | 1 | 1 | 4 | 4 | 2 | 2 | 0 | 0 | 0 | 0 | 0 | 0 | 0 | 3 | 3 | 6 | 4 | 4 | 1 | 4 | 0 | 0 | 0 | 0 | 0 | 0 | 0 | 5 | 4 | 7 | 8 | 8 | 3 | 6 | 0 | 0 | 0 | 0 | 0 | 0 | 0 |
|  | F410 | 4 | 10 | 12 | 31 | 25 | 37 | 12 | 0 | 0 | 0 | 0 | 0 | 0 | 0 | 13 | 16 | 12 | 47 | 44 | 61 | 23 | 0 | 0 | 0 | 0 | 0 | 0 | 0 | 17 | 26 | 24 | 78 | 69 | 98 | 35 | 0 | 0 | 0 | 0 | 0 | 0 | 0 |
|  | F411 | 17 | 26 | 30 | 64 | 115 | 129 | 109 | 0 | 0 | 0 | 0 | 0 | 0 | 0 | 47 | 66 | 45 | 170 | 182 | 204 | 164 | 0 | 0 | 0 | 0 | 0 | 0 | 0 | 64 | 92 | 75 | 234 | 297 | 333 | 273 | 0 | 0 | 0 | 0 | 0 | 0 | 0 |
|  | F412 | 60 | 97 | 127 | 234 | 201 | 240 | 167 | 0 | 0 | 0 | 0 | 0 | 0 | 0 | 199 | 322 | 319 | 599 | 481 | 570 | 384 | 0 | 0 | 0 | 0 | 0 | 0 | 0 | 259 | 419 | 446 | 833 | 682 | 810 | 551 | 0 | 0 | 0 | 0 | 0 | 0 | 0 |
|  | F413 | 8 | 26 | 14 | 24 | 42 | 31 | 24 | 0 | 0 | 0 | 0 | 0 | 0 | 0 | 17 | 38 | 21 | 49 | 59 | 59 | 33 | 0 | 0 | 0 | 0 | 0 | 0 | 0 | 25 | 64 | 35 | 73 | 101 | 90 | 57 | 0 | 0 | 0 | 0 | 0 | 0 | 0 |
|  | F418 | 21 | 26 | 24 | 41 | 47 | 45 | 21 | 0 | 0 | 0 | 0 | 0 | 0 | 0 | 34 | 54 | 41 | 56 | 57 | 89 | 54 | 0 | 0 | 0 | 0 | 0 | 0 | 0 | 55 | 80 | 65 | 97 | 104 | 134 | 75 | 0 | 0 | 0 | 0 | 0 | 0 | 0 |
|  | F419 | 54 | 61 | 58 | 145 | 204 | 170 | 99 | 0 | 0 | 0 | 0 | 0 | 0 | 0 | 106 | 135 | 103 | 237 | 302 | 320 | 197 | 0 | 0 | 0 | 0 | 0 | 0 | 0 | 160 | 196 | 161 | 382 | 506 | 490 | 296 | 0 | 0 | 0 | 0 | 0 | 0 | 0 |
|  | F420 | 2 | 1 | 1 | 1 | 1 | 4 | 0 | 0 | 0 | 0 | 0 | 0 | 0 | 0 | 2 | 2 | 5 | 4 | 1 | 2 | 1 | 0 | 0 | 0 | 0 | 0 | 0 | 0 | 4 | 3 | 6 | 5 | 2 | 6 | 1 | 0 | 0 | 0 | 0 | 0 | 0 | 0 |
|  | F421 | 0 | 2 | 0 | 0 | 0 | 0 | 0 | 0 | 0 | 0 | 0 | 0 | 0 | 0 | 0 | 3 | 2 | 1 | 2 | 3 | 1 | 0 | 0 | 0 | 0 | 0 | 0 | 0 | 0 | 5 | 2 | 1 | 2 | 3 | 1 | 0 | 0 | 0 | 0 | 0 | 0 | 0 |
|  | F422 | 1 | 0 | 0 | 3 | 1 | 2 | 2 | 0 | 0 | 0 | 0 | 0 | 0 | 0 | 0 | 0 | 2 | 3 | 3 | 2 | 0 | 0 | 0 | 0 | 0 | 0 | 0 | 0 | 1 | 0 | 2 | 6 | 4 | 4 | 2 | 0 | 0 | 0 | 0 | 0 | 0 | 0 |
|  | F428 | 2 | 1 | 1 | 3 | 2 | 1 | 1 | 0 | 0 | 0 | 0 | 0 | 0 | 0 | 3 | 2 | 0 | 2 | 2 | 4 | 2 | 0 | 0 | 0 | 0 | 0 | 0 | 0 | 5 | 3 | 1 | 5 | 4 | 5 | 3 | 0 | 0 | 0 | 0 | 0 | 0 | 0 |
|  | F429 | 6 | 4 | 7 | 8 | 4 | 6 | 3 | 0 | 0 | 0 | 0 | 0 | 0 | 0 | 10 | 11 | 11 | 16 | 8 | 16 | 6 | 0 | 0 | 0 | 0 | 0 | 0 | 0 | 16 | 15 | 18 | 24 | 12 | 22 | 9 | 0 | 0 | 0 | 0 | 0 | 0 | 0 |
|  | F430 | 3 | 6 | 4 | 14 | 21 | 24 | 11 | 0 | 0 | 0 | 0 | 0 | 0 | 0 | 3 | 5 | 7 | 40 | 57 | 75 | 35 | 0 | 0 | 0 | 0 | 0 | 0 | 0 | 6 | 11 | 11 | 54 | 78 | 99 | 46 | 0 | 0 | 0 | 0 | 0 | 0 | 0 |
|  | F431 | 6 | 9 | 19 | 32 | 30 | 24 | 11 | 0 | 0 | 0 | 0 | 0 | 0 | 0 | 15 | 21 | 26 | 58 | 23 | 29 | 19 | 0 | 0 | 0 | 0 | 0 | 0 | 0 | 21 | 30 | 45 | 90 | 53 | 53 | 30 | 0 | 0 | 0 | 0 | 0 | 0 | 0 |
|  | F432 | 10 | 13 | 19 | 39 | 39 | 37 | 13 | 0 | 0 | 0 | 0 | 0 | 0 | 0 | 20 | 28 | 25 | 74 | 57 | 60 | 33 | 0 | 0 | 0 | 0 | 0 | 0 | 0 | 30 | 41 | 44 | 113 | 96 | 97 | 46 | 0 | 0 | 0 | 0 | 0 | 0 | 0 |
|  | F438 | 0 | 1 | 2 | 3 | 1 | 3 | 4 | 0 | 0 | 0 | 0 | 0 | 0 | 0 | 3 | 1 | 7 | 12 | 13 | 5 | 8 | 0 | 0 | 0 | 0 | 0 | 0 | 0 | 3 | 2 | 9 | 15 | 14 | 8 | 12 | 0 | 0 | 0 | 0 | 0 | 0 | 0 |
|  | F439 | 0 | 0 | 0 | 0 | 1 | 2 | 5 | 0 | 0 | 0 | 0 | 0 | 0 | 0 | 1 | 2 | 1 | 8 | 11 | 5 | 2 | 0 | 0 | 0 | 0 | 0 | 0 | 0 | 1 | 2 | 1 | 8 | 12 | 7 | 7 | 0 | 0 | 0 | 0 | 0 | 0 | 0 |
| Quindio | F400 | 1 | 2 | 0 | 0 | 1 | 0 | 0 | 0 | 0 | 0 | 0 | 0 | 0 | 0 | 1 | 5 | 1 | 1 | 2 | 2 | 3 | 0 | 0 | 0 | 0 | 0 | 0 | 0 | 2 | 7 | 1 | 1 | 3 | 2 | 3 | 0 | 0 | 0 | 0 | 0 | 0 | 0 |
|  | F401 | 5 | 5 | 6 | 11 | 5 | 7 | 5 | 0 | 0 | 0 | 0 | 0 | 0 | 0 | 0 | 3 | 2 | 2 | 5 | 8 | 12 | 0 | 0 | 0 | 0 | 0 | 0 | 0 | 5 | 8 | 8 | 13 | 10 | 15 | 17 | 0 | 0 | 0 | 0 | 0 | 0 | 0 |
|  | F402 | 0 | 5 | 4 | 5 | 3 | 5 | 7 | 0 | 0 | 0 | 0 | 0 | 0 | 0 | 6 | 8 | 6 | 15 | 6 | 12 | 22 | 0 | 0 | 0 | 0 | 0 | 0 | 0 | 6 | 13 | 10 | 20 | 9 | 17 | 29 | 0 | 0 | 0 | 0 | 0 | 0 | 0 |
|  | F408 | 3 | 6 | 6 | 4 | 9 | 8 | 5 | 0 | 0 | 0 | 0 | 0 | 0 | 0 | 6 | 5 | 8 | 9 | 17 | 10 | 15 | 0 | 0 | 0 | 0 | 0 | 0 | 0 | 9 | 11 | 14 | 13 | 26 | 18 | 20 | 0 | 0 | 0 | 0 | 0 | 0 | 0 |
|  | F409 | 10 | 2 | 3 | 6 | 9 | 9 | 10 | 0 | 0 | 0 | 0 | 0 | 0 | 0 | 6 | 5 | 8 | 14 | 14 | 16 | 19 | 0 | 0 | 0 | 0 | 0 | 0 | 0 | 16 | 7 | 11 | 20 | 23 | 25 | 29 | 0 | 0 | 0 | 0 | 0 | 0 | 0 |
|  | F410 | 24 | 51 | 52 | 89 | 53 | 100 | 73 | 0 | 0 | 0 | 0 | 0 | 0 | 0 | 46 | 86 | 94 | 166 | 131 | 211 | 177 | 0 | 0 | 0 | 0 | 0 | 0 | 0 | 70 | 137 | 146 | 255 | 184 | 311 | 250 | 0 | 0 | 0 | 0 | 0 | 0 | 0 |
|  | F411 | 71 | 105 | 177 | 331 | 371 | 523 | 582 | 0 | 0 | 0 | 0 | 0 | 0 | 0 | 180 | 235 | 372 | 612 | 755 | 1165 | 1314 | 0 | 0 | 0 | 0 | 0 | 0 | 0 | 251 | 340 | 549 | 943 | 1126 | 1688 | 1896 | 0 | 0 | 0 | 0 | 0 | 0 | 0 |
|  | F412 | 209 | 309 | 425 | 773 | 1075 | 1333 | 1106 | 0 | 0 | 0 | 0 | 0 | 0 | 0 | 804 | 1097 | 1331 | 2509 | 2848 | 3541 | 3259 | 0 | 0 | 0 | 0 | 0 | 0 | 0 | 1013 | 1406 | 1756 | 3282 | 3923 | 4874 | 4365 | 0 | 0 | 0 | 0 | 0 | 0 | 0 |
|  | F413 | 29 | 40 | 73 | 77 | 89 | 99 | 86 | 0 | 0 | 0 | 0 | 0 | 0 | 0 | 60 | 106 | 152 | 191 | 184 | 199 | 163 | 0 | 0 | 0 | 0 | 0 | 0 | 0 | 89 | 146 | 225 | 268 | 273 | 298 | 249 | 0 | 0 | 0 | 0 | 0 | 0 | 0 |
|  | F418 | 89 | 103 | 97 | 177 | 279 | 228 | 221 | 0 | 0 | 0 | 0 | 0 | 0 | 0 | 145 | 205 | 166 | 377 | 463 | 412 | 365 | 0 | 0 | 0 | 0 | 0 | 0 | 0 | 234 | 308 | 263 | 554 | 742 | 640 | 586 | 0 | 0 | 0 | 0 | 0 | 0 | 0 |
|  | F419 | 186 | 259 | 344 | 554 | 561 | 757 | 629 | 0 | 0 | 0 | 0 | 0 | 0 | 0 | 449 | 624 | 735 | 1246 | 1135 | 1450 | 1254 | 0 | 0 | 0 | 0 | 0 | 0 | 0 | 635 | 883 | 1079 | 1800 | 1696 | 2207 | 1883 | 0 | 0 | 0 | 0 | 0 | 0 | 0 |
|  | F420 | 2 | 5 | 4 | 8 | 6 | 10 | 15 | 0 | 0 | 0 | 0 | 0 | 0 | 0 | 2 | 12 | 5 | 7 | 6 | 7 | 14 | 0 | 0 | 0 | 0 | 0 | 0 | 0 | 4 | 17 | 9 | 15 | 12 | 17 | 29 | 0 | 0 | 0 | 0 | 0 | 0 | 0 |
|  | F421 | 2 | 0 | 0 | 1 | 5 | 4 | 2 | 0 | 0 | 0 | 0 | 0 | 0 | 0 | 2 | 2 | 0 | 2 | 6 | 7 | 8 | 0 | 0 | 0 | 0 | 0 | 0 | 0 | 4 | 2 | 0 | 3 | 11 | 11 | 10 | 0 | 0 | 0 | 0 | 0 | 0 | 0 |
|  | F422 | 3 | 5 | 3 | 9 | 2 | 8 | 6 | 0 | 0 | 0 | 0 | 0 | 0 | 0 | 3 | 2 | 0 | 5 | 5 | 3 | 7 | 0 | 0 | 0 | 0 | 0 | 0 | 0 | 6 | 7 | 3 | 14 | 7 | 11 | 13 | 0 | 0 | 0 | 0 | 0 | 0 | 0 |
|  | F428 | 3 | 8 | 7 | 16 | 15 | 12 | 8 | 0 | 0 | 0 | 0 | 0 | 0 | 0 | 2 | 3 | 8 | 17 | 12 | 11 | 7 | 0 | 0 | 0 | 0 | 0 | 0 | 0 | 5 | 11 | 15 | 33 | 27 | 23 | 15 | 0 | 0 | 0 | 0 | 0 | 0 | 0 |
|  | F429 | 6 | 7 | 14 | 29 | 26 | 20 | 26 | 0 | 0 | 0 | 0 | 0 | 0 | 0 | 3 | 7 | 13 | 31 | 14 | 24 | 25 | 0 | 0 | 0 | 0 | 0 | 0 | 0 | 9 | 14 | 27 | 60 | 40 | 44 | 51 | 0 | 0 | 0 | 0 | 0 | 0 | 0 |
|  | F430 | 8 | 8 | 6 | 18 | 13 | 32 | 31 | 0 | 0 | 0 | 0 | 0 | 0 | 0 | 23 | 24 | 29 | 31 | 26 | 73 | 65 | 0 | 0 | 0 | 0 | 0 | 0 | 0 | 31 | 32 | 35 | 49 | 39 | 105 | 96 | 0 | 0 | 0 | 0 | 0 | 0 | 0 |
|  | F431 | 37 | 40 | 74 | 97 | 81 | 83 | 112 | 0 | 0 | 0 | 0 | 0 | 0 | 0 | 34 | 34 | 32 | 59 | 46 | 52 | 121 | 0 | 0 | 0 | 0 | 0 | 0 | 0 | 71 | 74 | 106 | 156 | 127 | 135 | 233 | 0 | 0 | 0 | 0 | 0 | 0 | 0 |
|  | F432 | 65 | 116 | 142 | 246 | 192 | 268 | 303 | 0 | 0 | 0 | 0 | 0 | 0 | 0 | 130 | 217 | 273 | 501 | 307 | 416 | 548 | 0 | 0 | 0 | 0 | 0 | 0 | 0 | 195 | 333 | 415 | 747 | 499 | 684 | 851 | 0 | 0 | 0 | 0 | 0 | 0 | 0 |
|  | F438 | 3 | 0 | 3 | 1 | 0 | 2 | 2 | 0 | 0 | 0 | 0 | 0 | 0 | 0 | 4 | 0 | 4 | 1 | 5 | 5 | 16 | 0 | 0 | 0 | 0 | 0 | 0 | 0 | 7 | 0 | 7 | 2 | 5 | 7 | 18 | 0 | 0 | 0 | 0 | 0 | 0 | 0 |
|  | F439 | 0 | 2 | 1 | 3 | 0 | 1 | 3 | 0 | 0 | 0 | 0 | 0 | 0 | 0 | 0 | 2 | 2 | 7 | 1 | 2 | 8 | 0 | 0 | 0 | 0 | 0 | 0 | 0 | 0 | 4 | 3 | 10 | 1 | 3 | 11 | 0 | 0 | 0 | 0 | 0 | 0 | 0 |
| Risaralda | F400 | 5 | 3 | 5 | 5 | 2 | 1 | 1 | 0 | 0 | 0 | 0 | 0 | 0 | 0 | 4 | 3 | 5 | 11 | 9 | 12 | 12 | 0 | 0 | 0 | 0 | 0 | 0 | 0 | 9 | 6 | 10 | 16 | 11 | 13 | 13 | 0 | 0 | 0 | 0 | 0 | 0 | 0 |
|  | F401 | 4 | 3 | 11 | 17 | 23 | 10 | 23 | 0 | 0 | 0 | 0 | 0 | 0 | 0 | 7 | 6 | 7 | 19 | 12 | 20 | 21 | 0 | 0 | 0 | 0 | 0 | 0 | 0 | 11 | 9 | 18 | 36 | 35 | 30 | 44 | 0 | 0 | 0 | 0 | 0 | 0 | 0 |
|  | F402 | 6 | 4 | 5 | 19 | 12 | 8 | 6 | 0 | 0 | 0 | 0 | 0 | 0 | 0 | 6 | 2 | 4 | 26 | 19 | 20 | 29 | 0 | 0 | 0 | 0 | 0 | 0 | 0 | 12 | 6 | 9 | 45 | 31 | 28 | 35 | 0 | 0 | 0 | 0 | 0 | 0 | 0 |
|  | F408 | 7 | 7 | 10 | 16 | 11 | 11 | 11 | 0 | 0 | 0 | 0 | 0 | 0 | 0 | 10 | 3 | 10 | 35 | 36 | 28 | 31 | 0 | 0 | 0 | 0 | 0 | 0 | 0 | 17 | 10 | 20 | 51 | 47 | 39 | 42 | 0 | 0 | 0 | 0 | 0 | 0 | 0 |
|  | F409 | 13 | 20 | 16 | 23 | 26 | 16 | 12 | 0 | 0 | 0 | 0 | 0 | 0 | 0 | 34 | 34 | 19 | 40 | 41 | 47 | 48 | 0 | 0 | 0 | 0 | 0 | 0 | 0 | 47 | 54 | 35 | 63 | 67 | 63 | 60 | 0 | 0 | 0 | 0 | 0 | 0 | 0 |
|  | F410 | 115 | 147 | 191 | 214 | 219 | 251 | 230 | 0 | 0 | 0 | 0 | 0 | 0 | 0 | 217 | 273 | 396 | 509 | 526 | 580 | 540 | 0 | 0 | 0 | 0 | 0 | 0 | 0 | 332 | 420 | 587 | 723 | 745 | 831 | 770 | 0 | 0 | 0 | 0 | 0 | 0 | 0 |
|  | F411 | 168 | 206 | 287 | 402 | 581 | 631 | 455 | 0 | 0 | 0 | 0 | 0 | 0 | 0 | 484 | 525 | 775 | 1117 | 1493 | 1836 | 1359 | 0 | 0 | 0 | 0 | 0 | 0 | 0 | 652 | 731 | 1062 | 1519 | 2074 | 2467 | 1814 | 0 | 0 | 0 | 0 | 0 | 0 | 0 |
|  | F412 | 600 | 718 | 855 | 1398 | 1763 | 2245 | 2686 | 0 | 0 | 0 | 0 | 0 | 0 | 0 | 2070 | 2502 | 2961 | 4281 | 5013 | 6288 | 7671 | 0 | 0 | 0 | 0 | 0 | 0 | 0 | 2670 | 3220 | 3816 | 5679 | 6776 | 8533 | 10357 | 0 | 0 | 0 | 0 | 0 | 0 | 0 |
|  | F413 | 75 | 77 | 97 | 193 | 201 | 217 | 165 | 0 | 0 | 0 | 0 | 0 | 0 | 0 | 211 | 221 | 213 | 416 | 508 | 533 | 410 | 0 | 0 | 0 | 0 | 0 | 0 | 0 | 286 | 298 | 310 | 609 | 709 | 750 | 575 | 0 | 0 | 0 | 0 | 0 | 0 | 0 |
|  | F418 | 90 | 90 | 128 | 328 | 445 | 509 | 324 | 0 | 0 | 0 | 0 | 0 | 0 | 0 | 216 | 226 | 280 | 727 | 908 | 983 | 684 | 0 | 0 | 0 | 0 | 0 | 0 | 0 | 306 | 316 | 408 | 1055 | 1353 | 1492 | 1008 | 0 | 0 | 0 | 0 | 0 | 0 | 0 |
|  | F419 | 554 | 639 | 898 | 1458 | 1835 | 2091 | 1722 | 0 | 0 | 0 | 0 | 0 | 0 | 0 | 1489 | 1536 | 2338 | 3447 | 3831 | 4514 | 3897 | 0 | 0 | 0 | 0 | 0 | 0 | 0 | 2043 | 2175 | 3236 | 4905 | 5666 | 6605 | 5619 | 0 | 0 | 0 | 0 | 0 | 0 | 0 |
|  | F420 | 9 | 8 | 12 | 14 | 12 | 10 | 23 | 0 | 0 | 0 | 0 | 0 | 0 | 0 | 2 | 7 | 14 | 7 | 11 | 16 | 13 | 0 | 0 | 0 | 0 | 0 | 0 | 0 | 11 | 15 | 26 | 21 | 23 | 26 | 36 | 0 | 0 | 0 | 0 | 0 | 0 | 0 |
|  | F421 | 3 | 1 | 5 | 3 | 4 | 6 | 5 | 0 | 0 | 0 | 0 | 0 | 0 | 0 | 3 | 6 | 4 | 9 | 6 | 13 | 7 | 0 | 0 | 0 | 0 | 0 | 0 | 0 | 6 | 7 | 9 | 12 | 10 | 19 | 12 | 0 | 0 | 0 | 0 | 0 | 0 | 0 |
|  | F422 | 1 | 1 | 3 | 5 | 14 | 37 | 10 | 0 | 0 | 0 | 0 | 0 | 0 | 0 | 0 | 2 | 6 | 1 | 55 | 92 | 11 | 0 | 0 | 0 | 0 | 0 | 0 | 0 | 1 | 3 | 9 | 6 | 69 | 129 | 21 | 0 | 0 | 0 | 0 | 0 | 0 | 0 |
|  | F428 | 6 | 13 | 18 | 20 | 23 | 29 | 22 | 0 | 0 | 0 | 0 | 0 | 0 | 0 | 9 | 14 | 27 | 32 | 30 | 31 | 32 | 0 | 0 | 0 | 0 | 0 | 0 | 0 | 15 | 27 | 45 | 52 | 53 | 60 | 54 | 0 | 0 | 0 | 0 | 0 | 0 | 0 |
|  | F429 | 15 | 36 | 63 | 80 | 72 | 81 | 82 | 0 | 0 | 0 | 0 | 0 | 0 | 0 | 23 | 22 | 69 | 76 | 78 | 79 | 87 | 0 | 0 | 0 | 0 | 0 | 0 | 0 | 38 | 58 | 132 | 156 | 150 | 160 | 169 | 0 | 0 | 0 | 0 | 0 | 0 | 0 |
|  | F430 | 18 | 18 | 29 | 27 | 41 | 29 | 49 | 0 | 0 | 0 | 0 | 0 | 0 | 0 | 49 | 63 | 55 | 86 | 54 | 93 | 96 | 0 | 0 | 0 | 0 | 0 | 0 | 0 | 67 | 81 | 84 | 113 | 95 | 122 | 145 | 0 | 0 | 0 | 0 | 0 | 0 | 0 |
|  | F431 | 44 | 46 | 93 | 104 | 96 | 102 | 94 | 0 | 0 | 0 | 0 | 0 | 0 | 0 | 67 | 68 | 94 | 122 | 90 | 121 | 140 | 0 | 0 | 0 | 0 | 0 | 0 | 0 | 111 | 114 | 187 | 226 | 186 | 223 | 234 | 0 | 0 | 0 | 0 | 0 | 0 | 0 |
|  | F432 | 206 | 342 | 632 | 1215 | 1050 | 887 | 1136 | 0 | 0 | 0 | 0 | 0 | 0 | 0 | 442 | 680 | 1033 | 1884 | 1531 | 1533 | 1895 | 0 | 0 | 0 | 1 | 0 | 0 | 0 | 648 | 1022 | 1665 | 3099 | 2581 | 2420 | 3031 | 0 | 0 | 0 | 1 | 0 | 0 | 0 |
|  | F438 | 6 | 1 | 3 | 6 | 7 | 7 | 11 | 0 | 0 | 0 | 0 | 0 | 0 | 0 | 11 | 11 | 6 | 4 | 12 | 21 | 18 | 0 | 0 | 0 | 0 | 0 | 0 | 0 | 17 | 12 | 9 | 10 | 19 | 28 | 29 | 0 | 0 | 0 | 0 | 0 | 0 | 0 |
|  | F439 | 8 | 7 | 9 | 6 | 4 | 4 | 6 | 0 | 0 | 0 | 0 | 0 | 0 | 0 | 19 | 10 | 16 | 12 | 5 | 11 | 14 | 0 | 0 | 0 | 0 | 0 | 0 | 0 | 27 | 17 | 25 | 18 | 9 | 15 | 20 | 0 | 0 | 0 | 0 | 0 | 0 | 0 |
| Santander | F400 | 0 | 3 | 7 | 7 | 8 | 10 | 11 | 0 | 0 | 0 | 0 | 0 | 0 | 0 | 11 | 13 | 10 | 19 | 10 | 4 | 20 | 0 | 0 | 0 | 0 | 0 | 0 | 0 | 11 | 16 | 17 | 26 | 18 | 14 | 31 | 0 | 0 | 0 | 0 | 0 | 0 | 0 |
|  | F401 | 7 | 13 | 19 | 19 | 17 | 19 | 38 | 0 | 0 | 0 | 0 | 0 | 0 | 0 | 6 | 5 | 15 | 21 | 18 | 24 | 42 | 0 | 0 | 0 | 0 | 0 | 0 | 0 | 13 | 18 | 34 | 40 | 35 | 43 | 80 | 0 | 0 | 0 | 0 | 0 | 0 | 0 |
|  | F402 | 10 | 13 | 16 | 15 | 14 | 21 | 35 | 0 | 0 | 0 | 0 | 0 | 0 | 0 | 11 | 18 | 25 | 34 | 21 | 25 | 44 | 0 | 0 | 0 | 0 | 0 | 0 | 0 | 21 | 31 | 41 | 49 | 35 | 46 | 79 | 0 | 0 | 0 | 0 | 0 | 0 | 0 |
|  | F408 | 24 | 28 | 50 | 52 | 50 | 34 | 40 | 0 | 0 | 0 | 0 | 0 | 0 | 0 | 34 | 72 | 63 | 85 | 80 | 63 | 56 | 0 | 0 | 0 | 0 | 0 | 0 | 0 | 58 | 100 | 113 | 137 | 130 | 97 | 96 | 0 | 0 | 0 | 0 | 0 | 0 | 0 |
|  | F409 | 31 | 29 | 39 | 71 | 58 | 74 | 72 | 0 | 0 | 0 | 0 | 0 | 0 | 0 | 38 | 52 | 67 | 112 | 114 | 129 | 113 | 0 | 0 | 0 | 0 | 0 | 0 | 0 | 69 | 81 | 106 | 183 | 172 | 203 | 185 | 0 | 0 | 0 | 0 | 0 | 0 | 0 |
|  | F410 | 141 | 199 | 324 | 395 | 472 | 436 | 500 | 0 | 0 | 0 | 0 | 0 | 0 | 0 | 217 | 438 | 534 | 707 | 777 | 905 | 950 | 0 | 0 | 0 | 0 | 0 | 0 | 0 | 358 | 637 | 858 | 1102 | 1249 | 1341 | 1450 | 0 | 0 | 0 | 0 | 0 | 0 | 0 |
|  | F411 | 287 | 403 | 763 | 698 | 864 | 1141 | 1069 | 0 | 0 | 0 | 0 | 0 | 0 | 0 | 507 | 683 | 1262 | 1229 | 1661 | 2242 | 2251 | 0 | 0 | 0 | 0 | 0 | 0 | 0 | 794 | 1086 | 2025 | 1927 | 2525 | 3383 | 3320 | 0 | 0 | 0 | 0 | 0 | 0 | 0 |
|  | F412 | 1020 | 1531 | 1921 | 2434 | 2935 | 3429 | 3938 | 0 | 0 | 0 | 0 | 0 | 0 | 0 | 2849 | 3978 | 4835 | 6204 | 7022 | 8611 | 10327 | 0 | 0 | 0 | 0 | 0 | 0 | 0 | 3869 | 5509 | 6756 | 8638 | 9957 | 12040 | 14265 | 0 | 0 | 0 | 0 | 0 | 0 | 0 |
|  | F413 | 108 | 135 | 203 | 280 | 269 | 341 | 199 | 0 | 0 | 0 | 0 | 0 | 0 | 0 | 230 | 327 | 413 | 571 | 533 | 606 | 457 | 0 | 0 | 0 | 0 | 0 | 0 | 0 | 338 | 462 | 616 | 851 | 802 | 947 | 656 | 0 | 0 | 0 | 0 | 0 | 0 | 0 |
|  | F418 | 114 | 249 | 302 | 447 | 617 | 754 | 588 | 0 | 0 | 0 | 0 | 0 | 0 | 0 | 235 | 489 | 568 | 838 | 1102 | 1248 | 983 | 0 | 0 | 0 | 0 | 0 | 0 | 0 | 349 | 738 | 870 | 1285 | 1719 | 2002 | 1571 | 0 | 0 | 0 | 0 | 0 | 0 | 0 |
|  | F419 | 1159 | 1625 | 1833 | 2829 | 3913 | 4662 | 4490 | 0 | 0 | 0 | 0 | 0 | 0 | 0 | 2055 | 2803 | 3191 | 4993 | 6857 | 8935 | 8749 | 0 | 0 | 0 | 0 | 0 | 0 | 1 | 3214 | 4428 | 5024 | 7822 | 10770 | 13597 | 13239 | 0 | 0 | 0 | 0 | 0 | 0 | 1 |
|  | F420 | 9 | 10 | 76 | 51 | 17 | 23 | 37 | 0 | 0 | 0 | 0 | 0 | 0 | 0 | 10 | 12 | 47 | 46 | 47 | 35 | 53 | 0 | 0 | 0 | 0 | 0 | 0 | 0 | 19 | 22 | 123 | 97 | 64 | 58 | 90 | 0 | 0 | 0 | 0 | 0 | 0 | 0 |
|  | F421 | 2 | 7 | 4 | 7 | 4 | 8 | 12 | 0 | 0 | 0 | 0 | 0 | 0 | 0 | 4 | 6 | 3 | 6 | 4 | 5 | 10 | 0 | 0 | 0 | 0 | 0 | 0 | 0 | 6 | 13 | 7 | 13 | 8 | 13 | 22 | 0 | 0 | 0 | 0 | 0 | 0 | 0 |
|  | F422 | 1 | 2 | 5 | 16 | 16 | 27 | 9 | 0 | 0 | 0 | 0 | 0 | 0 | 0 | 0 | 3 | 4 | 17 | 12 | 27 | 7 | 0 | 0 | 0 | 0 | 0 | 0 | 0 | 1 | 5 | 9 | 33 | 28 | 54 | 16 | 0 | 0 | 0 | 0 | 0 | 0 | 0 |
|  | F428 | 15 | 24 | 30 | 37 | 44 | 54 | 51 | 0 | 0 | 0 | 0 | 0 | 0 | 0 | 15 | 18 | 20 | 32 | 39 | 44 | 55 | 0 | 0 | 0 | 0 | 0 | 0 | 0 | 30 | 42 | 50 | 69 | 83 | 98 | 106 | 0 | 0 | 0 | 0 | 0 | 0 | 0 |
|  | F429 | 27 | 67 | 75 | 100 | 111 | 142 | 185 | 0 | 0 | 0 | 0 | 0 | 0 | 0 | 30 | 52 | 63 | 86 | 109 | 128 | 168 | 0 | 0 | 0 | 0 | 0 | 0 | 0 | 57 | 119 | 138 | 186 | 220 | 270 | 353 | 0 | 0 | 0 | 0 | 0 | 0 | 0 |
|  | F430 | 74 | 72 | 69 | 86 | 91 | 108 | 114 | 0 | 0 | 0 | 0 | 0 | 0 | 0 | 176 | 179 | 199 | 157 | 206 | 248 | 288 | 0 | 0 | 0 | 0 | 0 | 0 | 0 | 250 | 251 | 268 | 243 | 297 | 356 | 402 | 0 | 0 | 0 | 0 | 0 | 0 | 0 |
|  | F431 | 78 | 107 | 139 | 226 | 215 | 230 | 274 | 0 | 0 | 0 | 0 | 0 | 0 | 0 | 44 | 97 | 130 | 151 | 158 | 232 | 266 | 0 | 0 | 0 | 0 | 0 | 0 | 0 | 122 | 204 | 269 | 377 | 373 | 462 | 540 | 0 | 0 | 0 | 0 | 0 | 0 | 0 |
|  | F432 | 177 | 343 | 375 | 671 | 694 | 896 | 1312 | 0 | 0 | 0 | 0 | 0 | 0 | 0 | 226 | 544 | 678 | 1133 | 1283 | 2055 | 2523 | 0 | 0 | 0 | 0 | 0 | 0 | 0 | 403 | 887 | 1053 | 1804 | 1977 | 2951 | 3835 | 0 | 0 | 0 | 0 | 0 | 0 | 0 |
|  | F438 | 36 | 17 | 13 | 28 | 4 | 9 | 14 | 0 | 0 | 0 | 0 | 0 | 0 | 0 | 66 | 44 | 36 | 45 | 12 | 34 | 38 | 0 | 0 | 0 | 0 | 0 | 0 | 0 | 102 | 61 | 49 | 73 | 16 | 43 | 52 | 0 | 0 | 0 | 0 | 0 | 0 | 0 |
|  | F439 | 7 | 12 | 12 | 25 | 4 | 10 | 16 | 0 | 0 | 0 | 0 | 0 | 0 | 0 | 14 | 20 | 32 | 48 | 10 | 29 | 46 | 0 | 0 | 0 | 0 | 0 | 0 | 0 | 21 | 32 | 44 | 73 | 14 | 39 | 62 | 0 | 0 | 0 | 0 | 0 | 0 | 0 |
| Sucre | F400 | 0 | 0 | 2 | 3 | 7 | 0 | 3 | 0 | 0 | 0 | 0 | 0 | 0 | 0 | 1 | 1 | 3 | 4 | 1 | 2 | 4 | 0 | 0 | 0 | 0 | 0 | 0 | 0 | 1 | 1 | 5 | 7 | 8 | 2 | 7 | 0 | 0 | 0 | 0 | 0 | 0 | 0 |
|  | F401 | 1 | 2 | 0 | 4 | 5 | 6 | 7 | 0 | 0 | 0 | 0 | 0 | 0 | 0 | 1 | 2 | 2 | 5 | 6 | 3 | 3 | 0 | 0 | 0 | 0 | 0 | 0 | 0 | 2 | 4 | 2 | 9 | 11 | 9 | 10 | 0 | 0 | 0 | 0 | 0 | 0 | 0 |
|  | F402 | 1 | 1 | 4 | 9 | 2 | 3 | 3 | 0 | 0 | 0 | 0 | 0 | 0 | 0 | 3 | 3 | 1 | 11 | 7 | 5 | 9 | 0 | 0 | 0 | 0 | 0 | 0 | 0 | 4 | 4 | 5 | 20 | 9 | 8 | 12 | 0 | 0 | 0 | 0 | 0 | 0 | 0 |
|  | F408 | 4 | 3 | 4 | 13 | 18 | 5 | 12 | 0 | 0 | 0 | 0 | 0 | 0 | 0 | 5 | 10 | 4 | 20 | 21 | 28 | 32 | 0 | 0 | 0 | 0 | 0 | 0 | 0 | 9 | 13 | 8 | 33 | 39 | 33 | 44 | 0 | 0 | 0 | 0 | 0 | 0 | 0 |
|  | F409 | 5 | 7 | 11 | 14 | 20 | 19 | 22 | 0 | 0 | 0 | 0 | 0 | 0 | 0 | 15 | 24 | 36 | 38 | 37 | 30 | 44 | 0 | 0 | 0 | 0 | 0 | 0 | 0 | 20 | 31 | 47 | 52 | 57 | 49 | 66 | 0 | 0 | 0 | 0 | 0 | 0 | 0 |
|  | F410 | 20 | 52 | 146 | 171 | 139 | 101 | 148 | 0 | 0 | 0 | 0 | 0 | 0 | 0 | 39 | 92 | 256 | 290 | 269 | 267 | 320 | 0 | 0 | 0 | 0 | 0 | 0 | 0 | 59 | 144 | 402 | 461 | 408 | 368 | 468 | 0 | 0 | 0 | 0 | 0 | 0 | 0 |
|  | F411 | 15 | 34 | 49 | 99 | 104 | 301 | 377 | 0 | 0 | 0 | 0 | 0 | 0 | 0 | 61 | 69 | 97 | 268 | 263 | 484 | 542 | 0 | 0 | 0 | 0 | 0 | 0 | 0 | 76 | 103 | 146 | 367 | 367 | 785 | 919 | 0 | 0 | 0 | 0 | 0 | 0 | 0 |
|  | F412 | 117 | 185 | 341 | 475 | 614 | 909 | 1567 | 0 | 0 | 0 | 0 | 0 | 0 | 0 | 372 | 483 | 967 | 1254 | 1413 | 2297 | 3975 | 0 | 0 | 0 | 0 | 0 | 0 | 0 | 489 | 668 | 1308 | 1729 | 2027 | 3206 | 5542 | 0 | 0 | 0 | 0 | 0 | 0 | 0 |
|  | F413 | 14 | 17 | 65 | 42 | 41 | 61 | 97 | 0 | 0 | 0 | 0 | 0 | 0 | 0 | 20 | 49 | 129 | 75 | 101 | 133 | 246 | 0 | 0 | 0 | 0 | 0 | 0 | 0 | 34 | 66 | 194 | 117 | 142 | 194 | 343 | 0 | 0 | 0 | 0 | 0 | 0 | 0 |
|  | F418 | 24 | 40 | 88 | 85 | 114 | 100 | 150 | 0 | 0 | 0 | 0 | 0 | 0 | 0 | 60 | 106 | 161 | 182 | 256 | 340 | 325 | 0 | 0 | 0 | 0 | 0 | 0 | 0 | 84 | 146 | 249 | 267 | 370 | 440 | 475 | 0 | 0 | 0 | 0 | 0 | 0 | 0 |
|  | F419 | 176 | 221 | 291 | 399 | 424 | 571 | 908 | 0 | 0 | 0 | 0 | 0 | 0 | 0 | 446 | 478 | 596 | 826 | 890 | 1281 | 1962 | 0 | 0 | 0 | 0 | 0 | 0 | 0 | 622 | 699 | 887 | 1225 | 1314 | 1852 | 2870 | 0 | 0 | 0 | 0 | 0 | 0 | 0 |
|  | F420 | 0 | 0 | 2 | 5 | 6 | 4 | 1 | 0 | 0 | 0 | 0 | 0 | 0 | 0 | 1 | 2 | 0 | 6 | 6 | 1 | 5 | 0 | 0 | 0 | 0 | 0 | 0 | 0 | 1 | 2 | 2 | 11 | 12 | 5 | 6 | 0 | 0 | 0 | 0 | 0 | 0 | 0 |
|  | F421 | 0 | 0 | 0 | 4 | 3 | 1 | 2 | 0 | 0 | 0 | 0 | 0 | 0 | 0 | 1 | 1 | 0 | 8 | 6 | 0 | 2 | 0 | 0 | 0 | 0 | 0 | 0 | 0 | 1 | 1 | 0 | 12 | 9 | 1 | 4 | 0 | 0 | 0 | 0 | 0 | 0 | 0 |
|  | F422 | 0 | 0 | 1 | 2 | 1 | 2 | 2 | 0 | 0 | 0 | 0 | 0 | 0 | 0 | 0 | 0 | 0 | 3 | 4 | 1 | 4 | 0 | 0 | 0 | 0 | 0 | 0 | 0 | 0 | 0 | 1 | 5 | 5 | 3 | 6 | 0 | 0 | 0 | 0 | 0 | 0 | 0 |
|  | F428 | 3 | 2 | 8 | 10 | 17 | 9 | 14 | 0 | 0 | 0 | 0 | 0 | 0 | 0 | 2 | 3 | 2 | 8 | 11 | 6 | 12 | 0 | 0 | 0 | 0 | 0 | 0 | 0 | 5 | 5 | 10 | 18 | 28 | 15 | 26 | 0 | 0 | 0 | 0 | 0 | 0 | 0 |
|  | F429 | 3 | 5 | 13 | 11 | 25 | 38 | 52 | 0 | 0 | 0 | 0 | 0 | 0 | 0 | 9 | 4 | 8 | 10 | 25 | 20 | 37 | 0 | 0 | 0 | 0 | 0 | 0 | 0 | 12 | 9 | 21 | 21 | 50 | 58 | 89 | 0 | 0 | 0 | 0 | 0 | 0 | 0 |
|  | F430 | 6 | 4 | 16 | 9 | 6 | 6 | 23 | 0 | 0 | 0 | 0 | 0 | 0 | 0 | 12 | 11 | 11 | 18 | 22 | 30 | 97 | 0 | 0 | 0 | 0 | 0 | 0 | 0 | 18 | 15 | 27 | 27 | 28 | 36 | 120 | 0 | 0 | 0 | 0 | 0 | 0 | 0 |
|  | F431 | 12 | 9 | 64 | 78 | 47 | 35 | 37 | 0 | 0 | 0 | 0 | 0 | 0 | 0 | 10 | 4 | 38 | 46 | 33 | 34 | 55 | 0 | 0 | 0 | 0 | 0 | 0 | 0 | 22 | 13 | 102 | 124 | 80 | 69 | 92 | 0 | 0 | 0 | 0 | 0 | 0 | 0 |
|  | F432 | 5 | 3 | 5 | 7 | 6 | 7 | 23 | 0 | 0 | 0 | 0 | 0 | 0 | 0 | 5 | 3 | 9 | 15 | 17 | 19 | 80 | 0 | 0 | 0 | 0 | 0 | 0 | 0 | 10 | 6 | 14 | 22 | 23 | 26 | 103 | 0 | 0 | 0 | 0 | 0 | 0 | 0 |
|  | F438 | 6 | 4 | 6 | 4 | 5 | 2 | 5 | 0 | 0 | 0 | 0 | 0 | 0 | 0 | 15 | 13 | 15 | 20 | 5 | 8 | 6 | 0 | 0 | 0 | 0 | 0 | 0 | 0 | 21 | 17 | 21 | 24 | 10 | 10 | 11 | 0 | 0 | 0 | 0 | 0 | 0 | 0 |
|  | F439 | 1 | 2 | 2 | 2 | 4 | 1 | 4 | 0 | 0 | 0 | 0 | 0 | 0 | 0 | 4 | 1 | 3 | 8 | 1 | 3 | 16 | 0 | 0 | 0 | 0 | 0 | 0 | 0 | 5 | 3 | 5 | 10 | 5 | 4 | 20 | 0 | 0 | 0 | 0 | 0 | 0 | 0 |
| Tolima | F400 | 0 | 2 | 2 | 1 | 2 | 2 | 2 | 0 | 0 | 0 | 0 | 0 | 0 | 0 | 5 | 14 | 4 | 6 | 2 | 4 | 8 | 0 | 0 | 0 | 0 | 0 | 0 | 0 | 5 | 16 | 6 | 7 | 4 | 6 | 10 | 0 | 0 | 0 | 0 | 0 | 0 | 0 |
|  | F401 | 2 | 5 | 11 | 8 | 2 | 5 | 11 | 0 | 0 | 0 | 0 | 0 | 0 | 0 | 2 | 4 | 2 | 5 | 7 | 3 | 6 | 0 | 0 | 0 | 0 | 0 | 0 | 0 | 4 | 9 | 13 | 13 | 9 | 8 | 17 | 0 | 0 | 0 | 0 | 0 | 0 | 0 |
|  | F402 | 0 | 5 | 7 | 9 | 9 | 6 | 4 | 0 | 0 | 0 | 0 | 0 | 0 | 0 | 7 | 2 | 8 | 9 | 6 | 6 | 18 | 0 | 0 | 0 | 0 | 0 | 0 | 0 | 7 | 7 | 15 | 18 | 15 | 12 | 22 | 0 | 0 | 0 | 0 | 0 | 0 | 0 |
|  | F408 | 7 | 6 | 8 | 24 | 17 | 17 | 17 | 0 | 0 | 0 | 0 | 0 | 0 | 0 | 8 | 15 | 20 | 29 | 32 | 41 | 42 | 0 | 0 | 0 | 0 | 0 | 0 | 0 | 15 | 21 | 28 | 53 | 49 | 58 | 59 | 0 | 0 | 0 | 0 | 0 | 0 | 0 |
|  | F409 | 9 | 17 | 23 | 31 | 27 | 23 | 37 | 0 | 0 | 0 | 0 | 0 | 0 | 0 | 20 | 28 | 27 | 78 | 47 | 52 | 66 | 0 | 0 | 0 | 0 | 0 | 0 | 0 | 29 | 45 | 50 | 109 | 74 | 75 | 103 | 0 | 0 | 0 | 0 | 0 | 0 | 0 |
|  | F410 | 31 | 40 | 76 | 89 | 68 | 67 | 106 | 0 | 0 | 0 | 0 | 0 | 0 | 0 | 61 | 91 | 120 | 159 | 145 | 154 | 204 | 0 | 0 | 0 | 0 | 0 | 0 | 0 | 92 | 131 | 196 | 248 | 213 | 221 | 310 | 0 | 0 | 0 | 0 | 0 | 0 | 0 |
|  | F411 | 131 | 179 | 236 | 470 | 749 | 886 | 1123 | 0 | 0 | 0 | 0 | 0 | 0 | 0 | 246 | 310 | 464 | 967 | 1444 | 1920 | 2567 | 0 | 0 | 0 | 0 | 0 | 0 | 0 | 377 | 489 | 700 | 1437 | 2193 | 2806 | 3690 | 0 | 0 | 0 | 0 | 0 | 0 | 0 |
|  | F412 | 472 | 805 | 1097 | 1718 | 1889 | 1688 | 2012 | 0 | 0 | 0 | 0 | 0 | 0 | 0 | 1395 | 2197 | 2654 | 3952 | 3736 | 4224 | 5198 | 0 | 0 | 0 | 0 | 0 | 0 | 0 | 1867 | 3002 | 3751 | 5670 | 5625 | 5912 | 7210 | 0 | 0 | 0 | 0 | 0 | 0 | 0 |
|  | F413 | 38 | 83 | 94 | 125 | 141 | 260 | 190 | 0 | 0 | 0 | 0 | 0 | 0 | 0 | 80 | 198 | 186 | 227 | 259 | 486 | 316 | 0 | 0 | 0 | 0 | 0 | 0 | 0 | 118 | 281 | 280 | 352 | 400 | 746 | 506 | 0 | 0 | 0 | 0 | 0 | 0 | 0 |
|  | F418 | 70 | 133 | 162 | 393 | 492 | 618 | 408 | 0 | 0 | 0 | 0 | 0 | 0 | 0 | 178 | 260 | 410 | 759 | 942 | 1301 | 783 | 0 | 0 | 0 | 0 | 0 | 0 | 0 | 248 | 393 | 572 | 1152 | 1434 | 1919 | 1191 | 0 | 0 | 0 | 0 | 0 | 0 | 0 |
|  | F419 | 329 | 464 | 659 | 977 | 1022 | 1330 | 1394 | 0 | 0 | 0 | 0 | 0 | 0 | 0 | 840 | 1043 | 1257 | 1845 | 2026 | 2681 | 2817 | 0 | 0 | 0 | 0 | 0 | 0 | 0 | 1169 | 1507 | 1916 | 2822 | 3048 | 4011 | 4211 | 0 | 0 | 0 | 0 | 0 | 0 | 0 |
|  | F420 | 4 | 3 | 9 | 9 | 13 | 16 | 15 | 0 | 0 | 0 | 0 | 0 | 0 | 0 | 3 | 6 | 3 | 20 | 15 | 8 | 24 | 0 | 0 | 0 | 0 | 0 | 0 | 0 | 7 | 9 | 12 | 29 | 28 | 24 | 39 | 0 | 0 | 0 | 0 | 0 | 0 | 0 |
|  | F421 | 1 | 2 | 1 | 2 | 2 | 2 | 3 | 0 | 0 | 0 | 0 | 0 | 0 | 0 | 0 | 1 | 0 | 2 | 0 | 1 | 4 | 0 | 0 | 0 | 0 | 0 | 0 | 0 | 1 | 3 | 1 | 4 | 2 | 3 | 7 | 0 | 0 | 0 | 0 | 0 | 0 | 0 |
|  | F422 | 1 | 3 | 7 | 2 | 2 | 1 | 2 | 0 | 0 | 0 | 0 | 0 | 0 | 0 | 0 | 0 | 1 | 2 | 0 | 2 | 3 | 0 | 0 | 0 | 0 | 0 | 0 | 0 | 1 | 3 | 8 | 4 | 2 | 3 | 5 | 0 | 0 | 0 | 0 | 0 | 0 | 0 |
|  | F428 | 7 | 10 | 17 | 15 | 14 | 18 | 12 | 0 | 0 | 0 | 0 | 0 | 0 | 0 | 3 | 3 | 10 | 14 | 6 | 6 | 14 | 0 | 0 | 0 | 0 | 0 | 0 | 0 | 10 | 13 | 27 | 29 | 20 | 24 | 26 | 0 | 0 | 0 | 0 | 0 | 0 | 0 |
|  | F429 | 10 | 17 | 29 | 33 | 34 | 43 | 78 | 0 | 0 | 0 | 0 | 0 | 0 | 0 | 4 | 10 | 12 | 28 | 35 | 27 | 57 | 0 | 0 | 0 | 0 | 0 | 0 | 0 | 14 | 27 | 41 | 61 | 69 | 70 | 135 | 0 | 0 | 0 | 0 | 0 | 0 | 0 |
|  | F430 | 6 | 17 | 51 | 57 | 24 | 32 | 53 | 0 | 0 | 0 | 0 | 0 | 0 | 0 | 18 | 36 | 121 | 139 | 125 | 136 | 184 | 0 | 0 | 0 | 0 | 1 | 0 | 0 | 24 | 53 | 172 | 196 | 149 | 168 | 237 | 0 | 0 | 0 | 0 | 1 | 0 | 0 |
|  | F431 | 17 | 23 | 105 | 143 | 89 | 73 | 112 | 0 | 0 | 0 | 0 | 0 | 0 | 0 | 20 | 44 | 51 | 67 | 50 | 83 | 126 | 0 | 0 | 0 | 0 | 0 | 0 | 0 | 37 | 67 | 156 | 210 | 139 | 156 | 238 | 0 | 0 | 0 | 0 | 0 | 0 | 0 |
|  | F432 | 50 | 58 | 134 | 216 | 171 | 212 | 337 | 0 | 0 | 0 | 0 | 0 | 0 | 0 | 65 | 102 | 196 | 339 | 261 | 346 | 693 | 0 | 0 | 0 | 0 | 0 | 0 | 0 | 115 | 160 | 330 | 555 | 432 | 558 | 1030 | 0 | 0 | 0 | 0 | 0 | 0 | 0 |
|  | F438 | 0 | 5 | 1 | 6 | 4 | 13 | 9 | 0 | 0 | 0 | 0 | 0 | 0 | 0 | 5 | 4 | 6 | 3 | 5 | 34 | 16 | 0 | 0 | 0 | 0 | 0 | 0 | 0 | 5 | 9 | 7 | 9 | 9 | 47 | 25 | 0 | 0 | 0 | 0 | 0 | 0 | 0 |
|  | F439 | 0 | 3 | 1 | 4 | 2 | 7 | 5 | 0 | 0 | 0 | 0 | 0 | 0 | 0 | 1 | 4 | 4 | 7 | 7 | 11 | 18 | 0 | 0 | 0 | 0 | 0 | 0 | 0 | 1 | 7 | 5 | 11 | 9 | 18 | 23 | 0 | 0 | 0 | 0 | 0 | 0 | 0 |
| Valle del Cau | F400 | 3 | 5 | 11 | 4 | 12 | 7 | 12 | 0 | 0 | 0 | 0 | 0 | 0 | 0 | 11 | 4 | 9 | 8 | 20 | 11 | 17 | 0 | 0 | 0 | 0 | 0 | 0 | 0 | 14 | 9 | 20 | 12 | 32 | 18 | 29 | 0 | 0 | 0 | 0 | 0 | 0 | 0 |
|  | F401 | 15 | 16 | 13 | 15 | 17 | 21 | 19 | 0 | 0 | 0 | 0 | 0 | 0 | 0 | 11 | 11 | 11 | 22 | 18 | 21 | 23 | 0 | 0 | 0 | 0 | 0 | 0 | 0 | 26 | 27 | 24 | 37 | 35 | 42 | 42 | 0 | 0 | 0 | 0 | 0 | 0 | 0 |
|  | F402 | 14 | 8 | 17 | 35 | 11 | 21 | 26 | 0 | 0 | 0 | 0 | 0 | 0 | 0 | 32 | 19 | 37 | 53 | 40 | 48 | 43 | 0 | 0 | 0 | 0 | 0 | 0 | 0 | 46 | 27 | 54 | 88 | 51 | 69 | 69 | 0 | 0 | 0 | 0 | 0 | 0 | 0 |
|  | F408 | 23 | 33 | 35 | 53 | 48 | 65 | 47 | 0 | 0 | 0 | 0 | 0 | 0 | 0 | 61 | 69 | 63 | 122 | 70 | 121 | 97 | 0 | 0 | 0 | 0 | 0 | 0 | 0 | 84 | 102 | 98 | 175 | 118 | 186 | 144 | 0 | 0 | 0 | 0 | 0 | 0 | 0 |
|  | F409 | 56 | 69 | 50 | 84 | 93 | 127 | 108 | 0 | 0 | 0 | 0 | 0 | 0 | 0 | 127 | 135 | 123 | 159 | 147 | 223 | 224 | 0 | 0 | 0 | 0 | 0 | 0 | 0 | 183 | 204 | 173 | 243 | 240 | 350 | 332 | 0 | 0 | 0 | 0 | 0 | 0 | 0 |
|  | F410 | 169 | 234 | 213 | 330 | 388 | 412 | 379 | 0 | 0 | 0 | 0 | 0 | 0 | 0 | 334 | 403 | 404 | 608 | 685 | 899 | 776 | 0 | 0 | 0 | 0 | 0 | 0 | 0 | 503 | 637 | 617 | 938 | 1073 | 1311 | 1155 | 0 | 0 | 0 | 0 | 0 | 0 | 0 |
|  | F411 | 523 | 625 | 685 | 1442 | 2282 | 2980 | 2859 | 0 | 0 | 0 | 0 | 0 | 0 | 0 | 1321 | 1787 | 1589 | 3006 | 4372 | 6314 | 6366 | 0 | 0 | 0 | 0 | 0 | 0 | 0 | 1844 | 2412 | 2274 | 4448 | 6654 | 9294 | 9225 | 0 | 0 | 0 | 0 | 0 | 0 | 0 |
|  | F412 | 2204 | 2538 | 3192 | 5054 | 4837 | 7628 | 8307 | 0 | 0 | 0 | 1 | 0 | 0 | 0 | 7607 | 8315 | 9710 | 14752 | 12897 | 20951 | 23184 | 0 | 0 | 0 | 0 | 0 | 1 | 0 | 9811 | 10853 | 12902 | 19806 | 17734 | 28579 | 31491 | 0 | 0 | 0 | 1 | 0 | 1 | 0 |
|  | F413 | 212 | 298 | 366 | 579 | 554 | 579 | 588 | 0 | 0 | 0 | 0 | 0 | 0 | 0 | 504 | 728 | 838 | 1162 | 1128 | 1290 | 1079 | 0 | 0 | 0 | 0 | 0 | 0 | 0 | 716 | 1026 | 1204 | 1741 | 1682 | 1869 | 1667 | 0 | 0 | 0 | 0 | 0 | 0 | 0 |
|  | F418 | 411 | 532 | 568 | 932 | 1268 | 1946 | 1467 | 0 | 0 | 0 | 0 | 0 | 0 | 0 | 1115 | 1249 | 1324 | 1983 | 2715 | 4206 | 3085 | 0 | 0 | 0 | 0 | 0 | 0 | 0 | 1526 | 1781 | 1892 | 2915 | 3983 | 6152 | 4552 | 0 | 0 | 0 | 0 | 0 | 0 | 0 |
|  | F419 | 1947 | 2214 | 2480 | 4525 | 5051 | 6772 | 6041 | 0 | 0 | 0 | 0 | 0 | 0 | 0 | 4597 | 5008 | 5179 | 9143 | 9945 | 14213 | 12395 | 0 | 0 | 0 | 0 | 0 | 0 | 0 | 6544 | 7222 | 7659 | 13668 | 14996 | 20985 | 18436 | 0 | 0 | 0 | 0 | 0 | 0 | 0 |
|  | F420 | 8 | 17 | 12 | 33 | 32 | 35 | 47 | 0 | 0 | 0 | 0 | 0 | 0 | 0 | 21 | 20 | 16 | 51 | 28 | 34 | 50 | 0 | 0 | 0 | 0 | 0 | 0 | 0 | 29 | 37 | 28 | 84 | 60 | 69 | 97 | 0 | 0 | 0 | 0 | 0 | 0 | 0 |
|  | F421 | 2 | 6 | 7 | 17 | 17 | 16 | 17 | 0 | 0 | 0 | 0 | 0 | 0 | 0 | 7 | 13 | 9 | 19 | 9 | 13 | 25 | 0 | 0 | 0 | 0 | 0 | 0 | 0 | 9 | 19 | 16 | 36 | 26 | 29 | 42 | 0 | 0 | 0 | 0 | 0 | 0 | 0 |
|  | F422 | 6 | 9 | 7 | 10 | 16 | 23 | 13 | 0 | 0 | 0 | 0 | 0 | 0 | 0 | 10 | 6 | 10 | 19 | 24 | 29 | 19 | 0 | 0 | 0 | 0 | 0 | 0 | 0 | 16 | 15 | 17 | 29 | 40 | 52 | 32 | 0 | 0 | 0 | 0 | 0 | 0 | 0 |
|  | F428 | 25 | 30 | 24 | 48 | 47 | 60 | 73 | 0 | 0 | 0 | 0 | 0 | 0 | 0 | 19 | 24 | 31 | 37 | 41 | 59 | 68 | 0 | 0 | 0 | 0 | 0 | 0 | 0 | 44 | 54 | 55 | 85 | 88 | 119 | 141 | 0 | 0 | 0 | 0 | 0 | 0 | 0 |
|  | F429 | 105 | 106 | 104 | 157 | 175 | 200 | 181 | 0 | 0 | 0 | 0 | 0 | 0 | 0 | 73 | 74 | 75 | 139 | 124 | 157 | 165 | 0 | 0 | 0 | 0 | 0 | 0 | 0 | 178 | 180 | 179 | 296 | 299 | 357 | 346 | 0 | 0 | 0 | 0 | 0 | 0 | 0 |
|  | F430 | 123 | 163 | 97 | 158 | 143 | 132 | 107 | 0 | 0 | 0 | 0 | 0 | 0 | 0 | 339 | 563 | 201 | 375 | 446 | 345 | 259 | 0 | 0 | 0 | 0 | 0 | 0 | 0 | 462 | 726 | 298 | 533 | 589 | 477 | 366 | 0 | 0 | 0 | 0 | 0 | 0 | 0 |
|  | F431 | 66 | 95 | 157 | 224 | 201 | 216 | 432 | 0 | 0 | 0 | 0 | 0 | 0 | 0 | 142 | 137 | 171 | 255 | 233 | 271 | 601 | 0 | 0 | 0 | 0 | 0 | 0 | 0 | 208 | 232 | 328 | 479 | 434 | 487 | 1033 | 0 | 0 | 0 | 0 | 0 | 0 | 0 |
|  | F432 | 368 | 557 | 845 | 1136 | 942 | 1500 | 1317 | 0 | 0 | 0 | 0 | 0 | 0 | 0 | 986 | 1458 | 1523 | 2151 | 1689 | 2294 | 2312 | 0 | 0 | 0 | 0 | 0 | 0 | 0 | 1354 | 2015 | 2368 | 3287 | 2631 | 3794 | 3629 | 0 | 0 | 0 | 0 | 0 | 0 | 0 |
|  | F438 | 9 | 17 | 26 | 47 | 16 | 24 | 35 | 0 | 0 | 0 | 0 | 0 | 0 | 0 | 39 | 59 | 59 | 87 | 53 | 71 | 78 | 0 | 0 | 0 | 0 | 0 | 0 | 0 | 48 | 76 | 85 | 134 | 69 | 95 | 113 | 0 | 0 | 0 | 0 | 0 | 0 | 0 |
|  | F439 | 27 | 16 | 15 | 25 | 18 | 22 | 30 | 0 | 0 | 0 | 0 | 0 | 0 | 0 | 77 | 48 | 56 | 73 | 37 | 69 | 51 | 0 | 0 | 0 | 0 | 0 | 0 | 0 | 104 | 64 | 71 | 98 | 55 | 91 | 81 | 0 | 0 | 0 | 0 | 0 | 0 | 0 |
| Vaupés | F400 | 0 | 0 | 0 | 1 | 1 | 0 | 0 | 0 | 0 | 0 | 0 | 0 | 0 | 0 | 0 | 0 | 0 | 0 | 0 | 0 | 0 | 0 | 0 | 0 | 0 | 0 | 0 | 0 | 0 | 0 | 0 | 1 | 1 | 0 | 0 | 0 | 0 | 0 | 0 | 0 | 0 | 0 |
|  | F401 | 0 | 0 | 0 | 0 | 1 | 1 | 1 | 0 | 0 | 0 | 0 | 0 | 0 | 0 | 0 | 0 | 0 | 0 | 0 | 1 | 2 | 0 | 0 | 0 | 0 | 0 | 0 | 0 | 0 | 0 | 0 | 0 | 1 | 2 | 3 | 0 | 0 | 0 | 0 | 0 | 0 | 0 |
|  | F402 | 0 | 1 | 0 | 0 | 0 | 0 | 0 | 0 | 0 | 0 | 0 | 0 | 0 | 0 | 0 | 0 | 0 | 0 | 0 | 0 | 0 | 0 | 0 | 0 | 0 | 0 | 0 | 0 | 0 | 1 | 0 | 0 | 0 | 0 | 0 | 0 | 0 | 0 | 0 | 0 | 0 | 0 |
|  | F408 | 0 | 0 | 0 | 0 | 0 | 0 | 0 | 0 | 0 | 0 | 0 | 0 | 0 | 0 | 0 | 0 | 0 | 1 | 0 | 0 | 1 | 0 | 0 | 0 | 0 | 0 | 0 | 0 | 0 | 0 | 0 | 1 | 0 | 0 | 1 | 0 | 0 | 0 | 0 | 0 | 0 | 0 |
|  | F409 | 0 | 0 | 0 | 0 | 0 | 0 | 0 | 0 | 0 | 0 | 0 | 0 | 0 | 0 | 0 | 0 | 0 | 0 | 2 | 1 | 2 | 0 | 0 | 0 | 0 | 0 | 0 | 0 | 0 | 0 | 0 | 0 | 2 | 1 | 2 | 0 | 0 | 0 | 0 | 0 | 0 | 0 |
|  | F410 | 0 | 0 | 0 | 0 | 0 | 2 | 0 | 0 | 0 | 0 | 0 | 0 | 0 | 0 | 1 | 2 | 0 | 1 | 5 | 1 | 4 | 0 | 0 | 0 | 0 | 0 | 0 | 0 | 1 | 2 | 0 | 1 | 5 | 3 | 4 | 0 | 0 | 0 | 0 | 0 | 0 | 0 |
|  | F411 | 0 | 0 | 1 | 2 | 2 | 1 | 2 | 0 | 0 | 0 | 0 | 0 | 0 | 0 | 0 | 0 | 1 | 1 | 3 | 1 | 4 | 0 | 0 | 0 | 0 | 0 | 0 | 0 | 0 | 0 | 2 | 3 | 5 | 2 | 6 | 0 | 0 | 0 | 0 | 0 | 0 | 0 |
|  | F412 | 0 | 0 | 4 | 2 | 2 | 1 | 14 | 0 | 0 | 0 | 0 | 0 | 0 | 0 | 1 | 1 | 5 | 12 | 11 | 2 | 11 | 0 | 0 | 0 | 0 | 0 | 0 | 0 | 1 | 1 | 9 | 14 | 13 | 3 | 25 | 0 | 0 | 0 | 0 | 0 | 0 | 0 |
|  | F413 | 0 | 0 | 1 | 3 | 3 | 0 | 3 | 0 | 0 | 0 | 0 | 0 | 0 | 0 | 1 | 0 | 0 | 0 | 0 | 0 | 2 | 0 | 0 | 0 | 0 | 0 | 0 | 0 | 1 | 0 | 1 | 3 | 3 | 0 | 5 | 0 | 0 | 0 | 0 | 0 | 0 | 0 |
|  | F418 | 1 | 1 | 1 | 1 | 1 | 0 | 3 | 0 | 0 | 0 | 0 | 0 | 0 | 0 | 0 | 0 | 1 | 1 | 5 | 0 | 3 | 0 | 0 | 0 | 0 | 0 | 0 | 0 | 1 | 1 | 2 | 2 | 6 | 0 | 6 | 0 | 0 | 0 | 0 | 0 | 0 | 0 |
|  | F419 | 0 | 1 | 1 | 7 | 4 | 6 | 9 | 0 | 0 | 0 | 0 | 0 | 0 | 0 | 1 | 0 | 2 | 3 | 10 | 10 | 25 | 0 | 0 | 0 | 0 | 0 | 0 | 0 | 1 | 1 | 3 | 10 | 14 | 16 | 34 | 0 | 0 | 0 | 0 | 0 | 0 | 0 |
|  | F430 | 0 | 0 | 0 | 0 | 0 | 1 | 0 | 0 | 0 | 0 | 0 | 0 | 0 | 0 | 0 | 0 | 0 | 1 | 3 | 1 | 0 | 0 | 0 | 0 | 0 | 0 | 0 | 0 | 0 | 0 | 0 | 1 | 3 | 2 | 0 | 0 | 0 | 0 | 0 | 0 | 0 | 0 |
|  | F431 | 0 | 0 | 2 | 2 | 2 | 3 | 2 | 0 | 0 | 0 | 0 | 0 | 0 | 0 | 0 | 0 | 1 | 1 | 1 | 2 | 3 | 0 | 0 | 0 | 0 | 0 | 0 | 0 | 0 | 0 | 3 | 3 | 3 | 5 | 5 | 0 | 0 | 0 | 0 | 0 | 0 | 0 |
|  | F432 | 1 | 0 | 1 | 3 | 2 | 7 | 12 | 0 | 0 | 0 | 0 | 0 | 0 | 0 | 0 | 0 | 0 | 2 | 3 | 5 | 12 | 0 | 0 | 0 | 0 | 0 | 0 | 0 | 1 | 0 | 1 | 5 | 5 | 12 | 24 | 0 | 0 | 0 | 0 | 0 | 0 | 0 |
|  | F438 | 0 | 0 | 0 | 1 | 0 | 0 | 0 | 0 | 0 | 0 | 0 | 0 | 0 | 0 | 0 | 0 | 1 | 0 | 0 | 0 | 0 | 0 | 0 | 0 | 0 | 0 | 0 | 0 | 0 | 0 | 1 | 1 | 0 | 0 | 0 | 0 | 0 | 0 | 0 | 0 | 0 | 0 |
| Vichada | F401 | 0 | 0 | 0 | 0 | 0 | 0 | 0 | 0 | 0 | 0 | 0 | 0 | 0 | 0 | 0 | 0 | 0 | 0 | 0 | 1 | 0 | 0 | 0 | 0 | 0 | 0 | 0 | 0 | 0 | 0 | 0 | 0 | 0 | 1 | 0 | 0 | 0 | 0 | 0 | 0 | 0 | 0 |
|  | F402 | 0 | 0 | 0 | 0 | 0 | 0 | 0 | 0 | 0 | 0 | 0 | 0 | 0 | 0 | 0 | 0 | 1 | 1 | 2 | 1 | 0 | 0 | 0 | 0 | 0 | 0 | 0 | 0 | 0 | 0 | 1 | 1 | 2 | 1 | 0 | 0 | 0 | 0 | 0 | 0 | 0 | 0 |
|  | F408 | 0 | 0 | 0 | 0 | 0 | 1 | 0 | 0 | 0 | 0 | 0 | 0 | 0 | 0 | 0 | 1 | 0 | 0 | 0 | 1 | 0 | 0 | 0 | 0 | 0 | 0 | 0 | 0 | 0 | 1 | 0 | 0 | 0 | 2 | 0 | 0 | 0 | 0 | 0 | 0 | 0 | 0 |
|  | F409 | 0 | 0 | 0 | 0 | 0 | 1 | 0 | 0 | 0 | 0 | 0 | 0 | 0 | 0 | 0 | 0 | 0 | 0 | 0 | 3 | 0 | 0 | 0 | 0 | 0 | 0 | 0 | 0 | 0 | 0 | 0 | 0 | 0 | 4 | 0 | 0 | 0 | 0 | 0 | 0 | 0 | 0 |
|  | F410 | 0 | 1 | 1 | 2 | 2 | 2 | 3 | 0 | 0 | 0 | 0 | 0 | 0 | 0 | 1 | 2 | 0 | 7 | 2 | 1 | 11 | 0 | 0 | 0 | 0 | 0 | 0 | 0 | 1 | 3 | 1 | 9 | 4 | 3 | 14 | 0 | 0 | 0 | 0 | 0 | 0 | 0 |
|  | F411 | 0 | 2 | 2 | 15 | 18 | 11 | 21 | 0 | 0 | 0 | 0 | 0 | 0 | 0 | 1 | 2 | 7 | 21 | 32 | 21 | 43 | 0 | 0 | 0 | 0 | 0 | 0 | 0 | 1 | 4 | 9 | 36 | 50 | 32 | 64 | 0 | 0 | 0 | 0 | 0 | 0 | 0 |
|  | F412 | 0 | 6 | 3 | 21 | 19 | 16 | 14 | 0 | 0 | 0 | 0 | 0 | 0 | 0 | 2 | 12 | 19 | 31 | 37 | 36 | 37 | 0 | 0 | 0 | 0 | 0 | 0 | 0 | 2 | 18 | 22 | 52 | 56 | 52 | 51 | 0 | 0 | 0 | 0 | 0 | 0 | 0 |
|  | F413 | 0 | 0 | 2 | 0 | 0 | 0 | 1 | 0 | 0 | 0 | 0 | 0 | 0 | 0 | 0 | 0 | 1 | 0 | 2 | 1 | 0 | 0 | 0 | 0 | 0 | 0 | 0 | 0 | 0 | 0 | 3 | 0 | 2 | 1 | 1 | 0 | 0 | 0 | 0 | 0 | 0 | 0 |
|  | F418 | 1 | 0 | 1 | 5 | 6 | 5 | 2 | 0 | 0 | 0 | 0 | 0 | 0 | 0 | 1 | 1 | 4 | 3 | 8 | 10 | 9 | 0 | 0 | 0 | 0 | 0 | 0 | 0 | 2 | 1 | 5 | 8 | 14 | 15 | 11 | 0 | 0 | 0 | 0 | 0 | 0 | 0 |
|  | F419 | 0 | 9 | 8 | 11 | 17 | 14 | 26 | 0 | 0 | 0 | 0 | 0 | 0 | 0 | 1 | 17 | 7 | 25 | 38 | 23 | 44 | 0 | 0 | 0 | 0 | 0 | 0 | 0 | 1 | 26 | 15 | 36 | 55 | 37 | 70 | 0 | 0 | 0 | 0 | 0 | 0 | 0 |
|  | F420 | 0 | 0 | 0 | 0 | 0 | 0 | 2 | 0 | 0 | 0 | 0 | 0 | 0 | 0 | 0 | 0 | 0 | 0 | 0 | 0 | 0 | 0 | 0 | 0 | 0 | 0 | 0 | 0 | 0 | 0 | 0 | 0 | 0 | 0 | 2 | 0 | 0 | 0 | 0 | 0 | 0 | 0 |
|  | F422 | 0 | 0 | 0 | 1 | 0 | 0 | 0 | 0 | 0 | 0 | 0 | 0 | 0 | 0 | 0 | 0 | 0 | 0 | 0 | 0 | 0 | 0 | 0 | 0 | 0 | 0 | 0 | 0 | 0 | 0 | 0 | 1 | 0 | 0 | 0 | 0 | 0 | 0 | 0 | 0 | 0 | 0 |
|  | F428 | 0 | 0 | 0 | 0 | 0 | 0 | 1 | 0 | 0 | 0 | 0 | 0 | 0 | 0 | 0 | 0 | 0 | 0 | 0 | 0 | 0 | 0 | 0 | 0 | 0 | 0 | 0 | 0 | 0 | 0 | 0 | 0 | 0 | 0 | 1 | 0 | 0 | 0 | 0 | 0 | 0 | 0 |
|  | F429 | 0 | 0 | 0 | 1 | 0 | 2 | 0 | 0 | 0 | 0 | 0 | 0 | 0 | 0 | 0 | 0 | 0 | 0 | 0 | 0 | 1 | 0 | 0 | 0 | 0 | 0 | 0 | 0 | 0 | 0 | 0 | 1 | 0 | 2 | 1 | 0 | 0 | 0 | 0 | 0 | 0 | 0 |
|  | F430 | 0 | 0 | 1 | 2 | 1 | 1 | 1 | 0 | 0 | 0 | 0 | 0 | 0 | 0 | 0 | 0 | 2 | 3 | 0 | 3 | 2 | 0 | 0 | 0 | 0 | 0 | 0 | 0 | 0 | 0 | 3 | 5 | 1 | 4 | 3 | 0 | 0 | 0 | 0 | 0 | 0 | 0 |
|  | F431 | 0 | 0 | 0 | 0 | 0 | 1 | 0 | 0 | 0 | 0 | 0 | 0 | 0 | 0 | 0 | 2 | 1 | 4 | 5 | 3 | 8 | 0 | 0 | 0 | 0 | 0 | 0 | 0 | 0 | 2 | 1 | 4 | 5 | 4 | 8 | 0 | 0 | 0 | 0 | 0 | 0 | 0 |
|  | F432 | 3 | 0 | 7 | 4 | 2 | 2 | 4 | 0 | 0 | 0 | 0 | 0 | 0 | 0 | 1 | 2 | 2 | 3 | 3 | 4 | 9 | 0 | 0 | 0 | 0 | 0 | 0 | 0 | 4 | 2 | 9 | 7 | 5 | 6 | 13 | 0 | 0 | 0 | 0 | 0 | 0 | 0 |
|  | F438 | 0 | 0 | 0 | 0 | 1 | 0 | 0 | 0 | 0 | 0 | 0 | 0 | 0 | 0 | 0 | 0 | 0 | 0 | 1 | 0 | 0 | 0 | 0 | 0 | 0 | 0 | 0 | 0 | 0 | 0 | 0 | 0 | 2 | 0 | 0 | 0 | 0 | 0 | 0 | 0 | 0 | 0 |
| Colombia | F400 | 66 | 84 | 124 | 151 | 125 | 173 | 124 | 0 | 0 | 0 | 0 | 0 | 0 | 0 | 133 | 197 | 143 | 273 | 207 | 238 | 218 | 0 | 0 | 0 | 0 | 0 | 0 | 0 | 199 | 281 | 267 | 424 | 332 | 411 | 342 | 0 | 0 | 0 | 0 | 0 | 0 | 0 |
|  | F401 | 174 | 213 | 258 | 392 | 320 | 394 | 595 | 0 | 0 | 0 | 0 | 0 | 0 | 0 | 161 | 181 | 207 | 365 | 340 | 547 | 711 | 0 | 0 | 0 | 0 | 0 | 0 | 0 | 335 | 394 | 465 | 757 | 660 | 941 | 1306 | 0 | 0 | 0 | 0 | 0 | 0 | 0 |
|  | F402 | 135 | 159 | 198 | 302 | 206 | 295 | 265 | 0 | 0 | 0 | 0 | 0 | 0 | 0 | 251 | 255 | 347 | 593 | 413 | 444 | 483 | 1 | 0 | 0 | 0 | 0 | 0 | 0 | 386 | 414 | 545 | 895 | 619 | 739 | 748 | 1 | 0 | 0 | 0 | 0 | 0 | 0 |
|  | F408 | 293 | 345 | 469 | 684 | 669 | 643 | 571 | 0 | 0 | 0 | 0 | 0 | 0 | 0 | 507 | 634 | 800 | 1176 | 1217 | 1301 | 1197 | 0 | 0 | 0 | 0 | 0 | 0 | 0 | 800 | 979 | 1269 | 1860 | 1886 | 1944 | 1768 | 0 | 0 | 0 | 0 | 0 | 0 | 0 |
|  | F409 | 521 | 622 | 672 | 1027 | 1163 | 1328 | 1002 | 0 | 0 | 0 | 0 | 0 | 0 | 0 | 1056 | 1285 | 1342 | 2100 | 2138 | 2460 | 2126 | 0 | 0 | 0 | 0 | 0 | 0 | 0 | 1577 | 1907 | 2014 | 3127 | 3301 | 3788 | 3128 | 0 | 0 | 0 | 0 | 0 | 0 | 0 |
|  | F410 | 2266 | 2892 | 3582 | 5005 | 4948 | 5248 | 4518 | 0 | 0 | 0 | 0 | 0 | 0 | 0 | 4387 | 5346 | 6794 | 9626 | 9082 | 10792 | 9521 | 0 | 0 | 0 | 0 | 0 | 0 | 0 | 6653 | 8238 | 10376 | 14631 | 14030 | 16040 | 14039 | 0 | 0 | 0 | 0 | 0 | 0 | 0 |
|  | F411 | 5654 | 7461 | 10283 | 18584 | 21754 | 26677 | 23783 | 0 | 0 | 0 | 0 | 0 | 0 | 0 | 13399 | 16444 | 21650 | 39224 | 44178 | 57131 | 50803 | 0 | 0 | 0 | 0 | 0 | 0 | 0 | 19053 | 23905 | 31933 | 57808 | 65932 | 83808 | 74586 | 0 | 0 | 0 | 0 | 0 | 0 | 0 |
|  | F412 | 18969 | 24973 | 33927 | 50831 | 49873 | 63678 | 68584 | 0 | 0 | 0 | 2 | 2 | 0 | 1 | 60431 | 75558 | 94754 | 141305 | 130083 | 172971 | 185237 | 0 | 0 | 0 | 1 | 1 | 1 | 0 | 79400 | 100531 | 128681 | 192136 | 179956 | 236649 | 253821 | 0 | 0 | 0 | 3 | 3 | 1 | 1 |
|  | F413 | 1564 | 2294 | 3254 | 4803 | 4921 | 5832 | 4694 | 0 | 0 | 0 | 0 | 0 | 0 | 0 | 3637 | 5431 | 7014 | 9971 | 10060 | 12678 | 10167 | 1 | 0 | 0 | 0 | 0 | 0 | 0 | 5201 | 7725 | 10268 | 14774 | 14981 | 18510 | 14861 | 1 | 0 | 0 | 0 | 0 | 0 | 0 |
|  | F418 | 3383 | 4649 | 6141 | 9829 | 12246 | 14092 | 10755 | 0 | 0 | 0 | 0 | 0 | 0 | 0 | 7518 | 10145 | 11794 | 19510 | 22808 | 28685 | 21495 | 0 | 0 | 1 | 0 | 0 | 0 | 0 | 10901 | 14794 | 17935 | 29339 | 35054 | 42777 | 32250 | 0 | 0 | 1 | 0 | 0 | 0 | 0 |
|  | F419 | 16918 | 21196 | 26051 | 39768 | 43422 | 53919 | 48002 | 0 | 0 | 0 | 0 | 0 | 0 | 2 | 37998 | 45448 | 54320 | 81630 | 84909 | 110353 | 98706 | 0 | 0 | 0 | 0 | 2 | 2 | 1 | 54916 | 66644 | 80371 | 121398 | 128331 | 164272 | 146708 | 0 | 0 | 0 | 0 | 2 | 2 | 3 |
|  | F420 | 319 | 347 | 469 | 657 | 504 | 567 | 537 | 0 | 0 | 0 | 0 | 0 | 0 | 0 | 367 | 401 | 437 | 671 | 574 | 595 | 674 | 0 | 0 | 0 | 0 | 0 | 0 | 0 | 686 | 748 | 906 | 1328 | 1078 | 1162 | 1211 | 0 | 0 | 0 | 0 | 0 | 0 | 0 |
|  | F421 | 69 | 89 | 122 | 173 | 172 | 164 | 181 | 0 | 0 | 0 | 0 | 0 | 0 | 0 | 97 | 122 | 128 | 218 | 213 | 184 | 198 | 0 | 0 | 0 | 0 | 0 | 0 | 0 | 166 | 211 | 250 | 391 | 385 | 348 | 379 | 0 | 0 | 0 | 0 | 0 | 0 | 0 |
|  | F422 | 107 | 140 | 140 | 216 | 194 | 244 | 193 | 0 | 0 | 0 | 0 | 0 | 0 | 0 | 158 | 161 | 163 | 274 | 244 | 354 | 230 | 0 | 0 | 0 | 0 | 0 | 0 | 0 | 265 | 301 | 303 | 490 | 438 | 598 | 423 | 0 | 0 | 0 | 0 | 0 | 0 | 0 |
|  | F428 | 349 | 397 | 576 | 1029 | 967 | 1369 | 1597 | 0 | 0 | 0 | 0 | 0 | 0 | 0 | 289 | 331 | 471 | 1750 | 2154 | 3005 | 3395 | 0 | 0 | 0 | 0 | 0 | 0 | 0 | 638 | 728 | 1047 | 2779 | 3121 | 4374 | 4992 | 0 | 0 | 0 | 0 | 0 | 0 | 0 |
|  | F429 | 768 | 1026 | 1334 | 1955 | 2015 | 2063 | 2250 | 0 | 0 | 0 | 0 | 0 | 0 | 0 | 671 | 750 | 1106 | 2124 | 2271 | 2215 | 2412 | 0 | 0 | 0 | 0 | 0 | 0 | 0 | 1439 | 1776 | 2440 | 4079 | 4286 | 4278 | 4662 | 0 | 0 | 0 | 0 | 0 | 0 | 0 |
|  | F430 | 804 | 1030 | 1363 | 2089 | 1988 | 1765 | 1930 | 0 | 0 | 0 | 0 | 0 | 0 | 0 | 3314 | 3459 | 2929 | 5163 | 6364 | 6594 | 5972 | 0 | 0 | 0 | 0 | 1 | 1 | 0 | 4118 | 4489 | 4292 | 7252 | 8352 | 8359 | 7902 | 0 | 0 | 0 | 0 | 1 | 1 | 0 |
|  | F431 | 1085 | 1428 | 2987 | 4111 | 2969 | 3296 | 3701 | 0 | 0 | 0 | 0 | 0 | 0 | 0 | 1501 | 1836 | 2316 | 3487 | 2546 | 3377 | 4147 | 0 | 0 | 0 | 0 | 0 | 0 | 0 | 2586 | 3264 | 5303 | 7598 | 5515 | 6673 | 7848 | 0 | 0 | 0 | 0 | 0 | 0 | 0 |
|  | F432 | 4607 | 5412 | 7939 | 12848 | 11750 | 13628 | 15348 | 0 | 0 | 0 | 0 | 0 | 0 | 0 | 12527 | 13847 | 17159 | 26028 | 23783 | 27071 | 29757 | 0 | 0 | 0 | 1 | 0 | 0 | 0 | 17134 | 19259 | 25098 | 38876 | 35533 | 40699 | 45105 | 0 | 0 | 0 | 1 | 0 | 0 | 0 |
|  | F438 | 172 | 204 | 205 | 381 | 351 | 345 | 364 | 0 | 0 | 0 | 0 | 0 | 0 | 0 | 458 | 485 | 434 | 802 | 1036 | 981 | 994 | 0 | 0 | 0 | 0 | 0 | 0 | 0 | 630 | 689 | 639 | 1183 | 1387 | 1326 | 1358 | 0 | 0 | 0 | 0 | 0 | 0 | 0 |
|  | F439 | 129 | 161 | 184 | 320 | 266 | 234 | 294 | 0 | 0 | 0 | 0 | 0 | 0 | 0 | 292 | 339 | 392 | 690 | 726 | 629 | 652 | 0 | 0 | 0 | 0 | 0 | 0 | 0 | 421 | 500 | 576 | 1010 | 992 | 863 | 946 | 0 | 0 | 0 | 0 | 0 | 0 | 0 |
