# Supplementary material for: Burden of anxiety disorders in Colombia: subnational DALY estimates and territorial inequalities, 2016–2022
Source: Popul Health Metr. 2026 Sep 15;24:55. doi: 10.1186/s12963-026-00510-4 (PMC13591821; doi:10.1186/s12963-026-00510-4)
Supplement: Supplementary file 2 [file 12963_2026_510_MOESM2_ESM.docx]

# Supplementary Table S2.

Department-level burden of anxiety disorders in Colombia by sex: cumulative crude rates, cumulative age-standardized DALY rates, and annual age-standardized DALY rates, 2016–2022

Detailed department-level estimates stratified by sex including cumulative crude rates, cumulative age-standardized DALY rates, annual percentage change (APC), and annual age-standardized DALY rates for the study period. Rates are expressed per 100,000 population.

| Department | Sex | APC (95% CI) | Crude Rate (Period, 95% CI) | Age-Standardized Rate (Period, 95% CI) | Crude Rate 2016 (95% CI) | Crude Rate 2017 (95% CI) | Crude Rate 2018 (95% CI) | Crude Rate 2019 (95% CI) | Crude Rate 2020 (95% CI) | Crude Rate 2021 (95% CI) | Crude Rate 2022 (95% CI) | Age-Standardized Rate 2016 (95% CI) | Age-Standardized Rate 2017 (95% CI) | Age-Standardized Rate 2018 (95% CI) | Age-Standardized Rate 2019 (95% CI) | Age-Standardized Rate 2020 (95% CI) | Age-Standardized Rate 2021 (95% CI) | Age-Standardized Rate 2022 (95% CI) |
| --- | --- | --- | --- | --- | --- | --- | --- | --- | --- | --- | --- | --- | --- | --- | --- | --- | --- | --- |
| Amazonas | Men | 25.48 (7.33-46.69) | 80.2 (66.2-94.1) | 20.2 (16.6-23.8) | 22.0 (2.1-41.8) | 43.4 (15.8-71.0) | 59.0 (26.5-91.5) | 112.0 (68.0-155.9) | 121.6 (76.6-166.5) | 59.1 (28.2-89.9) | 135.0 (89.0-180.9) | 6.0 (0.2-11.8) | 12.4 (4.2-20.5) | 14.9 (6.5-23.2) | 27.9 (16.7-39.1) | 30.5 (19.0-42.1) | 14.7 (6.8-22.5) | 35.1 (22.8-47.3) |
|  | Women | 19.55 (8.60-31.61) | 210.8 (187.2-234.3) | 54.1 (47.9-60.3) | 69.6 (32.5-106.7) | 170.5 (113.1-227.9) | 170.2 (112.5-227.8) | 240.1 (173.1-307.2) | 246.7 (180.2-313.3) | 214.2 (153.3-275.1) | 336.5 (261.5-411.6) | 20.5 (9.2-31.8) | 46.4 (30.5-62.3) | 44.9 (29.3-60.5) | 62.2 (44.5-79.9) | 62.8 (45.5-80.2) | 54.4 (38.6-70.2) | 87.2 (67.4-107.0) |
|  | Total | 21.11 (8.66-34.98) | 142.8 (129.3-156.2) | 37.1 (33.5-40.7) | 44.6 (24.1-65.1) | 103.9 (73.0-134.8) | 112.1 (79.8-144.5) | 173.4 (134.0-212.9) | 181.7 (142.1-221.3) | 133.9 (100.5-167.3) | 232.4 (189.1-275.8) | 13.2 (6.9-19.6) | 29.4 (20.4-38.3) | 29.9 (21.1-38.7) | 45.0 (34.6-55.5) | 46.7 (36.3-57.1) | 34.5 (25.7-43.4) | 61.1 (49.5-72.8) |
| Antioquia | Men | 14.23 (1.79-28.20) | 365.0 (362.0-368.0) | 488.8 (443.6-534.0) | 202.6 (196.5-208.6) | 261.5 (254.7-268.3) | 330.7 (323.0-338.3) | 501.1 (491.8-510.3) | 398.7 (390.6-406.9) | 509.7 (500.5-518.8) | 331.7 (324.4-339.0) | 288.4 (186.1-390.8) | 316.0 (211.7-420.4) | 310.6 (222.1-399.1) | 673.7 (537.0-810.4) | 695.1 (553.2-836.9) | 679.3 (541.2-817.4) | 458.4 (343.4-573.3) |
|  | Women | 13.37 (1.72-26.34) | 876.5 (872.1-880.9) | 967.3 (917.2-1017.4) | 526.8 (517.5-536.0) | 672.0 (661.7-682.3) | 814.4 (803.0-825.7) | 1189.9 (1176.4-1203.5) | 906.2 (894.5-917.9) | 1184.0 (1170.8-1197.3) | 808.6 (797.8-819.5) | 509.6 (400.2-619.0) | 790.2 (650.0-930.4) | 674.7 (574.2-775.1) | 1157.6 (1021.8-1293.4) | 1266.7 (1117.4-1416.0) | 1505.5 (1343.7-1667.3) | 866.8 (746.3-987.4) |
|  | Total | 13.60 (2.00-26.53) | 633.8 (631.1-636.5) | 728.1 (694.3-761.8) | 373.6 (368.0-379.2) | 477.9 (471.6-484.2) | 585.2 (578.2-592.1) | 863.0 (854.6-871.4) | 665.0 (657.7-672.2) | 863.2 (855.0-871.4) | 581.6 (574.9-588.3) | 399.0 (324.1-474.0) | 553.1 (465.8-640.5) | 492.6 (425.7-559.6) | 915.7 (819.3-1012.0) | 980.9 (877.9-1083.9) | 1092.4 (986.0-1198.8) | 662.6 (579.3-745.9) |
| Arauca | Men | 130.59 (75.93-202.24) | 190.0 (178.9-201.0) | 263.5 (68.8-458.1) | 25.5 (14.4-36.7) | 58.2 (41.6-74.8) | 98.0 (76.7-119.3) | 168.7 (141.1-196.2) | 225.9 (194.4-257.5) | 323.8 (286.4-361.2) | 391.7 (350.8-432.6) | 6.2 (3.4-8.9) | 14.7 (10.4-18.9) | 24.2 (18.9-29.5) | 369.1 (-261.5-999.6) | 215.4 (-218.4-649.1) | 606.2 (-181.7-1394.2) | 608.5 (-197.6-1414.5) |
|  | Women | 65.26 (33.86-104.01) | 441.1 (424.2-458.1) | 381.6 (184.6-578.6) | 96.0 (74.1-117.9) | 176.2 (147.1-205.4) | 232.9 (199.9-265.9) | 429.4 (385.2-473.6) | 476.5 (430.6-522.4) | 701.2 (646.1-756.3) | 878.7 (817.6-939.8) | 24.3 (18.6-29.9) | 193.3 (-210.9-597.5) | 201.8 (-189.1-592.7) | 241.6 (-123.3-606.4) | 258.7 (-123.1-640.6) | 839.0 (30.2-1647.8) | 912.4 (105.1-1719.7) |
|  | Total | 79.34 (47.08-118.68) | 315.0 (304.9-325.1) | 322.5 (184.1-461.0) | 60.3 (48.1-72.5) | 116.6 (99.9-133.3) | 165.0 (145.4-184.6) | 298.4 (272.4-324.4) | 351.0 (323.1-378.8) | 512.6 (479.3-545.9) | 635.8 (599.0-672.6) | 15.2 (12.1-18.4) | 104.0 (-98.1-306.1) | 113.0 (-82.5-308.5) | 305.3 (-58.9-669.5) | 237.0 (-51.9-526.0) | 722.6 (158.0-1287.2) | 760.4 (190.0-1330.8) |
| Archipiélago | Men | 116.38 (29.12-262.61) | 140.9 (121.9-160.0) | 247.3 (-163.9-658.6) | 24.0 (3.1-44.9) | 90.3 (49.7-130.8) | 26.6 (4.6-48.7) | 72.7 (36.4-109.0) | 225.3 (161.8-288.7) | 230.5 (166.7-294.2) | 305.9 (232.7-379.1) | 5.6 (0.7-10.6) | 22.6 (12.2-33.1) | 6.2 (1.0-11.4) | 17.4 (8.6-26.1) | 801.8 (-1233.8-2837.4) | 55.7 (40.1-71.4) | 821.9 (-1213.7-2857.6) |
|  | Women | 76.81 (-15.89-271.66) | 326.3 (298.7-353.9) | 597.1 (-200.0-1394.3) | 61.0 (29.3-92.7) | 184.8 (129.8-239.8) | 36.9 (12.1-61.6) | 203.0 (145.2-260.8) | 407.6 (326.2-489.1) | 517.2 (426.0-608.5) | 849.6 (733.1-966.0) | 14.5 (6.9-22.2) | 1353.4 (-2208.9-4915.6) | 9.1 (2.9-15.2) | 45.9 (32.6-59.2) | 93.3 (74.3-112.3) | 1042.2 (-1472.4-3556.8) | 1621.5 (-1860.2-5103.2) |
|  | Total | 90.26 (-2.72-272.10) | 238.1 (221.0-255.1) | 422.2 (-26.3-870.7) | 43.4 (24.1-62.8) | 140.0 (105.3-174.8) | 32.0 (15.3-48.7) | 141.0 (106.1-175.8) | 320.7 (268.4-373.0) | 380.4 (323.8-437.0) | 590.2 (520.0-660.4) | 10.1 (5.5-14.6) | 688.0 (-1093.1-2469.1) | 7.6 (3.6-11.7) | 31.6 (23.7-39.6) | 447.5 (-570.3-1465.4) | 548.9 (-708.4-1806.3) | 1221.7 (-794.8-3238.3) |
| Atlántico | Men | 16.87 (9.81-24.39) | 334.9 (330.3-339.5) | 428.1 (355.5-500.6) | 157.0 (148.2-165.7) | 183.9 (174.5-193.2) | 297.3 (285.6-309.0) | 384.9 (371.8-397.9) | 348.9 (336.7-361.1) | 424.2 (410.9-437.5) | 501.8 (487.5-516.1) | 258.2 (94.0-422.5) | 250.2 (92.8-407.7) | 388.5 (206.2-570.7) | 446.5 (253.9-639.2) | 524.9 (314.1-735.7) | 423.1 (243.3-603.0) | 705.0 (462.3-947.7) |
|  | Women | 23.01 (17.47-28.82) | 639.2 (633.1-645.3) | 872.9 (790.3-955.5) | 329.1 (317.1-341.2) | 355.6 (343.2-368.0) | 457.0 (443.1-470.8) | 677.3 (660.7-693.8) | 641.9 (626.0-657.7) | 894.1 (875.6-912.6) | 1023.4 (1003.8-1043.0) | 508.9 (318.8-699.0) | 457.9 (281.8-634.0) | 574.9 (404.0-745.8) | 832.9 (627.3-1038.6) | 938.1 (717.5-1158.7) | 1330.7 (1067.2-1594.3) | 1466.9 (1188.2-1745.7) |
|  | Total | 21.07 (17.02-25.25) | 493.8 (489.9-497.6) | 650.5 (595.5-705.5) | 246.9 (239.3-254.5) | 273.5 (265.7-281.4) | 380.9 (371.7-390.0) | 537.7 (527.1-548.4) | 501.8 (491.7-511.9) | 669.2 (657.6-680.7) | 773.5 (761.2-785.8) | 383.6 (257.9-509.2) | 354.1 (235.9-472.2) | 481.7 (356.8-606.6) | 639.7 (498.9-780.6) | 731.5 (578.9-884.0) | 876.9 (717.4-1036.5) | 1086.0 (901.1-1270.8) |
| Bogotá, D.C. | Men | 20.73 (9.80-32.76) | 418.9 (416.0-421.8) | 667.9 (613.4-722.4) | 222.6 (216.8-228.3) | 251.5 (245.4-257.6) | 323.2 (316.2-330.1) | 475.9 (467.6-484.1) | 510.7 (502.2-519.1) | 581.9 (573.0-590.9) | 530.9 (522.4-539.4) | 264.8 (166.8-362.8) | 502.1 (363.1-641.2) | 470.5 (350.3-590.7) | 718.4 (571.8-865.0) | 951.7 (782.5-1121.0) | 999.2 (826.7-1171.8) | 768.2 (619.0-917.3) |
|  | Women | 15.27 (4.89-26.68) | 865.6 (861.6-869.5) | 1061.0 (1010.3-1111.8) | 490.3 (482.3-498.4) | 528.6 (520.3-536.9) | 664.5 (655.2-673.9) | 990.1 (978.8-1001.4) | 1024.3 (1013.0-1035.6) | 1202.6 (1190.4-1214.7) | 1103.6 (1092.0-1115.2) | 525.6 (419.5-631.8) | 874.3 (732.3-1016.3) | 735.9 (629.3-842.6) | 1200.7 (1063.0-1338.3) | 1538.7 (1380.1-1697.3) | 1443.3 (1292.8-1593.8) | 1108.7 (980.3-1237.1) |
|  | Total | 17.29 (6.80-28.81) | 654.9 (652.4-657.4) | 864.4 (827.2-901.7) | 364.9 (359.8-370.0) | 398.8 (393.5-404.0) | 503.8 (497.9-509.7) | 747.4 (740.3-754.5) | 781.3 (774.1-788.5) | 908.5 (900.8-916.1) | 831.9 (824.6-839.2) | 395.2 (323.0-467.5) | 688.2 (588.8-787.6) | 603.2 (522.9-683.6) | 959.5 (859.0-1060.1) | 1245.2 (1129.2-1361.2) | 1221.3 (1106.8-1335.7) | 938.4 (840.0-1036.8) |
| Bolívar | Men | 33.69 (23.52-44.71) | 253.0 (248.4-257.5) | 356.7 (286.3-427.1) | 89.0 (81.8-96.3) | 132.4 (123.7-141.2) | 186.5 (176.1-196.9) | 258.6 (246.5-270.8) | 249.8 (238.1-261.6) | 344.1 (330.4-357.8) | 481.4 (465.4-497.5) | 113.8 (12.2-215.4) | 142.4 (29.9-254.9) | 312.8 (137.7-487.8) | 326.6 (149.9-503.2) | 434.8 (226.3-643.3) | 415.0 (218.2-611.7) | 751.5 (476.6-1026.5) |
|  | Women | 40.08 (22.05-60.78) | 537.3 (530.9-543.8) | 685.7 (604.3-767.0) | 232.0 (220.5-243.4) | 307.1 (294.0-320.1) | 397.6 (382.7-412.4) | 511.6 (494.9-528.2) | 497.0 (480.8-513.2) | 741.2 (721.6-760.8) | 1016.5 (993.7-1039.3) | 116.5 (43.5-189.5) | 402.6 (227.9-577.4) | 514.2 (327.4-700.9) | 674.5 (462.1-886.8) | 775.1 (543.9-1006.3) | 731.4 (518.9-944.0) | 1585.4 (1253.8-1917.1) |
|  | Total | 37.03 (23.57-51.95) | 398.6 (394.6-402.6) | 521.2 (467.4-575.0) | 162.1 (155.3-169.0) | 221.8 (213.8-229.7) | 294.7 (285.6-303.9) | 388.3 (377.9-398.7) | 376.5 (366.4-386.5) | 547.5 (535.4-559.5) | 755.3 (741.3-769.4) | 115.1 (52.6-177.7) | 272.5 (168.6-376.4) | 413.5 (285.5-541.4) | 500.5 (362.4-638.6) | 604.9 (449.3-760.6) | 573.2 (428.4-718.0) | 1168.5 (953.1-1383.9) |
| Boyacá | Men | 29.19 (19.57-39.57) | 304.5 (298.3-310.8) | 323.5 (257.7-389.4) | 110.3 (100.1-120.4) | 160.2 (148.1-172.4) | 251.0 (235.8-266.2) | 326.3 (309.1-343.4) | 358.9 (341.1-376.7) | 394.1 (375.6-412.6) | 503.2 (482.4-523.9) | 108.0 (8.8-207.1) | 192.0 (56.0-327.9) | 196.4 (67.7-325.0) | 344.3 (165.9-522.7) | 469.4 (255.8-683.1) | 468.7 (256.1-681.2) | 486.1 (274.2-698.1) |
|  | Women | 42.56 (29.40-57.06) | 655.0 (646.1-663.9) | 539.9 (472.9-607.0) | 239.6 (225.1-254.0) | 350.2 (332.9-367.6) | 513.3 (492.2-534.4) | 710.1 (685.5-734.7) | 715.6 (691.1-740.0) | 889.2 (862.2-916.2) | 1116.8 (1086.8-1146.9) | 111.2 (40.9-181.5) | 223.2 (107.4-339.1) | 308.2 (180.9-435.5) | 593.7 (404.0-783.4) | 752.5 (533.8-971.3) | 894.7 (662.6-1126.7) | 896.0 (676.9-1115.1) |
|  | Total | 36.86 (25.59-49.15) | 484.9 (479.5-490.4) | 431.7 (384.8-478.7) | 177.2 (168.3-186.2) | 258.6 (247.8-269.3) | 386.0 (372.9-399.1) | 523.6 (508.5-538.8) | 542.0 (526.8-557.3) | 648.2 (631.7-664.8) | 818.1 (799.7-836.5) | 109.6 (48.8-170.4) | 207.6 (118.3-296.9) | 252.3 (161.8-342.8) | 469.0 (338.8-599.2) | 611.0 (458.1-763.9) | 681.7 (524.3-839.0) | 691.1 (538.6-843.5) |
| Caldas | Men | 50.59 (25.51-80.69) | 528.2 (519.2-537.1) | 554.2 (454.3-654.0) | 204.2 (189.3-219.1) | 236.4 (220.5-252.4) | 336.1 (317.0-355.1) | 683.1 (656.2-710.0) | 727.1 (699.6-754.6) | 777.0 (748.8-805.2) | 696.9 (670.3-723.5) | 81.0 (3.2-158.7) | 167.8 (25.3-310.4) | 230.8 (74.1-387.5) | 719.8 (418.7-1021.0) | 1059.3 (681.9-1436.7) | 887.3 (550.8-1223.8) | 733.1 (431.2-1035.0) |
|  | Women | 24.21 (11.94-37.82) | 1214.9 (1202.0-1227.9) | 1107.9 (992.6-1223.3) | 556.5 (533.0-580.0) | 586.2 (562.2-610.1) | 822.7 (794.2-851.2) | 1567.0 (1528.1-1605.9) | 1538.4 (1500.3-1576.6) | 1746.2 (1705.8-1786.6) | 1611.8 (1573.3-1650.4) | 381.9 (194.9-568.8) | 761.5 (471.0-1052.1) | 736.5 (489.8-983.3) | 1311.5 (988.1-1634.8) | 1580.8 (1216.2-1945.4) | 1641.9 (1278.7-2005.0) | 1341.4 (1021.6-1661.3) |
|  | Total | 30.75 (15.49-48.02) | 887.5 (879.5-895.5) | 831.0 (754.8-907.3) | 388.5 (374.3-402.7) | 419.5 (404.8-434.2) | 590.6 (573.2-608.1) | 1145.5 (1121.4-1169.5) | 1151.5 (1127.6-1175.3) | 1284.1 (1259.0-1309.1) | 1175.8 (1151.9-1199.6) | 231.4 (130.2-332.6) | 464.7 (302.8-626.5) | 483.7 (337.6-629.8) | 1015.6 (794.7-1236.6) | 1320.1 (1057.7-1582.4) | 1264.6 (1017.0-1512.1) | 1037.3 (817.3-1257.2) |
| Caquetá | Men | 38.12 (-12.12-117.07) | 160.1 (151.8-168.4) | 197.2 (65.6-328.9) | 56.0 (42.9-69.0) | 67.8 (53.5-82.2) | 115.2 (96.2-134.2) | 164.5 (142.0-186.9) | 156.9 (135.2-178.5) | 228.3 (202.5-254.2) | 317.9 (287.7-348.1) | 112.1 (-156.8-381.0) | 16.8 (13.2-20.4) | 245.0 (-171.9-661.9) | 40.3 (34.8-45.8) | 39.2 (33.7-44.6) | 469.7 (-93.2-1032.6) | 457.4 (-77.5-992.3) |
|  | Women | 10.05 (-11.91-37.48) | 357.8 (345.5-370.2) | 236.8 (109.7-363.9) | 146.2 (124.9-167.5) | 169.5 (146.7-192.3) | 227.7 (201.0-254.4) | 316.9 (285.9-348.0) | 336.8 (305.3-368.3) | 538.0 (498.7-577.2) | 717.1 (672.3-761.8) | 250.6 (-161.7-662.9) | 146.7 (-140.1-433.6) | 260.3 (-132.7-653.3) | 77.0 (69.4-84.6) | 176.3 (-79.7-432.3) | 229.6 (-39.4-498.6) | 517.4 (20.6-1014.2) |
|  | Total | 19.64 (-9.18-57.60) | 259.3 (251.8-266.7) | 217.0 (125.5-308.5) | 100.7 (88.3-113.2) | 118.3 (104.9-131.8) | 171.5 (155.1-187.9) | 241.0 (221.8-260.1) | 247.4 (228.3-266.6) | 384.7 (361.1-408.3) | 520.1 (493.0-547.2) | 181.3 (-64.8-427.5) | 81.8 (-61.7-225.2) | 252.6 (-33.8-539.1) | 58.6 (53.9-63.3) | 107.7 (-20.3-235.8) | 349.7 (37.7-661.6) | 487.4 (122.4-852.4) |
| Casanare | Men | -20.42 (-34.28--3.64) | 200.5 (191.7-209.3) | 440.1 (201.9-678.2) | 115.2 (97.0-133.4) | 171.3 (149.4-193.2) | 156.9 (135.9-177.9) | 222.3 (197.6-246.9) | 179.2 (157.4-200.9) | 281.3 (254.4-308.2) | 261.8 (236.1-287.5) | 742.5 (-124.0-1609.0) | 878.8 (-47.8-1805.5) | 465.0 (-204.1-1134.2) | 186.3 (-172.9-545.6) | 171.2 (-176.2-518.5) | 442.2 (-146.3-1030.7) | 194.3 (-161.5-550.2) |
|  | Women | -3.45 (-30.73-34.59) | 408.0 (395.4-420.5) | 509.3 (290.4-728.2) | 264.5 (236.7-292.3) | 374.2 (341.6-406.8) | 280.6 (252.4-308.8) | 452.8 (417.7-488.0) | 303.1 (274.9-331.3) | 555.2 (517.6-592.8) | 586.0 (547.8-624.2) | 331.5 (-174.0-837.1) | 833.0 (13.5-1652.5) | 638.5 (-52.8-1329.7) | 430.5 (-70.3-931.4) | 74.2 (67.2-81.2) | 715.0 (70.8-1359.2) | 542.3 (15.2-1069.5) |
|  | Total | -11.49 (-29.78-11.56) | 304.2 (296.6-311.9) | 474.7 (312.9-636.4) | 189.3 (172.8-205.9) | 272.2 (252.6-291.7) | 218.5 (201.0-236.1) | 337.4 (316.0-358.9) | 241.3 (223.5-259.1) | 418.9 (395.8-442.1) | 425.1 (402.1-448.2) | 537.0 (35.4-1038.6) | 855.9 (237.4-1474.4) | 551.7 (70.7-1032.8) | 308.4 (0.2-616.7) | 122.7 (-51.0-296.4) | 578.6 (142.3-1014.9) | 368.3 (50.3-686.3) |
| Cauca | Men | 33.61 (25.04-42.76) | 178.2 (173.8-182.6) | 339.3 (263.7-414.9) | 73.6 (65.9-81.2) | 93.0 (84.4-101.5) | 123.9 (114.0-133.9) | 197.9 (185.4-210.3) | 194.8 (182.6-207.0) | 264.9 (250.9-279.0) | 283.6 (269.2-298.1) | 109.0 (3.3-214.7) | 175.1 (38.4-311.8) | 232.6 (60.1-405.0) | 231.4 (70.8-391.9) | 443.6 (211.9-675.3) | 615.8 (343.6-888.0) | 567.7 (309.4-826.0) |
|  | Women | 27.88 (19.07-37.33) | 403.4 (396.9-409.9) | 429.7 (360.5-499.0) | 160.4 (149.3-171.5) | 237.2 (223.8-250.6) | 286.1 (271.3-300.8) | 446.6 (428.4-464.8) | 411.3 (394.0-428.6) | 611.7 (590.8-632.5) | 635.2 (614.1-656.3) | 201.9 (70.8-333.1) | 181.7 (67.3-296.1) | 318.0 (155.0-481.1) | 444.5 (258.5-630.5) | 387.7 (217.9-557.5) | 736.0 (495.7-976.4) | 738.3 (500.4-976.2) |
|  | Total | 30.16 (24.83-35.72) | 293.5 (289.6-297.5) | 384.5 (333.3-435.8) | 118.0 (111.2-124.8) | 166.7 (158.7-174.7) | 207.1 (198.2-216.1) | 325.4 (314.3-336.6) | 305.7 (295.1-316.4) | 442.5 (429.8-455.2) | 463.6 (450.7-476.4) | 155.5 (71.3-239.7) | 178.4 (89.3-267.5) | 275.3 (156.6-394.0) | 337.9 (215.1-460.8) | 415.6 (272.0-559.3) | 675.9 (494.4-857.5) | 653.0 (477.4-828.6) |
| Cesar | Men | 24.71 (10.16-41.18) | 278.4 (272.0-284.7) | 319.4 (221.3-417.6) | 127.6 (115.8-139.5) | 181.0 (167.1-194.9) | 243.3 (227.1-259.5) | 362.6 (343.3-381.9) | 309.5 (292.1-326.8) | 316.2 (299.0-333.4) | 373.9 (355.5-392.3) | 117.5 (-41.6-276.6) | 168.2 (-16.6-352.9) | 207.9 (7.0-408.8) | 510.0 (173.3-846.8) | 377.4 (97.9-656.8) | 478.5 (153.0-804.0) | 376.7 (103.4-650.0) |
|  | Women | 14.59 (6.91-22.83) | 499.8 (491.5-508.0) | 699.9 (559.5-840.2) | 210.3 (195.4-225.1) | 321.4 (303.4-339.5) | 403.1 (382.8-423.3) | 623.2 (598.6-647.7) | 540.8 (518.6-563.1) | 596.4 (573.5-619.4) | 725.3 (700.4-750.2) | 369.8 (70.2-669.5) | 692.6 (285.9-1099.4) | 500.1 (192.3-807.9) | 742.3 (370.6-1114.1) | 696.4 (341.2-1051.6) | 922.2 (504.6-1339.8) | 975.7 (555.7-1395.8) |
|  | Total | 17.23 (8.87-26.24) | 392.2 (387.0-397.5) | 509.7 (424.0-595.3) | 170.0 (160.4-179.5) | 253.0 (241.5-264.5) | 325.6 (312.5-338.7) | 496.8 (481.0-512.5) | 428.6 (414.3-442.8) | 460.4 (446.0-474.9) | 554.8 (539.2-570.4) | 243.7 (74.0-413.3) | 430.4 (207.0-653.8) | 354.0 (170.2-537.8) | 626.2 (375.4-877.0) | 536.9 (310.9-762.9) | 700.3 (435.6-965.1) | 676.2 (425.6-926.8) |
| Chocó | Men | 123.80 (47.08-240.54) | 98.1 (92.2-104.1) | 197.5 (86.2-308.7) | 18.2 (11.3-25.1) | 19.8 (12.7-26.9) | 43.4 (32.7-54.1) | 56.5 (44.4-68.5) | 49.1 (38.0-60.1) | 54.5 (43.0-65.9) | 419.0 (387.5-450.4) | 4.4 (2.7-6.1) | 5.1 (3.2-6.9) | 10.4 (7.8-13.0) | 91.6 (-120.1-303.2) | 12.0 (9.2-14.7) | 85.8 (-109.4-281.0) | 1173.0 (449.2-1896.7) |
|  | Women | 73.55 (25.88-139.26) | 279.0 (269.4-288.7) | 290.5 (179.7-401.3) | 70.7 (57.6-83.8) | 64.4 (52.1-76.8) | 78.4 (64.6-92.3) | 139.6 (121.4-157.8) | 127.7 (110.6-144.9) | 192.9 (172.1-213.8) | 1205.7 (1154.2-1257.3) | 33.7 (-55.0-122.5) | 30.0 (-47.0-107.1) | 36.3 (-47.0-119.7) | 566.1 (107.4-1024.8) | 336.4 (-3.4-676.1) | 172.9 (-42.8-388.5) | 858.2 (401.7-1314.7) |
|  | Total | 89.70 (38.95-159.00) | 191.9 (186.1-197.7) | 244.0 (165.5-322.5) | 45.4 (37.8-52.9) | 42.9 (35.6-50.2) | 61.6 (52.8-70.5) | 99.6 (88.5-110.7) | 89.8 (79.5-100.2) | 126.2 (114.0-138.3) | 825.8 (795.2-856.5) | 19.1 (-25.3-63.4) | 17.5 (-21.0-56.1) | 23.4 (-18.3-65.1) | 328.8 (76.2-581.4) | 174.2 (4.3-344.1) | 129.3 (-16.1-274.8) | 1015.6 (587.7-1443.4) |
| Cundinamarca | Men | 18.77 (7.51-31.21) | 245.1 (241.5-248.7) | 353.2 (297.4-409.1) | 128.8 (121.5-136.2) | 156.4 (148.5-164.3) | 220.4 (211.3-229.5) | 262.8 (253.0-272.5) | 274.9 (265.2-284.7) | 336.4 (325.8-347.0) | 296.6 (286.8-306.3) | 199.7 (83.1-316.4) | 153.8 (58.3-249.3) | 389.3 (228.7-549.9) | 356.2 (209.2-503.2) | 457.6 (290.4-624.7) | 465.7 (300.0-631.5) | 450.3 (285.4-615.3) |
|  | Women | 15.09 (8.18-22.43) | 568.6 (563.3-573.9) | 648.0 (586.3-709.7) | 335.5 (324.0-347.1) | 389.6 (377.5-401.7) | 534.9 (521.0-548.8) | 623.6 (608.9-638.2) | 589.2 (575.2-603.1) | 761.7 (746.1-777.3) | 671.7 (657.3-686.2) | 349.0 (214.9-483.1) | 513.7 (347.5-679.9) | 510.0 (369.4-650.6) | 692.2 (527.1-857.2) | 751.9 (579.6-924.3) | 977.9 (783.7-1172.1) | 741.1 (577.7-904.6) |
|  | Total | 16.03 (10.01-22.39) | 410.3 (407.0-413.5) | 500.6 (459.0-542.2) | 234.7 (227.8-241.6) | 275.9 (268.6-283.2) | 381.0 (372.6-389.4) | 446.9 (438.0-455.7) | 435.2 (426.6-443.7) | 553.2 (543.7-562.7) | 487.8 (479.0-496.6) | 274.4 (185.5-363.2) | 333.8 (237.9-429.6) | 449.7 (342.9-556.4) | 524.2 (413.7-634.7) | 604.7 (484.7-724.8) | 721.8 (594.1-849.5) | 595.7 (479.6-711.9) |
| Córdoba | Men | 36.34 (25.64-47.96) | 146.0 (142.3-149.7) | 162.6 (117.3-207.9) | 49.5 (43.8-55.3) | 71.4 (64.6-78.3) | 88.9 (81.1-96.7) | 144.7 (134.9-154.5) | 161.1 (150.9-171.3) | 195.8 (184.7-206.9) | 294.1 (280.6-307.6) | 61.6 (-13.5-136.6) | 78.8 (-2.6-160.3) | 71.7 (-7.3-150.8) | 106.5 (15.1-197.9) | 197.5 (62.8-332.1) | 212.5 (76.7-348.3) | 409.5 (217.2-601.7) |
|  | Women | 32.16 (27.99-36.47) | 338.8 (333.3-344.3) | 443.9 (372.6-515.1) | 122.0 (113.1-130.9) | 178.2 (167.5-188.9) | 228.2 (215.9-240.4) | 310.8 (296.7-324.9) | 338.8 (324.3-353.3) | 462.2 (445.5-479.0) | 689.2 (669.0-709.4) | 155.9 (36.9-274.9) | 233.5 (86.8-380.2) | 279.0 (121.5-436.4) | 409.7 (223.9-595.5) | 511.3 (306.1-716.5) | 580.1 (371.7-788.5) | 937.5 (676.6-1198.4) |
|  | Total | 33.25 (28.58-38.08) | 244.3 (240.9-247.6) | 303.2 (261.0-345.4) | 86.3 (80.9-91.6) | 125.6 (119.3-132.0) | 160.1 (152.7-167.4) | 229.5 (220.9-238.2) | 251.8 (242.9-260.8) | 331.8 (321.7-341.9) | 495.7 (483.5-508.0) | 108.7 (38.4-179.1) | 156.2 (72.3-240.1) | 175.4 (87.3-263.4) | 258.1 (154.6-361.6) | 354.4 (231.7-477.1) | 396.3 (271.9-520.7) | 673.5 (511.5-835.5) |
| Guainía | Men | 57.31 (13.39-118.25) | 87.1 (68.1-106.0) | 20.3 (15.7-24.9) | 22.5 (-4.8-49.8) | 8.6 (-7.9-25.0) | 12.6 (-7.2-32.3) | 145.5 (80.4-210.6) | 122.2 (64.1-180.4) | 145.2 (83.4-207.1) | 121.2 (65.9-176.4) | 6.5 (-1.6-14.6) | 2.0 (-1.9-5.9) | 2.8 (-1.9-7.6) | 34.6 (18.3-50.9) | 29.6 (14.9-44.3) | 37.4 (21.2-53.5) | 29.2 (15.3-43.1) |
|  | Women | 70.88 (27.67-128.71) | 144.3 (118.5-170.2) | 34.1 (27.8-40.4) | 19.0 (-7.7-45.8) | 16.9 (-7.6-41.4) | 16.5 (-7.5-40.5) | 198.4 (117.8-279.1) | 224.1 (140.7-307.4) | 261.8 (174.2-349.3) | 209.6 (133.3-285.9) | 5.4 (-2.2-13.0) | 4.1 (-2.0-10.1) | 4.0 (-2.0-9.9) | 50.8 (29.5-72.1) | 58.1 (36.0-80.2) | 64.5 (42.3-86.7) | 51.9 (32.4-71.4) |
|  | Total | 64.62 (21.33-123.35) | 114.1 (98.3-129.9) | 27.2 (23.3-31.1) | 20.9 (1.7-40.0) | 12.5 (-2.0-26.9) | 14.4 (-1.0-29.8) | 170.4 (119.1-221.6) | 170.3 (120.4-220.2) | 200.4 (147.7-253.1) | 163.2 (116.8-209.6) | 6.0 (0.4-11.5) | 3.0 (-0.6-6.6) | 3.4 (-0.4-7.2) | 42.7 (29.3-56.1) | 43.9 (30.6-57.1) | 50.9 (37.2-64.7) | 40.6 (28.6-52.5) |
| Guaviare | Men | 104.34 (50.05-178.28) | 94.1 (80.4-107.9) | 125.9 (-147.1-399.0) | 14.5 (-0.1-29.1) | 33.5 (11.6-55.3) | 61.3 (31.6-91.0) | 118.5 (77.6-159.3) | 112.3 (72.9-151.7) | 133.3 (90.7-175.9) | 174.7 (126.3-223.2) | 3.2 (-0.0-6.4) | 7.6 (2.6-12.7) | 14.6 (7.4-21.8) | 29.6 (19.3-39.9) | 27.7 (17.7-37.6) | 32.7 (22.1-43.3) | 766.1 (-1145.3-2677.6) |
|  | Women | 42.40 (26.23-60.64) | 252.5 (227.9-277.1) | 60.5 (54.5-66.5) | 73.7 (37.3-110.2) | 85.3 (46.8-123.7) | 91.7 (51.9-131.5) | 284.5 (215.2-353.9) | 396.4 (315.6-477.2) | 365.8 (289.0-442.6) | 429.8 (347.4-512.2) | 17.4 (8.4-26.3) | 20.1 (10.9-29.3) | 22.5 (12.6-32.4) | 70.2 (52.8-87.6) | 97.0 (76.9-117.0) | 89.6 (70.5-108.8) | 106.8 (85.9-127.7) |
|  | Total | 73.41 (44.80-107.67) | 166.2 (152.7-179.7) | 93.2 (-43.3-229.8) | 41.2 (22.9-59.4) | 56.9 (35.8-78.0) | 75.1 (50.8-99.4) | 194.0 (155.4-232.6) | 241.9 (199.2-284.5) | 239.7 (197.7-281.8) | 291.9 (245.8-337.9) | 10.3 (5.5-15.0) | 13.9 (8.6-19.1) | 18.6 (12.4-24.7) | 49.9 (39.8-60.0) | 62.3 (51.1-73.5) | 61.2 (50.2-72.1) | 436.5 (-519.3-1392.3) |
| Huila | Men | 19.01 (-0.09-41.75) | 260.5 (254.2-266.8) | 440.2 (335.4-545.0) | 126.5 (114.7-138.3) | 202.6 (187.8-217.4) | 189.3 (174.9-203.6) | 249.3 (233.0-265.6) | 294.4 (276.9-311.9) | 404.0 (383.7-424.4) | 339.6 (321.1-358.0) | 120.9 (-21.0-262.7) | 361.6 (102.2-621.0) | 428.1 (159.3-696.9) | 529.2 (228.2-830.2) | 635.5 (301.9-969.0) | 637.9 (302.7-973.0) | 368.3 (115.3-621.2) |
|  | Women | 27.06 (13.03-42.84) | 491.4 (482.9-499.8) | 560.5 (459.1-661.9) | 252.5 (236.1-268.9) | 391.0 (370.8-411.3) | 372.6 (352.8-392.4) | 479.9 (457.6-502.1) | 536.0 (512.7-559.2) | 767.4 (739.9-794.9) | 607.6 (583.3-631.9) | 151.5 (24.2-278.8) | 366.6 (142.8-590.4) | 554.5 (277.9-831.1) | 558.0 (290.1-826.0) | 778.2 (456.2-1100.2) | 659.2 (385.0-933.4) | 855.6 (523.1-1188.1) |
|  | Total | 24.01 (8.49-41.75) | 377.8 (372.5-383.1) | 500.4 (427.4-573.3) | 190.5 (180.3-200.6) | 298.3 (285.7-310.9) | 282.4 (270.1-294.7) | 366.5 (352.6-380.3) | 417.2 (402.6-431.8) | 588.8 (571.6-606.0) | 475.9 (460.6-491.3) | 136.2 (40.9-231.5) | 364.1 (192.8-535.4) | 491.3 (298.4-684.1) | 543.6 (342.1-745.1) | 706.8 (475.0-938.7) | 648.5 (432.0-865.0) | 611.9 (403.1-820.8) |
| La Guajira | Men | 89.41 (50.55-138.29) | 117.8 (112.7-122.8) | 161.5 (62.6-260.3) | 35.8 (28.0-43.6) | 62.1 (52.0-72.2) | 94.2 (81.8-106.7) | 136.8 (122.2-151.4) | 131.3 (117.4-145.1) | 162.0 (146.9-177.2) | 177.6 (162.0-193.1) | 9.5 (7.4-11.7) | 16.6 (13.9-19.4) | 98.5 (-101.4-298.3) | 36.2 (32.2-40.2) | 109.7 (-87.1-306.6) | 480.6 (3.8-957.4) | 379.1 (-36.2-794.4) |
|  | Women | 13.07 (-5.23-34.91) | 257.5 (250.3-264.6) | 242.8 (152.6-333.0) | 98.1 (85.7-110.5) | 135.0 (120.7-149.2) | 154.9 (139.7-170.1) | 269.5 (250.0-288.9) | 259.7 (241.1-278.3) | 391.9 (369.5-414.3) | 427.2 (404.2-450.1) | 74.4 (-58.3-207.2) | 327.3 (3.0-651.6) | 290.7 (12.9-568.5) | 234.3 (11.7-456.9) | 202.5 (0.3-404.7) | 269.4 (43.1-495.8) | 300.9 (61.8-539.9) |
|  | Total | 31.24 (10.58-55.76) | 191.0 (186.5-195.4) | 202.1 (135.2-269.0) | 68.3 (60.8-75.7) | 100.1 (91.2-108.9) | 126.1 (116.2-136.0) | 206.4 (194.1-218.8) | 198.7 (186.9-210.5) | 282.7 (268.9-296.5) | 308.6 (294.4-322.7) | 42.0 (-24.4-108.4) | 172.0 (9.8-334.1) | 194.6 (23.5-365.7) | 135.2 (23.9-246.5) | 156.1 (15.0-297.2) | 375.0 (111.1-638.9) | 340.0 (100.4-579.6) |
| Magdalena | Men | 32.82 (22.54-43.97) | 190.7 (185.8-195.6) | 229.8 (154.6-304.9) | 63.6 (55.8-71.3) | 95.0 (85.6-104.4) | 167.4 (154.9-180.0) | 213.2 (199.3-227.1) | 229.5 (215.4-243.6) | 238.4 (224.3-252.5) | 302.1 (286.4-317.9) | 102.4 (-34.0-238.8) | 80.2 (-29.3-189.8) | 177.3 (6.8-347.7) | 155.3 (5.5-305.0) | 273.4 (58.6-488.1) | 352.6 (102.5-602.8) | 467.2 (173.7-760.8) |
|  | Women | 33.30 (21.52-46.22) | 345.3 (338.8-351.8) | 485.2 (388.3-582.1) | 125.2 (114.4-135.9) | 176.4 (163.7-189.0) | 258.9 (243.5-274.2) | 392.2 (373.7-410.7) | 389.1 (371.0-407.1) | 458.5 (439.2-477.8) | 563.0 (541.9-584.1) | 194.3 (21.8-366.9) | 157.3 (11.3-303.2) | 330.8 (120.9-540.8) | 588.4 (302.4-874.3) | 508.3 (248.5-768.2) | 739.5 (423.1-1055.9) | 877.7 (538.2-1217.2) |
|  | Total | 33.15 (23.35-43.73) | 269.2 (265.1-273.3) | 357.5 (296.2-418.8) | 94.7 (88.1-101.4) | 136.2 (128.3-144.0) | 213.9 (204.0-223.8) | 304.2 (292.6-315.8) | 310.6 (299.1-322.1) | 350.3 (338.2-362.3) | 434.7 (421.5-447.9) | 148.4 (38.4-258.3) | 118.7 (27.5-210.0) | 254.0 (118.8-389.3) | 371.8 (210.4-533.2) | 390.8 (222.3-559.4) | 546.1 (344.4-747.7) | 672.5 (448.0-896.9) |
| Meta | Men | 37.03 (-2.17-91.95) | 223.4 (217.6-229.2) | 266.1 (179.2-353.0) | 59.3 (51.1-67.4) | 90.4 (80.4-100.3) | 164.4 (150.9-177.8) | 272.5 (255.4-289.5) | 273.9 (257.1-290.7) | 333.5 (315.1-351.8) | 340.9 (322.4-359.3) | 187.8 (-22.9-398.5) | 22.3 (19.8-24.7) | 109.1 (-23.8-242.0) | 396.3 (108.7-683.8) | 422.9 (128.2-717.5) | 387.5 (113.4-661.7) | 336.9 (85.3-588.4) |
|  | Women | 38.94 (19.54-61.49) | 435.1 (427.0-443.3) | 633.3 (500.7-765.8) | 152.1 (139.0-165.3) | 199.6 (184.8-214.5) | 269.9 (252.6-287.2) | 521.6 (497.9-545.3) | 531.6 (508.1-555.1) | 635.6 (610.3-660.9) | 670.1 (644.4-695.8) | 223.7 (-3.4-450.8) | 138.9 (-14.8-292.5) | 395.5 (107.8-683.2) | 903.1 (469.6-1336.5) | 749.9 (374.2-1125.5) | 911.5 (499.2-1323.8) | 1110.4 (658.9-1562.0) |
|  | Total | 36.87 (12.97-65.83) | 329.0 (324.0-334.0) | 449.7 (370.4-528.9) | 105.4 (97.7-113.1) | 144.7 (135.8-153.6) | 216.8 (205.8-227.7) | 396.5 (381.9-411.1) | 402.5 (388.1-417.0) | 484.7 (469.0-500.4) | 506.1 (490.2-521.9) | 205.8 (50.9-360.6) | 80.6 (3.7-157.4) | 252.3 (93.8-410.8) | 649.7 (389.6-909.7) | 586.4 (347.6-825.1) | 649.5 (402.0-897.1) | 723.6 (465.2-982.1) |
| Nariño | Men | 11.70 (-5.39-31.89) | 297.2 (291.7-302.6) | 322.1 (257.3-386.9) | 157.2 (146.7-167.7) | 215.3 (203.1-227.6) | 245.4 (232.1-258.8) | 414.2 (397.0-431.4) | 422.4 (405.2-439.5) | 422.4 (405.3-439.4) | 204.8 (193.0-216.6) | 136.1 (33.6-238.6) | 255.4 (108.5-402.3) | 275.6 (110.8-440.5) | 407.1 (213.8-600.5) | 499.2 (282.8-715.7) | 473.1 (264.6-681.5) | 208.1 (71.3-344.9) |
|  | Women | 8.67 (-4.25-23.33) | 690.3 (682.3-698.3) | 530.3 (465.6-595.0) | 450.3 (433.2-467.5) | 546.0 (527.3-564.8) | 573.8 (554.2-593.4) | 989.0 (963.5-1014.5) | 928.9 (904.4-953.4) | 928.3 (903.9-952.6) | 419.3 (403.1-435.5) | 314.9 (180.7-449.1) | 407.3 (254.3-560.2) | 412.4 (259.7-565.2) | 719.4 (521.5-917.3) | 798.9 (586.3-1011.4) | 662.4 (479.0-845.7) | 396.9 (246.8-546.9) |
|  | Total | 9.70 (-4.48-26.00) | 501.1 (496.2-506.0) | 426.2 (380.4-472.0) | 308.9 (298.7-319.1) | 386.6 (375.2-398.0) | 416.1 (404.1-428.2) | 712.8 (697.2-728.4) | 685.4 (670.2-700.5) | 684.9 (669.9-700.0) | 316.1 (305.9-326.3) | 225.5 (141.1-309.9) | 331.3 (225.3-437.4) | 344.0 (231.7-456.4) | 563.3 (424.9-701.6) | 649.1 (497.4-800.8) | 567.7 (428.9-706.5) | 302.5 (200.9-404.0) |
| Norte de Santander | Men | 33.76 (14.74-55.93) | 265.3 (260.0-270.6) | 297.8 (224.5-371.2) | 86.7 (78.3-95.1) | 144.1 (133.4-154.8) | 198.0 (185.6-210.4) | 287.5 (272.9-302.1) | 310.2 (295.3-325.0) | 402.4 (385.7-419.0) | 377.7 (361.7-393.7) | 88.6 (-16.2-193.4) | 84.5 (-4.5-173.6) | 282.7 (81.1-484.2) | 380.8 (155.0-606.6) | 414.7 (186.0-643.4) | 443.7 (212.3-675.2) | 389.9 (172.9-607.0) |
|  | Women | 29.65 (18.11-42.32) | 534.5 (527.2-541.8) | 537.7 (457.2-618.2) | 184.4 (172.5-196.3) | 311.1 (295.8-326.4) | 382.0 (365.3-398.7) | 565.3 (545.4-585.2) | 613.7 (593.5-634.0) | 807.7 (784.7-830.6) | 778.9 (756.5-801.2) | 156.0 (36.9-275.1) | 302.3 (133.8-470.8) | 438.2 (240.2-636.1) | 624.5 (392.1-856.9) | 565.0 (352.4-777.5) | 890.2 (616.9-1163.5) | 787.5 (538.4-1036.6) |
|  | Total | 30.70 (18.14-44.61) | 403.9 (399.3-408.4) | 417.8 (363.3-472.2) | 137.0 (129.6-144.3) | 230.1 (220.6-239.5) | 293.0 (282.5-303.5) | 430.7 (418.2-443.1) | 466.4 (453.7-479.1) | 610.7 (596.4-625.0) | 583.8 (569.9-597.6) | 122.3 (43.0-201.6) | 193.4 (98.1-288.7) | 360.4 (219.2-501.7) | 502.7 (340.7-664.7) | 489.8 (333.7-645.9) | 667.0 (487.9-846.0) | 588.7 (423.5-753.9) |
| Putumayo | Men | 1.06 (-15.41-20.74) | 171.8 (162.7-180.9) | 228.4 (80.3-376.6) | 78.4 (61.6-95.1) | 108.5 (89.1-128.0) | 121.3 (100.6-142.1) | 233.2 (204.9-261.6) | 251.0 (222.1-279.9) | 244.0 (215.9-272.2) | 154.9 (132.8-177.0) | 118.4 (-151.8-388.6) | 351.1 (-158.5-860.7) | 149.9 (-174.3-474.1) | 320.1 (-157.0-797.1) | 311.6 (-137.5-760.7) | 198.3 (-140.3-536.9) | 149.8 (-155.6-455.2) |
|  | Women | 16.40 (-12.04-54.02) | 360.0 (346.9-373.2) | 331.0 (161.8-500.2) | 193.6 (167.2-220.0) | 283.2 (251.7-314.6) | 254.1 (224.2-284.0) | 493.4 (452.4-534.3) | 453.6 (414.9-492.2) | 503.5 (463.5-543.6) | 314.9 (283.7-346.1) | 165.6 (-153.5-484.7) | 330.5 (-142.7-803.7) | 63.1 (55.6-70.6) | 469.1 (-75.2-1013.4) | 611.7 (-27.5-1250.8) | 390.2 (-68.5-848.8) | 286.9 (-116.2-690.0) |
|  | Total | 9.20 (-11.19-34.27) | 266.3 (258.3-274.3) | 279.7 (167.3-392.2) | 135.7 (120.1-151.3) | 195.5 (177.0-213.9) | 188.1 (169.9-206.3) | 364.1 (339.1-389.0) | 352.9 (328.8-377.1) | 374.8 (350.3-399.3) | 235.6 (216.4-254.8) | 142.0 (-67.1-351.0) | 340.8 (-6.9-688.5) | 106.5 (-55.6-268.7) | 394.6 (32.7-756.5) | 461.6 (71.0-852.2) | 294.2 (9.2-579.3) | 218.4 (-34.5-471.2) |
| Quindío | Men | 27.95 (22.18-33.99) | 435.7 (424.7-446.7) | 477.7 (357.2-598.2) | 156.2 (138.6-173.9) | 215.5 (194.9-236.2) | 288.8 (264.8-312.7) | 483.7 (452.8-514.5) | 580.6 (547.1-614.1) | 697.1 (660.7-733.6) | 603.2 (569.4-637.0) | 215.3 (-19.7-450.3) | 229.9 (-5.3-465.1) | 307.2 (53.6-560.8) | 438.1 (145.3-730.9) | 650.0 (283.4-1016.6) | 726.8 (342.2-1111.5) | 776.6 (366.1-1187.2) |
|  | Women | 15.86 (-0.82-35.36) | 942.5 (927.1-957.8) | 812.6 (677.6-947.6) | 383.1 (356.8-409.5) | 526.3 (495.7-557.0) | 628.2 (594.7-661.7) | 1122.2 (1077.7-1166.7) | 1169.3 (1124.2-1214.4) | 1418.0 (1368.6-1467.4) | 1300.9 (1253.8-1348.0) | 453.7 (143.2-764.2) | 630.7 (264.1-997.3) | 380.8 (160.0-601.7) | 807.4 (474.8-1140.0) | 1215.7 (780.5-1650.9) | 1523.5 (1034.5-2012.4) | 676.4 (403.1-949.8) |
|  | Total | 20.61 (8.59-33.96) | 702.4 (692.7-712.0) | 645.1 (554.7-735.6) | 275.5 (259.3-291.7) | 379.0 (360.2-397.9) | 467.6 (446.6-488.6) | 819.9 (792.3-847.5) | 890.4 (861.9-919.0) | 1076.4 (1045.2-1107.6) | 970.3 (940.8-999.8) | 334.5 (139.8-529.2) | 430.3 (212.5-648.1) | 344.0 (175.9-512.1) | 622.7 (401.2-844.3) | 932.8 (648.3-1217.3) | 1125.1 (814.1-1436.2) | 726.5 (479.9-973.2) |
| Risaralda | Men | 36.04 (14.32-61.89) | 566.1 (556.5-575.8) | 673.2 (552.4-793.9) | 235.8 (219.1-252.5) | 276.7 (258.7-294.7) | 376.4 (355.3-397.5) | 619.8 (593.0-646.6) | 741.3 (712.3-770.3) | 854.2 (823.3-885.1) | 818.6 (788.6-848.7) | 242.3 (39.6-444.9) | 230.9 (39.2-422.6) | 226.5 (65.4-387.5) | 720.6 (393.3-1047.9) | 1267.6 (818.4-1716.9) | 1235.7 (798.9-1672.5) | 788.5 (455.2-1121.9) |
|  | Women | 33.54 (21.97-46.20) | 1253.7 (1240.2-1267.2) | 1164.0 (1037.1-1290.8) | 614.3 (588.9-639.7) | 686.0 (659.4-712.7) | 904.5 (873.9-935.2) | 1363.2 (1325.9-1400.4) | 1522.7 (1483.7-1561.6) | 1793.9 (1751.9-1835.9) | 1803.8 (1762.0-1845.6) | 459.9 (232.2-687.6) | 547.3 (295.1-799.4) | 477.2 (291.3-663.1) | 1173.2 (843.6-1502.8) | 1640.4 (1239.1-2041.7) | 1803.5 (1391.4-2215.7) | 2046.3 (1600.6-2492.0) |
|  | Total | 34.65 (19.81-51.33) | 931.6 (923.1-940.1) | 918.6 (831.0-1006.1) | 436.2 (420.6-451.8) | 493.9 (477.5-510.4) | 657.6 (638.5-676.6) | 1015.5 (992.0-1038.9) | 1157.0 (1132.2-1181.8) | 1354.1 (1327.5-1380.7) | 1342.8 (1316.5-1369.1) | 351.1 (198.7-503.5) | 389.1 (230.7-547.4) | 351.8 (228.8-474.8) | 946.9 (714.7-1179.2) | 1454.0 (1152.9-1755.2) | 1519.6 (1219.4-1819.9) | 1417.4 (1139.1-1695.7) |
| Santander | Men | 30.63 (20.26-41.89) | 446.2 (440.6-451.8) | 610.0 (528.6-691.4) | 188.2 (178.3-198.1) | 272.2 (260.4-284.0) | 330.6 (317.6-343.6) | 447.9 (433.0-462.9) | 548.5 (532.2-564.7) | 642.7 (625.3-660.1) | 643.8 (626.5-661.1) | 205.5 (79.6-331.3) | 367.7 (196.6-538.8) | 324.6 (171.3-477.9) | 568.6 (361.9-775.3) | 965.0 (691.8-1238.3) | 845.5 (595.7-1095.3) | 993.0 (716.5-1269.5) |
|  | Women | 34.82 (25.23-45.14) | 862.5 (855.0-870.0) | 1110.5 (1022.5-1198.5) | 377.8 (364.3-391.3) | 525.4 (509.6-541.2) | 629.3 (612.0-646.6) | 849.5 (829.7-869.3) | 1008.6 (987.3-1029.8) | 1247.6 (1224.2-1271.1) | 1307.3 (1283.5-1331.1) | 355.3 (219.0-491.6) | 481.0 (323.6-638.3) | 662.6 (484.3-840.9) | 1003.7 (784.4-1223.0) | 1797.0 (1494.8-2099.2) | 1664.1 (1382.3-1945.9) | 1810.0 (1517.9-2102.2) |
|  | Total | 33.19 (23.82-43.27) | 661.8 (657.1-666.6) | 860.3 (800.3-920.2) | 286.6 (278.1-295.1) | 403.6 (393.6-413.6) | 485.4 (474.5-496.4) | 655.9 (643.3-668.4) | 786.5 (773.0-800.0) | 955.6 (940.9-970.4) | 987.0 (972.1-1001.9) | 280.4 (187.6-373.2) | 424.3 (308.1-540.5) | 493.6 (376.0-611.2) | 786.1 (635.5-936.8) | 1381.0 (1177.3-1584.7) | 1254.8 (1066.5-1443.1) | 1401.5 (1200.4-1602.6) |
| Sucre | Men | 22.43 (9.38-37.04) | 215.1 (208.8-221.3) | 329.7 (232.6-426.7) | 63.9 (54.7-73.1) | 86.8 (76.2-97.5) | 153.5 (139.2-167.7) | 189.2 (173.6-204.7) | 217.5 (201.1-233.9) | 291.9 (273.1-310.6) | 466.5 (443.0-489.9) | 158.7 (-16.1-333.5) | 171.1 (-11.9-354.1) | 300.3 (47.8-552.9) | 335.0 (73.7-596.4) | 305.2 (62.6-547.8) | 272.9 (52.6-493.2) | 764.6 (367.0-1162.1) |
|  | Women | 30.83 (13.26-51.12) | 491.4 (482.0-500.7) | 669.8 (549.2-790.4) | 170.3 (155.3-185.3) | 204.3 (188.1-220.6) | 342.1 (320.9-363.2) | 426.3 (403.2-449.5) | 463.4 (439.6-487.1) | 680.4 (652.1-708.8) | 1061.7 (1026.7-1096.7) | 155.6 (0.6-310.6) | 490.6 (189.5-791.6) | 543.5 (241.2-845.9) | 652.0 (331.1-972.9) | 705.3 (378.7-1031.8) | 586.9 (310.8-862.9) | 1555.0 (1085.2-2024.7) |
|  | Total | 27.03 (12.89-42.95) | 354.2 (348.6-359.8) | 499.8 (422.4-577.2) | 117.3 (108.5-126.1) | 145.8 (136.1-155.5) | 248.3 (235.6-261.1) | 308.6 (294.6-322.6) | 341.4 (327.0-355.9) | 488.0 (470.9-505.0) | 767.2 (746.1-788.4) | 157.2 (40.3-274.0) | 330.8 (154.7-507.0) | 421.9 (225.0-618.9) | 493.5 (286.6-700.4) | 505.2 (301.8-708.7) | 429.9 (253.3-606.5) | 1159.8 (852.1-1467.5) |
| Tolima | Men | 40.01 (27.17-54.15) | 332.0 (325.8-338.3) | 351.6 (287.0-416.2) | 111.2 (101.6-120.9) | 179.2 (167.1-191.4) | 250.3 (235.9-264.8) | 391.2 (373.3-409.2) | 424.7 (406.1-443.2) | 464.7 (445.5-484.0) | 485.7 (466.1-505.3) | 91.4 (7.6-175.1) | 115.0 (26.6-203.3) | 239.6 (99.4-379.8) | 370.7 (197.8-543.5) | 491.3 (288.8-693.8) | 601.3 (372.6-830.1) | 552.0 (335.5-768.6) |
|  | Women | 24.28 (17.60-31.34) | 687.9 (679.1-696.7) | 722.8 (639.4-806.1) | 281.8 (266.8-296.9) | 413.3 (395.2-431.4) | 513.1 (492.8-533.3) | 768.7 (744.2-793.3) | 794.1 (769.3-818.9) | 971.6 (944.3-998.8) | 1035.3 (1007.4-1063.3) | 295.1 (140.8-449.3) | 457.4 (265.6-649.3) | 526.4 (338.6-714.2) | 749.5 (530.2-968.8) | 818.6 (587.0-1050.1) | 1185.8 (904.4-1467.1) | 1026.7 (774.6-1278.8) |
|  | Total | 28.57 (21.06-36.54) | 514.2 (508.8-519.6) | 537.2 (484.5-589.9) | 198.4 (189.4-207.4) | 298.9 (287.9-309.9) | 385.0 (372.5-397.5) | 584.6 (569.3-599.9) | 613.8 (598.2-629.4) | 724.2 (707.4-741.1) | 767.2 (750.0-784.4) | 193.2 (105.5-281.0) | 286.2 (180.6-391.8) | 383.0 (265.8-500.2) | 560.1 (420.4-699.7) | 654.9 (501.1-808.7) | 893.6 (712.3-1074.9) | 789.3 (623.2-955.5) |
| Valle del Cauca | Men | 26.54 (18.69-34.91) | 386.7 (383.0-390.5) | 553.6 (501.9-605.3) | 186.8 (179.8-193.8) | 217.9 (210.3-225.4) | 248.5 (240.6-256.5) | 410.7 (400.6-420.8) | 425.1 (415.0-435.3) | 600.8 (588.8-612.8) | 577.1 (565.4-588.8) | 222.7 (130.8-314.6) | 326.3 (213.8-438.9) | 335.0 (229.6-440.5) | 598.9 (457.7-740.0) | 691.5 (539.6-843.5) | 901.0 (728.3-1073.7) | 799.6 (638.2-961.0) |
|  | Women | 25.62 (17.78-33.98) | 807.2 (802.3-812.2) | 942.2 (889.5-994.9) | 460.8 (450.5-471.1) | 510.7 (499.9-521.5) | 537.6 (526.8-548.4) | 838.9 (825.5-852.3) | 817.6 (804.5-830.7) | 1223.6 (1207.6-1239.5) | 1184.8 (1169.2-1200.4) | 412.1 (310.8-513.4) | 574.1 (451.9-696.4) | 542.9 (440.5-645.4) | 931.2 (796.3-1066.1) | 1197.8 (1040.8-1354.8) | 1584.1 (1406.5-1761.8) | 1352.8 (1191.6-1513.9) |
|  | Total | 25.95 (18.22-34.19) | 612.0 (608.8-615.2) | 747.9 (710.9-784.8) | 333.0 (326.6-339.4) | 374.4 (367.6-381.1) | 403.8 (396.9-410.6) | 640.5 (631.9-649.1) | 635.5 (627.1-644.0) | 934.6 (924.4-944.8) | 902.8 (892.8-912.8) | 317.4 (249.0-385.8) | 450.2 (367.2-533.3) | 439.0 (365.5-512.5) | 765.0 (667.4-862.7) | 944.7 (835.4-1053.9) | 1242.6 (1118.7-1366.4) | 1076.2 (962.2-1190.2) |
| Vaupés | Men | 57.08 (27.86-92.97) | 60.5 (42.3-78.6) | 15.6 (10.8-20.4) | 7.3 (-10.4-25.0) | 11.4 (-10.2-33.1) | 42.9 (1.7-84.1) | 81.5 (25.8-137.3) | 57.6 (11.7-103.6) | 48.9 (7.2-90.6) | 154.9 (81.6-228.1) | 2.1 (-3.0-7.1) | 2.6 (-2.4-7.6) | 11.0 (0.3-21.7) | 23.5 (7.2-39.7) | 15.8 (2.9-28.8) | 14.5 (2.0-27.0) | 39.5 (20.1-58.9) |
|  | Women | 59.18 (21.85-107.93) | 105.6 (79.5-131.8) | 26.0 (19.4-32.7) | 21.1 (-11.7-53.8) | 7.4 (-11.6-26.3) | 61.1 (7.2-114.9) | 107.0 (37.2-176.7) | 170.4 (84.3-256.6) | 81.7 (23.2-140.2) | 254.9 (153.0-356.8) | 5.8 (-3.3-14.9) | 2.1 (-3.2-7.4) | 14.9 (1.4-28.4) | 27.5 (9.1-46.0) | 45.9 (22.3-69.5) | 21.4 (5.6-37.1) | 64.5 (37.5-91.4) |
|  | Total | 57.66 (25.78-97.63) | 81.1 (65.6-96.6) | 20.8 (16.7-24.9) | 13.6 (-4.2-31.4) | 9.6 (-5.0-24.2) | 51.1 (17.9-84.4) | 93.1 (49.2-137.1) | 109.2 (62.6-155.8) | 64.0 (28.9-99.1) | 200.8 (139.5-262.1) | 3.9 (-1.2-9.1) | 2.3 (-1.3-6.0) | 12.9 (4.3-21.6) | 25.5 (13.2-37.8) | 30.9 (17.4-44.3) | 17.9 (7.9-28.0) | 52.0 (35.4-68.6) |
| Vichada | Men | 81.95 (-12.50-278.35) | 48.2 (39.3-57.0) | 152.4 (-229.2-534.1) | 3.1 (-3.2-9.4) | 26.5 (8.2-44.8) | 28.1 (9.3-46.9) | 70.1 (41.5-98.7) | 71.2 (43.4-99.0) | 56.8 (32.7-80.9) | 67.0 (41.6-92.5) | 0.6 (-0.6-1.8) | 6.6 (1.8-11.4) | 7.3 (2.1-12.5) | 18.1 (10.4-25.7) | 1002.5 (-1669.1-3674.2) | 14.6 (8.3-21.0) | 17.4 (10.6-24.1) |
|  | Women | 46.87 (17.57-83.48) | 110.5 (95.8-125.1) | 27.6 (23.8-31.5) | 9.6 (-2.6-21.8) | 64.3 (33.3-95.4) | 70.1 (37.5-102.8) | 128.0 (85.6-170.4) | 169.7 (122.7-216.7) | 129.1 (89.4-168.8) | 166.5 (122.8-210.2) | 2.6 (-0.7-6.0) | 15.6 (7.7-23.5) | 19.4 (9.7-29.1) | 33.2 (21.9-44.6) | 45.5 (32.5-58.5) | 35.2 (24.0-46.5) | 41.8 (30.4-53.2) |
|  | Total | 65.12 (-4.01-184.07) | 76.5 (68.3-84.8) | 90.0 (-100.8-280.9) | 6.1 (-0.5-12.6) | 43.8 (26.5-61.1) | 47.1 (29.1-65.1) | 96.3 (71.5-121.1) | 116.0 (89.8-142.2) | 89.8 (67.4-112.1) | 112.5 (88.2-136.8) | 1.6 (-0.2-3.4) | 11.1 (6.5-15.7) | 13.3 (7.8-18.8) | 25.6 (18.8-32.5) | 524.0 (-811.8-1859.9) | 24.9 (18.5-31.4) | 29.6 (22.9-36.2) |
| ^a^ Archipiélago de San Andrés, Providencia y Santa Catalina | | | | | | | | | | | | | | | | | | |
