# Supplementary material for: Burden of anxiety disorders in Colombia: subnational DALY estimates and territorial inequalities, 2016–2022
Source: Popul Health Metr. 2026 Sep 15;24:55. doi: 10.1186/s12963-026-00510-4 (PMC13591821; doi:10.1186/s12963-026-00510-4)
Supplement: Supplementary file 3 [file 12963_2026_510_MOESM3_ESM.docx]

## Supplementary Table S3.

## Comparison of cumulative age-standardized DALY rates and annual percentage change (APC) in anxiety disorders across Colombian departments, Colombia, 2016–2022

| Rank | Department | Cumulative age-standardized DALY rate (per 100,000) | APC (%) | 95% CI | Trend |
| --- | --- | --- | --- | --- | --- |
| 1 | Archipiélago | 422.2 | 90.26 | -2.72 to 272.10 | Not significant |
| 2 | Chocó | 244.0 | 89.70 | 38.95 to 159.00 | Increasing |
| 3 | Arauca | 322.5 | 79.34 | 47.08 to 118.68 | Increasing |
| 4 | Guaviare | 93.2 | 73.41 | 44.80 to 107.67 | Increasing |
| 5 | Vichada | 90.0 | 65.12 | -4.01 to 184.07 | Not significant |
| 6 | Guainía | 27.2 | 64.62 | 21.33 to 123.35 | Increasing |
| 7 | Vaupés | 20.8 | 57.66 | 25.78 to 97.63 | Increasing |
| 8 | Bolívar | 521.2 | 37.03 | 23.57 to 51.95 | Increasing |
| 9 | Meta | 449.7 | 36.87 | 12.97 to 65.83 | Increasing |
| 10 | Boyacá | 431.7 | 36.86 | 25.59 to 49.15 | Increasing |
| 11 | Risaralda | 918.6 | 34.65 | 19.81 to 51.33 | Increasing |
| 12 | Córdoba | 303.2 | 33.25 | 28.58 to 38.08 | Increasing |
| 13 | Santander | 860.3 | 33.19 | 23.82 to 43.27 | Increasing |
| 14 | Magdalena | 357.5 | 33.15 | 23.35 to 43.73 | Increasing |
| 15 | La Guajira | 202.1 | 31.24 | 10.58 to 55.76 | Increasing |
| 16 | Caldas | 831.0 | 30.75 | 15.49 to 48.02 | Increasing |
| 17 | Norte de Santander | 417.8 | 30.70 | 18.14 to 44.61 | Increasing |
| 18 | Cauca | 384.5 | 30.16 | 24.83 to 35.72 | Increasing |
| 19 | Tolima | 537.2 | 28.57 | 21.06 to 36.54 | Increasing |
| 20 | Sucre | 499.8 | 27.03 | 12.89 to 42.95 | Increasing |
| 21 | Valle del Cauca | 747.9 | 25.95 | 18.22 to 34.19 | Increasing |
| 22 | Huila | 500.4 | 24.01 | 8.49 to 41.75 | Increasing |
| 23 | Amazonas | 37.1 | 21.11 | 8.66 to 34.98 | Increasing |
| 24 | Atlántico | 650.5 | 21.07 | 17.02 to 25.25 | Increasing |
| 25 | Quindío | 645.1 | 20.61 | 8.59 to 33.96 | Increasing |
| 26 | Caquetá | 217.0 | 19.64 | -9.18 to 57.60 | Not significant |
| 27 | Bogotá, D.C. | 864.4 | 17.29 | 6.80 to 28.81 | Increasing |
| 28 | Cesar | 509.7 | 17.23 | 8.87 to 26.24 | Increasing |
| 29 | Cundinamarca | 500.6 | 16.03 | 10.01 to 22.39 | Increasing |
| 30 | Antioquia | 728.1 | 13.60 | 2.00 to 26.53 | Increasing |
| 31 | Nariño | 426.2 | 9.70 | -4.48 to 26.00 | Not significant |
| 32 | Putumayo | 279.7 | 9.20 | -11.19 to 34.27 | Not significant |
| 33 | Casanare | 474.7 | -11.49 | -29.78 to 11.56 | Not significant |

Abbreviations: APC, annual percentage change; CI, confidence interval; DALY, disability-adjusted life year.

Departments are ranked according to the annual percentage change (APC) in age-standardized DALY rates. Trends were classified as Increasing when the 95% CI excluded zero, Not significant when the 95% CI included zero, and Decreasing when the entire 95% CI was below zero.
