# Supplementary material for: Burden of anxiety disorders in Colombia: subnational DALY estimates and territorial inequalities, 2016–2022
Source: Popul Health Metr. 2026 Sep 15;24:55. doi: 10.1186/s12963-026-00510-4 (PMC13591821; doi:10.1186/s12963-026-00510-4)
Supplement: Supplementary file 4 [file 12963_2026_510_MOESM4_ESM.docx]

**Supplementary table 3. GATHER**

| **Item** | **recommendation** | **Compliance statement** |
| --- | --- | --- |
| **1** | Define the indicator(s), the population (including age, sex, and geographical data), and the period(s) for which the estimates were produced. | Disability-Adjusted Life Years (DALYs) were used as the primary indicator, defined as the sum of Years Lived with Disability (YLDs) and Years of Life Lost (YLLs). The study population included adults (≥18 years) residing in Colombia between 2016 and 2022, stratified by sex, age group, department, and Bogotá D.C. Estimates were produced at national and subnational levels for anxiety disorders (ICD-10 codes F40–F44), consistent with the WHO Global Health Estimates (GHE) classification. |
| **2** | State the funding sources for the activity. | This study was funded by the Ministry of Science, Technology and Innovation of Colombia (MINCIENCIAS) and Universidad de los Llanos through the project “Development of a Mental Health, Family and Social Coexistence Observatory…” (Code 112291891873; Contract 655/2022). Funders had no role in study design, analysis, interpretation, or publication decisions. |
| **3** | Describe how the data were identified and obtained. | Morbidity data were obtained from the Colombian Individual Health Service Provision Registry (RIPS) and mortality data from the National Vital Statistics System (RUAF), both available through SISPRO. Population denominators were obtained from the National Administrative Department of Statistics (DANE). Administrative datasets were cleaned, validated, harmonized, and integrated into analytical datasets using reproducible Python scripts. |
| **4** | Specify inclusion and exclusion criteria, and all specific exclusions. | All records corresponding to anxiety disorders (ICD-10 codes F40–F44) between 2016 and 2022 were included. Eligible records required valid information on year, sex, age, and department. Records with inconsistent or incomplete information were excluded during data cleaning. |
| **5** | Provide information on all data sources and their main characteristics. For each source used, include reference information or institutional contact, the represented population, data collection method, year(s) of data collection, sex and age range, diagnostic criteria or measurement method, and sample size, if applicable. | Data sources included RIPS (morbidity), RUAF (mortality), and DANE population projections. Variables included sex, single-year age, year, department, and ICD-10 diagnosis. Analyses were restricted to adults (≥18 years. Age-standardized rates were calculated using the WHO world standard population. |
| **6** | Mention and describe all types of input data that may be subject to substantial bias | Administrative healthcare databases may be affected by healthcare access, underreporting, diagnostic variability, reporting quality, and territorial differences in service availability. Morbidity estimates represent treated cases identified through healthcare utilization rather than the true prevalence of anxiety disorders. Mortality estimates were based on the underlying cause of death following the WHO GHE methodology. Residual bias may remain |
| **7** | List any other input data and specify their source. | Population denominators were obtained from official DANE projections. Age standardization used the WHO world standard population. |
| **8** | Provide all input data in a file from which data can be efficiently extracted (e.g., preferably in a spreadsheet rather than a PDF), including all relevant metadata specified in item 5. For data that cannot be publicly shared for ethical or legal reasons, such as third-party data, provide the name of the contact person or institution holding the rights. | Data are available through SISPRO under national regulations. Analytical datasets derived in this study are available from the authors upon reasonable request. |
| **9** | Provide a conceptual overview of the data analysis method; a diagram may be helpful | DALYs were estimated using the WHO Global Health Estimates (GHE) abbreviated methodology, integrating premature mortality (YLLs) and non-fatal health loss (YLDs). |
| **10** | Describe in detail all stages of data analysis, including the mathematical formulas used. This description should include, as applicable, data cleaning, preprocessing, adjustments, weighting of data sources, and the mathematical or statistical models applied. | DALYs were calculated as DALYs = YLDs + YLLs. YLDs were estimated using treated prevalence derived from unique individuals recorded in RIPS multiplied by disability weights from the Global Burden of Disease Study 2021. Morbidity estimates were based on unique individuals receiving healthcare rather than healthcare encounters. YLLs were calculated by multiplying deaths recorded in RUAF by the corresponding remaining life expectancy from the WHO GHE standard life table. No age-weighting or time discounting was applied. Crude and age-standardized DALY rates per 100,000 population were calculated using the WHO world standard population (2000–2025). Annual percentage change (APC) was estimated using a single log-linear regression model. |
| **11** | Describe how the different models were evaluated and how the model(s) used were selected. | The WHO GHE abbreviated approach was selected due to its applicability to administrative data and consistency with the WHO Global Health Estimates methodological framework for burden of disease estimation. |
| **12** | Report the results of model performance evaluation, if conducted, and the corresponding sensitivity analyses | No predictive models were used. Internal consistency checks and validation of aggregated totals across years and territories were performed. |
| **13** | Describe the methods used to calculate uncertainty in the estimates. Indicate which sources of uncertainty were considered in the analysis and which were not. | Uncertainty was quantified using 95% confidence intervals for crude and age-standardized DALY rates assuming a Poisson distribution of observed counts. Confidence intervals for age-standardized rates were derived using the normal approximation after direct standardization. |
| **14** | Indicate how to access the analytical or statistical source code used to produce the estimates. | Data processing and statistical analyses were performed using Microsoft Excel and Python 3.12. Figures were generated using Matplotlib, and thematic maps were produced in QGIS 3.44.11. Analytical code is available from the corresponding author upon reasonable request. |
| **15** | Provide the published estimates in a file format that allows efficient data extraction | Final estimates are provided in supplementary Excel files, including national and subnational DALYs and rates. |
| **16** | Provide a quantitative measure of the uncertainty of the estimates (e.g., confidence intervals). | All key estimates include 95% confidence intervals where applicable. |
| **17** | Interpret the results in light of the available evidence. If this is an update of previous estimates, describe the reasons for any changes | The findings indicate a substantial burden of anxiety disorders in Colombia, characterized by marked territorial inequalities, sex differences, and increasing temporal trends between 2016 and 2022, with disability accounting for nearly all DALYs. |
| **18** | Explain the limitations of the estimates. Present an analysis of the model assumptions and the data limitations that affect the interpretation of the estimates. | The study is limited by the use of secondary administrative healthcare databases, which capture treated cases rather than the true population prevalence of anxiety disorders. Potential sources of bias include underreporting, diagnostic variability, differences in healthcare access and reporting quality across departments, and residual uncertainty associated with Poisson-based confidence intervals, particularly in sparsely populated areas. APC estimates should be interpreted cautiously because only seven annual observations were available. The ecological design precludes causal inference. |
